# Innovative strategy for the conservation of a millennial mausoleum from biodeterioration through artificial light management

**DOI:** 10.1038/s41522-023-00438-9

**Published:** 2023-09-23

**Authors:** Yuanyuan Bao, Yan Ma, Wenjing Liu, Xin Li, Yonghui Li, Peng Zhou, Youzhi Feng, Manuel Delgado-Baquerizo

**Affiliations:** 1 Jiangsu Co-innovation Center of Efficient Processing and Utilization of Forest Resources, College of Chemical Engineering, Nanjing Forestry University, 210037 Nanjing, PR China; 2 School of Architecture, Southeast University, 210096 Nanjing, PR China; 3 Shandong Key Laboratory of Environmental Processes and Health, School of Environmental Science and Engineering, Shandong University, 266237 Qingdao, PR China; 4 Jiangning Cultural Heritage Protection Center, 211100 Nanjing, PR China; 5 State Key Laboratory of Soil and Sustainable Agriculture, Institute of Soil Science, Chinese Academy of Sciences, 210008 Nanjing, Jiangsu PR China; 6 Jiangsu Collaborative Innovation Center for Solid Organic Waste Resource Utilization, 210095 Nanjing, China; 7 Laboratorio de Biodiversidad y Funcionamiento Ecosistémico, Instituto de Recursos Naturales y Agrobiología de Sevilla (IRNAS), CSIC, Av. Reina Mercedes 10, E-41012 Sevilla, Spain

**Keywords:** Soil microbiology, Next-generation sequencing

## Abstract

Artificial lights can cause critical microbial biodeterioration of heritage monuments by promoting the outbreak of phototrophic microbiomes when they are used for touristic viewing. Here, with the ultimate aim of providing innovative solutions for the conservation and visiting of such monuments, we conducted a pioneering two-year in situ manipulative experiment to evaluate the impacts of different artificial light wavelengths (i.e., blue, green and red lights compared to white light) on the phototrophic microbiome of a millennial Chinese imperial mausoleum. Our results show that artificial light can shape the ecophysiological features of the phototrophic bacteriome in this monument and reduce its potential for further biodeterioration. In general, Cyanobacteria dominated (42.0% of the total relative abundance) the phototrophic bacteriome of this cultural relic; however, they were also very sensitive to the choice of artificial light. Compared to white light, monochromatic light, especially green light, reduced Cyanobacteria abundances (18.6%) by decreasing photosynthetic pigment abundances (42.9%); decreased the abundances of heterotrophic species belonging to Proteobacteria (4.5%) and the proportion of genes (6.1%) associated with carbon (i.e., carbon fixation), nitrogen (i.e., denitrification), and sulfur (i.e., dissimilatory sulfate reduction) cycling; and further decreased organic acid (10.1–14.1%) production of the phototrophic bacteriome, which is known to be involved in biodeterioration. Taken together, our findings constitute a major advancement in understanding how light wavelengths influence the phototrophic microbiome in cultural relics, and we found that artificial lights with certain wavelengths (e.g., green light) can help long-term conservation while allowing tourism activities.

## Introduction

Historical monuments are fundamental to preserving humanity for future generations. How to preserve this precious heritage left by human ancestors has been a continuous challenge for conservation scientists for decades^1^, especially when they are open to visitors. Tourism management plays a critical role in the future of many monuments by attracting tourists for the appreciation of their beauty and as the source of sustainable funding for their conservation. For example, illumination with artificial lights is essential for exhibiting cultural heritage and facilitating tourism in indoor environments. However, it is also an important inducer of the microbial-driven biodeterioration of these relics^2–4^. Many monuments are subjected to the impacts of artificial lights in combination with elevated concentrations of carbon dioxide (CO_2_) exhaled by tourists^5^. Under such circumstances, outbreaks of the phototrophic microbiome and critical biodeterioration of monuments have been reported, for example, in some of the most important heritage monuments, such as Lascaux Cave^6^ and Altamira Cave^5^. Hence, it is paramount to identify innovative and practical approaches to ensure the conservation of cultural monuments while allowing tourism in such environments.

Artificial white light (the full visible spectrum of 400–700 nm) is often used to illuminate historical monuments in indoor environments worldwide. This type of light is known to influence the capacity of phototrophic microbiomes to colonize and thrive on cultural monuments and eventually cause biodeterioration^2,3^. However, cell pigments can control the capacity of phototrophic microbes to absorb photons within a specific light spectrum. For example, two *Synechococcus*-type picocyanobacteria both have strong absorbance at ~430 nm (i.e., blue light). In addition, each of them has an orange‒red component (620–630 nm) and a green‒yellow component (560–570 nm) of the spectrum, by which red or green light *vs*. blue or white light had different influences^7^. Subsequently, light wavelengths can influence the ecophysiological features of the phototrophic microbiome as well as their driving ecological functions^8–10^. In this respect, we hypothesize that types of artificial light with different wavelengths can largely impact the status of conservation of historical monuments associated with the phototrophic microbiome. Identifying the light wavelengths by which phototrophic microbiomes cause the least biodeterioration could help mitigate and/or prevent their negative impacts on long-term conservation for future generations. However, despite its importance, in situ experimental investigations aiming to provide innovative solutions for the conservation of visitable monuments are rarely conducted, and the influence of different light wavelengths in shaping the phototrophic microbiome and its damage to historical monuments remains virtually unknown^11–13^.

**Fig. 1:**
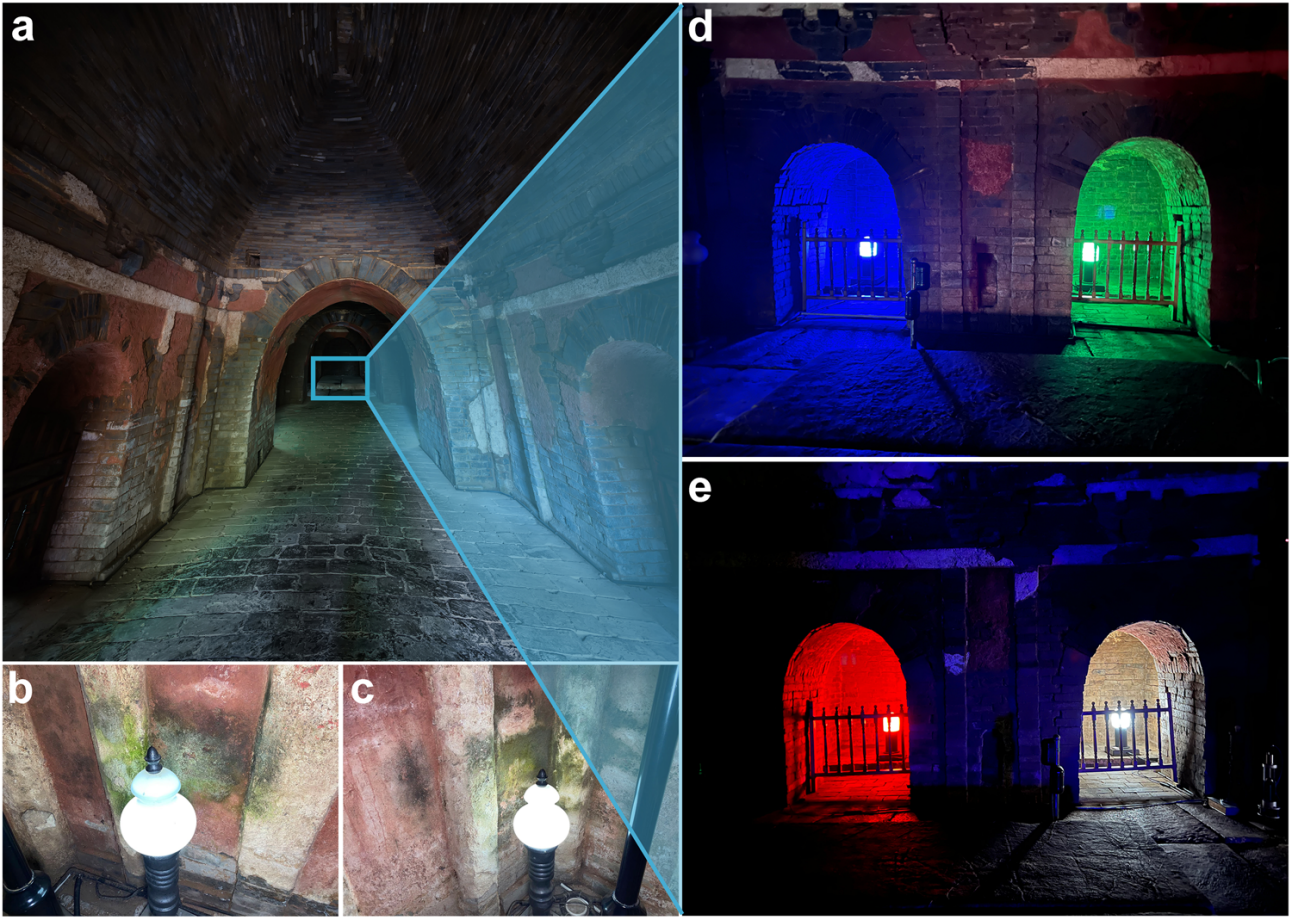
Overview of the Shunling imperial mausoleum with an over 1060-year history that has been open to the public for over 60 years. The appearance of the Shunling Mausoleum (**a**). The green phototrophic microbiome is widespread on the tomb walls exposed to filament lamps with white light (400–700 nm) (**b** and **c** in the rear chambers of the Two Mausoleums of the Southern Tang Dynasty). A 2-year in situ experiment on three typical artificial light wavelengths (i.e., blue (~455 nm), green (~530 nm) and red (~640 nm) lights) is conducted to replace the white light lamps in the rear chambers in the Shunling Mausoleum (**d**, **e**). More detailed information is presented in Supplementary Fig. 1b.

This study provides, to our knowledge, the long-term in situ manipulative experiment that evaluated the phototrophic microbiome on a millennial heritage monument in an indoor environment. Here, we combined next-generation sequencing strategies (amplicon and shotgun metagenomics) and microbial metabolic assays (enzyme activities and metabonomics) with an innovative in situ experiment in the Shunling Mausoleum (China) to shed some light on this important issue. In particular, we conducted a 2-year in situ manipulative experiment to assess the influence of three representative artificial light wavelengths (i.e., blue, green and red lights), in addition to the commonly used artificial white light, on the ecophysiological features of phototrophic microbiomes on walls in this heritage monument. The Qinling Mausoleum of Emperor Bian Li (built in 943 A.D.) and the Shunling Mausoleum of Emperor Jing Li (961 A.D.) (Fig. 1a) constitute the Two Mausoleums of the Southern Tang Dynasty (937 A.D. to 975 A.D.), which are the two largest imperial mausoleums in the Five Dynasties and Ten Kingdoms period (902 A.D. to 979 A.D.) of China with more than 1060 years of history (Supplementary Fig. 1a). The Two Mausoleums of the Southern Tang Dynasty are the only currently existing mausoleums retaining murals in southern China. Their relics authentically record various aspects of daily life (e.g., sacrifice, agriculture, entertainment, and costumes) during that period, and they provide extremely precious historical materials for research on Chinese history and culture. While being open to tourists for over 60 years, artificial white light illuminating systems have been used to facilitate tourism. Green phototrophic microbiomes are widespread on tomb walls exposed to filament lamps and cause both aesthetic and structural deterioration of these monuments (Fig. 1b, c). In September 2019, white light lamps in the three rear chambers of the Shunling Mausoleum were replaced by blue, green or red LED lamps (Fig. 1d, e). Our study aims to provide guidance on what light wavelength is more appropriate for the long-term conservation of cultural monuments in indoor environments worldwide.

## Results and discussion

### The phototrophic bacteriome of a millennial mausoleum

**Fig. 2:**
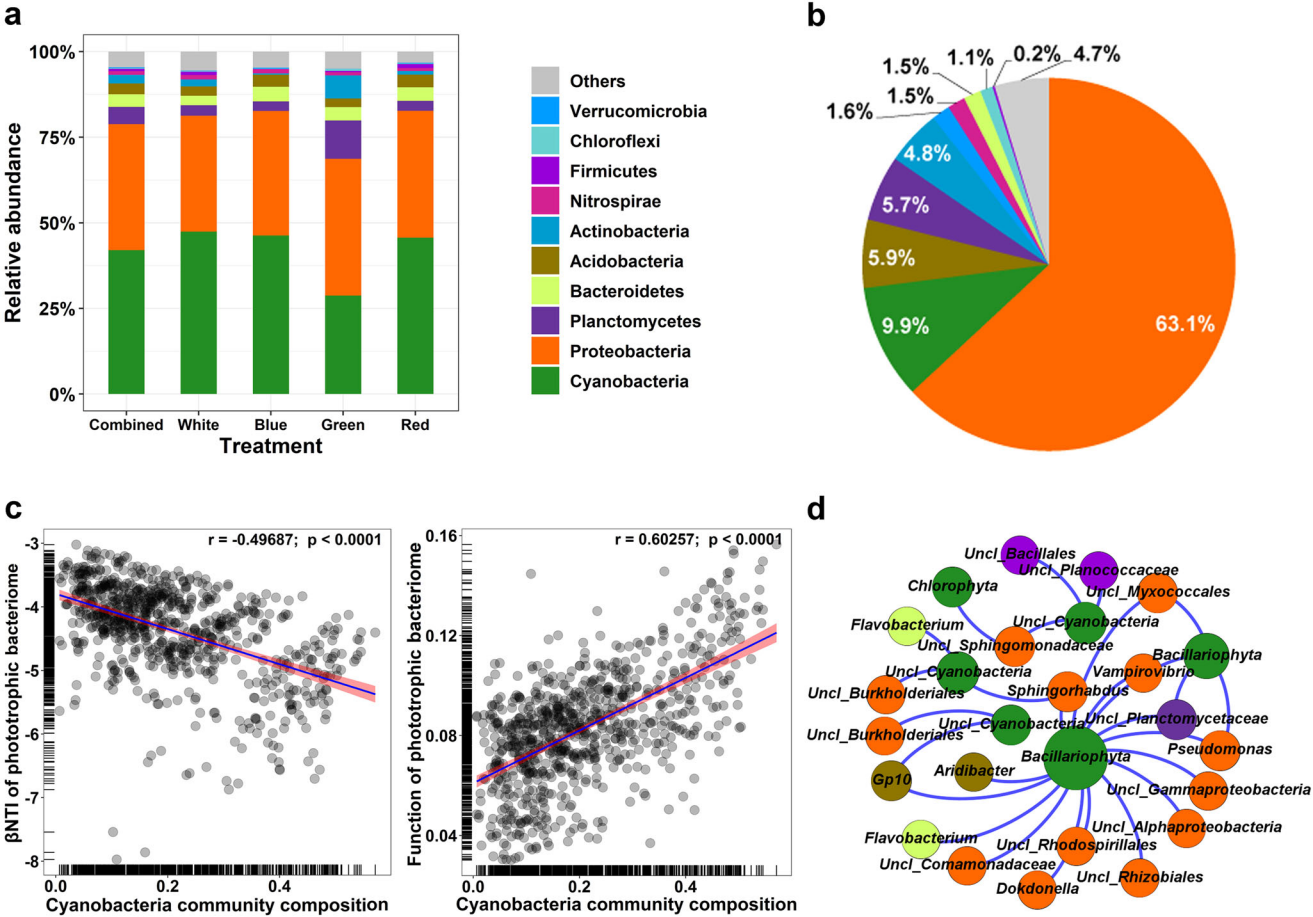
The phototrophic bacteriome of the Shunling imperial mausoleum. Compositional (**a**) and functional (**b**) features of the phototrophic bacteriome on walls irradiated by artificial light wavelengths in the Shunling Mausoleum. The detailed functional gene profiles of (**b**) are listed in Supplementary Table 1. Distance matrix regressions between the Cyanobacteria community composition and the ecological assembly process as well as the functional profile of the phototrophic bacteriome (**c**). The community assembly process was estimated by the *β*NTI; the Cyanobacteria community composition was estimated based on the Bray‒Curtis distance and the ecological functional profile of the phototrophic bacteriome was estimated based on the Bray‒Curtis distance of microbial Kyoto Encyclopedia of Genes and Genomes (KEGG) Orthology annotations by metagenomic sequencing. Ecological co-occurrence network of the significant positive interactions between Cyanobacteria and bacterial taxa mainly affiliated with Proteobacteria, Firmicutes, Bacteroidetes, Acidobacteria and Planctomycetes in the phototrophic bacteriome (**d**). Each node represents a microbial phylotype with an OTU clustered at a 97% identity threshold; the node size is proportional to the number of connections (degree); “Uncl” of node text denotes “unclassified”. The same colors in panels (**a**), (**b**), and (**d**) represent the same bacterial taxa.

After 2 years of continuous exposure, light wavelengths led to an aesthetic alteration of the tomb walls. For example, blue and green light led to the colonization of brown phototrophic biofilms on the walls, while red and white light led to green phototrophic biofilm colonization (Supplementary Fig. 1b). Metagenomic sequencing indicated that the phototrophic bacteriome on the Shunling tomb walls was dominated by bacterial taxa (accounting for 98.8% of reads) (Supplementary Fig. 2a). At a higher resolution, Cyanobacteria was the most dominant bacterial taxon (42.0% for amplicon sequencing and 16.5% for metagenomic sequencing), followed by Proteobacteria (36.8% and 54.4%), Planctomycetes (5.0% and 6.3%), Bacteroidetes (3.7% and 2.5%), Acidobacteria (3.1% and 5.9%), and Actinobacteria (2.5% and 6.7%) (Fig. 2a and Supplementary Fig. 2b). Cyanobacteria dominated the phototrophic taxa in this study, and other phototrophic taxa affiliated with Proteobacteria (0.74% for metagenomic sequencing), Chlorobi (<0.01%), and Firmicutes (<0.01%) phyla accounted for only a very small proportion. Similar results were found when we explored the proportions of functional genes associated with different phyla. Proteobacteria (63.1%), Cyanobacteria (9.9%), Acidobacteria (5.9%), Planctomycetes (5.7%), Actinobacteria (4.8%) and Bacteroidetes (1.1%) together accounted for ~95% of functional genes involved in carbon fixation, denitrification, and dissimilatory sulfate reduction related to phototrophic bacteriome biodeterioration^4^ (Fig. 2b; the detailed information in Supplementary Table 1).

Our results highlight the ecological importance of Cyanobacteria in the phototrophic bacteriome in this millennial monument. As photoautotrophs, Cyanobacteria are pioneering organisms that can initially lodge in illuminated oligotrophic environments^14,15^. Previous studies have shown that Cyanobacteria have great ecological competitiveness in indoor monuments with illumination^16,17^, such as the Monumental Cemetery of Milano^18^ and Tomba Bartoccini^19^ in Italy, the tombs of the Necropolis of Carmona in Spain^20^, and the Carlsbad Cavern in New Mexico^21^. In our study, Cyanobacteria showed, among all bacterial taxa, the greatest correlations with the assembly process (characterized by *β*NTI, which governs the community structure) (*r* = −0.497, *P* < 0.0001) and functional composition (*r* = 0.603, *P* < 0.0001) of the phototrophic bacteriome (Fig. 2c and Supplementary Fig. 3), suggesting that Cyanobacteria are the critical taxa that govern the microbiome of this monument. As primary producers, Cyanobacteria facilitate the colonization and growth of heterotrophic organisms by providing small molecular weight carboxylic acids derived from atmospheric carbon fixation (Supplementary Fig. 4 revealed by metabonomics)^22,23^. In our study, we found multiple positive correlations between Cyanobacteria and *Flavobacterium*, *Pseudomonas*, Burkholderiales, Comamonadaceae, Myxococcales, Planctomycetaceae, Rhizobiales and Sphingomonadaceae (Fig. 2d). All these heterotrophic taxa have been previously reported to be involved in the biodeterioration of historical relics worldwide^24–31^. These results suggest that Cyanobacteria may not only dominate the phototrophic bacteriome of this monument but may also provide a habitat for other microbial taxa heavily involved in biodeterioration. Any alteration in the structure and composition of the Cyanobacteria community may directly impact the heterotrophic ones, which ultimately influence the underlying historical relics.

### Light wavelength regulates the ecophysiological features of the phototrophic bacteriome and may deactivate their biodeterioration potential

**Fig. 3:**
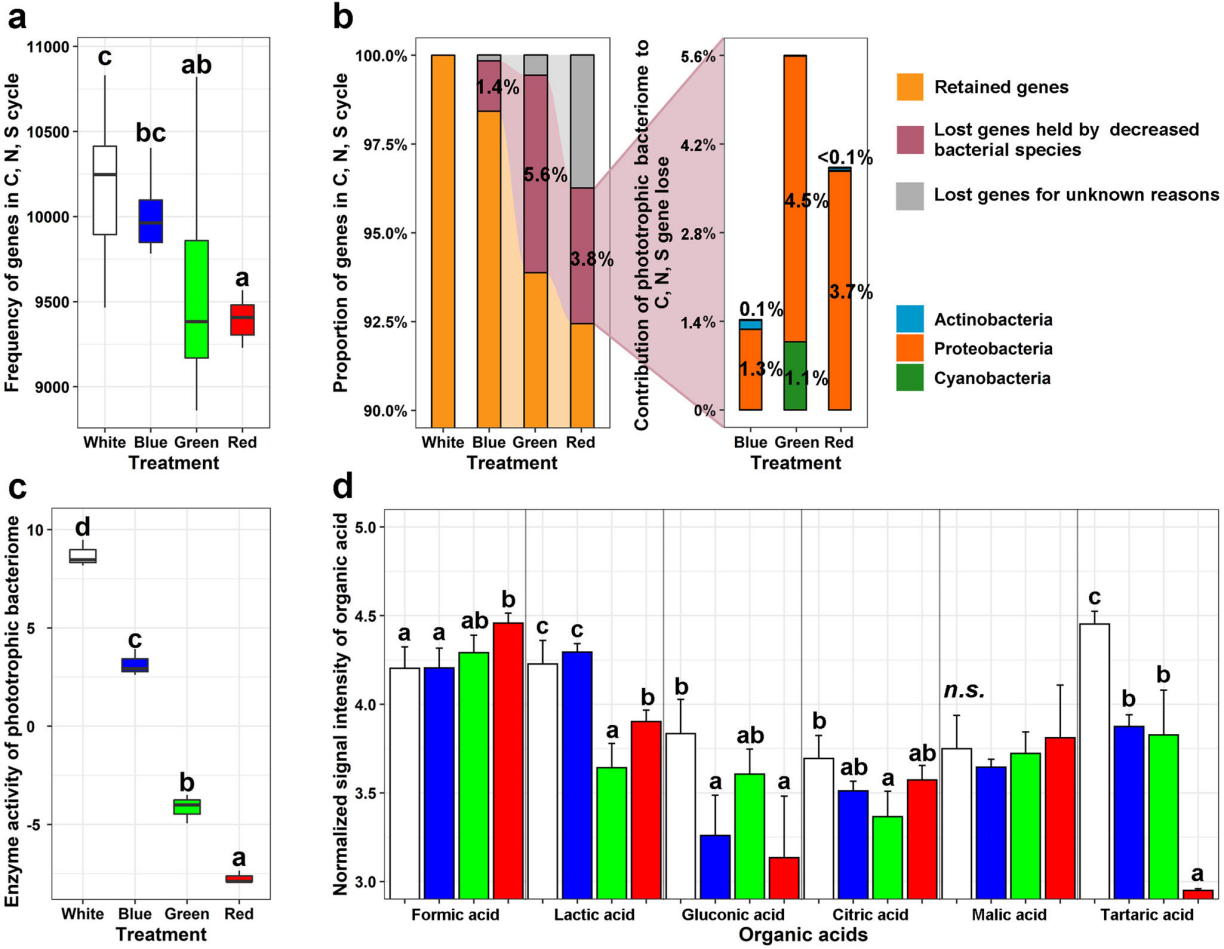
The ecophysiological features of phototrophic bacteriome under different artificial lights. Changes in the functional profiles of the phototrophic bacteriome related to biodeterioration in response to blue, green and red light compared to white light (**a**). Proportions of the reduced functional genes related to biodeterioration contributed by the decreased keystone species (or biomarkers) illuminated by blue, green and red lights compared to the white light (*P* < 0.05) (**b**). C, N, and S cycling denote carbon fixation, denitrification, and dissimilatory sulfate reduction, respectively (for more detailed functional gene profiles, please see Supplementary Table 1). Changes in enzyme activity (**c**) and organic acid signal intensity (**d**) of the phototrophic bacteriome under blue, green and red light compared to white light. Enzyme activities were Z score transformed by the eight extracellular enzymes (i.e., *β*-glucosidase, cellobiohydrolase, xylanase, *β*-galactosidase, leucine amino peptidase, *β*-N-acetylglucosaminidase, N-acetyl-*β*-galactosaminidase and sulfatase) activities (for more detailed enzyme profiles, please see Supplementary Table 6). The center line of the boxplot indicates the median of the data, the bounds of the box represent the interquartile range, and the whiskers indicate the range of the data, excluding outliers. Different letters over error bars denote significant differences (*P* < 0.05), and “*n.s*.” denotes *P* > 0.05. The same colors in panels (**a**), (**c**), and (**d**) represent the same artificial light wavelengths.

Our 2-year in situ manipulative experiment further showed that certain light wavelengths may influence the biodeterioration potential of the microbiome of visitable millennial monuments by regulating their structural and functional profiles. Generally, after 2 years of continuous exposure to monochromatic light wavelengths (i.e., green and red lights), our analyses revealed that the structural and functional profiles of the phototrophic bacteriome on the walls of this monument shifted compared to white light (*P* < 0.05) (Fig. 3a; Supplementary Figs. 5–7; Supplementary Tables 2 and 3). In this experiment, except for illumination, other environmental variables, such as temperature and humidity, were almost identical for the four adjacent rear chambers when compared to the local environment (the maximum distance between the chambers was <10 m, Supplementary Fig. 1b) (Supplementary Fig. 8). In this respect, the changes in the ecophysiological features of the phototrophic bacteriome largely originate from the specificity of the absorbance spectrum of phototrophic organisms (e.g., Cyanobacteria) (e.g., green phototrophic species do not absorb green light)^7^. Specifically, in this study, the abundance of Cyanobacteria photosynthetic pigments was observed to decrease in response to green light compared to white light (*P* < 0.05) (Supplementary Fig. 9). Consequently, for the Cyanobacteria populations (Fig. 2a) and their functional genes associated with carbon (Supplementary Fig. 10a; detailed information in Supplementary Table 1) and nitrogen (Supplementary Fig. 10b; detailed information in Supplementary Table 4) fixation as well as related to biodeterioration (Supplementary Fig. 10c; detailed information in Supplementary Table 1), their abundances all decreased under such environmental conditions (*P* < 0.05). Cyanobacteria can support the growth of heterotrophic species that biodeteriorate monuments via carboxylic acids (Fig. 2d and Supplementary Fig. 4). Thus, the suppression of Cyanobacteria by green and red light may diminish their capacity to produce carboxylic acids (*P* < 0.05) (Supplementary Fig. 4). In this respect, the abundances of heterotrophic species (including *Flavobacterium*, *Pseudomonas*, Burkholderiales, Comamonadaceae, Myxococcales, Planctomycetaceae, Rhizobiales and Sphingomonadaceae) and their held functional gene abundances related to biodeterioration were all found to have decreased (*P* < 0.05) (Fig. 3b and Supplementary Table 5).

Such changes in the ecophysiological features of the phototrophic bacteriome can help deactivate biodeterioration potential. Functional analyses provided evidence that for the whole phototrophic bacteriome, the abundances of both the total functional genes (Supplementary Fig. 7) and the specific functional genes involved in carbon fixation, denitrification, and dissimilatory sulfate reduction related to biodeterioration (Fig. 3a) decreased in response to green and red light compared to white light (*P* < 0.05)^3,4,12^. Meanwhile, multiple extracellular enzymatic activities involved in biodeterioration processes were reduced under green and red light compared to white light (Fig. 3c). In addition, organic acids excreted by the microbiome have been proven to be destructive to monuments^4,32^, such as lactic, gluconic, citric and tartaric acids^33–36^. Metabonomics also indicated that these acids excreted by the phototrophic bacteriome were all decreased by green and red light compared to white light (*P* < 0.05) (Fig. 3d). The significant positive correlation (*r* = 0.856, *P* < 0.001) between the proportion of genes associated with C, N, and S cycling and the excreted organic acids (Supplementary Fig. 11) implies that light wavelength can regulate the abundance of functional genes of the phototrophic bacteriome, which may further impact metabolite (for example, organic acids related to potential biodeterioration) production.

**Fig. 4:**
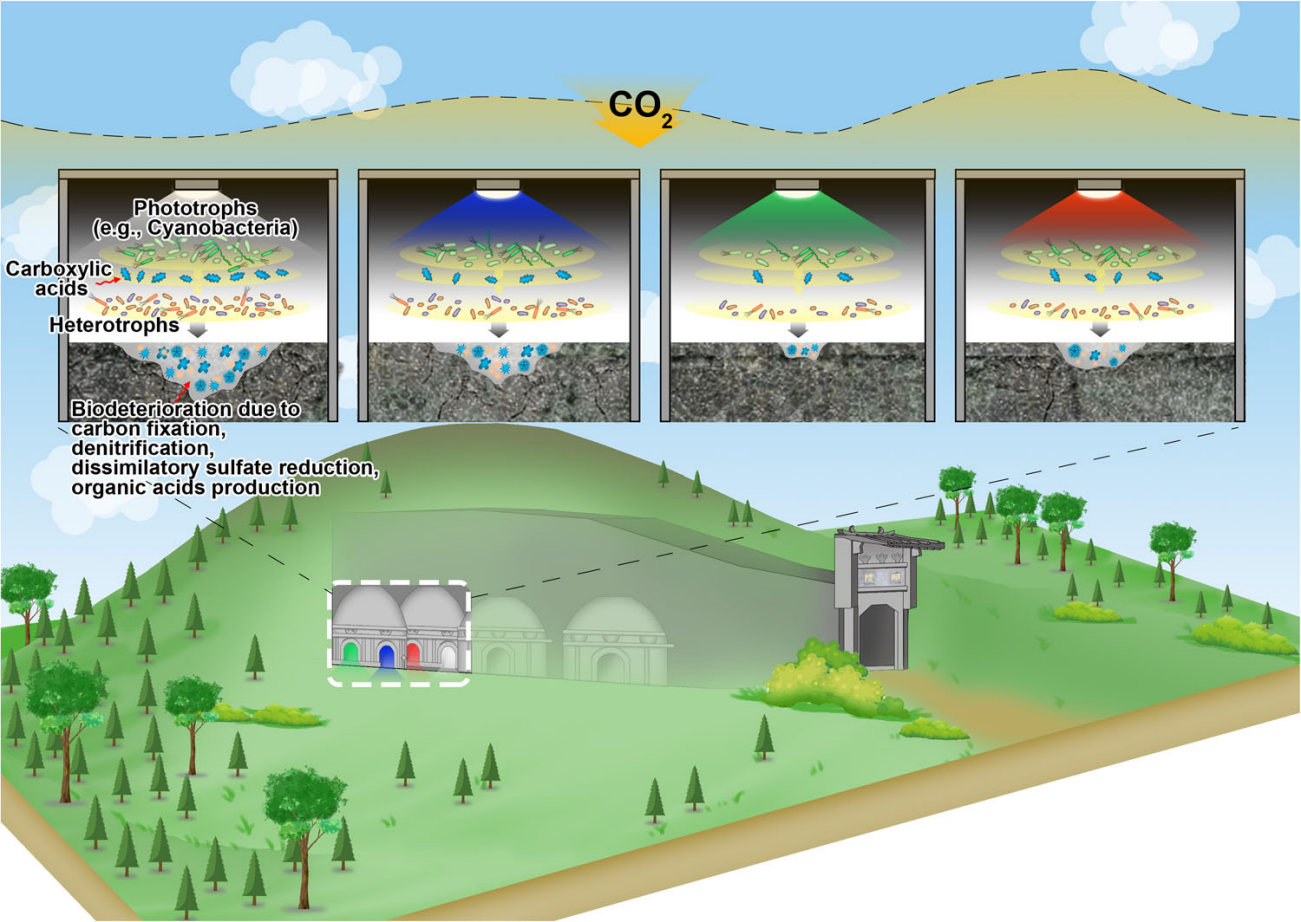
Mechanistic map of artificial lights impacting the phototrophic microbiome on monuments in indoor environments. In brief, Cyanobacteria govern community composition and ecological functions (involved in carbon fixation, denitrification, and dissimilatory sulfate reduction, as well as organic acid production) related to biodeterioration of the phototrophic microbiome on monuments under artificial lights. Due to the weak absorbance at ~530 nm of photosynthetic pigments, green light can decrease Cyanobacteria abundances and their functional traits, further influencing the compositional and functional profiles of its oriented heterotrophic microorganisms related to biodeterioration and finally weakening the biodeterioration potential of the whole phototrophic microbiome on monuments, all of which help for the long-term conservation of historical monuments worldwide.

Interestingly, our results suggested that blue light does not have the capacity to neutralize the biodeterioration potential of cultural relic microbiomes. As a consequence of the strong absorbance at ~430 nm by the widespread Cyanobacteria species^7^, blue light had the least impact on the phototrophic bacteriome, regardless of Cyanobacteria (Fig. 2a), heterotrophic species (Figs. 2d and 3b and Supplementary Table 5), functional genes (Fig. 3a), enzymatic activities (Fig. 3c) and excreted organic acids (Fig. 3d). Taken together, all of the above results support our hypothesis that changes in the ecophysiological features of the phototrophic bacteriome in response to certain light wavelengths can influence the biodeterioration potential of indoor monuments with illumination. Monochromatic light, especially green light, can be the most promising artificial light to help the long-term conservation of historical monuments suffering from Cyanobacteria-oriented phototrophic microbiomes in indoor environments (Fig. 4).

Some recent studies have revealed that biofilm communities (e.g., phototrophic microbiome) on historical monuments can assume neutral or even protective roles, for example, by shielding the surfaces from the direct impacts of rain droplets, wind abrasion, and solar radiation^17^. However, the indoor environments evaluated in this study suggest that light-induced biofilm communities play only slightly positive roles. Moreover, it is also known that observable biodeterioration usually takes a long time (for example, decades or hundreds of years)^37,38^. Unlike many previous related studies based on already existing biodeterioration, even though no quantitative differences (in situ detected by X-ray fluorescence spectrometry) were observed in biodeterioration in this study, the amplicon and shotgun metagenomic sequencing data combined with the enzymatic activities and metabolomics of this study provided potentially biologically integrative methods and ideas for early diagnosis^39^ (before the emergence of detectable biodeterioration). In regards to the impacts of artificial light wavelengths on the biodeterioration potential of cultural relic microbiomes, early diagnosis is extremely important for the prevention and protection of cultural relics^40,41^.

In summary, we conducted a 2-year in situ manipulative experiment in a millennial mausoleum to investigate the impacts of three typical light wavelengths (i.e., blue, green and red lights) on the community structure, ecological function and biodeterioration potential of the phototrophic microbiome compared to white light with a full visible spectrum. Specifically, due to the absorbance spectrum specificity of photosynthetic pigments, certain monochromatic lights, especially green light in this study, greatly influenced the ecophysiological features of the Cyanobacteria-oriented phototrophic bacteriome, weakened their metabolic abilities involving element cycling, and finally deactivated their biodeterioration potential on monuments. This information helps build a blueprint essential for developing approaches and strategies aimed at supporting tourism benefits while preserving indoor cultural monuments exposed to artificial lights worldwide for future generations.

## Methods

### Description of the Shunling imperial mausoleum

The Shunling imperial mausoleum is located in Nanjing City, Jiangsu Province, China (31°89’N, 118°74’E), which has a mean annual average temperature of 15.4 °C and a mean annual precipitation of ∼1106.5 mm. The tomb was built in 961 A.D. with a multichamber structure composed of brick. It is approximately 9 m below ground level, 21.90 m in length, 10.12 m in width, and 5.42 m in height and is composed of one corridor with four ear chambers along each side. It was excavated by the Nanjing Museum in 1950, with more than 600 precious cultural relics unearthed, and opened to public visits in 1985.

### The in situ illumination experimental design and sample collection

Four ear chambers in the back part of the tomb were selected for the artificial light irradiation experiment (Supplementary Fig. 1b). Before this study, these ear chambers were illuminated with white LED light containing full wavelengths (400–700 nm). Three of these four ear chambers were randomly selected, and the white LED light in each chamber was replaced by light of different wavelengths. The LEDs were blue peaking at ~455 nm, green at ~530 nm, and red at ~640 nm. The lighting systems were set up from September 2019 to August 2021 with a 12 h:12 h light:dark photoperiod per day. The temperature and relative humidity values of each ear chamber were recorded every 30 min from September 1, 2020, to August 31, 2021, by using a HOBO MX2301A (Onset Computer Corporation, Bourne, USA). Samples with 2 years of exposure to different wavelengths of artificial lights were collected on August 20, 2021 (10 for each, and a total of 40 samples were collected). Specifically, a sterile scalpel was carefully used to scrape the colonies to avoid damaging the cultural relic. After collection, all the samples were immediately delivered to the laboratory on ice and frozen at −80 °C until further use.

### DNA extraction

Genomic DNA was extracted from each sample. In brief, a 0.5 g sample was extracted using the FastDNA® SPIN Kit for Soil (MP Biomedicals, Santa Ana, CA), including a negative control. The extracted DNA was eluted in 50 μL of TE buffer. The DNA extract was checked on a 1% agarose gel, and DNA concentration and purity were determined using a NanoDrop 2000 UV‒vis spectrophotometer (Thermo Scientific, Wilmington, USA) and stored at −20 °C until further use.

### Shotgun metagenomic and amplicon sequencing

DNA was fragmented to an average size of approximately 400 bp using Covaris M220 (Gene Company Limited, China) for paired-end library construction. A paired-end library was constructed using NEXTflexTM Rapid DNA-Seq (Bioo Scientific, Austin, TX, USA). Adapters containing the full complement of sequencing primer hybridization sites were ligated to the blunt end of fragments. Paired-end sequencing was performed on an Illumina NovaSeq 6000 (Illumina Inc., San Diego, CA, USA) at Majorbio Bio-Pharm Technology Co., Ltd. (Shanghai, China) using NovaSeq Reagent Kits according to the manufacturer’s instructions (www.illumina.com).

For amplicon sequencing, the primer set 519F/907R was used to amplify approximately 400 bp of bacterial 16S *rRNA* gene V4-V5 fragments^42^. Briefly, the unique 5-bp barcoded oligonucleotide sequence was fused to the forward primer to distinguish different samples. The PCR mixtures contained 4 μL of 5 × TransStart FastPfu buffer, 2 μL of 2.5 mM dNTPs, 0.8 μL of forward primer (5 μM), 0.8 μL of reverse primer (5 μM), 0.4 μL of TransStart FastPfu DNA Polymerase, 10 ng of template DNA, and ddH_2_O up to 20 μL. The negative control was run with water as the template instead of sample DNA. Thirty-five cycles (95 °C for 45 s, 56 °C for 45 s, and 72 °C for 60 s) were performed with a final extension at 72 °C for 7 min. PCRs were performed in triplicate. The PCR product was extracted from a 2% agarose gel, purified using the AxyPrep DNA Gel Extraction Kit (Axygen Biosciences, Union City, CA, USA) according to the manufacturer’s instructions and quantified using a Quantus™ Fluorometer (Promega, USA). Purified amplicons were pooled in equimolar amounts and paired-end sequenced on an Illumina MiSeq PE300 platform (Illumina, San Diego, USA) according to the standard protocols by Majorbio Bio-Pharm Technology Co. Ltd. (Shanghai, China).

### Sequencing data processing

For shotgun metagenomic data, the raw reads from metagenome sequencing were used to generate clean reads by removing adapter sequences, trimming and removing low-quality reads (reads with N bases, a minimum length threshold of 50 bp, and a minimum quality threshold of 20) using fastp^43^ on the free online Majorbio Cloud Platform (cloud.majorbio.com). These high-quality reads were then assembled into contigs using MEGAHIT^44^ (parameters: kmer_min = 47, kmer_max = 97, step = 10), which makes use of succinct de Bruijn graphs. Contigs with lengths over 300 bp were selected as the final assembly result. Open reading frames (ORFs) in contigs were identified using MetaGene^45^. The predicted ORFs with a length over 100 bp were retrieved and translated into amino acid sequences using the NCBI translation table (http://www.ncbi.nlm.nih.gov/Taxonomy/taxonomyhome.html/index.cgi?chapter=tgencodes#SG1). A nonredundant gene catalog was constructed using CD-HIT^46^ with 90% sequence identity and 90% coverage. After quality control, reads were mapped to the nonredundant gene catalog with 95% identity using SOAPaligner^47^, and gene abundance in each sample was evaluated. Representative sequences of the nonredundant gene catalog were annotated based on the NCBI NR database using blastp as implemented in DIAMOND v0.9.19 with an e-value cutoff of 1e^−5^ using Diamond^48^ for taxonomic annotations. Clusters of orthologous groups of proteins (COG) annotation for the representative sequences was performed using Diamond^48^ against the eggNOG database (version 4.5.1) with an e-value cutoff of 1e^−5^. KEGG annotation was conducted using Diamond^48^ against the Kyoto Encyclopedia of Genes and Genomes database (http://www.genome.jp/keeg/, version 94.2) with an e-value cutoff of 1e^−^^5^. All the shotgun metagenomic sequencing data were normalized with the reads assigned per kilobase of target per million mapped reads (RPKM) method^49^.

For amplicon sequence data, the raw 16S *rRNA* gene sequencing reads were processed using the Quantitative Insights Into Microbial Ecology (QIIME, USA) pipeline^50^. Sequences with a quality score below 25 and a length less than 300 bp were trimmed and the rest were then assigned to samples based on unique 5-bp barcodes. Sequences were denoised, and the quality reads were then binned into operational taxonomic units (OTUs) using a 97% identity threshold. The most abundant sequence from each OTU was selected as a representative sequence. Taxonomy was assigned to bacterial OTUs with reference to a subset of the SILVA 119 database^51^. A phylogenetic tree was constructed using FastTree to support phylogenetic diversity calculations. In total, we obtained 2,762,970 quality sequences of the bacterial 16S *rRNA* gene and between 41,737 and 80,226 sequences per sample, with a median value of 55,608 sequences per sample. All samples were randomly rarified to 41,737 sequences for downstream analyses because an even depth of sampling is required for alpha (*α*) and beta (*β*) diversity comparisons^52^.

### Molecular ecological network analysis

To reveal the co-occurrence patterns between Cyanobacteria and others on the tomb walls, phylogenetic molecular ecological networks (pMENs) among different artificial lights were constructed using the random matrix theory (RMT)-based network approach^53^. The pMEN construction and analysis were performed with the online pipeline of Deng et al.^54^ with default parameters. The constructed network was then visualized using Gephi software^55^.

### Microbial community assembly process analyses

The phylogenetic turnover (phylogenetic *β*-diversity) between samples was quantified using the between-community *β*-mean nearest taxon distance (*β*MNTD)^56^ and the *β*-nearest taxon index (*β*NTI)^57^ metrics. The *β*NTI measures the difference between the observed *β*MNTD and the null *β*MNTD distribution. The null distribution was generated by 1000 randomizations in which a null value of the *β*MNTD was calculated after shuffling OTU labels randomly across the tips of the phylogeny of all taxa investigated. *β*NTI values below −2 or above +2 indicate phylogenetic turnover that is less than or greater than expected under stochastic assembly, respectively, thereby indicating a primary role of deterministic assembly processes (homogeneous and variable selections, respectively)^57^.

### Enzyme activity measurement

Eight extracellular enzymes involved in carbon (that is, *β*-glucosidase, cellobiohydrolase, xylanase, and *β*-galactosidase), nitrogen (leucine amino peptidase, *β*-N-acetylglucosaminidase, and N-acetyl-*β*-galactosaminidase), and sulfur (sulfatase) cycling that are potentially involved in biodeterioration^4^ were measured as representatives of tomb microbiome function by using a microplate fluorimetric assay according to the description by Marx et al.^58^ with some modifications. Four of the ten samples of each treatment were randomly selected for enzyme activity measurement. In brief, a 1 g sample was weighed into a 50 mL jar with a lid. Then, 10 mL of sterile deionized water was added and vortexed for 60 s. After vortexing, 100 μL of suspension was added to each well of a microplate (Biolog Inc., Hayward, CA). Then, 50 μL of enzymatic reaction buffer and 50 μL of substrate were added to each well and mixed. Then, the microplates were incubated at 37 °C for 30 min. After incubation, the fluorescence intensity was measured at 460 nm with a computerized microplate fluorimeter (Biolog Inc., Hayward, CA). The detailed design of the buffer and substrate for each enzyme is provided in Supplementary Table 6.

### Metabonomics extraction, UHPLC‒MS/MS analysis, and data preprocessing

Four of the ten samples of each treatment were randomly selected for metabonomics measurement. Fifty milligrams of sample was accurately weighed, and the metabolites were extracted using a 400 µL methanol:water (4:1, v/v) solution. The mixture was allowed to settle at −20 °C and treated by a high-throughput tissue crusher (Wonbio-96c, Shanghai Wanbo Biotechnology Co., Ltd.) at 50 Hz for 6 min, followed by vortexing for 30 s and ultrasound at 40 kHz for 30 min at 5 °C. The samples were placed at −20 °C for 30 min to precipitate proteins. After centrifugation at 13,000×*g* at 4 °C for 15 min, the supernatant was carefully transferred to sample vials for LC‒MS/MS analysis. As a part of the system conditioning and quality control process, a pooled quality control (QC) sample was prepared by mixing equal volumes of all samples. The QC samples were disposed and tested in the same manner as the analytic samples. It helped to represent the whole sample set, which was injected at regular intervals to monitor the stability of the analysis.

Chromatographic separation of the metabolites was performed on a Thermo UHPLC system equipped with an ACQUITY BEH C18 column (100 mm × 2.1 mm i.d., 1.7 µm; Waters, Milford, USA). The mobile phases consisted of 0.1% formic acid in water (solvent A) and 0.1% formic acid in acetonitrile: isopropanol (1:1, v/v) (solvent B). The sample injection volume was 2 µL, and the flow rate was set to 0.4 mL/min. The column temperature was maintained at 40 °C. During the period of analysis, all samples were stored at 4 °C.

The mass spectrometric data were collected using a Thermo UHPLC-Q Exactive Mass Spectrometer equipped with an electrospray ionization (ESI) source operating in either positive or negative ion mode. The optimal conditions were set as follows: Aus gas heater temperature, 400 °C; Sheath gas flow rate 40 psi; Aus gas flow rate 30 psi; ion-spray voltage floating (ISVF), −2800 V in negative mode and 3500 V in positive mode; Normalized collision energy, 20-40-60 V rolling for MS/MS. Data acquisition was performed in data-dependent acquisition (DDA) mode. The detection was carried out over a mass range of 70–1050 m/z.

After UPLC-TOF/MS analyses, the raw data were imported into Progenesis QI 2.3 (Nonlinear Dynamics, Waters, USA) for peak detection and alignment. The preprocessing results generated a data matrix that consisted of the retention time (RT), mass-to-charge ratio (m/z) values, and peak intensity. Metabolic features detected in at least 80% of any set of samples were retained. After filtering, minimum metabolite values were imputed for specific samples in which the metabolite levels fell below the lower limit of quantitation, and each metabolic feature was normalized by sum. The internal standard was used for data QC, and metabolic features with a relative standard deviation (RSD) of QC > 30% were discarded. Following normalization procedures and imputation, statistical analysis was performed on log-transformed data to identify significant differences in metabolite levels between comparable groups. Mass spectra of these metabolic features were identified by using accurate mass, MS/MS fragment spectra and isotope ratio differences by searching reliable biochemical databases in the Human Metabolome Database (HMDB) (http://www.hmdb.ca/) and Metlin database (https://metlin.scripps.edu/). For metabolites with MS/MS confirmation, only those with MS/MS fragment scores above 30 were considered confidently identified. Multivariate statistical analyses, including principal component analysis (PCA), orthogonal partial least squares discriminate analysis (OPLS-DA), and pathway analysis, were performed on the Majorbio Cloud Platform (https://cloud.majorbio.com) with the R package “ropls” (Version 1.6.2) from Bioconductor (http://bioconductor.org/packages/release/bioc/html/ropls.html).

### Statistical analysis

The bacterial community and functional composition of the phototrophic bacteriome on tomb walls among different artificial lights were visualized by nonmetric multidimensional scaling analyses (NMDS) based on the Bray‒Curtis distance of the bacterial OTU table annotated by amplicon sequencing and bacterial KEGG orthologies annotated by metagenomic sequencing, respectively. Permutational multivariate analysis of variation (PERMANOVA) tests were conducted to test for statistically significant differences in the community and functional composition of the phototrophic bacteriome among artificial lights using R software (the “vegan” package, Version 2.2-1)^59,60^. For each monochromatic light (i.e., blue, green and red lights), the Wilcoxon rank-sum test was used to find phototrophic species that had significantly decreased abundances when compared to the white light (paired tests when indicated) (after multiple hypothesis testing correction)^61^. The separation of mean values among artificial lights was evaluated with one-way ANOVA followed by post hoc Tukey’s HSD tests using IBM Statistical Product and Service Solutions (SPSS) Statistics for Windows (Version 13). The significance of the linear regression slope was tested by permutation tests^62^. *P* < 0.05 was considered significantly different.

### Reporting summary

Further information on research design is available in the Nature Research Reporting Summary linked to this article.

### Supplementary information

Supplementary information

Reporting Summary

## Data Availability

The amplicon sequences (accession number: DRA014568) and the shotgun metagenomics (DRA014569) were deposited in the DNA Data Bank of Japan (DDBJ) database (https://www.ddbj.nig.ac.jp/index-e.html). All other data generated or analyzed during this study are included in this published article or are available from the corresponding author upon reasonable request.

## Code availability

The code used in this study is available upon request.
