# Supplementary material for: Innovative strategy for the conservation of a millennial mausoleum from biodeterioration through artificial light management
Source: NPJ Biofilms Microbiomes. 2023 Sep 23;9:69. doi: 10.1038/s41522-023-00438-9 (PMC10516906; doi:10.1038/s41522-023-00438-9)
Supplement: Supplementary file 1 — Supplementary information [file 41522_2023_438_MOESM1_ESM.pdf]

Supplementary information

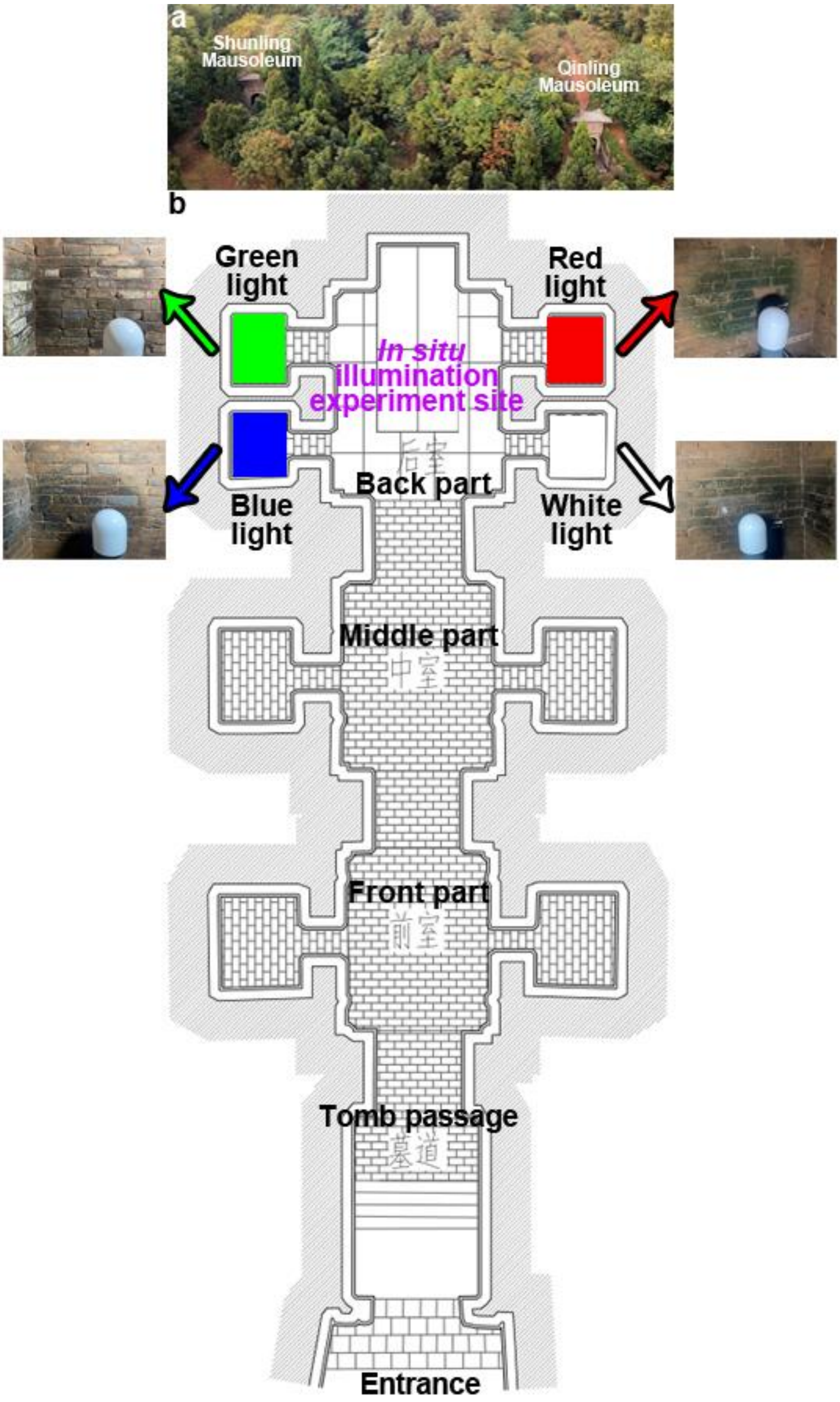

Supplementary Figure 1. Overview of the Two Mausoleums of the Southern Tang Dynasty (Qinling Mausoleum of Emperor Bian Li (built in 943 A.D.) and the Shunling Mausoleum of Emperor Jing Li (built in 961 A.D.)) (a). Details of the Shunling Mausoleum and the in situ illumination experiment design (b).

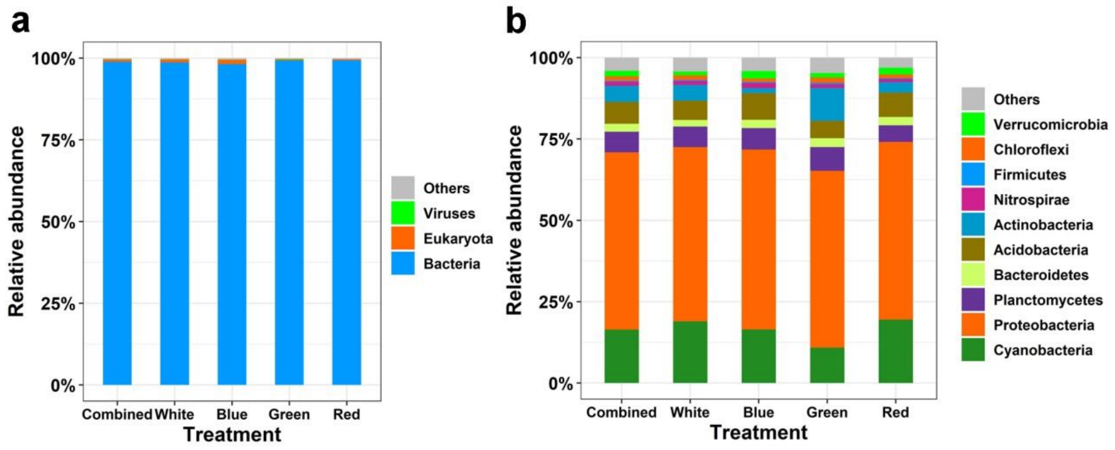

Supplementary Figure 2. Domain (a) and bacterial phyla (b) level community composition on the tomb walls after two years of exposure to blue, green and red lights were revealed by metagenomic sequencing (n = 40).

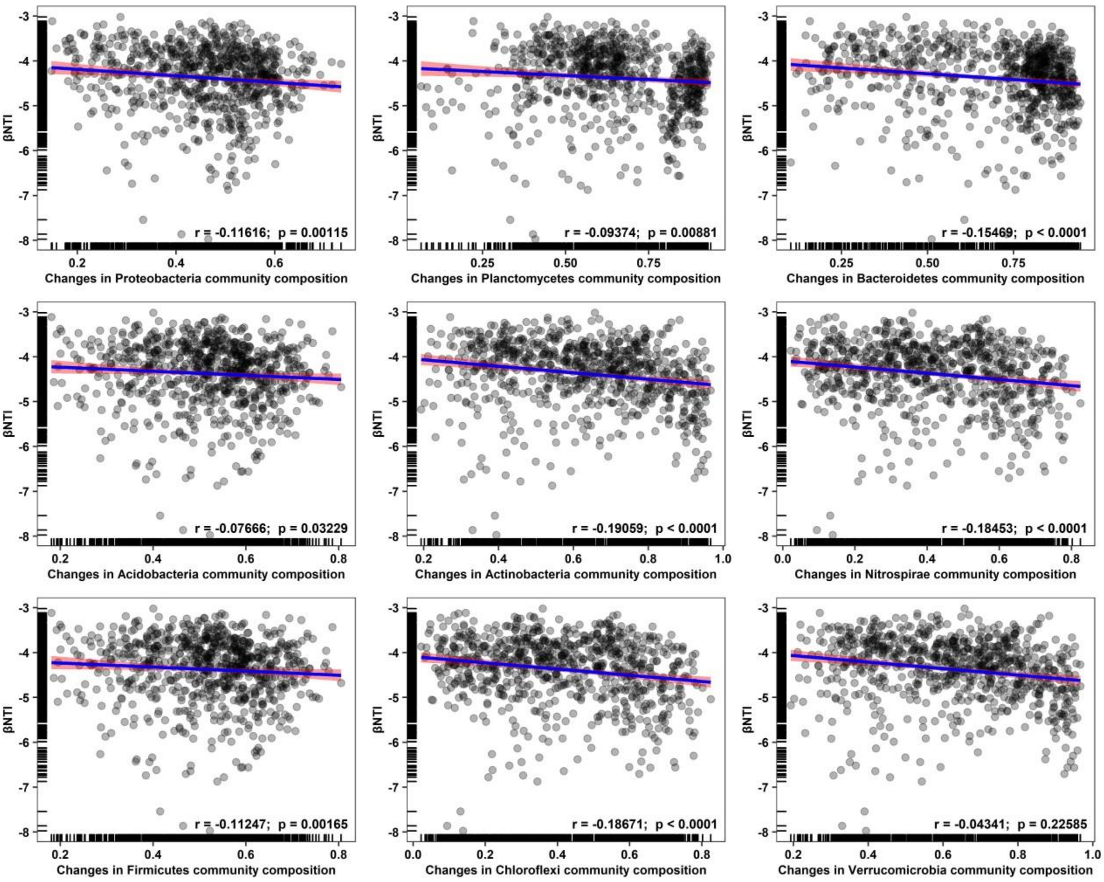

Supplementary Figure 3. Distance matrix regressions between community composition of the other dominant bacterial phyla (e.g., Proteobacteria, Planctomycetes, Bacteroidetes, Acidobacteria, Actinobacteria, Nitrospirae, Firmicutes, Chloroflexi, and Verrucomicrobia) and the ecological assembly process of phototrophic bacteriome. The community assembly process was estimated by the *β*NTI; the community composition of each dominant bacterial phylum was estimated based on the Bray-Curtis distance of each phylum (n = 40).

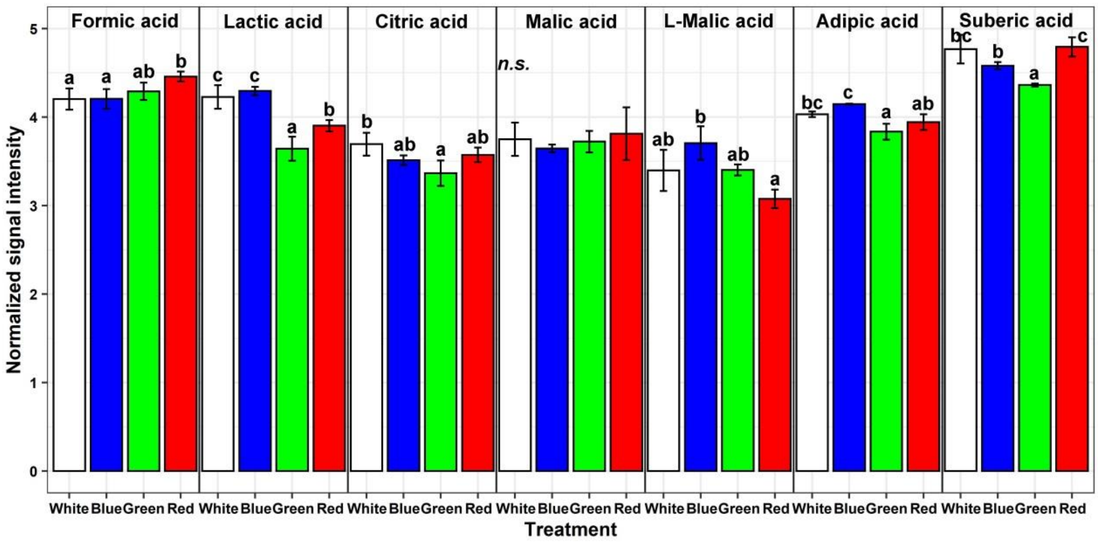

Supplementary Figure 4. Carboxylic acids produced by Cyanobacteria-oriented phototrophic bacteriome under blue, green and red lights. Carboxylic acids signal intensity was revealed by metabonomics (n = 16). Different letters over error bars denote significant differences ($P < 0.05$); "*n.s.*" denotes $P > 0.05$.

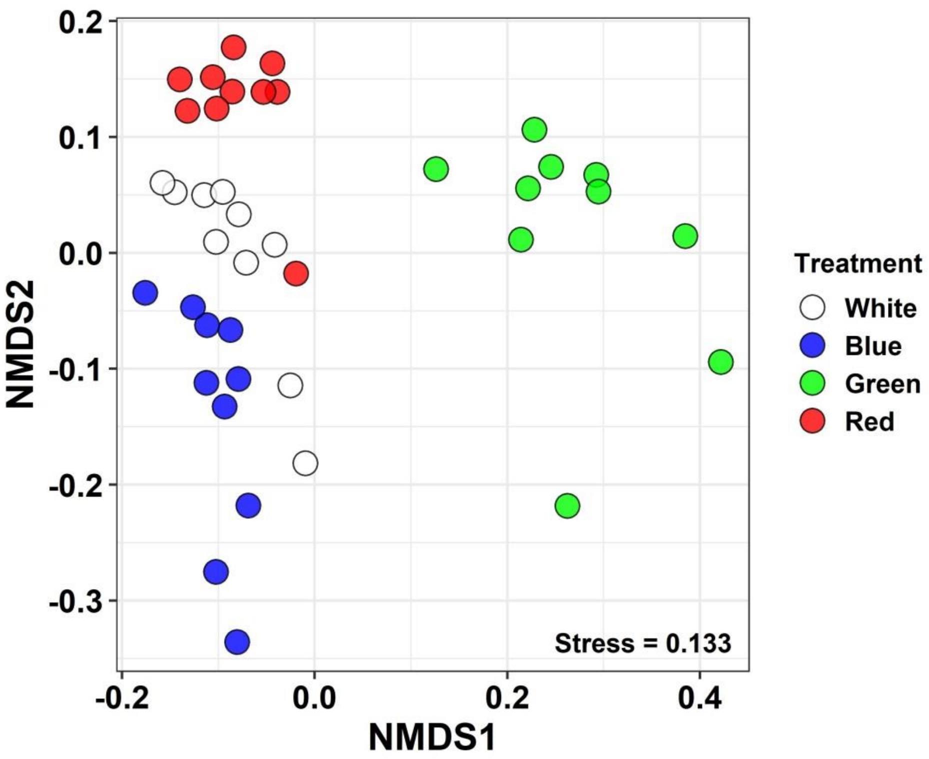

Supplementary Figure 5. Nonmetric multidimensional (NMDS) analysis of Cyanobacteria-oriented phototrophic bacteriome composition on the tomb walls exposed to white, blue, green, and red artificial lights, based on the Bray-Curtis distance of bacterial OTU table annotated by amplicon sequencing (n = 40).

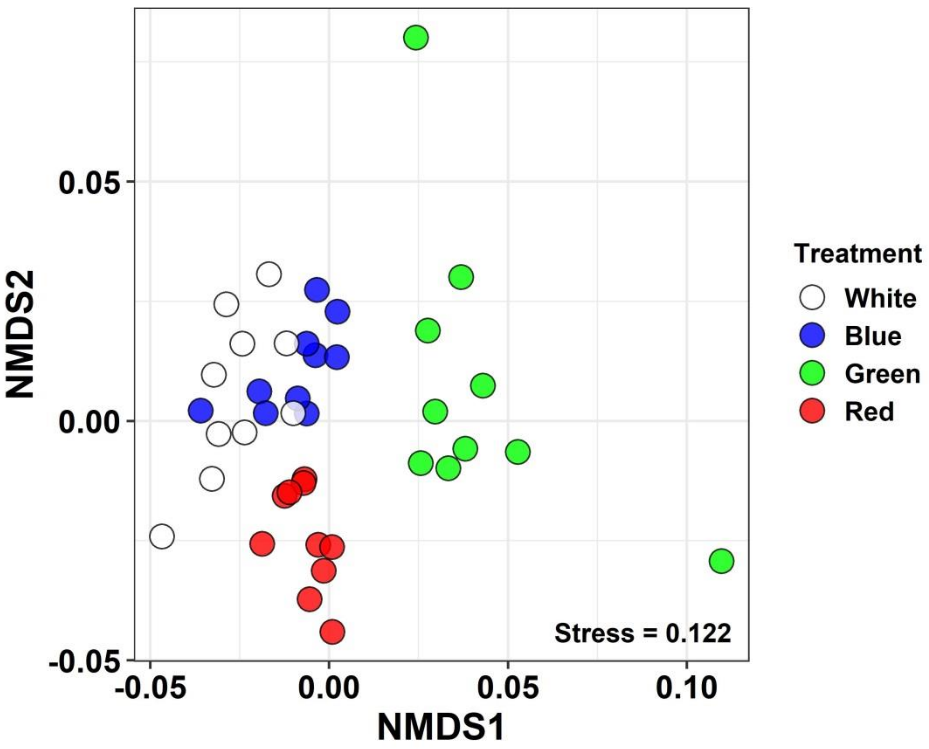

Supplementary Figure 6. Nonmetric multidimensional (NMDS) analysis of functional composition of Cyanobacteria-oriented phototrophic bacteriome on the tomb walls exposed to white, blue, green, and red artificial lights, based on the Bray-Curtis distance of bacterial KEGG Orthologies annotated by metagenomic sequencing (n = 40).

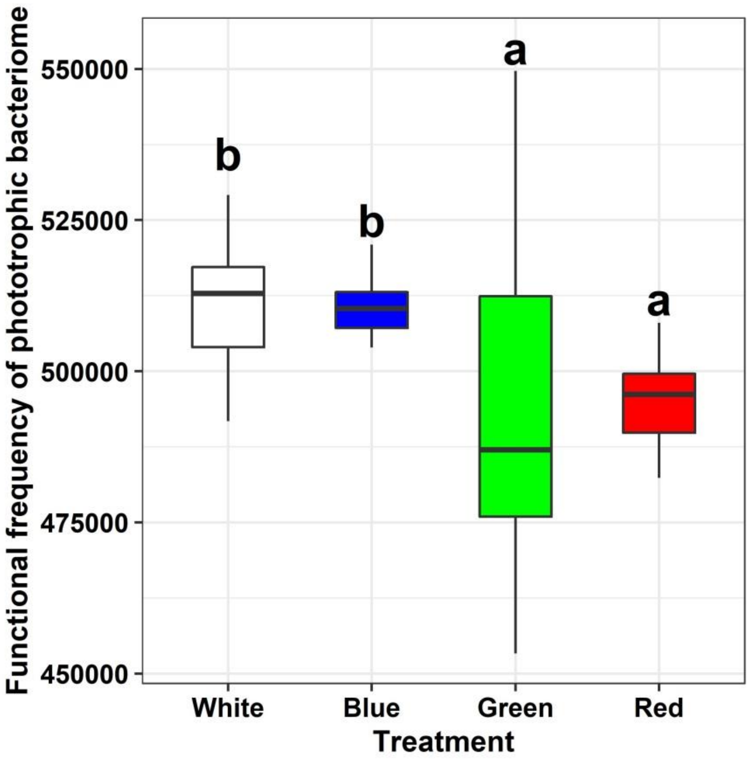

Supplementary Figure 7. Functional frequency of Cyanobacteria-oriented phototrophic bacteriome on the tomb walls under blue, green, and red lights. Functional frequency was the sum of each KEGG Orthology frequency annotated by metagenomic sequencing (n = 40). The centre line indicates the median of the data, the bounds of the box represent the interquartile range, and the whiskers indicate the range of the data, excluding outliers. Different letters over error bars denote significant differences ($P < 0.05$).

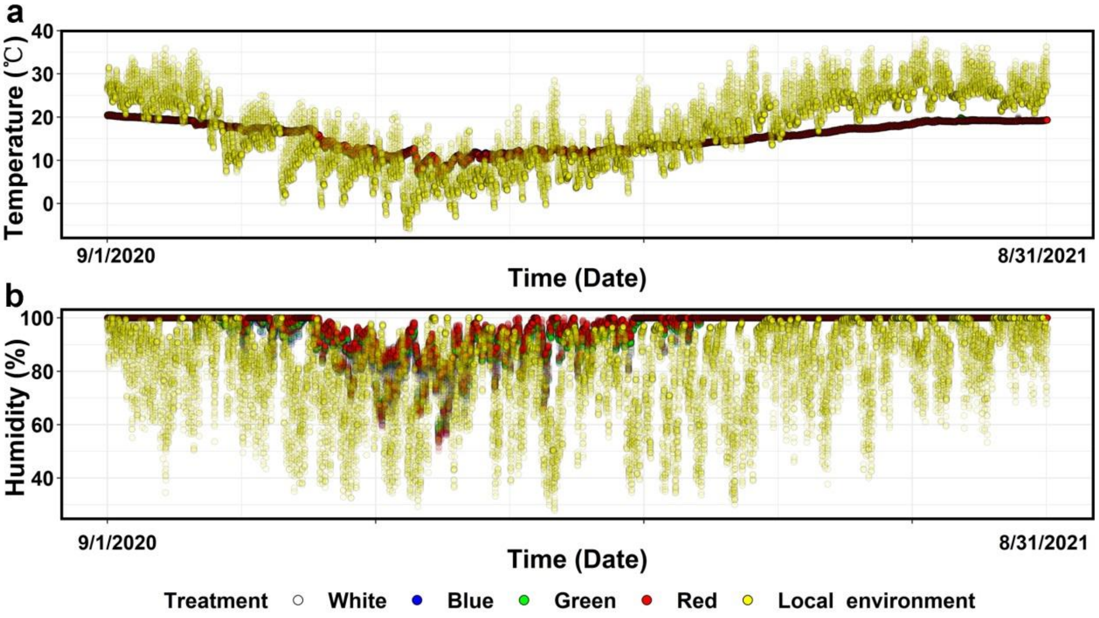

Supplementary Figure 8. Annual temperature (a) and humidity (b) of the tomb walls exposure to white, blue, green, and red artificial lights as well as the local environment.

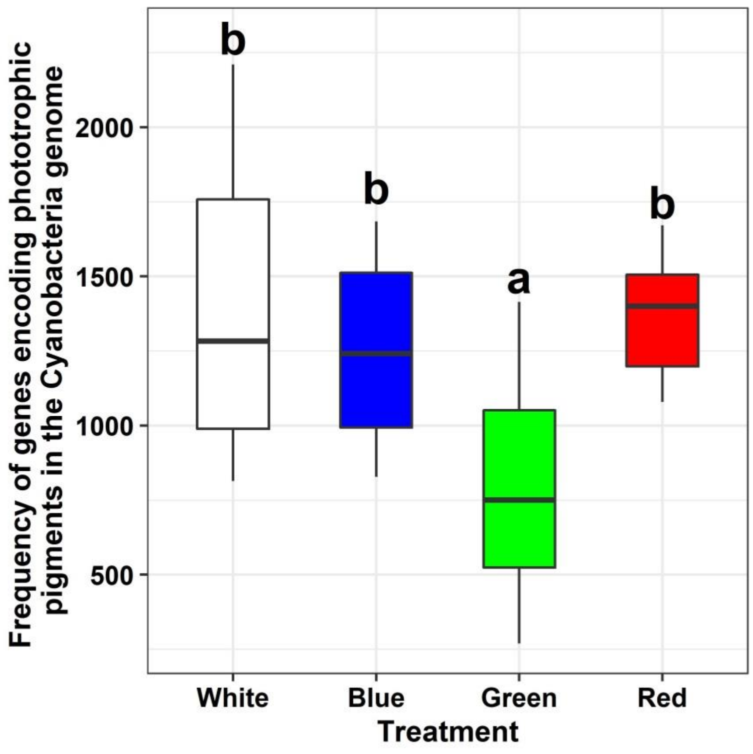

Supplementary Figure 9. Frequency of genes encoding phototrophic pigments in Cyanobacteria genome after 2 years of exposure to blue, green, and red lights, revealed by metagenomic sequencing (n = 40). The centre line indicates the median of the data, the bounds of the box represent the interquartile range, and the whiskers indicate the range of the data, excluding outliers. Different letters over error bars denote significant differences ($P < 0.05$).

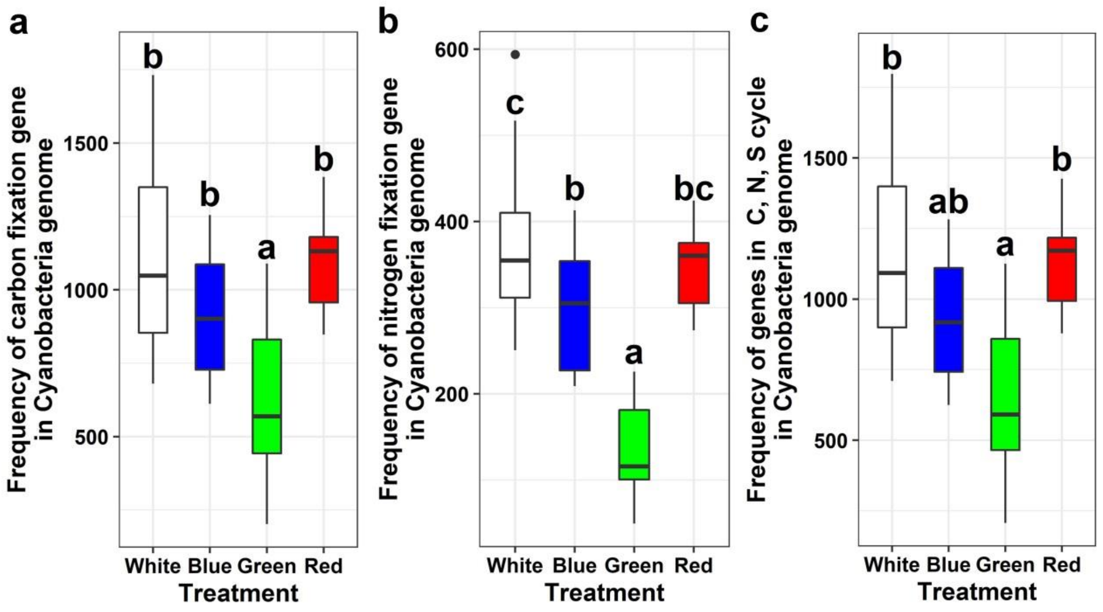

Supplementary Figure 10. Functional profiles of carbon (a) and nitrogen (b) fixation, as well as carbon fixation, denitrification, and dissimilatory sulfate reduction abilities (c) that related to biodeteriation of Cyanobacteria revealed by metagenomic sequencing. The centre line indicates the median of the data, the bounds of the box represent the interquartile range, and the whiskers indicate the range of the data, excluding outliers. Different letters over error bars denote significant differences ($P < 0.05$) (n = 40). For more detailed functional gene profiles, see Supplementary Tables 1 and 5.

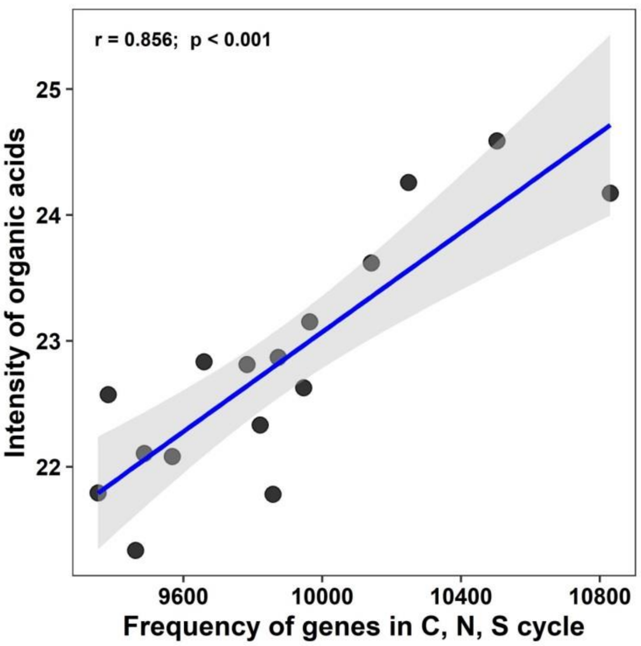

Supplementary Figure 11. Linear regressions between the proportions of genes associated with C, N, and S cycling and the excreted organic acids of the phototrophic bacteriome (n = 16). Linear models (shown as blue lines) and associated correlation coefficients (grey area) are provided on the panel.

Supplementary Table 1. Genes involved in carbon fixation, denitrification, and dissimilatory sulfate reduction related to biodeterioration.

Gene	KEGG Orthology (KO)	Function	Description
rbcL	K01601	Reductive pentose phosphate cycle (Calvin cycle)	ribulose-bisphosphate carboxylase large chain [EC:4.1.1.39]
rbcS	K01602	Reductive pentose phosphate cycle (Calvin cycle)	ribulose-bisphosphate carboxylase small chain [EC:4.1.1.39]
GAPA	K05298	Reductive pentose phosphate cycle (Calvin cycle)	glyceraldehyde-3-phosphate dehydrogenase (NADP+) (phosphorylating) [EC:1.2.1.13]
gap2	K00150	Reductive pentose phosphate cycle (Calvin cycle)	glyceraldehyde-3-phosphate dehydrogenase (NAD(P)) [EC:1.2.1.59]
GAPDH	K00134	Reductive pentose phosphate cycle (Calvin cycle)	glyceraldehyde 3-phosphate dehydrogenase (phosphorylating) [EC:1.2.1.12]
ALDO	K01623	Reductive pentose phosphate cycle (Calvin cycle)	fructose-bisphosphate aldolase, class I [EC:4.1.2.13]
FBA	K01624	Reductive pentose phosphate cycle (Calvin cycle)	fructose-bisphosphate aldolase, class II [EC:4.1.2.13]
FBP	K03841	Reductive pentose phosphate cycle (Calvin cycle)	fructose-1,6-bisphosphatase I [EC:3.1.3.11]
glpX	K02446	Reductive pentose phosphate cycle (Calvin cycle)	fructose-1,6-bisphosphatase II [EC:3.1.3.11]
glpX-SEBP	K11532	Reductive pentose phosphate cycle (Calvin cycle)	fructose-1,6-bisphosphatase II / sedoheptulose-1,7-bisphosphatase [EC:3.1.3.11 3.1.3.37]
fbp-SEBP	K01086	Reductive pentose phosphate cycle (Calvin cycle)	fructose-1,6-bisphosphatase I / sedoheptulose-1,7-bisphosphatase [EC:3.1.3.11 3.1.3.37]
E2.2.1.1	K00615	Reductive pentose phosphate cycle (Calvin cycle)	transketolase [EC:2.2.1.1]
E3.1.3.37	K01100	Reductive pentose phosphate cycle (Calvin cycle)	sedoheptulose-bisphosphatase [EC:3.1.3.37]
rpiA	K01807	Reductive pentose phosphate cycle (Calvin cycle)	ribose 5-phosphate isomerase A [EC:5.3.1.6]
rpiB	K01808	Reductive pentose phosphate cycle (Calvin cycle)	ribose 5-phosphate isomerase B [EC:5.3.1.6]
porA	K00169	Reductive citrate cycle (Arnon-Buchanan cycle)	pyruvate ferredoxin oxidoreductase alpha subunit [EC:1.2.7.1]
porB	K00170	Reductive citrate cycle (Arnon-Buchanan cycle)	pyruvate ferredoxin oxidoreductase beta subunit [EC:1.2.7.1]
porD	K00171	Reductive citrate cycle (Arnon-Buchanan cycle)	pyruvate ferredoxin oxidoreductase delta subunit [EC:1.2.7.1]
porC	K00172	Reductive citrate cycle (Arnon-Buchanan cycle)	pyruvate ferredoxin oxidoreductase gamma subunit [EC:1.2.7.1]
por	K03737	Reductive citrate cycle (Arnon-Buchanan cycle)	pyruvate-ferredoxin/flavodoxin oxidoreductase [EC:1.2.7.1 1.2.7.-]
pps	K01007	Reductive citrate cycle (Arnon-Buchanan cycle)	pyruvate, water dikinase [EC:2.7.9.2]
ppdK	K01006	Reductive citrate cycle (Arnon-Buchanan cycle)	orthophosphate dikinase [EC:2.7.9.1]
ppc	K01595	Reductive citrate cycle (Arnon-Buchanan cycle)	phosphoenolpyruvate carboxylase [EC:4.1.1.31]

pycA	K01959	Reductive citrate cycle (Arnon-Buchanan cycle)	pyruvate carboxylase subunit A [EC:6.4.1.1]
pycB	K01960	Reductive citrate cycle (Arnon-Buchanan cycle)	pyruvate carboxylase subunit B [EC:6.4.1.1]
PC	K01958	Reductive citrate cycle (Arnon-Buchanan cycle)	pyruvate carboxylase [EC:6.4.1.1]
mdh	K00024	Reductive citrate cycle (Arnon-Buchanan cycle)	malate dehydrogenase [EC:1.1.1.37]
E4.2.1.2A	K01676	Reductive citrate cycle (Arnon-Buchanan cycle)	fumarate hydratase, class I [EC:4.2.1.2]
E4.2.1.2B	K01679	Reductive citrate cycle (Arnon-Buchanan cycle)	fumarate hydratase, class II [EC:4.2.1.2]
E4.2.1.2AA	K01677	Reductive citrate cycle (Arnon-Buchanan cycle)	fumarate hydratase subunit alpha [EC:4.2.1.2]
E4.2.1.2AB	K01678	Reductive citrate cycle (Arnon-Buchanan cycle)	fumarate hydratase subunit beta [EC:4.2.1.2]
sdhA	K00239	Reductive citrate cycle (Arnon-Buchanan cycle)	succinate dehydrogenase / fumarate reductase, flavoprotein subunit [EC:1.3.5.1 1.3.5.4]
sdhB	K00240	Reductive citrate cycle (Arnon-Buchanan cycle)	succinate dehydrogenase / fumarate reductase, iron-sulfur subunit [EC:1.3.5.1 1.3.5.4]
sdhC	K00241	Reductive citrate cycle (Arnon-Buchanan cycle)	succinate dehydrogenase / fumarate reductase, cytochrome b subunit
sdhD	K00242	Reductive citrate cycle (Arnon-Buchanan cycle)	succinate dehydrogenase / fumarate reductase, membrane anchor subunit
frdA	K00244	Reductive citrate cycle (Arnon-Buchanan cycle)	fumarate reductase flavoprotein subunit [EC:1.3.5.4]
frdB	K00245	Reductive citrate cycle (Arnon-Buchanan cycle)	fumarate reductase iron-sulfur subunit [EC:1.3.5.4]
frdC	K00246	Reductive citrate cycle (Arnon-Buchanan cycle)	fumarate reductase subunit C
frdD	K00247	Reductive citrate cycle (Arnon-Buchanan cycle)	fumarate reductase subunit D
sucD	K01902	Reductive citrate cycle (Arnon-Buchanan cycle)	succinyl-CoA synthetase alpha subunit [EC:6.2.1.5]
sucC	K01903	Reductive citrate cycle (Arnon-Buchanan cycle)	succinyl-CoA synthetase beta subunit [EC:6.2.1.5]
korA	K00174	Reductive citrate cycle (Arnon-Buchanan cycle)	2-oxoglutarate/2-oxoacid ferredoxin oxidoreductase subunit alpha [EC:1.2.7.3 1.2.7.11]
korB	K00175	Reductive citrate cycle (Arnon-Buchanan cycle)	2-oxoglutarate/2-oxoacid ferredoxin oxidoreductase subunit beta [EC:1.2.7.3 1.2.7.11]
korC	K00177	Reductive citrate cycle (Arnon-Buchanan cycle)	2-oxoglutarate ferredoxin oxidoreductase subunit gamma [EC:1.2.7.3]
korD	K00176	Reductive citrate cycle (Arnon-Buchanan cycle)	2-oxoglutarate ferredoxin oxidoreductase subunit delta [EC:1.2.7.3]
IDH1	K00031	Reductive citrate cycle (Arnon-Buchanan cycle)	isocitrate dehydrogenase [EC:1.1.1.42]
ACO	K01681	Reductive citrate cycle (Arnon-Buchanan cycle)	aconitate hydratase [EC:4.2.1.3]
acnB	K01682	Reductive citrate cycle (Arnon-Buchanan cycle)	aconitate hydratase 2 / 2-methylisocitrate dehydratase [EC:4.2.1.3 4.2.1.99]
aclA	K15230	Reductive citrate cycle (Arnon-Buchanan cycle)	ATP-citrate lyase alpha-subunit [EC:2.3.3.8]
aclB	K15231	Reductive citrate cycle (Arnon-Buchanan cycle)	ATP-citrate lyase beta-subunit [EC:2.3.3.8]

ccsA	K15232	Reductive citrate cycle (Arnon-Buchanan cycle)	citryl-CoA synthetase large subunit [EC:6.2.1.18]
ccl	K15234	Reductive citrate cycle (Arnon-Buchanan cycle)	citryl-CoA lyase [EC:4.1.3.34]
accB	K02160	3-Hydroxypropionate bi-cycle	acetyl-CoA carboxylase biotin carboxyl carrier protein
accC	K01961	3-Hydroxypropionate bi-cycle	acetyl-CoA carboxylase, biotin carboxylase subunit [EC:6.4.1.2 6.3.4.14]
accA	K01962	3-Hydroxypropionate bi-cycle	acetyl-CoA carboxylase carboxyl transferase subunit alpha [EC:6.4.1.2 2.1.3.15]
accD	K01963	3-Hydroxypropionate bi-cycle	acetyl-CoA carboxylase carboxyl transferase subunit beta [EC:6.4.1.2 2.1.3.15]
mcr	K14468	3-Hydroxypropionate bi-cycle	malonyl-CoA reductase / 3-hydroxypropionate dehydrogenase (NADP+) [EC:1.2.1.75 1.1.1.298]
K14469	K14469	3-Hydroxypropionate bi-cycle	acrylyl-CoA reductase (NADPH) / 3-hydroxypropionyl-CoA dehydratase / 3-hydroxypropionyl-CoA synthetase [EC:1.3.1.84 4.2.1.116 6.2.1.36]
K15052	K15052	3-Hydroxypropionate bi-cycle	propionyl-CoA carboxylase [EC:6.4.1.3 2.1.3.15]
MCEE	K05606	3-Hydroxypropionate bi-cycle	methylmalonyl-CoA/ethylmalonyl-CoA epimerase [EC:5.1.99.1]
MUT	K01847	3-Hydroxypropionate bi-cycle	methylmalonyl-CoA mutase [EC:5.4.99.2]
E5.4.99.2A	K01848	3-Hydroxypropionate bi-cycle	methylmalonyl-CoA mutase, N-terminal domain [EC:5.4.99.2]
E5.4.99.2B	K01849	3-Hydroxypropionate bi-cycle	methylmalonyl-CoA mutase, C-terminal domain [EC:5.4.99.2]
smtA1	K14471	3-Hydroxypropionate bi-cycle	succinyl-CoA:(S)-malate CoA-transferase subunit A [EC:2.8.3.22]
smtB	K14472	3-Hydroxypropionate bi-cycle	succinyl-CoA:(S)-malate CoA-transferase subunit B [EC:2.8.3.22]
mcl	K08691	3-Hydroxypropionate bi-cycle	malyl-CoA/(S)-citramalyl-CoA lyase [EC:4.1.3.24 4.1.3.25]
mch	K14449	3-Hydroxypropionate bi-cycle	2-methylfumaryl-CoA hydratase [EC:4.2.1.148]
mct	K14470	3-Hydroxypropionate bi-cycle	2-methylfumaryl-CoA isomerase [EC:5.4.1.3]
meh	K09709	3-Hydroxypropionate bi-cycle	3-methylfumaryl-CoA hydratase [EC:4.2.1.153]
K15019	K15019	Hydroxypropionate-hydroxybutylate cycle	3-hydroxypropionyl-coenzyme A dehydratase [EC:4.2.1.116]
K15020	K15020	Hydroxypropionate-hydroxybutylate cycle	acryloyl-coenzyme A reductase [EC:1.3.1.84]
K14465	K14465	Hydroxypropionate-hydroxybutylate cycle	succinate semialdehyde reductase (NADPH) [EC:1.1.1.-]
abfD	K14534	Hydroxypropionate-hydroxybutylate cycle	4-hydroxybutyryl-CoA dehydratase / vinylacetyl-CoA-Delta-isomerase [EC:4.2.1.120 5.3.3.3]
K15016	K15016	Hydroxypropionate-hydroxybutylate cycle	enoyl-CoA hydratase / 3-hydroxyacyl-CoA dehydrogenase [EC:4.2.1.17 1.1.1.35]
ACAT	K00626	Hydroxypropionate-hydroxybutylate cycle	acetyl-CoA C-acetyltransferase [EC:2.3.1.9]
fdhA	K05299	Reductive acetyl-CoA pathway (Wood-Ljungdahl pathway)	formate dehydrogenase (NADP+) alpha subunit [EC:1.17.1.10]
fdhB	K15022	Reductive acetyl-CoA pathway (Wood-Ljungdahl pathway)	formate dehydrogenase (NADP+) beta subunit [EC:1.17.1.10]

fdhF	K22015	Reductive acetyl-CoA pathway (Wood-Ljungdahl pathway)	formate dehydrogenase (hydrogenase) [EC:1.17.98.4 1.17.98.-]
fhs	K01938	Reductive acetyl-CoA pathway (Wood-Ljungdahl pathway)	formate--tetrahydrofolate ligase [EC:6.3.4.3]
folD	K01491	Reductive acetyl-CoA pathway (Wood-Ljungdahl pathway)	methylenetetrahydrofolate dehydrogenase (NADP+) / methenyltetrahydrofolate cyclohydrolase [EC:1.5.1.5 3.5.4.9]
fchA	K01500	Reductive acetyl-CoA pathway (Wood-Ljungdahl pathway)	methenyltetrahydrofolate cyclohydrolase [EC:3.5.4.9]
metF	K00297	Reductive acetyl-CoA pathway (Wood-Ljungdahl pathway)	methylenetetrahydrofolate reductase (NADH) [EC:1.5.1.54]
acsE	K15023	Reductive acetyl-CoA pathway (Wood-Ljungdahl pathway)	5-methyltetrahydrofolate corrinoid/iron sulfur protein methyltransferase [EC:2.1.1.258]
tfrA	K18209	Incomplete reductive citrate cycle	fumarate reductase (CoM/CoB) subunit A [EC:1.3.4.1]
tfrB	K18210	Incomplete reductive citrate cycle	fumarate reductase (CoM/CoB) subunit B [EC:1.3.4.1]
nirS	K15864	Denitrification	nitrite reductase (NO-forming) / hydroxylamine reductase [EC:1.7.2.1 1.7.99.1]
nirK	K00368	Denitrification	nitrite reductase (NO-forming) [EC:1.7.2.1]
norB	K04561	Denitrification	nitric oxide reductase subunit B [EC:1.7.2.5]
norC	K02305	Denitrification	nitric oxide reductase subunit C
nosZ	K00376	Denitrification	nitrous-oxide reductase [EC:1.7.2.4]
narG/nxrA	K00370	Denitrification	nitrate reductase / nitrite oxidoreductase, alpha subunit [EC:1.7.5.1 1.7.99.-]
narH/nxrB	K00371	Denitrification	nitrate reductase / nitrite oxidoreductase, beta subunit [EC:1.7.5.1 1.7.99.-]
narI	K00374	Denitrification	nitrate reductase gamma subunit [EC:1.7.5.1 1.7.99.-]
napA	K02567	Denitrification	nitrate reductase (cytochrome) [EC:1.9.6.1]
napB	K02568	Denitrification	nitrate reductase (cytochrome), electron transfer subunit
napC	K02569	Denitrification	cytochrome c-type protein NapC
sat	K00958	Dissimilatory sulfate reduction	sulfate adenylyltransferase [EC:2.7.7.4]
aprA	K00394	Dissimilatory sulfate reduction	adenylylsulfate reductase, subunit A [EC:1.8.99.2]
aprB	K00395	Dissimilatory sulfate reduction	adenylylsulfate reductase, subunit B [EC:1.8.99.2]
dsrA	K11180	Dissimilatory sulfate reduction	dissimilatory sulfite reductase alpha subunit [EC:1.8.99.5]
dsrB	K11181	Dissimilatory sulfate reduction	dissimilatory sulfite reductase beta subunit [EC:1.8.99.5]

Supplementary Table 2. PERMANOVA shows the community dissimilarities of the Cyanobacteria-oriented phototrophic bacteriome between different lights (that is, white, blue, green, and red) based on the Bray-Curtis distance of the bacterial OTU table annotated by amplicon sequencing (n = 40).

Pairs	F.Model	R^2	*P* <
Global test	6.461	0.350	0.001
White *vs*. Blue	8.917	0.331	0.001
White *vs*. Green	17.120	0.487	0.001
White *vs*. Red	8.730	0.327	0.001
Blue *vs*. Green	18.164	0.502	0.001
Blue *vs*. Red	17.595	0.494	0.001
Green *vs*. Red	20.060	0.527	0.001

Supplementary Table 3. PERMANOVA shows the functional dissimilarities of the Cyanobacteria-oriented phototrophic bacteriome between different lights (that is, white, blue, green, and red) based on the Bray-Curtis distance bacterial KEGG Orthologies annotated by metagenomic sequencing (n = 40).

Pairs	F.Model	R^2	$P <$
Global test	11.580	0.491	0.001
White *vs*. Blue	9.433	0.344	0.001
White *vs*. Green	11.651	0.393	0.001
White *vs*. Red	11.749	0.395	0.001
Blue *vs*. Green	10.705	0.373	0.001
Blue *vs*. Red	16.192	0.474	0.001
Green *vs*. Red	11.138	0.382	0.001

Supplementary Table 4. Genes involved in nitrogen fixation.

Gene	KEGG Orthology (KO)	Function	Description
nifD	K02586	Nitrogen fixation	nitrogenase molybdenum-iron protein alpha chain [EC:1.18.6.1]
nifH	K02588	Nitrogen fixation	nitrogenase iron protein NifH
nifK	K02591	Nitrogen fixation	nitrogenase molybdenum-iron protein beta chain [EC:1.18.6.1]
vnfK	K22897	Nitrogen fixation	vanadium-dependent nitrogenase beta chain [EC:1.18.6.2]
NRT	K02575	Nitrate assimilation/Nitrogen transport	MFS transporter, NNP family, nitrate/nitrite transporter
nrtA	K15576	Nitrate assimilation/Nitrogen transport	nitrate/nitrite transport system substrate-binding protein
nrtB	K15577	Nitrate assimilation/Nitrogen transport	nitrate/nitrite transport system permease protein
nrtC	K15578	Nitrate assimilation/Nitrogen transport	nitrate/nitrite transport system ATP-binding protein [EC:7.3.2.4]
nrtD	K15579	Nitrate assimilation/Nitrogen transport	nitrate/nitrite transport system ATP-binding protein [EC:7.3.2.4]

Supplementary Table 5. Taxa suppressed under blue, green and red lights, compared to white light

Taxa abundances significantlly decreased under blue light when compared to white light

Phylum	Genus	White-Mean (%	White-Sd (%	Blue-Mean (%	Blue-Sd (%	P value	Corrected pvalu	Lower ci	Upper ci	Effect size
p__Cyanobacteria	g__Aphanothece	0.1074	0.1102	0.02587	0.006821	0.03764	0.06884	-0.1527	-0.02242	-0.08155
p__Cyanobacteria	g__Myxacorys	0.08901	0.02301	0.04027	0.0102	0.000183	0.001654	-0.06313	-0.03463	-0.04874
p__Cyanobacteria	g__Hydrococcus_f__Hydrococcaceae	0.1009	0.02829	0.07314	0.01787	0.02575	0.05065	-0.04878	-0.00726	-0.02781
p__Cyanobacteria	g__Rippkaea	0.02975	0.01239	0.01089	0.003897	0.000583	0.002674	-0.02654	-0.01142	-0.01885
p__Cyanobacteria	g__Synechococcus	0.04956	0.01043	0.03681	0.00603	0.004586	0.01226	-0.02041	-0.00553	-0.01275
p__Cyanobacteria	g__Chlorogloea	0.02792	0.007361	0.01638	0.003662	0.000769	0.003257	-0.01671	-0.00698	-0.01154
p__Cyanobacteria	g__Lyngbya	0.02326	0.004845	0.01258	0.003239	0.00033	0.002032	-0.01413	-0.00735	-0.01067
p__Cyanobacteria	g__Crocosphaera	0.02446	0.01051	0.01425	0.003393	0.01133	0.02551	-0.01713	-0.004	-0.01021
p__Cyanobacteria	g__unclassified_o__Synechococcales	0.02888	0.007991	0.02018	0.004177	0.01133	0.02551	-0.01393	-0.0035	-0.0087
p__Cyanobacteria	g__Spirulina	0.01464	0.004223	0.006174	0.000698	0.000183	0.001654	-0.01109	-0.00609	-0.00847
p__Cyanobacteria	g__Chroogloeocystis	0.02197	0.006029	0.01478	0.003694	0.007285	0.01777	-0.01117	-0.00298	-0.00719
p__Cyanobacteria	g__unclassified_f__Oscillatoriaceae	0.007656	0.002195	0.001031	0.000249	0.000183	0.001654	-0.00799	-0.0054	-0.00663
p__Cyanobacteria	g__unclassified_f__Acaryochloridaceae	0.02143	0.006436	0.01486	0.003055	0.01133	0.02551	-0.0113	-0.00243	-0.00658
p__Cyanobacteria	g__Nodularia_f__Aphanizomenonaceae	0.02236	0.005748	0.01633	0.00388	0.01726	0.03608	-0.0103	-0.00186	-0.00604
p__Cyanobacteria	g__Gloeomargarita	0.01803	0.004517	0.01262	0.00324	0.01726	0.03608	-0.00873	-0.00212	-0.00541
p__Cyanobacteria	g__Dolichospermum	0.01038	0.001847	0.005131	0.000388	0.000183	0.001654	-0.00642	-0.00416	-0.00525
p__Cyanobacteria	g__Hapalosiphon	0.01006	0.003254	0.006244	0.001172	0.005795	0.01483	-0.00575	-0.00171	-0.00382
p__Cyanobacteria	g__Myxosarcina	0.007337	0.002002	0.005048	0.001438	0.01133	0.02551	-0.00383	-0.00083	-0.00229
p__Cyanobacteria	g__Geminocystis	0.004217	0.001239	0.002759	0.000505	0.001706	0.00577	-0.00223	-0.00076	-0.00146
p__Cyanobacteria	g__Euhalothece	0.002336	0.0005445	0.001099	0.000263	0.000183	0.001654	-0.00162	-0.00088	-0.00124
p__Cyanobacteria	g__Hydrocoleum	0.002585	0.000448	0.00151	0.000342	0.00033	0.002032	-0.00142	-0.00071	-0.00108
p__Cyanobacteria	g__Trichodesmium	0.001088	0.0003422	0.0003264	0.000158	0.00033	0.002032	-0.00098	-0.00054	-0.00076
p__Cyanobacteria	g__Dactylococcopsis	0.001132	0.0003781	0.0006422	0.000253	0.002827	0.00826	-0.00078	-0.00022	-0.00049
p__Cyanobacteria	g__Prochloron	0.0004801	0.000639	0.0001018	4.44E-05	0.01133	0.02551	-0.00079	-0.00011	-0.00038
p__Cyanobacteria	g__unclassified_f__Aphanizomenonaceae	0.0003858	0.0001464	1.01E-05	7.31E-06	0.000183	0.001654	-0.00045	-0.00029	-0.00038
p__Cyanobacteria	g__Candidatus_Atelocyanobacterium	9.88E-05	3.90E-05	3.36E-05	2.72E-05	0.003611	0.01003	-9.18E-05	-3.42E-05	-6.52E-05
p__Cyanobacteria	g__Roholtiella	6.75E-06	1.14E-05	0	0	0.01493	0.03223	-1.42E-05	-9.36E-07	-6.75E-06
p__Proteobacteria	g__Sphingomonas	3.913	0.71	1.52	0.5535	0.000183	0.001654	-2.907	-1.795	-2.392
p__Proteobacteria	g__Hyphomicrobium	1.329	0.3437	0.5824	0.09739	0.000183	0.001654	-0.9615	-0.5451	-0.7467
p__Proteobacteria	g__Luteimonas	0.6334	0.8375	0.1359	0.01984	0.004586	0.01226	-1.071	-0.08762	-0.4975
p__Proteobacteria	g__Pseudoxanthomonas	0.9661	0.4535	0.5241	0.2927	0.02575	0.05065	-0.7663	-0.1111	-0.442
p__Proteobacteria	g__unclassified_f__Phyllobacteriaceae	0.7923	0.2451	0.4658	0.2178	0.004586	0.01226	-0.5286	-0.132	-0.3264
p__Proteobacteria	g__Methylibium	0.6906	0.1858	0.4796	0.1092	0.003611	0.01003	-0.3494	-0.09456	-0.2111
p__Proteobacteria	g__Aquincola	0.2786	0.1277	0.0911	0.03344	0.000183	0.001654	-0.2831	-0.1159	-0.1875
p__Proteobacteria	g__Panacagrimonas	0.2002	0.07216	0.03179	0.006449	0.000183	0.001654	-0.2132	-0.1277	-0.1684
p__Proteobacteria	g__unclassified_f__Comamonadaceae	0.3602	0.1297	0.2082	0.06919	0.002827	0.00826	-0.2411	-0.06579	-0.1519
p__Proteobacteria	g__unclassified_f__Hyphomicrobiaceae	0.5193	0.123	0.3731	0.07989	0.01726	0.03608	-0.2311	-0.05846	-0.1462
p__Proteobacteria	g__Rubrivivax	0.3105	0.1054	0.1809	0.04537	0.002202	0.00693	-0.2049	-0.06618	-0.1296
p__Proteobacteria	g__Sphingosinicella	0.223	0.03469	0.1137	0.02278	0.000183	0.001654	-0.1337	-0.08689	-0.1093
p__Proteobacteria	g__Novosphingobium	0.2616	0.04393	0.1657	0.03866	0.000583	0.002674	-0.1315	-0.05948	-0.0959
p__Proteobacteria	g__Azohydromonas	0.1651	0.06505	0.08136	0.02838	0.001008	0.003952	-0.1295	-0.04122	-0.08375
p__Proteobacteria	g__Nordella	0.1452	0.02864	0.06669	0.02261	0.00044	0.002332	-0.09865	-0.05638	-0.07852
p__Proteobacteria	g__unclassified_o__Burkholderiales	0.3688	0.09154	0.2965	0.07222	0.03121	0.05963	-0.1413	-0.0006	-0.07232
p__Proteobacteria	g__unclassified_f__Sphingomonadaceae	0.2537	0.05503	0.1825	0.07233	0.03764	0.06884	-0.1243	-0.01095	-0.07126

p__Proteobacteria	g__Sphingobium	0.1998	0.02959	0.1344	0.03557	0.002202	0.00693	-0.09205	-0.037	-0.06547
p__Proteobacteria	g__Ideonella	0.218	0.06468	0.1585	0.04281	0.02113	0.04273	-0.1075	-0.018	-0.05956
p__Proteobacteria	g__Chakrabartia	0.1211	0.03332	0.07239	0.04464	0.01402	0.03047	-0.07844	-0.01363	-0.04868
p__Proteobacteria	g__Filomicrobium	0.07029	0.03025	0.02488	0.007539	0.00044	0.002332	-0.06442	-0.02617	-0.04541
p__Proteobacteria	g__Piscinibacter	0.2267	0.07613	0.1848	0.1158	0.04515	0.08009	-0.118	0.0443	-0.04196
p__Proteobacteria	g__Altererythrobacter	0.09315	0.01491	0.0544	0.01171	0.000583	0.002674	-0.04989	-0.02698	-0.03875
p__Proteobacteria	g__Erythrobacter	0.07671	0.00967	0.04496	0.009942	0.000246	0.001813	-0.0402	-0.02339	-0.03175
p__Proteobacteria	g__Rivibacter	0.09552	0.02206	0.06388	0.01765	0.002202	0.00693	-0.04775	-0.01401	-0.03163
p__Proteobacteria	g__Hydrogenophaga	0.09053	0.01841	0.06254	0.009703	0.002827	0.00826	-0.04095	-0.01613	-0.02799
p__Proteobacteria	g__Aquabacterium	0.1096	0.03046	0.08175	0.02331	0.01402	0.03047	-0.051	-0.00493	-0.02788
p__Proteobacteria	g__Polaromonas	0.08773	0.02542	0.06271	0.0131	0.005795	0.01483	-0.0434	-0.00989	-0.02502
p__Proteobacteria	g__unclassified_f__Burkholderiaceae	0.1257	0.03581	0.1008	0.03541	0.03764	0.06884	-0.05394	0.006767	-0.02496
p__Proteobacteria	g__Ramlibacter	0.09593	0.02766	0.07499	0.01691	0.02575	0.05065	-0.0412	-0.00287	-0.02094
p__Proteobacteria	g__Blastomonas	0.04658	0.00698	0.02675	0.01155	0.002827	0.00826	-0.0277	-0.01182	-0.01982
p__Proteobacteria	g__Caulobacter	0.162	0.01129	0.1438	0.01098	0.009108	0.0215	-0.02758	-0.00947	-0.01815
p__Proteobacteria	g__Parasphingopyxis	0.02435	0.00469	0.006309	0.002799	0.000183	0.001654	-0.02133	-0.01491	-0.01804
p__Proteobacteria	g__Skermanella	0.07414	0.01433	0.05789	0.005849	0.004586	0.01226	-0.02559	-0.00754	-0.01625
p__Proteobacteria	g__Zhizhongheella	0.0396	0.01242	0.02501	0.008979	0.002827	0.00826	-0.02347	-0.0055	-0.01459
p__Proteobacteria	g__Porphyrobacter	0.03089	0.004664	0.01654	0.003901	0.000246	0.001813	-0.01767	-0.01081	-0.01434
p__Proteobacteria	g__Sandarakinorhabdus	0.03103	0.00531	0.01706	0.00513	0.000583	0.002674	-0.01813	-0.00932	-0.01398
p__Proteobacteria	g__Roseomonas	0.09032	0.01098	0.07656	0.009419	0.01133	0.02551	-0.02162	-0.00552	-0.01376
p__Proteobacteria	g__Polycyclovorans	0.01904	0.00638	0.006079	0.001246	0.000183	0.001654	-0.01661	-0.00935	-0.01296
p__Proteobacteria	g__Rhodoplanes	0.0874	0.01019	0.07564	0.01489	0.03764	0.06884	-0.02235	-0.00151	-0.01176
p__Proteobacteria	g__Parasphingorhabdus	0.0198	0.003512	0.00915	0.002178	0.000183	0.001654	-0.01303	-0.00806	-0.01065
p__Proteobacteria	g__Tatlockia	0.01401	0.006246	0.004108	0.001717	0.000769	0.003257	-0.01383	-0.00603	-0.0099
p__Proteobacteria	g__Simplicispira	0.01666	0.007179	0.00712	0.002025	0.000246	0.001813	-0.01439	-0.00543	-0.00954
p__Proteobacteria	g__Oceanibaculum	0.03674	0.005047	0.0278	0.003324	0.001315	0.004744	-0.01242	-0.00539	-0.00894
p__Proteobacteria	g__Silvanigrella	0.009192	0.005861	0.0004563	0.000241	0.000183	0.001654	-0.01271	-0.00572	-0.00874
p__Proteobacteria	g__Leptothrix_r__root	0.03378	0.009285	0.02531	0.008521	0.01726	0.03608	-0.01582	-0.00132	-0.00847
p__Proteobacteria	g__Tistlia	0.03691	0.006878	0.02855	0.008946	0.02575	0.05065	-0.0148	-0.00117	-0.00836
p__Proteobacteria	g__Defluviicoccus	0.02358	0.003635	0.01645	0.002469	0.00044	0.002332	-0.00986	-0.00464	-0.00713
p__Proteobacteria	g__Caldimonas	0.02123	0.007086	0.0142	0.005825	0.003611	0.01003	-0.01242	-0.00133	-0.00703
p__Proteobacteria	g__unclassified_f__Ectothiorhodospiraceae	0.01839	0.007839	0.01153	0.002698	0.009108	0.0215	-0.01189	-0.00234	-0.00686
p__Proteobacteria	g__Novosphingopyxis	0.007942	0.001586	0.001186	0.0003	0.000183	0.001654	-0.00774	-0.00586	-0.00676
p__Proteobacteria	g__Hephaestia	0.01166	0.001482	0.006176	0.003381	0.002827	0.00826	-0.00751	-0.00309	-0.00548
p__Proteobacteria	g__Xanthobacter	0.02802	0.005578	0.02259	0.004402	0.04515	0.08009	-0.00932	-0.00121	-0.00544
p__Proteobacteria	g__Ottowia	0.01642	0.005123	0.01126	0.001548	0.002202	0.00693	-0.00852	-0.00226	-0.00517
p__Proteobacteria	g__unclassified_f__Kiloniellaceae	0.03595	0.0061	0.03104	0.008138	0.02113	0.04273	-0.01046	0.001625	-0.00491
p__Proteobacteria	g__Legionella	0.01409	0.002819	0.009241	0.001386	0.00033	0.002032	-0.00689	-0.00293	-0.00485
p__Proteobacteria	g__Methylocystis	0.02448	0.002144	0.0197	0.001125	0.000183	0.001654	-0.00626	-0.00329	-0.00478
p__Proteobacteria	g__Pacificimonas	0.007048	0.001407	0.002311	0.000647	0.000183	0.001654	-0.00554	-0.00375	-0.00474
p__Proteobacteria	g__unclassified_f__Geminicoccaceae	0.01324	0.003896	0.008755	0.003232	0.01402	0.03047	-0.00758	-0.00139	-0.00449
p__Proteobacteria	g__Stakelama	0.006273	0.00112	0.001916	0.00086	0.000183	0.001654	-0.00513	-0.00353	-0.00436
p__Proteobacteria	g__Ferrovibrio	0.02363	0.003977	0.01943	0.002033	0.01133	0.02551	-0.00684	-0.00171	-0.0042
p__Proteobacteria	g__Phreatobacter	0.03647	0.00369	0.03236	0.002641	0.009108	0.0215	-0.00678	-0.00127	-0.00412
p__Proteobacteria	g__Telmatospirillum	0.0231	0.003792	0.01916	0.002155	0.02113	0.04273	-0.0066	-0.00152	-0.00395
p__Proteobacteria	g__Sandaracinobacter	0.00595	0.001019	0.002225	0.000552	0.000183	0.001654	-0.00444	-0.00301	-0.00373
p__Proteobacteria	g__unclassified_o__Silvanigrellales	0.003994	0.002622	0.0002757	9.06E-05	0.000183	0.001654	-0.00539	-0.00235	-0.00372

p__Proteobacteria	g__unclassified_o__Caulobacterales	0.01528	0.002649	0.01184	0.001439	0.003611	0.01003	-0.00523	-0.00153	-0.00343
p__Proteobacteria	g__Fluviispira	0.003581	0.002261	0.0001927	7.94E-05	0.000183	0.001654	-0.00481	-0.00223	-0.00339
p__Proteobacteria	g__Pelagibius	0.02299	0.002864	0.01974	0.005028	0.03121	0.05963	-0.00619	0.000289	-0.00325
p__Proteobacteria	g__Azorhizobium	0.01596	0.003719	0.01283	0.002977	0.04515	0.08009	-0.00612	-0.00046	-0.00313
p__Proteobacteria	g__Citromicrobium	0.007982	0.00104	0.004942	0.001107	0.00033	0.002032	-0.0039	-0.00218	-0.00304
p__Proteobacteria	g__Thalassospira	0.01365	0.001267	0.01078	0.001583	0.000583	0.002674	-0.00404	-0.00162	-0.00287
p__Proteobacteria	g__Sphingosinithalassobacter	0.00567	0.0008371	0.002892	0.001028	0.00033	0.002032	-0.00351	-0.00197	-0.00278
p__Proteobacteria	g__Paracandidimonas	0.003693	0.001546	0.001038	0.000247	0.000183	0.001654	-0.00362	-0.00181	-0.00266
p__Proteobacteria	g__Sulfitobacter	0.009395	0.001086	0.006854	0.000491	0.000183	0.001654	-0.00325	-0.00186	-0.00254
p__Proteobacteria	g__Methylopila	0.009765	0.0008067	0.007351	0.000987	0.00044	0.002332	-0.00318	-0.00163	-0.00241
p__Proteobacteria	g__Methyloceanibacter	0.01301	0.001864	0.01087	0.001101	0.009108	0.0215	-0.00346	-0.00089	-0.00214
p__Proteobacteria	g__Cysteiniphilum	0.002763	0.0006758	0.0008168	0.000186	0.000183	0.001654	-0.0024	-0.00154	-0.00195
p__Proteobacteria	g__Vitreoscilla	0.005503	0.00176	0.00357	0.001163	0.002202	0.00693	-0.00315	-0.00061	-0.00193
p__Proteobacteria	g__Enterobacter	0.003768	0.0007742	0.00195	0.000339	0.000183	0.001654	-0.00231	-0.00135	-0.00182
p__Proteobacteria	g__Indioceanicola	0.01009	0.001413	0.00831	0.000845	0.005795	0.01483	-0.00281	-0.00078	-0.00178
p__Proteobacteria	g__Aquicella	0.002385	0.001435	0.0007496	0.000223	0.000183	0.001654	-0.00262	-0.00093	-0.00164
p__Proteobacteria	g__Croceibacterium	0.002637	0.0005726	0.001017	0.000305	0.000183	0.001654	-0.00198	-0.00124	-0.00162
p__Proteobacteria	g__Salmonella	0.007998	0.0007567	0.00639	0.000537	0.00044	0.002332	-0.00213	-0.00103	-0.00161
p__Proteobacteria	g__Croceicoccus	0.006806	0.001355	0.005224	0.00064	0.01726	0.03608	-0.00246	-0.0007	-0.00158
p__Proteobacteria	g__Acidocella	0.005375	0.0008471	0.003831	0.000598	0.000583	0.002674	-0.00217	-0.00088	-0.00154
p__Proteobacteria	g__Desulfobacterium	0.005465	0.001521	0.003964	0.000649	0.004586	0.01226	-0.00248	-0.00064	-0.0015
p__Proteobacteria	g__Gluconobacter	0.002678	0.0003598	0.001187	0.000239	0.000183	0.001654	-0.00176	-0.00125	-0.00149
p__Proteobacteria	g__Siccirubricoccus	0.007594	0.0008315	0.006125	0.000904	0.005795	0.01483	-0.00224	-0.00078	-0.00147
p__Proteobacteria	g__Roseibaca	0.001621	0.0003391	0.0002148	7.13E-05	0.000183	0.001654	-0.00161	-0.00121	-0.00141
p__Proteobacteria	g__Qipengyuania	0.002825	0.0009584	0.001508	0.000523	0.003611	0.01003	-0.00198	-0.00065	-0.00132
p__Proteobacteria	g__Pseudorhodobacter	0.005505	0.001429	0.004189	0.000612	0.01133	0.02551	-0.00227	-0.00045	-0.00132
p__Proteobacteria	g__Oleisolibacter	0.005748	0.0009043	0.004453	0.000569	0.005795	0.01483	-0.00192	-0.00064	-0.0013
p__Proteobacteria	g__Pseudobacteriovorax	0.002866	0.001503	0.001583	0.000503	0.03121	0.05963	-0.00239	-0.00046	-0.00128
p__Proteobacteria	g__Magnetofaba	0.003649	0.0004566	0.00239	0.000639	0.000583	0.002674	-0.00171	-0.00082	-0.00126
p__Proteobacteria	g__Chromohalobacter	0.001548	0.0004397	0.0002949	0.000104	0.000183	0.001654	-0.00152	-0.00098	-0.00125
p__Proteobacteria	g__Cohaesibacter	0.003007	0.0005635	0.001773	0.000223	0.000183	0.001654	-0.0016	-0.00089	-0.00123
p__Proteobacteria	g__Methylomicrobium	0.008514	0.00099	0.007338	0.000815	0.01133	0.02551	-0.00201	-0.00046	-0.00118
p__Proteobacteria	g__unclassified_f__Methylocystaceae	0.00506	0.0009113	0.003888	0.000314	0.01133	0.02551	-0.0017	-0.00059	-0.00117
p__Proteobacteria	g__Oceanicola	0.003486	0.0004859	0.002322	0.000444	0.00033	0.002032	-0.00156	-0.00079	-0.00116
p__Proteobacteria	g__Rugamonas	0.005311	0.001288	0.004176	0.000573	0.03121	0.05963	-0.00203	-0.00034	-0.00114
p__Proteobacteria	g__Spirobacillus	0.001283	0.0006953	0.0001752	4.14E-05	0.000183	0.001654	-0.00154	-0.00075	-0.00111
p__Proteobacteria	g__Phaeospirillum	0.005445	0.0008916	0.004348	0.000377	0.007285	0.01777	-0.00166	-0.00054	-0.0011
p__Proteobacteria	g__unclassified_f__Bacteriovoracaceae	0.001924	0.0006198	0.0008959	0.000319	0.000583	0.002674	-0.00141	-0.0006	-0.00103
p__Proteobacteria	g__unclassified_o__Sneathiellales	0.001665	0.0003214	0.000662	0.000105	0.000183	0.001654	-0.00119	-0.00079	-0.001
p__Proteobacteria	g__'Geomonas'_Xu_et_al._2019	0.002375	0.0005347	0.001377	0.00032	0.000583	0.002674	-0.00136	-0.00063	-0.001
p__Proteobacteria	g__Defluviimonas	0.004568	0.0003589	0.003603	0.000525	0.000583	0.002674	-0.00134	-0.0006	-0.00096
p__Proteobacteria	g__Malikia	0.002642	0.000817	0.001686	0.000458	0.009108	0.0215	-0.00154	-0.00046	-0.00096
p__Proteobacteria	g__Raoultella	0.003287	0.0004867	0.002352	0.000428	0.001706	0.00577	-0.00129	-0.00056	-0.00094
p__Proteobacteria	g__Maribius	0.001361	0.0004057	0.0004625	0.000144	0.000183	0.001654	-0.00114	-0.00066	-0.0009
p__Proteobacteria	g__Marinovum	0.001766	0.0004361	0.0008927	0.000174	0.00033	0.002032	-0.00116	-0.00059	-0.00087
p__Proteobacteria	g__Roseiarcus	0.005958	0.0006607	0.005138	0.000685	0.01726	0.03608	-0.00138	-0.00024	-0.00082
p__Proteobacteria	g__Magnetospira	0.002937	0.000276	0.002137	0.000431	0.00044	0.002332	-0.00111	-0.0005	-0.0008
p__Proteobacteria	g__unclassified_f__Acidiferrobacteraceae	0.004034	0.0005515	0.003239	0.000546	0.003611	0.01003	-0.00124	-0.00033	-0.00079

p__Proteobacteria	g__Rhodobium_f__Rhodobiaceae	0.003051	0.0003507	0.002271	0.000435	0.001706	0.00577	-0.00109	-0.00046	-0.00078
p__Proteobacteria	g__Corallincola	0.001399	0.0002793	0.0007091	0.000123	0.000183	0.001654	-0.00087	-0.00051	-0.00069
p__Proteobacteria	g__Luminiphilus	0.001948	0.0004185	0.00127	0.000435	0.005795	0.01483	-0.001	-0.00032	-0.00068
p__Proteobacteria	g__Paraglaciecola	0.004319	0.0007342	0.003649	0.000963	0.02575	0.05065	-0.00135	0.000163	-0.00067
p__Proteobacteria	g__Methylosarcina	0.004797	0.0004004	0.00413	0.000741	0.02113	0.04273	-0.00113	-0.0002	-0.00067
p__Proteobacteria	g__Limimonas	0.001915	0.0002722	0.001251	0.000408	0.003611	0.01003	-0.00094	-0.00033	-0.00066
p__Proteobacteria	g__Lutibaculum	0.003647	0.0003424	0.002995	0.000503	0.004586	0.01226	-0.00103	-0.00033	-0.00065
p__Proteobacteria	g__Desulfotignum	0.001941	0.000585	0.001362	0.00036	0.01133	0.02551	-0.00099	-0.00018	-0.00058
p__Proteobacteria	g__Candidatus_Marithrix	0.001309	0.0004201	0.0007351	0.000216	0.001706	0.00577	-0.00086	-0.00029	-0.00057
p__Proteobacteria	g__Rickettsia	0.001279	0.000275	0.0007154	0.000128	0.00044	0.002332	-0.00075	-0.00039	-0.00056
p__Proteobacteria	g__Tepidicella	0.001416	0.0003019	0.0008602	0.000131	0.000183	0.001654	-0.00076	-0.00039	-0.00056
p__Proteobacteria	g__Hankyongella	0.001338	0.0002774	0.0007895	0.000173	0.00044	0.002332	-0.00075	-0.00035	-0.00055
p__Proteobacteria	g__Mongoliimonas	0.00474	0.0006786	0.0042	0.000367	0.04515	0.08009	-0.00098	-0.00012	-0.00054
p__Proteobacteria	g__Planktomarina	0.0008089	0.0003634	0.0002733	0.000121	0.000246	0.001813	-0.00076	-0.00033	-0.00054
p__Proteobacteria	g__Glaciimonas	0.002794	0.0006031	0.002263	0.00033	0.03764	0.06884	-0.00093	-0.00013	-0.00053
p__Proteobacteria	g__unclassified_f__Salinarimonadaceae	0.003009	0.0003653	0.002502	0.000355	0.004586	0.01226	-0.00082	-0.00021	-0.00051
p__Proteobacteria	g__unclassified_o__Legionellales	0.001135	0.0002957	0.0006303	0.00024	0.001008	0.003952	-0.00074	-0.00027	-0.0005
p__Proteobacteria	g__Palleronia	0.0009341	0.0001925	0.0004488	7.54E-05	0.000183	0.001654	-0.0006	-0.00037	-0.00049
p__Proteobacteria	g__Pelagibaca	0.0007533	0.0002044	0.0002796	0.000104	0.00044	0.002332	-0.0006	-0.00034	-0.00047
p__Proteobacteria	g__Anderseniella	0.00229	0.0005124	0.001817	0.000372	0.04515	0.08009	-0.00083	-6.92E-05	-0.00047
p__Proteobacteria	g__Desulfospira	0.0008539	0.0006495	0.0003843	0.000129	0.03764	0.06884	-0.00088	-0.00013	-0.00047
p__Proteobacteria	g__Pseudomethylobacillus	0.001625	0.0002785	0.001158	0.000258	0.003611	0.01003	-0.00069	-0.00024	-0.00047
p__Proteobacteria	g__Oceanimonas	0.001164	0.0004023	0.0007197	0.000208	0.007285	0.01777	-0.00073	-0.0002	-0.00044
p__Proteobacteria	g__Roseococcus	0.00232	0.0004576	0.001877	0.000323	0.01726	0.03608	-0.0008	-0.00012	-0.00044
p__Proteobacteria	g__Mangrovicoccus	0.001539	0.0003075	0.001104	0.000271	0.004586	0.01226	-0.00068	-0.0002	-0.00044
p__Proteobacteria	g__Marivita	0.001621	0.0002795	0.001192	0.000128	0.000583	0.002674	-0.0006	-0.00024	-0.00043
p__Proteobacteria	g__unclassified_f__Idiomarinaceae	0.0005935	0.0002518	0.000198	8.10E-05	0.000183	0.001654	-0.00057	-0.00027	-0.0004
p__Proteobacteria	g__Celeribacter	0.002063	0.0003168	0.001681	0.000185	0.01402	0.03047	-0.0006	-0.00015	-0.00038
p__Proteobacteria	g__Geopsychrobacter	0.0007502	0.0003344	0.0003737	0.00017	0.005795	0.01483	-0.00061	-0.00017	-0.00038
p__Proteobacteria	g__unclassified_f__Rickettsiaceae	0.000398	0.0003251	2.94E-05	1.92E-05	0.000183	0.001654	-0.00058	-0.0002	-0.00037
p__Proteobacteria	g__Melaminivora	0.001133	0.0003108	0.0007777	0.000322	0.02113	0.04273	-0.00061	-6.77E-05	-0.00036
p__Proteobacteria	g__Pelagicola	0.0009261	0.0003635	0.0005735	0.000135	0.005795	0.01483	-0.00058	-0.00013	-0.00035
p__Proteobacteria	g__Maritimibacter	0.003915	0.0002314	0.003566	0.000265	0.01133	0.02551	-0.00055	-0.00012	-0.00035
p__Proteobacteria	g__unclassified_o__Enterobacterales	0.0008177	0.0001666	0.0004746	0.000123	0.000583	0.002674	-0.00046	-0.00022	-0.00034
p__Proteobacteria	g__Salinicola	0.001966	0.0003392	0.001623	0.000426	0.03764	0.06884	-0.00065	-2.34E-05	-0.00034
p__Proteobacteria	g__Candidatus_Magnetaquicoccus	0.0005895	0.0004083	0.0002559	9.31E-05	0.01402	0.03047	-0.00059	-0.00011	-0.00033
p__Proteobacteria	g__Amphritea	0.0008549	0.0001642	0.0005222	7.33E-05	0.000246	0.001813	-0.00045	-0.00023	-0.00033
p__Proteobacteria	g__Serpentinomonas	0.0007988	0.0002155	0.0004855	0.000184	0.009108	0.0215	-0.00048	-0.00014	-0.00031
p__Proteobacteria	g__Limimaricola	0.001245	0.0003771	0.000936	0.000128	0.03121	0.05963	-0.00056	-8.95E-05	-0.00031
p__Proteobacteria	g__Rhodophyticola	0.0005556	0.0001492	0.0002629	7.04E-05	0.00044	0.002332	-0.00039	-0.00019	-0.00029
p__Proteobacteria	g__Haematospirillum	0.001004	0.0001741	0.0007385	0.000119	0.001315	0.004744	-0.00039	-0.00014	-0.00027
p__Proteobacteria	g__Hydrogenovibrio	0.0005471	0.0002345	0.0002932	0.000124	0.01726	0.03608	-0.00041	-8.67E-05	-0.00025
p__Proteobacteria	g__Fastidiosibacter	0.0003873	8.95E-05	0.0001527	5.31E-05	0.000183	0.001654	-0.00029	-0.00017	-0.00023
p__Proteobacteria	g__Neomegalonema	0.0006007	0.0001864	0.0003697	0.000109	0.002202	0.00693	-0.00036	-0.00011	-0.00023
p__Proteobacteria	g__Candidatus_Fonsibacter	0.0006632	0.0002109	0.0004399	9.01E-05	0.01402	0.03047	-0.00036	-9.05E-05	-0.00022
p__Proteobacteria	g__Loktanella	0.001244	0.000203	0.001021	0.000182	0.03121	0.05963	-0.00039	-4.78E-05	-0.00022
p__Proteobacteria	g__Monaibacterium	0.0004929	5.65E-05	0.0002708	0.000134	0.002827	0.00826	-0.0003	-0.00013	-0.00022
p__Proteobacteria	g__Dichotomicrobium	0.001558	0.000398	0.001338	0.000148	0.04515	0.08009	-0.00047	2.07E-05	-0.00022

p__Proteobacteria	g__Parashewanella	0.0004006	0.0002086	0.0001812	8.53E-05	0.004586	0.01226	-0.00038	-9.93E-05	-0.00022
p__Proteobacteria	g__Conchiformibius	0.0002484	0.0002882	3.78E-05	1.96E-05	0.03764	0.06884	-0.0004	-6.57E-05	-0.00021
p__Proteobacteria	g__Simulacricoccus	0.0002592	0.0001079	5.73E-05	2.86E-05	0.000183	0.001654	-0.00026	-0.00014	-0.0002
p__Proteobacteria	g__Zymomonas	0.0004369	0.0001541	0.0002386	7.17E-05	0.001706	0.00577	-0.0003	-0.00011	-0.0002
p__Proteobacteria	g__Guyparkeria	0.0003916	9.45E-05	0.0002109	0.000109	0.003611	0.01003	-0.00026	-9.20E-05	-0.00018
p__Proteobacteria	g__Desulfovermiculus	0.0003443	0.0001106	0.0001672	5.19E-05	0.000583	0.002674	-0.00025	-0.00011	-0.00018
p__Proteobacteria	g__Pelagivirga	0.000277	9.62E-05	0.0001026	2.68E-05	0.000183	0.001654	-0.00024	-0.00012	-0.00017
p__Proteobacteria	g__Sulfurovum	0.0006296	0.0001503	0.0004611	0.000107	0.01726	0.03608	-0.00027	-5.74E-05	-0.00017
p__Proteobacteria	g__Saezia	0.0005214	0.0001135	0.0003573	0.000114	0.004586	0.01226	-0.00025	-6.73E-05	-0.00016
p__Proteobacteria	g__Rouxiella	0.0001961	0.0001194	3.51E-05	1.61E-05	0.000183	0.001654	-0.00024	-9.39E-05	-0.00016
p__Proteobacteria	g__Wolbachia	0.0002702	0.0001057	0.0001152	3.89E-05	0.001706	0.00577	-0.00022	-8.55E-05	-0.00016
p__Proteobacteria	g__Ciceribacter	0.0007356	0.0001353	0.0005901	9.57E-05	0.03121	0.05963	-0.00025	-5.48E-05	-0.00015
p__Proteobacteria	g__Candidatus_Methylopumilus	0.0002257	0.000133	8.31E-05	3.72E-05	0.000769	0.003257	-0.00023	-7.34E-05	-0.00014
p__Proteobacteria	g__unclassified_f__Brucellaceae	0.0002158	8.55E-05	8.79E-05	3.10E-05	0.002202	0.00693	-0.00018	-7.45E-05	-0.00013
p__Proteobacteria	g__Reinekea	0.0003389	0.0001448	0.0002144	8.62E-05	0.01726	0.03608	-0.00022	-2.50E-05	-0.00012
p__Proteobacteria	g__Sediminimonas	0.0002211	0.0001499	0.0001127	4.06E-05	0.02575	0.05065	-0.00021	-2.90E-05	-0.00011
p__Proteobacteria	g__Fluoribacter	0.0003295	9.78E-05	0.0002233	6.18E-05	0.02575	0.05065	-0.00017	-3.38E-05	-0.00011
p__Proteobacteria	g__Pseudoroseicyclus	0.0005457	0.0001068	0.0004399	6.02E-05	0.03121	0.05963	-0.00017	-2.96E-05	-0.00011
p__Proteobacteria	g__Cognatishimia	0.0001801	9.87E-05	8.86E-05	2.80E-05	0.03764	0.06884	-0.00015	-2.78E-05	-9.15E-05
p__Proteobacteria	g__Aliishimia	0.0001507	8.65E-05	6.72E-05	2.53E-05	0.002202	0.00693	-0.00014	-3.86E-05	-8.36E-05
p__Proteobacteria	g__Donghicola	0.0002922	7.47E-05	0.0002097	5.41E-05	0.01726	0.03608	-0.00014	-3.17E-05	-8.25E-05
p__Proteobacteria	g__Rickettsiella	0.0001088	9.52E-05	2.76E-05	2.79E-05	0.002827	0.00826	-0.00015	-3.28E-05	-8.12E-05
p__Proteobacteria	g__Thiovulum	0.0001093	7.95E-05	2.98E-05	2.17E-05	0.000583	0.002674	-0.00014	-3.90E-05	-7.95E-05
p__Proteobacteria	g__Aestuariivita	0.0001769	2.99E-05	0.0001	3.11E-05	0.00044	0.002332	-0.0001	-5.25E-05	-7.69E-05
p__Proteobacteria	g__Pulveribacter	0.0001445	5.39E-05	6.92E-05	2.63E-05	0.002202	0.00693	-0.00011	-4.05E-05	-7.53E-05
p__Proteobacteria	g__Thiofilum	0.0002092	5.65E-05	0.0001354	6.63E-05	0.01133	0.02551	-0.00012	-2.17E-05	-7.39E-05
p__Proteobacteria	g__Pajaroellobacter	0.0001284	5.67E-05	5.66E-05	3.86E-05	0.009108	0.0215	-0.00011	-3.27E-05	-7.18E-05
p__Proteobacteria	g__Desulfoplanes	0.0002846	6.65E-05	0.0002161	6.22E-05	0.03121	0.05963	-0.00012	-1.54E-05	-6.85E-05
p__Proteobacteria	g__Thalassobium	0.0001653	6.32E-05	9.93E-05	3.88E-05	0.01133	0.02551	-0.00011	-2.54E-05	-6.60E-05
p__Proteobacteria	g__Parasutterella	0.0001726	4.65E-05	0.0001108	6.48E-05	0.009108	0.0215	-0.00011	-1.27E-05	-6.19E-05
p__Proteobacteria	g__Nissabacter	7.74E-05	3.61E-05	2.01E-05	1.41E-05	0.00044	0.002332	-8.10E-05	-3.52E-05	-5.73E-05
p__Proteobacteria	g__Aggregatibacter	0.0001032	2.82E-05	4.60E-05	2.87E-05	0.002202	0.00693	-8.03E-05	-3.16E-05	-5.72E-05
p__Proteobacteria	g__Frischella	8.93E-05	4.25E-05	3.76E-05	3.21E-05	0.01133	0.02551	-8.49E-05	-2.05E-05	-5.17E-05
p__Proteobacteria	g__Rodentibacter	0.0001102	3.38E-05	5.91E-05	3.04E-05	0.005795	0.01483	-7.62E-05	-2.28E-05	-5.10E-05
p__Proteobacteria	g__Atlantibacter	8.48E-05	3.33E-05	3.43E-05	1.52E-05	0.001008	0.003952	-7.17E-05	-2.88E-05	-5.05E-05
p__Proteobacteria	g__Ignatzschineria	7.05E-05	5.72E-05	2.36E-05	3.26E-05	0.02569	0.05065	-8.76E-05	-1.09E-05	-4.69E-05
p__Proteobacteria	g__Bibersteinia	4.24E-05	3.98E-05	4.99E-07	1.58E-06	0.000121	0.001654	-6.72E-05	-1.86E-05	-4.19E-05
p__Proteobacteria	g__Ventosimonas	5.72E-05	2.73E-05	2.10E-05	1.48E-05	0.001706	0.00577	-5.45E-05	-1.91E-05	-3.62E-05
p__Proteobacteria	g__Duodenibacillus	4.00E-05	4.27E-05	4.97E-06	6.95E-06	0.006852	0.0174	-6.21E-05	-1.18E-05	-3.50E-05
p__Proteobacteria	g__Saccharibacter	4.72E-05	3.52E-05	1.55E-05	1.25E-05	0.02109	0.04273	-5.46E-05	-1.04E-05	-3.17E-05
p__Proteobacteria	g__Fangia	4.31E-05	2.71E-05	1.29E-05	1.07E-05	0.004571	0.01226	-4.79E-05	-1.29E-05	-3.01E-05
p__Proteobacteria	g__Sulfurivirga	4.54E-05	2.81E-05	1.57E-05	1.18E-05	0.005795	0.01483	-4.78E-05	-1.19E-05	-2.97E-05
p__Proteobacteria	g__Candidatus_Finniella	7.80E-05	2.94E-05	5.14E-05	2.64E-05	0.03121	0.05963	-5.01E-05	-3.84E-06	-2.66E-05
p__Proteobacteria	g__Gibbsiella	4.66E-05	1.80E-05	2.26E-05	1.43E-05	0.007285	0.01777	-3.73E-05	-9.65E-06	-2.40E-05
p__Proteobacteria	g__Pseudospirillum	3.08E-05	1.45E-05	7.73E-06	9.66E-06	0.002119	0.00693	-3.34E-05	-1.27E-05	-2.31E-05
p__Proteobacteria	g__Siccibacter	1.98E-05	1.81E-05	5.77E-06	6.93E-06	0.02464	0.04965	-2.62E-05	-3.13E-06	-1.40E-05
p__Proteobacteria	g__Necropsobacter	1.24E-05	1.82E-05	0	0	0.002213	0.00693	-2.46E-05	-3.86E-06	-1.24E-05
p__Proteobacteria	g__Thorsellia	1.13E-05	1.81E-05	0	0	0.002213	0.00693	-2.45E-05	-3.37E-06	-1.13E-05

p__Proteobacteria	g__Shimwellia	1.35E-05	1.12E-05	2.23E-06	3.09E-06	0.01114	0.02551	-1.82E-05	-4.50E-06	-1.12E-05
p__Proteobacteria	g__Candidatus_Aquarickettsia	9.08E-06	1.09E-05	0	0	0.01493	0.03223	-1.55E-05	-2.87E-06	-9.08E-06
p__Proteobacteria	g__Phocoenobacter	7.32E-06	1.18E-05	0	0	0.01493	0.03223	-1.50E-05	-1.73E-06	-7.32E-06
p__Proteobacteria	g__Ehrlichia	7.91E-06	7.70E-06	1.40E-06	3.09E-06	0.04496	0.08009	-1.14E-05	-1.55E-06	-6.51E-06
p__Proteobacteria	g__Vespertiliibacter	5.84E-06	1.22E-05	0	0	0.03498	0.06617	-1.44E-05	-4.35E-07	-5.84E-06
p__Proteobacteria	g__unclassified_f__Erwiniaceae	5.72E-06	6.98E-06	5.15E-07	1.63E-06	0.01875	0.03907	-9.67E-06	-1.15E-06	-5.21E-06
p__Planctomycetes	g__unclassified_f__Lacipirellulaceae	0.2902	0.04607	0.1955	0.06177	0.002827	0.00826	-0.1397	-0.04703	-0.09469
p__Planctomycetes	g__Lacipirellula	0.1994	0.03116	0.1395	0.04322	0.005795	0.01483	-0.08982	-0.02482	-0.05995
p__Planctomycetes	g__unclassified_c__Planctomycetia	0.1492	0.02575	0.101	0.02042	0.001008	0.003952	-0.06703	-0.02643	-0.04823
p__Planctomycetes	g__unclassified_o__Planctomycetales	0.1644	0.02524	0.1337	0.03036	0.04515	0.08009	-0.05395	-0.00736	-0.03082
p__Planctomycetes	g__Crateriforma	0.00132	0.0003664	0.0007726	0.00014	0.000583	0.002674	-0.00079	-0.00033	-0.00055
p__Bacteroidetes	g__unclassified_f__Flavobacteriaceae	0.08324	0.06161	0.02425	0.009474	0.002827	0.00826	-0.09769	-0.02511	-0.05899
p__Bacteroidetes	g__unclassified_f__Cyclobacteriaceae	0.0865	0.04685	0.04412	0.01273	0.01402	0.03047	-0.07325	-0.01524	-0.04239
p__Bacteroidetes	g__unclassified_o__Cytophagales	0.05933	0.03377	0.03473	0.009065	0.04515	0.08009	-0.04555	-0.00445	-0.02459
p__Bacteroidetes	g__Chryseotalea	0.03056	0.01836	0.01332	0.004951	0.003611	0.01003	-0.02899	-0.00734	-0.01723
p__Bacteroidetes	g__Pseudoflavitalea	0.01878	0.01172	0.005936	0.004419	0.001706	0.00577	-0.02033	-0.00623	-0.01285
p__Bacteroidetes	g__Paraflavitalea	0.01522	0.01028	0.003199	0.002483	0.00044	0.002332	-0.01875	-0.00684	-0.01203
p__Bacteroidetes	g__Fulvivirga	0.02423	0.007891	0.01283	0.002281	0.000246	0.001813	-0.01611	-0.00693	-0.0114
p__Bacteroidetes	g__Muricauda	0.01571	0.01034	0.004824	0.001583	0.000583	0.002674	-0.01778	-0.00488	-0.01088
p__Bacteroidetes	g__unclassified_c__Cytophagia	0.02145	0.01397	0.01058	0.00288	0.01726	0.03608	-0.02006	-0.0034	-0.01088
p__Bacteroidetes	g__Maribacter	0.007204	0.00493	0.002137	0.000847	0.001315	0.004744	-0.00825	-0.00247	-0.00507
p__Bacteroidetes	g__Arenibacter	0.005673	0.003859	0.002124	0.000807	0.007285	0.01777	-0.00596	-0.00147	-0.00355
p__Bacteroidetes	g__Zeaxanthinibacter	0.002198	0.00167	0.0002718	0.000258	0.000769	0.003257	-0.00294	-0.00097	-0.00193
p__Bacteroidetes	g__Ekhidna	0.002269	0.000419	0.0005989	0.000184	0.000183	0.001654	-0.00193	-0.0014	-0.00167
p__Bacteroidetes	g__Eudoraea	0.002173	0.001369	0.000537	0.000351	0.000769	0.003257	-0.0025	-0.00084	-0.00164
p__Bacteroidetes	g__Robiginitalea	0.001704	0.001333	0.0002317	0.000239	0.000583	0.002674	-0.00227	-0.00073	-0.00147
p__Bacteroidetes	g__Rufibacter	0.004629	0.001084	0.003204	0.000549	0.002202	0.00693	-0.00225	-0.00077	-0.00143
p__Bacteroidetes	g__Paludibacter	0.002437	0.000779	0.00114	0.000292	0.000246	0.001813	-0.00181	-0.00083	-0.0013
p__Bacteroidetes	g__Catalinimonas	0.004984	0.0004753	0.003819	0.00053	0.00044	0.002332	-0.00155	-0.00075	-0.00117
p__Bacteroidetes	g__Kriegella	0.001524	0.0009031	0.0003998	0.00014	0.00033	0.002032	-0.00171	-0.00062	-0.00112
p__Bacteroidetes	g__Muriicola	0.001437	0.0008162	0.0003286	0.000128	0.000183	0.001654	-0.00167	-0.00067	-0.00111
p__Bacteroidetes	g__Pseudobacter	0.001705	0.0007355	0.0008432	0.000421	0.002202	0.00693	-0.00141	-0.00038	-0.00086
p__Bacteroidetes	g__Zobellia	0.001098	0.0008892	0.0002564	0.000141	0.002202	0.00693	-0.00144	-0.00037	-0.00084
p__Bacteroidetes	g__Sinomicrobium	0.001373	0.0007266	0.0005967	0.000173	0.005795	0.01483	-0.00126	-0.00037	-0.00078
p__Bacteroidetes	g__Proteiniphilum	0.0009726	0.000503	0.0002532	8.88E-05	0.000183	0.001654	-0.00103	-0.00043	-0.00072
p__Bacteroidetes	g__Aquiflexum	0.001826	0.0007388	0.001189	0.000326	0.03121	0.05963	-0.00113	-0.00019	-0.00064
p__Bacteroidetes	g__Microscilla	0.001291	0.001341	0.0006758	0.000157	0.03121	0.05963	-0.00153	-3.28E-05	-0.00062
p__Bacteroidetes	g__Gramella	0.001579	0.0006829	0.0009758	0.000267	0.01726	0.03608	-0.00107	-0.00022	-0.0006
p__Bacteroidetes	g__Algibacter	0.001061	0.0003594	0.0006088	0.000189	0.004586	0.01226	-0.00071	-0.00021	-0.00045
p__Bacteroidetes	g__Saonia	0.0007133	0.0005106	0.0002748	0.000148	0.01402	0.03047	-0.00077	-0.00014	-0.00044
p__Bacteroidetes	g__Rhodonellum	0.0007769	0.0003809	0.000385	0.000131	0.009108	0.0215	-0.00065	-0.00016	-0.00039
p__Bacteroidetes	g__Mariniradius	0.0008467	0.0004069	0.0004764	0.000196	0.01726	0.03608	-0.00064	-0.00011	-0.00037
p__Bacteroidetes	g__Sediminicola	0.0005031	0.0003777	0.0001889	7.21E-05	0.04515	0.08009	-0.00054	-0.0001	-0.00031
p__Bacteroidetes	g__Robertkochia	0.0004775	0.0003372	0.0002008	0.000135	0.02575	0.05065	-0.00051	-7.02E-05	-0.00028
p__Bacteroidetes	g__Arenitalea	0.000357	0.000141	8.20E-05	4.76E-05	0.00044	0.002332	-0.00036	-0.00018	-0.00028
p__Bacteroidetes	g__Acetobacteroides	0.0002661	0.0002215	3.26E-05	2.81E-05	0.000583	0.002674	-0.00036	-0.00011	-0.00023
p__Bacteroidetes	g__Salinivirga	0.0003718	0.0001176	0.0001539	8.37E-05	0.000769	0.003257	-0.0003	-0.00013	-0.00022
p__Bacteroidetes	g__Euzebyella	0.0002843	0.0002396	9.84E-05	4.28E-05	0.03764	0.06884	-0.00034	-5.50E-05	-0.00019

p__Bacteroidetes	g__Tangfeifania	0.0002424	0.0001835	6.48E-05	4.30E-05	0.01402	0.03047	-0.0003	-7.73E-05	-0.00018
p__Bacteroidetes	g__Nibribacter	0.0002165	0.0001436	8.74E-05	5.65E-05	0.01726	0.03608	-0.00023	-4.09E-05	-0.00013
p__Bacteroidetes	g__Joostella	0.0001752	0.0001417	4.88E-05	3.97E-05	0.01133	0.02551	-0.00021	-3.93E-05	-0.00013
p__Bacteroidetes	g__Croceibacter	0.0001472	9.02E-05	4.36E-05	1.93E-05	0.001706	0.00577	-0.00016	-5.19E-05	-0.0001
p__Bacteroidetes	g__Wenyingzhuangia	0.0001622	0.0001071	6.69E-05	2.59E-05	0.01402	0.03047	-0.00017	-3.70E-05	-9.54E-05
p__Bacteroidetes	g__Schleiferia	0.0001286	9.69E-05	4.12E-05	3.35E-05	0.01726	0.03608	-0.00015	-2.85E-05	-8.74E-05
p__Bacteroidetes	g__Zunongwangia	0.0001764	0.0001007	9.02E-05	4.35E-05	0.03764	0.06884	-0.00015	-2.55E-05	-8.62E-05
p__Bacteroidetes	g__Imtechella	0.0001041	8.01E-05	1.93E-05	1.83E-05	0.001008	0.003952	-0.00014	-4.35E-05	-8.48E-05
p__Bacteroidetes	g__Litoribacter	0.0002089	5.12E-05	0.0001294	6.51E-05	0.01133	0.02551	-0.00012	-3.18E-05	-7.95E-05
p__Bacteroidetes	g__Urechidicola	0.0001362	7.10E-05	5.72E-05	3.56E-05	0.005795	0.01483	-0.00012	-3.27E-05	-7.90E-05
p__Bacteroidetes	g__unclassified_f__Marinifilaceae	0.0002382	7.87E-05	0.00016	3.89E-05	0.02575	0.05065	-0.00013	-2.81E-05	-7.83E-05
p__Bacteroidetes	g__Hyunsoonleella	0.0001185	3.81E-05	4.37E-05	1.95E-05	0.00044	0.002332	-0.0001	-4.77E-05	-7.48E-05
p__Bacteroidetes	g__Galbibacter	0.0001066	7.11E-05	3.35E-05	3.78E-05	0.009082	0.0215	-0.00012	-2.83E-05	-7.31E-05
p__Bacteroidetes	g__Algoriella	9.52E-05	6.34E-05	2.75E-05	1.69E-05	0.01133	0.02551	-0.00011	-2.72E-05	-6.77E-05
p__Bacteroidetes	g__Williamwhitmania	0.0001666	5.00E-05	9.93E-05	5.49E-05	0.02113	0.04273	-0.00011	-2.46E-05	-6.73E-05
p__Bacteroidetes	g__Flavimarina	8.67E-05	7.54E-05	2.40E-05	1.49E-05	0.03121	0.05963	-0.00011	-1.80E-05	-6.28E-05
p__Bacteroidetes	g__Meridianimaribacter	8.01E-05	8.28E-05	2.39E-05	1.99E-05	0.03121	0.05963	-0.00011	-1.29E-05	-5.62E-05
p__Bacteroidetes	g__Alkaliflexus	6.78E-05	3.91E-05	1.24E-05	1.23E-05	0.000765	0.003257	-8.05E-05	-3.12E-05	-5.55E-05
p__Bacteroidetes	g__Subsaximicrobium	7.00E-05	2.41E-05	1.58E-05	1.37E-05	0.000328	0.002032	-7.06E-05	-3.70E-05	-5.43E-05
p__Bacteroidetes	g__Oceanihabitans	0.0001007	4.58E-05	4.78E-05	3.13E-05	0.001706	0.00577	-8.62E-05	-1.94E-05	-5.29E-05
p__Bacteroidetes	g__Paraprevotella	0.0001176	3.33E-05	7.51E-05	3.55E-05	0.02575	0.05065	-7.25E-05	-1.55E-05	-4.26E-05
p__Bacteroidetes	g__Allopseudarcicella	6.41E-05	2.01E-05	2.59E-05	9.16E-06	0.000769	0.003257	-5.13E-05	-2.50E-05	-3.82E-05
p__Bacteroidetes	g__Microbacter	3.75E-05	2.73E-05	0	0	6.39E-05	0.001654	-5.52E-05	-2.24E-05	-3.75E-05
p__Bacteroidetes	g__Candidatus_Cardinium	5.92E-05	3.33E-05	2.36E-05	1.66E-05	0.02575	0.05065	-5.53E-05	-1.18E-05	-3.56E-05
p__Bacteroidetes	g__Aureicoccus	3.92E-05	3.08E-05	1.12E-05	1.25E-05	0.03362	0.0642	-4.72E-05	-7.78E-06	-2.80E-05
p__Bacteroidetes	g__Nitritalea	4.14E-05	2.17E-05	1.63E-05	1.08E-05	0.01133	0.02551	-3.92E-05	-1.10E-05	-2.51E-05
p__Bacteroidetes	g__Coprobacter	1.16E-05	1.46E-05	3.99E-06	1.26E-05	0.02124	0.0429	-1.86E-05	4.19E-06	-7.62E-06
p__Bacteroidetes	g__unclassified_f__Odoribacteraceae	2.09E-06	2.73E-06	0	0	0.03498	0.06617	-3.80E-06	-5.29E-07	-2.09E-06
p__Acidobacteria	g__Holophaga	0.001563	0.0003598	0.000907	0.000208	0.000246	0.001813	-0.00091	-0.00042	-0.00066
p__Actinobacteria	g__Pseudonocardia	1.164	0.4846	0.1025	0.03923	0.000183	0.001654	-1.343	-0.7978	-1.062
p__Actinobacteria	g__Alloactinosynnema	0.2313	0.2283	0.002429	0.000767	0.000183	0.001654	-0.3732	-0.1058	-0.2289
p__Actinobacteria	g__Desertimonas	0.4099	0.09351	0.2436	0.1	0.004586	0.01226	-0.2435	-0.07748	-0.1662
p__Actinobacteria	g__Actinokineospora	0.1604	0.1571	0.004029	0.001888	0.000183	0.001654	-0.254	-0.0693	-0.1564
p__Actinobacteria	g__unclassified_o__Pseudonocardiales	0.1279	0.1212	0.008973	0.0038	0.000183	0.001654	-0.1977	-0.05959	-0.119
p__Actinobacteria	g__Ilumatobacter	0.1428	0.04044	0.04659	0.01719	0.000183	0.001654	-0.1229	-0.07136	-0.09621
p__Actinobacteria	g__Streptomyces	0.1855	0.06204	0.09897	0.0113	0.000183	0.001654	-0.1287	-0.05247	-0.08651
p__Actinobacteria	g__unclassified_c__Actinobacteria	0.2663	0.04055	0.1844	0.03904	0.001315	0.004744	-0.1163	-0.05107	-0.08189
p__Actinobacteria	g__Amycolatopsis	0.09831	0.05166	0.01941	0.003715	0.000183	0.001654	-0.1111	-0.04923	-0.07889
p__Actinobacteria	g__Agromyces	0.07906	0.1094	0.005749	0.002272	0.000183	0.001654	-0.142	-0.01408	-0.07331
p__Actinobacteria	g__unclassified_c__Acidimicrobiia	0.09661	0.03599	0.03242	0.007894	0.000183	0.001654	-0.08539	-0.04376	-0.0642
p__Actinobacteria	g__Saccharothrix	0.06424	0.05035	0.006228	0.001016	0.000183	0.001654	-0.09054	-0.02961	-0.05802
p__Actinobacteria	g__unclassified_f__Pseudonocardiaceae	0.06577	0.04912	0.00935	0.001851	0.000183	0.001654	-0.08877	-0.03112	-0.05642
p__Actinobacteria	g__unclassified_f__Acidimicrobiaceae	0.09608	0.02163	0.0414	0.01268	0.000183	0.001654	-0.06999	-0.0392	-0.05467
p__Actinobacteria	g__Actinophytocola	0.04447	0.03086	0.004314	0.000745	0.000183	0.001654	-0.05899	-0.02166	-0.04016
p__Actinobacteria	g__Micromonospora	0.06318	0.01945	0.03078	0.003492	0.000183	0.001654	-0.04462	-0.0211	-0.0324
p__Actinobacteria	g__Sporichthya	0.04883	0.01691	0.01986	0.005947	0.000583	0.002674	-0.03887	-0.01826	-0.02897
p__Actinobacteria	g__Kribbella	0.03778	0.01168	0.01234	0.001852	0.000183	0.001654	-0.03205	-0.0184	-0.02544
p__Actinobacteria	g__Nocardia	0.03871	0.01139	0.01554	0.001844	0.000183	0.001654	-0.03048	-0.01627	-0.02318

p__Actinobacteria	g__unclassified_o__Acidimicrobiales	0.04119	0.007985	0.02147	0.005306	0.000183	0.001654	-0.02511	-0.01432	-0.01973
p__Actinobacteria	g__Frankia	0.03516	0.01151	0.01676	0.003875	0.000183	0.001654	-0.02569	-0.01163	-0.0184
p__Actinobacteria	g__Actinomadura	0.04206	0.009943	0.02543	0.003863	0.00033	0.002032	-0.02327	-0.01111	-0.01664
p__Actinobacteria	g__Lentzea	0.01958	0.0131	0.002996	0.001051	0.000246	0.001813	-0.02373	-0.00926	-0.01659
p__Actinobacteria	g__Rhodococcus_f__Nocardiaceae	0.03322	0.008049	0.01758	0.003138	0.000183	0.001654	-0.02082	-0.01039	-0.01564
p__Actinobacteria	g__Mycobacterium	0.07486	0.01138	0.05963	0.01036	0.01133	0.02551	-0.02466	-0.00628	-0.01523
p__Actinobacteria	g__Prauserella	0.01669	0.00879	0.002073	0.000505	0.000183	0.001654	-0.0202	-0.00969	-0.01462
p__Actinobacteria	g__Nocardioides	0.03452	0.005765	0.02046	0.004064	0.000183	0.001654	-0.0184	-0.01011	-0.01406
p__Actinobacteria	g__Geodermatophilus	0.02096	0.006042	0.007404	0.001179	0.000183	0.001654	-0.01719	-0.01	-0.01355
p__Actinobacteria	g__Lechevalieria	0.01282	0.009579	0.001333	0.000272	0.000183	0.001654	-0.01745	-0.00581	-0.01149
p__Actinobacteria	g__Kibdelosporangium	0.01167	0.007334	0.001824	0.000368	0.000183	0.001654	-0.01461	-0.00589	-0.00984
p__Actinobacteria	g__Kutzneria	0.011	0.0062	0.00124	0.000312	0.000183	0.001654	-0.0137	-0.00655	-0.00976
p__Actinobacteria	g__Nonomuraea	0.02376	0.005197	0.01408	0.001525	0.000183	0.001654	-0.01294	-0.00664	-0.00968
p__Actinobacteria	g__Blastococcus	0.01719	0.004006	0.007536	0.001756	0.000183	0.001654	-0.01224	-0.00704	-0.00965
p__Actinobacteria	g__Saccharopolyspora	0.01324	0.007172	0.00364	0.0006	0.000183	0.001654	-0.01434	-0.00595	-0.0096
p__Actinobacteria	g__Jiangella	0.02216	0.006576	0.01338	0.001672	0.00044	0.002332	-0.01302	-0.00496	-0.00878
p__Actinobacteria	g__Actinomycetospora	0.009574	0.003281	0.002088	0.000662	0.000183	0.001654	-0.00935	-0.00542	-0.00749
p__Actinobacteria	g__Mycolicibacterium	0.03613	0.008677	0.02885	0.004086	0.02113	0.04273	-0.01358	-0.00235	-0.00728
p__Actinobacteria	g__unclassified_p__Actinobacteria	0.0193	0.008054	0.01272	0.001842	0.002202	0.00693	-0.01228	-0.00262	-0.00658
p__Actinobacteria	g__Thermocrispum	0.007053	0.00553	0.0004752	0.000131	0.000183	0.001654	-0.01054	-0.00387	-0.00658
p__Actinobacteria	g__Saccharomonospora	0.007813	0.003949	0.001255	0.000247	0.000183	0.001654	-0.00905	-0.00448	-0.00656
p__Actinobacteria	g__Cellulomonas	0.01296	0.00289	0.00706	0.001497	0.000246	0.001813	-0.00784	-0.00394	-0.0059
p__Actinobacteria	g__Labedaea	0.006102	0.004337	0.0003587	0.000185	0.000183	0.001654	-0.00822	-0.00323	-0.00574
p__Actinobacteria	g__Herbihabitans	0.006385	0.004003	0.0007467	0.000211	0.000183	0.001654	-0.00806	-0.00339	-0.00564
p__Actinobacteria	g__Streptoalloteichus	0.00587	0.004489	0.0004089	0.000174	0.000183	0.001654	-0.00856	-0.00328	-0.00546
p__Actinobacteria	g__Modestobacter	0.008463	0.002543	0.003079	0.000675	0.000183	0.001654	-0.00688	-0.00383	-0.00538
p__Actinobacteria	g__Actinoplanes	0.01575	0.00446	0.01051	0.001586	0.004586	0.01226	-0.0083	-0.00254	-0.00524
p__Actinobacteria	g__Umezawaea	0.005552	0.004324	0.0004214	7.01E-05	0.000183	0.001654	-0.00784	-0.00275	-0.00513
p__Actinobacteria	g__Allokutzneria	0.005466	0.002965	0.0005421	0.000186	0.000183	0.001654	-0.00675	-0.00345	-0.00492
p__Actinobacteria	g__Actinosynnema	0.005397	0.003863	0.0005514	0.000131	0.000183	0.001654	-0.00727	-0.00265	-0.00485
p__Actinobacteria	g__Streptosporangium	0.00899	0.003801	0.004174	0.000589	0.000246	0.001813	-0.00736	-0.00271	-0.00482
p__Actinobacteria	g__Actinoalloteichus	0.005501	0.003495	0.0007416	0.000177	0.000183	0.001654	-0.00706	-0.00298	-0.00476
p__Actinobacteria	g__Kineosporia	0.005928	0.001833	0.001499	0.000343	0.000183	0.001654	-0.00559	-0.00331	-0.00443
p__Actinobacteria	g__Phytoactinopolyspora	0.007689	0.002635	0.003378	0.000587	0.000183	0.001654	-0.00593	-0.00283	-0.00431
p__Actinobacteria	g__unclassified_o__Streptosporangiales	0.009756	0.002666	0.005598	0.000671	0.000246	0.001813	-0.00585	-0.00268	-0.00416
p__Actinobacteria	g__Kitasatospora	0.01023	0.002382	0.006073	0.001097	0.00044	0.002332	-0.00583	-0.00262	-0.00416
p__Actinobacteria	g__Gandjariella	0.00604	0.004335	0.001985	0.00035	0.000183	0.001654	-0.00703	-0.00204	-0.00406
p__Actinobacteria	g__unclassified_f__Iamiaceae	0.007865	0.001356	0.003899	0.000567	0.000183	0.001654	-0.00486	-0.00309	-0.00397
p__Actinobacteria	g__Phytohabitans	0.008873	0.002874	0.004957	0.000977	0.000583	0.002674	-0.0058	-0.00228	-0.00392
p__Actinobacteria	g__unclassified_o__Solirubrobacterales	0.01023	0.001859	0.006427	0.001341	0.000583	0.002674	-0.00511	-0.00247	-0.0038
p__Actinobacteria	g__Nocardiopsis	0.008097	0.002881	0.004333	0.000565	0.001315	0.004744	-0.00568	-0.00196	-0.00377
p__Actinobacteria	g__unclassified_f__Ilumatobacteraceae	0.006271	0.002849	0.002529	0.000956	0.00044	0.002332	-0.0057	-0.00214	-0.00374
p__Actinobacteria	g__Salinispora	0.00437	0.003093	0.0007151	0.000113	0.000183	0.001654	-0.00582	-0.00222	-0.00366
p__Actinobacteria	g__Goodfellowiella	0.00387	0.002844	0.0004304	0.000221	0.000183	0.001654	-0.00536	-0.00216	-0.00344
p__Actinobacteria	g__Yuhushiella	0.004458	0.002739	0.001052	0.000404	0.000246	0.001813	-0.00514	-0.00182	-0.00341
p__Actinobacteria	g__unclassified_f__Micromonosporaceae	0.007229	0.002685	0.003964	0.001162	0.001315	0.004744	-0.00513	-0.0017	-0.00327
p__Actinobacteria	g__Actinopolymorpha	0.006055	0.002091	0.002826	0.000333	0.000183	0.001654	-0.0044	-0.00201	-0.00323
p__Actinobacteria	g__Actinocrispum	0.00472	0.002886	0.00168	0.000265	0.00033	0.002032	-0.00498	-0.00166	-0.00304

p__Actinobacteria	g__Arthrobacter	0.01023	0.002714	0.007203	0.001448	0.007285	0.01777	-0.00475	-0.00133	-0.00303
p__Actinobacteria	g__Microbispora	0.008075	0.002807	0.005223	0.000911	0.009108	0.0215	-0.0048	-0.00122	-0.00285
p__Actinobacteria	g__Conexibacter	0.006944	0.001312	0.004225	0.000742	0.000769	0.003257	-0.00363	-0.00184	-0.00272
p__Actinobacteria	g__Gordonia_f__Gordoniaceae	0.005014	0.0005153	0.002355	0.000333	0.000183	0.001654	-0.00303	-0.0023	-0.00266
p__Actinobacteria	g__Nakamurella	0.005455	0.0004961	0.002896	0.000569	0.000183	0.001654	-0.00298	-0.00206	-0.00256
p__Actinobacteria	g__Verrucosispora	0.003625	0.001177	0.001201	0.000287	0.000183	0.001654	-0.00317	-0.00175	-0.00242
p__Actinobacteria	g__Georgenia	0.004092	0.0007661	0.001839	0.000449	0.000183	0.001654	-0.00278	-0.00172	-0.00225
p__Actinobacteria	g__Cryptosporangium	0.004656	0.00117	0.002578	0.000385	0.00044	0.002332	-0.00283	-0.0014	-0.00208
p__Actinobacteria	g__unclassified_o__Frankiales	0.003517	0.0009242	0.001477	0.000335	0.000183	0.001654	-0.00263	-0.00145	-0.00204
p__Actinobacteria	g__Catellatospora	0.004816	0.001142	0.002777	0.000477	0.000583	0.002674	-0.00273	-0.00128	-0.00204
p__Actinobacteria	g__unclassified_o__Actinomycetales	0.004185	0.001245	0.002204	0.000765	0.001008	0.003952	-0.00285	-0.0011	-0.00198
p__Actinobacteria	g__Planosporangium	0.004874	0.00182	0.002934	0.000704	0.002827	0.00826	-0.00315	-0.00089	-0.00194
p__Actinobacteria	g__Nitriliruptor	0.003865	0.0007041	0.001957	0.00054	0.000183	0.001654	-0.00242	-0.00138	-0.00191
p__Actinobacteria	g__Dactylosporangium	0.00238	0.001263	0.0005971	0.000149	0.000183	0.001654	-0.0026	-0.00113	-0.00178
p__Actinobacteria	g__Mycobacteroides	0.003882	0.000976	0.002102	0.000522	0.000183	0.001654	-0.00243	-0.0012	-0.00178
p__Actinobacteria	g__Cryobacterium	0.003755	0.001219	0.001977	0.00037	0.00033	0.002032	-0.00257	-0.00109	-0.00178
p__Actinobacteria	g__Acrocarpospora	0.005361	0.001529	0.003603	0.000468	0.01402	0.03047	-0.00268	-0.00078	-0.00176
p__Actinobacteria	g__Crossiella	0.002143	0.001506	0.0004076	0.000221	0.000246	0.001813	-0.00267	-0.00096	-0.00174
p__Actinobacteria	g__Phycicoccus	0.002865	0.0005918	0.001166	0.000317	0.000183	0.001654	-0.00208	-0.00127	-0.0017
p__Actinobacteria	g__Aeromicrobium	0.003582	0.00094	0.001894	0.000415	0.000246	0.001813	-0.00229	-0.00109	-0.00169
p__Actinobacteria	g__Marmoricola	0.003403	0.00083	0.001774	0.000438	0.000183	0.001654	-0.00221	-0.00106	-0.00163
p__Actinobacteria	g__Thermoactinospora	0.00196	0.0007783	0.0003471	6.98E-05	0.000183	0.001654	-0.00208	-0.00115	-0.00161
p__Actinobacteria	g__Corynebacterium	0.003073	0.0008978	0.001477	0.000445	0.00044	0.002332	-0.0022	-0.00104	-0.0016
p__Actinobacteria	g__Motilibacter	0.003404	0.0008047	0.001856	0.000363	0.000183	0.001654	-0.00207	-0.00108	-0.00155
p__Actinobacteria	g__Actinopolyspora	0.001897	0.001008	0.0003843	0.000141	0.000183	0.001654	-0.00218	-0.00098	-0.00151
p__Actinobacteria	g__Humibacillus	0.002262	0.0004976	0.001009	0.000332	0.000246	0.001813	-0.0016	-0.00093	-0.00125
p__Actinobacteria	g__Candidatus_Microthrix	0.002831	0.0007589	0.00162	0.000574	0.002202	0.00693	-0.00181	-0.00066	-0.00121
p__Actinobacteria	g__Streptacidiphilus	0.004039	0.001094	0.002864	0.00024	0.002827	0.00826	-0.00188	-0.00049	-0.00118
p__Actinobacteria	g__unclassified_o__Nitriliruptorales	0.003616	0.0009308	0.002445	0.000297	0.003611	0.01003	-0.00175	-0.00055	-0.00117
p__Actinobacteria	g__unclassified_f__Mycobacteriaceae	0.002283	0.0008139	0.00114	0.000355	0.00044	0.002332	-0.00171	-0.00066	-0.00114
p__Actinobacteria	g__Janibacter	0.001667	0.0005654	0.000542	0.000193	0.000183	0.001654	-0.00149	-0.00078	-0.00113
p__Actinobacteria	g__unclassified_f__Nocardiaceae	0.001336	0.0004356	0.0002112	7.89E-05	0.000183	0.001654	-0.00141	-0.00086	-0.00112
p__Actinobacteria	g__Glycomyces	0.00229	0.0005662	0.001174	0.000328	0.000183	0.001654	-0.00149	-0.00074	-0.00112
p__Actinobacteria	g__Fodinicola	0.001682	0.0008424	0.0005823	0.000143	0.000183	0.001654	-0.00163	-0.00064	-0.0011
p__Actinobacteria	g__Krasilnikovia	0.001312	0.0005014	0.0002297	8.12E-05	0.000183	0.001654	-0.0014	-0.00079	-0.00108
p__Actinobacteria	g__Agrococcus	0.002156	0.0006694	0.001097	0.000213	0.000183	0.001654	-0.00148	-0.00069	-0.00106
p__Actinobacteria	g__Kineococcus	0.002168	0.0006237	0.001126	0.000272	0.001315	0.004744	-0.00145	-0.00064	-0.00104
p__Actinobacteria	g__Herbidospora	0.003003	0.0009531	0.001978	0.000489	0.03121	0.05963	-0.00172	-0.00042	-0.00103
p__Actinobacteria	g__Microlunatus	0.003106	0.0009067	0.002088	0.000546	0.009108	0.0215	-0.00167	-0.00038	-0.00102
p__Actinobacteria	g__Leifsonia	0.002888	0.0009283	0.001875	0.000338	0.000769	0.003257	-0.00165	-0.00047	-0.00101
p__Actinobacteria	g__Isoptericola	0.001893	0.0002564	0.0008928	0.000369	0.000583	0.002674	-0.00125	-0.00071	-0.001
p__Actinobacteria	g__Couchioplanes	0.001494	0.0006152	0.0005217	0.000201	0.00033	0.002032	-0.00134	-0.00059	-0.00097
p__Actinobacteria	g__Tetrasphaera	0.001675	0.0005055	0.000705	8.98E-05	0.00044	0.002332	-0.00126	-0.00067	-0.00097
p__Actinobacteria	g__Klenkia	0.001283	0.0004476	0.0003139	8.12E-05	0.000183	0.001654	-0.00124	-0.00071	-0.00097
p__Actinobacteria	g__unclassified_f__Geodermatophilaceae	0.001322	0.0004291	0.0003562	8.36E-05	0.000183	0.001654	-0.0012	-0.0007	-0.00097
p__Actinobacteria	g__Egibacter	0.001425	0.0006158	0.0004766	9.80E-05	0.000183	0.001654	-0.00133	-0.00061	-0.00095
p__Actinobacteria	g__Plantactinospora	0.002347	0.0009738	0.001412	0.000229	0.001706	0.00577	-0.00163	-0.00044	-0.00093
p__Actinobacteria	g__Actinoallomurus	0.001527	0.0007274	0.0006032	0.000104	0.00033	0.002032	-0.00139	-0.00052	-0.00092

p__Actinobacteria	g__Euzebya	0.002386	0.0006263	0.001478	0.000494	0.005795	0.01483	-0.00137	-0.00041	-0.00091
p__Actinobacteria	g__Catelliglobosispora	0.001698	0.0004282	0.000795	0.000148	0.000183	0.001654	-0.00116	-0.00064	-0.0009
p__Actinobacteria	g__unclassified_o__Propionibacteriales	0.001976	0.0008007	0.001089	0.000467	0.009108	0.0215	-0.00142	-0.00033	-0.00089
p__Actinobacteria	g__Haloechinothrix	0.001198	0.0005173	0.0003265	7.81E-05	0.000183	0.001654	-0.00117	-0.00056	-0.00087
p__Actinobacteria	g__Embleya	0.002241	0.0005045	0.001373	0.000372	0.000583	0.002674	-0.00125	-0.00052	-0.00087
p__Actinobacteria	g__Catenulispora	0.001637	0.0008366	0.0007969	0.000191	0.00044	0.002332	-0.00142	-0.00043	-0.00084
p__Actinobacteria	g__Planomonospora	0.001454	0.000481	0.0006197	0.000159	0.000183	0.001654	-0.00117	-0.00055	-0.00083
p__Actinobacteria	g__Luteipulveratus	0.001071	0.0003269	0.0002535	6.71E-05	0.000183	0.001654	-0.00101	-0.00062	-0.00082
p__Actinobacteria	g__Knoellia	0.001643	0.0004544	0.0008294	0.000105	0.000183	0.001654	-0.0011	-0.00054	-0.00081
p__Actinobacteria	g__Planobispora	0.001429	0.0003259	0.0006213	0.000155	0.000183	0.001654	-0.00103	-0.00061	-0.00081
p__Actinobacteria	g__Thermobifida	0.001306	0.0005927	0.0005144	8.27E-05	0.000183	0.001654	-0.00117	-0.00047	-0.00079
p__Actinobacteria	g__Tamaricihabitans	0.0009009	0.0003102	0.0001211	5.86E-05	0.000183	0.001654	-0.00097	-0.00059	-0.00078
p__Actinobacteria	g__Mycetocola	0.001528	0.0004689	0.000752	0.000253	0.000583	0.002674	-0.00111	-0.00048	-0.00078
p__Actinobacteria	g__Sphaerisporangium	0.001863	0.000685	0.001102	0.000323	0.007285	0.01777	-0.00121	-0.00031	-0.00076
p__Actinobacteria	g__Quadrisphaera	0.0008898	0.0003468	0.0001362	4.02E-05	0.000183	0.001654	-0.00097	-0.00056	-0.00075
p__Actinobacteria	g__Propionibacterium	0.001643	0.0003535	0.0008956	0.000244	0.00044	0.002332	-0.001	-0.00047	-0.00075
p__Actinobacteria	g__Cellulosimicrobium	0.001418	0.0004216	0.0006806	0.00011	0.000183	0.001654	-0.00102	-0.0005	-0.00074
p__Actinobacteria	g__Acidiferrimicrobium	0.001012	0.0003051	0.0003089	0.000115	0.000183	0.001654	-0.0009	-0.00052	-0.0007
p__Actinobacteria	g__Streptomonospora	0.0009167	0.0003156	0.000223	1.00E-04	0.000183	0.001654	-0.00089	-0.0005	-0.00069
p__Actinobacteria	g__Diaminobutyricimonas	0.0008555	0.0008367	0.0001719	6.23E-05	0.000183	0.001654	-0.00125	-0.00025	-0.00068
p__Actinobacteria	g__Kocuria	0.001692	0.000482	0.001028	0.000169	0.009108	0.0215	-0.00094	-0.00035	-0.00066
p__Actinobacteria	g__Longispora_f__Micromonosporaceae	0.001195	0.0004626	0.0005346	0.000244	0.001008	0.003952	-0.00098	-0.00037	-0.00066
p__Actinobacteria	g__Microtetraspora	0.001875	0.000242	0.001223	0.000333	0.001315	0.004744	-0.00088	-0.0004	-0.00065
p__Actinobacteria	g__Thermomonospora	0.001479	0.0006127	0.0008321	0.000154	0.00044	0.002332	-0.00107	-0.00032	-0.00065
p__Actinobacteria	g__Halopolyspora	0.000781	0.0006302	0.0001386	6.63E-05	0.000183	0.001654	-0.00105	-0.00035	-0.00064
p__Actinobacteria	g__Baekduia	0.00122	0.0003935	0.0005801	0.000128	0.000583	0.002674	-0.00089	-0.0004	-0.00064
p__Actinobacteria	g__Auraticoccus	0.0007501	0.0002665	0.0001148	5.43E-05	0.000183	0.001654	-0.00079	-0.00047	-0.00064
p__Actinobacteria	g__Sciscionella	0.000918	0.0003034	0.0002859	8.50E-05	0.000183	0.001654	-0.00083	-0.00047	-0.00063
p__Actinobacteria	g__Haloactinobacterium	0.001928	0.0006368	0.0013	0.001226	0.005795	0.01483	-0.00134	0.000225	-0.00063
p__Actinobacteria	g__Haloactinopolyspora	0.0009344	0.0006651	0.0003078	0.000109	0.00033	0.002032	-0.00105	-0.00033	-0.00063
p__Actinobacteria	g__Asanoa	0.002705	0.0004141	0.002081	0.000523	0.01726	0.03608	-0.00101	-0.00024	-0.00062
p__Actinobacteria	g__Allorhizocola	0.001179	0.0004713	0.0005649	0.000138	0.001315	0.004744	-0.00092	-0.0003	-0.00061
p__Actinobacteria	g__Oerskovia	0.000763	0.000257	0.000152	7.40E-05	0.000183	0.001654	-0.00077	-0.00045	-0.00061
p__Actinobacteria	g__Allonocardiopsis	0.0009518	0.0004227	0.0003579	0.000109	0.000583	0.002674	-0.00088	-0.00036	-0.00059
p__Actinobacteria	g__Rathayibacter	0.001046	0.0005676	0.0004593	0.000164	0.003611	0.01003	-0.00096	-0.00025	-0.00059
p__Actinobacteria	g__Actinomyces	0.004447	0.0004882	0.00387	0.000502	0.03121	0.05963	-0.00096	-0.00016	-0.00058
p__Actinobacteria	g__Thermostaphylospora	0.0009292	0.0004447	0.0003544	9.79E-05	0.00033	0.002032	-0.00086	-0.00033	-0.00057
p__Actinobacteria	g__unclassified_f__Microbacteriaceae	0.001421	0.0003504	0.0008491	0.000162	0.000583	0.002674	-0.00082	-0.00035	-0.00057
p__Actinobacteria	g__Actinospica	0.0006292	0.0003171	9.85E-05	8.16E-05	0.00033	0.002032	-0.00072	-0.00035	-0.00053
p__Actinobacteria	g__Hamadaea	0.001897	0.0003999	0.001394	0.000532	0.02113	0.04273	-0.00088	-7.75E-05	-0.0005
p__Actinobacteria	g__Pseudarthrobacter	0.001235	0.0002183	0.0007478	0.000284	0.001706	0.00577	-0.00068	-0.00026	-0.00049
p__Actinobacteria	g__Bifidobacterium	0.0009679	0.000287	0.0004961	0.00015	0.00044	0.002332	-0.00067	-0.0003	-0.00047
p__Actinobacteria	g__Friedmanniella	0.0008199	0.0001355	0.0003572	0.000139	0.000183	0.001654	-0.00058	-0.00035	-0.00046
p__Actinobacteria	g__unclassified_f__Nocardiopsaceae	0.0004946	0.0001944	3.20E-05	1.64E-05	0.000183	0.001654	-0.00057	-0.00035	-0.00046
p__Actinobacteria	g__Cumulibacter	0.0006233	0.0002522	0.0001668	4.63E-05	0.000183	0.001654	-0.0006	-0.00031	-0.00046
p__Actinobacteria	g__Murinocardiopsis	0.0007054	0.0002414	0.0002713	9.81E-05	0.000583	0.002674	-0.00059	-0.00027	-0.00043
p__Actinobacteria	g__Oryzihumus	0.00118	0.0001709	0.0007537	0.000178	0.000769	0.003257	-0.00056	-0.00027	-0.00043
p__Actinobacteria	g__Glaciibacter	0.000574	0.0003698	0.000158	7.84E-05	0.00033	0.002032	-0.00066	-0.00021	-0.00042

p__Actinobacteria	g__Mycolicibacter	0.001325	0.0002314	0.0009356	0.000183	0.001706	0.00577	-0.00057	-0.00022	-0.00039
p__Actinobacteria	g__Haloactinospora	0.0005426	0.0001736	0.0001565	6.27E-05	0.000183	0.001654	-0.0005	-0.00027	-0.00039
p__Actinobacteria	g__Beutenbergia	0.00066	0.0002545	0.0002765	9.43E-05	0.000183	0.001654	-0.00055	-0.00024	-0.00038
p__Actinobacteria	g__Thermopolyspora	0.001205	0.0004326	0.0008247	0.000204	0.02113	0.04273	-0.00069	-0.00012	-0.00038
p__Actinobacteria	g__Sanguibacter	0.0006139	0.000154	0.0002485	3.17E-05	0.000183	0.001654	-0.00046	-0.00027	-0.00037
p__Actinobacteria	g__Mumia	0.001177	0.0002505	0.0008166	0.000268	0.01133	0.02551	-0.00057	-0.00014	-0.00036
p__Actinobacteria	g__Desertiactinospora	0.0004985	0.0002332	0.0001394	6.57E-05	0.000246	0.001813	-0.00051	-0.00021	-0.00036
p__Actinobacteria	g__Lapillicoccus	0.0004809	0.0001837	0.0001226	5.88E-05	0.000183	0.001654	-0.00048	-0.00025	-0.00036
p__Actinobacteria	g__Marinitenerispora	0.0007165	0.0002511	0.0003796	0.000171	0.001706	0.00577	-0.00054	-0.00017	-0.00034
p__Actinobacteria	g__Sinosporangium	0.0007656	0.0003498	0.0004335	0.000123	0.007285	0.01777	-0.00056	-0.00012	-0.00033
p__Actinobacteria	g__Curtobacterium	0.001234	0.0002574	0.0009082	0.000171	0.004586	0.01226	-0.00051	-0.00016	-0.00033
p__Actinobacteria	g__Pseudactinotalea	0.0006337	0.0002108	0.0003252	0.000125	0.002202	0.00693	-0.00045	-0.00017	-0.00031
p__Actinobacteria	g__Intrasporangium	0.0009776	0.0002375	0.0006791	0.000143	0.007285	0.01777	-0.00046	-0.00013	-0.0003
p__Actinobacteria	g__Smaragdicoccus	0.0005068	0.0002021	0.0002222	9.32E-05	0.001706	0.00577	-0.00042	-0.00015	-0.00028
p__Actinobacteria	g__Serinicoccus	0.0005537	0.000285	0.0002756	4.54E-05	0.01133	0.02551	-0.00045	-0.00012	-0.00028
p__Actinobacteria	g__unclassified_f__Kineosporiaceae	0.0003933	0.0001182	0.0001222	5.23E-05	0.000183	0.001654	-0.00035	-0.0002	-0.00027
p__Actinobacteria	g__Acidothermus	0.0004938	0.0002318	0.0002285	6.05E-05	0.000583	0.002674	-0.00041	-0.00014	-0.00027
p__Actinobacteria	g__unclassified_f__Nocardioidaceae	0.0005255	0.0001352	0.0002606	0.000108	0.000769	0.003257	-0.00037	-0.00016	-0.00027
p__Actinobacteria	g__Schaalia	0.0004437	0.0001935	0.0001854	5.70E-05	0.000769	0.003257	-0.00038	-0.00014	-0.00026
p__Actinobacteria	g__Antribacter	0.0005041	0.0001067	0.0002462	0.000101	0.000583	0.002674	-0.00033	-0.00017	-0.00026
p__Actinobacteria	g__Tessaracoccus	0.0007956	0.0001197	0.0005406	0.000155	0.007285	0.01777	-0.00036	-0.00013	-0.00026
p__Actinobacteria	g__Jishengella	0.0005374	0.0002545	0.000283	0.000144	0.01402	0.03047	-0.00043	-9.28E-05	-0.00025
p__Actinobacteria	g__Arsenicicoccus	0.000459	9.95E-05	0.0002194	7.30E-05	0.000183	0.001654	-0.00031	-0.00016	-0.00024
p__Actinobacteria	g__Thermobispora	0.0003646	0.0001692	0.0001295	3.91E-05	0.000246	0.001813	-0.00035	-0.00015	-0.00024
p__Actinobacteria	g__Leekyejoonella	0.0007222	0.000159	0.0004875	0.000277	0.005795	0.01483	-0.00042	-3.09E-05	-0.00023
p__Actinobacteria	g__Spirillospora	0.0003214	0.0001544	8.79E-05	3.27E-05	0.000183	0.001654	-0.00033	-0.00014	-0.00023
p__Actinobacteria	g__Nesterenkonia	0.0004822	0.0001806	0.0002565	0.000114	0.005795	0.01483	-0.00035	-9.98E-05	-0.00023
p__Actinobacteria	g__Sediminihabitans	0.000292	0.0001151	6.89E-05	2.38E-05	0.000183	0.001654	-0.00029	-0.00016	-0.00022
p__Actinobacteria	g__Agreia	0.0003798	0.0001159	0.0001618	6.01E-05	0.00033	0.002032	-0.0003	-0.00014	-0.00022
p__Actinobacteria	g__Actinorugispora	0.0002815	0.0002192	6.38E-05	2.70E-05	0.000769	0.003257	-0.00037	-0.00011	-0.00022
p__Actinobacteria	g__Microterricola	0.0005131	0.0002271	0.0002982	0.000125	0.01402	0.03047	-0.00037	-7.52E-05	-0.00021
p__Actinobacteria	g__Frigoribacterium	0.0004928	0.0001297	0.0002805	6.42E-05	0.001315	0.004744	-0.0003	-0.00013	-0.00021
p__Actinobacteria	g__Pseudoclavibacter	0.000512	0.0002054	0.0003131	7.69E-05	0.01402	0.03047	-0.00034	-6.80E-05	-0.0002
p__Actinobacteria	g__unclassified_f__Streptomycetaceae	0.000355	0.000156	0.0001595	4.35E-05	0.001008	0.003952	-0.00029	-0.0001	-0.0002
p__Actinobacteria	g__Ornithinibacter	0.0003447	0.0001033	0.0001498	5.60E-05	0.00044	0.002332	-0.00027	-0.00013	-0.0002
p__Actinobacteria	g__Barrientosiimonas	0.0002879	0.0001426	9.52E-05	5.52E-05	0.002202	0.00693	-0.00028	-0.0001	-0.00019
p__Actinobacteria	g__Mobilicoccus	0.0002652	0.0001223	7.67E-05	4.64E-05	0.00044	0.002332	-0.00027	-0.00012	-0.00019
p__Actinobacteria	g__Paeniglutamicibacter	0.0003642	8.89E-05	0.0001776	9.32E-05	0.002202	0.00693	-0.00026	-0.00011	-0.00019
p__Actinobacteria	g__Acidithrix	0.0004477	0.0001151	0.0002777	7.48E-05	0.003611	0.01003	-0.00026	-8.67E-05	-0.00017
p__Actinobacteria	g__Catenuloplanes	0.000394	0.0001174	0.0002248	6.88E-05	0.003611	0.01003	-0.00025	-8.81E-05	-0.00017
p__Actinobacteria	g__Thermasporomyces	0.0006391	0.0002168	0.0004708	0.000113	0.04515	0.08009	-0.00032	-2.85E-05	-0.00017
p__Actinobacteria	g__Antricoccus	0.0004628	0.0001481	0.0002951	9.97E-05	0.01726	0.03608	-0.00027	-6.48E-05	-0.00017
p__Actinobacteria	g__Planctomonas	0.0002313	3.05E-05	6.69E-05	1.57E-05	0.000183	0.001654	-0.00019	-0.00014	-0.00016
p__Actinobacteria	g__Thermoleophilum	0.0004068	8.01E-05	0.0002446	8.82E-05	0.001706	0.00577	-0.00023	-9.23E-05	-0.00016
p__Actinobacteria	g__Acidimicrobium	0.0003484	0.0001047	0.0001876	7.95E-05	0.001008	0.003952	-0.00023	-8.38E-05	-0.00016
p__Actinobacteria	g__Mycolicibacillus	0.0001862	3.99E-05	2.70E-05	1.80E-05	0.000183	0.001654	-0.00019	-0.00014	-0.00016
p__Actinobacteria	g__Rudaeicoccus	0.0001799	7.14E-05	2.29E-05	2.04E-05	0.000183	0.001654	-0.0002	-0.00012	-0.00016
p__Actinobacteria	g__Krasilnikoviella	0.0002318	9.25E-05	7.52E-05	4.27E-05	0.00033	0.002032	-0.00022	-9.62E-05	-0.00016

p__Actinobacteria	g__Pseudokineococcus	0.0003503	0.0001744	0.000195	7.82E-05	0.03764	0.06884	-0.00028	-4.54E-05	-0.00016
p__Actinobacteria	g__Brachybacterium	0.0004548	0.0001602	0.000302	9.23E-05	0.02575	0.05065	-0.00026	-4.74E-05	-0.00015
p__Actinobacteria	g__Micropruina	0.0002811	0.0001097	0.000132	3.47E-05	0.000583	0.002674	-0.00022	-8.73E-05	-0.00015
p__Actinobacteria	g__Desertihabitans	0.0002369	8.99E-05	8.96E-05	4.17E-05	0.00044	0.002332	-0.00021	-9.31E-05	-0.00015
p__Actinobacteria	g__Flexivirga	0.0003214	0.0001028	0.0001751	9.28E-05	0.004586	0.01226	-0.00023	-6.72E-05	-0.00015
p__Actinobacteria	g__Marisediminicola	0.0001971	6.84E-05	5.96E-05	3.03E-05	0.000183	0.001654	-0.00018	-9.80E-05	-0.00014
p__Actinobacteria	g__Dietzia	0.0003208	7.92E-05	0.0001877	4.75E-05	0.002827	0.00826	-0.00019	-7.07E-05	-0.00013
p__Actinobacteria	g__Piscicoccus	0.0001904	7.17E-05	5.92E-05	3.50E-05	0.000769	0.003257	-0.00018	-8.54E-05	-0.00013
p__Actinobacteria	g__Cutibacterium	0.0002368	0.0001095	0.0001151	6.09E-05	0.01133	0.02551	-0.0002	-4.55E-05	-0.00012
p__Actinobacteria	g__Propionicicella	0.000152	0.0001007	3.22E-05	3.44E-05	0.004586	0.01226	-0.00018	-5.55E-05	-0.00012
p__Actinobacteria	g__Millisia	0.0002306	3.71E-05	0.0001118	3.53E-05	0.000246	0.001813	-0.00015	-8.55E-05	-0.00012
p__Actinobacteria	g__Luteimicrobium	0.0001741	5.27E-05	5.74E-05	3.19E-05	0.00044	0.002332	-0.00015	-8.25E-05	-0.00012
p__Actinobacteria	g__Paenarthrobacter	0.0002401	0.0001159	0.0001248	6.86E-05	0.02575	0.05065	-0.00019	-3.90E-05	-0.00012
p__Actinobacteria	g__Tsukamurella	0.0004558	0.0001012	0.0003415	9.05E-05	0.01726	0.03608	-0.0002	-4.06E-05	-0.00011
p__Actinobacteria	g__Microcella	0.0001787	0.0001164	6.54E-05	3.41E-05	0.001315	0.004744	-0.00019	-5.16E-05	-0.00011
p__Actinobacteria	g__Dermacoccus	0.0001791	4.73E-05	7.25E-05	3.06E-05	0.00033	0.002032	-0.00014	-7.16E-05	-0.00011
p__Actinobacteria	g__Hoyosella	0.0002482	4.98E-05	0.0001444	7.84E-05	0.005795	0.01483	-0.00015	-4.41E-05	-0.0001
p__Actinobacteria	g__Myceligenerans	0.0001364	6.57E-05	3.34E-05	2.02E-05	0.00044	0.002332	-0.00014	-6.53E-05	-0.0001
p__Actinobacteria	g__Flavimobilis	0.0001657	7.28E-05	6.51E-05	4.31E-05	0.003611	0.01003	-0.00015	-4.95E-05	-0.0001
p__Actinobacteria	g__Calidifontibacter	0.0001706	3.76E-05	7.41E-05	4.56E-05	0.000769	0.003257	-0.00013	-5.99E-05	-9.65E-05
p__Actinobacteria	g__Tomitella	0.0002236	4.14E-05	0.0001274	5.35E-05	0.002202	0.00693	-0.00013	-5.27E-05	-9.62E-05
p__Actinobacteria	g__Branchiibius	0.0002172	5.06E-05	0.0001211	4.04E-05	0.001706	0.00577	-0.00013	-5.86E-05	-9.61E-05
p__Actinobacteria	g__Frondihabitans	0.0001527	0.0001345	6.12E-05	3.83E-05	0.03121	0.05963	-0.00018	-1.62E-05	-9.15E-05
p__Actinobacteria	g__Egicoccus	0.0003173	0.0001077	0.0002301	2.33E-05	0.02575	0.05065	-0.00015	-2.62E-05	-8.73E-05
p__Actinobacteria	g__Gulosibacter	0.000146	6.07E-05	6.51E-05	3.11E-05	0.003611	0.01003	-0.00013	-3.76E-05	-8.09E-05
p__Actinobacteria	g__Kribbia	8.96E-05	4.02E-05	1.12E-05	1.03E-05	0.000183	0.001654	-0.0001	-5.48E-05	-7.85E-05
p__Actinobacteria	g__Lysinimonas	7.69E-05	9.56E-05	3.82E-06	5.94E-06	0.002282	0.007141	-0.00013	-1.68E-05	-7.30E-05
p__Actinobacteria	g__Ruania	0.0001723	5.80E-05	0.0001011	5.74E-05	0.01726	0.03608	-0.00012	-2.08E-05	-7.12E-05
p__Actinobacteria	g__Tersicoccus	9.76E-05	3.70E-05	2.92E-05	1.90E-05	0.000246	0.001813	-9.28E-05	-4.48E-05	-6.83E-05
p__Actinobacteria	g__Brevilactibacter	0.000172	4.96E-05	0.0001065	4.76E-05	0.007285	0.01777	-0.0001	-2.64E-05	-6.55E-05
p__Actinobacteria	g__Brooklawnia	0.0001303	4.45E-05	6.56E-05	4.75E-05	0.007285	0.01777	-0.0001	-2.28E-05	-6.48E-05
p__Actinobacteria	g__Xylanimonas	0.0001773	6.37E-05	0.0001132	3.53E-05	0.03764	0.06884	-0.0001	-1.93E-05	-6.42E-05
p__Actinobacteria	g__Actinobaculum	7.24E-05	0.0001207	9.06E-06	1.22E-05	0.006852	0.0174	-0.00014	-1.01E-05	-6.34E-05
p__Actinobacteria	g__Xylanimicrobium	8.71E-05	3.61E-05	2.69E-05	2.28E-05	0.001008	0.003952	-8.46E-05	-3.33E-05	-6.02E-05
p__Actinobacteria	g__Segeticoccus	8.63E-05	4.91E-05	2.79E-05	1.11E-05	0.000583	0.002674	-8.70E-05	-2.99E-05	-5.84E-05
p__Actinobacteria	g__Gryllotalpicola	0.0001335	5.39E-05	7.94E-05	5.65E-05	0.02575	0.05065	-9.97E-05	-4.97E-06	-5.42E-05
p__Actinobacteria	g__Bogoriella_f__Bogoriellaceae	9.80E-05	4.75E-05	4.54E-05	3.28E-05	0.01133	0.02551	-8.57E-05	-1.66E-05	-5.26E-05
p__Actinobacteria	g__unclassified_o__Candidatus_Nanopelagicales	9.82E-05	3.02E-05	4.65E-05	2.04E-05	0.001315	0.004744	-7.31E-05	-3.09E-05	-5.16E-05
p__Actinobacteria	g__Marihabitans	5.50E-05	3.67E-05	1.35E-05	1.48E-05	0.007197	0.01777	-6.58E-05	-1.82E-05	-4.15E-05
p__Actinobacteria	g__Miniimonas	8.20E-05	2.93E-05	4.07E-05	2.61E-05	0.007285	0.01777	-6.64E-05	-1.79E-05	-4.13E-05
p__Actinobacteria	g__Serinibacter	6.12E-05	3.23E-05	2.19E-05	2.02E-05	0.005795	0.01483	-6.38E-05	-1.83E-05	-3.93E-05
p__Actinobacteria	g__Zhihengliuella	5.00E-05	3.38E-05	2.27E-05	1.68E-05	0.04515	0.08009	-5.05E-05	-5.20E-06	-2.73E-05
p__Actinobacteria	g__Polymorphospora	2.50E-05	2.32E-05	0	0	0.000231	0.001813	-3.95E-05	-1.32E-05	-2.50E-05
p__Actinobacteria	g__Lysinibacter	2.57E-05	2.34E-05	1.26E-06	2.79E-06	0.003198	0.009319	-3.94E-05	-1.05E-05	-2.44E-05
p__Actinobacteria	g__Ponticoccus_f__Propionibacteriaceae	3.18E-05	2.70E-05	7.57E-06	1.38E-05	0.005171	0.0138	-4.38E-05	-6.47E-06	-2.42E-05
p__Actinobacteria	g__Devriesea	3.13E-05	2.35E-05	8.42E-06	9.63E-06	0.02464	0.04965	-3.80E-05	-8.78E-06	-2.28E-05
p__Actinobacteria	g__Flaviflexus	3.60E-05	2.08E-05	2.02E-05	1.43E-05	0.02113	0.04273	-3.16E-05	-2.46E-06	-1.58E-05
p__Actinobacteria	g__Enterorhabdus	2.23E-05	1.69E-05	7.62E-06	8.22E-06	0.04354	0.07931	-2.58E-05	-3.52E-06	-1.47E-05

p__Actinobacteria	g__Aurantimicrobium	1.20E-05	1.31E-05	1.70E-06	3.60E-06	0.01095	0.02551	-1.80E-05	-3.24E-06	-1.03E-05
p__Actinobacteria	g__Haematomicrobium	1.48E-05	1.03E-05	5.08E-06	4.58E-06	0.02777	0.05457	-1.61E-05	-2.34E-06	-9.74E-06
p__Actinobacteria	g__Aestuariimicrobium	5.91E-06	8.09E-06	0	0	0.005972	0.01522	-1.20E-05	-1.95E-06	-5.91E-06
p__Actinobacteria	g__Ancrocorticia	4.96E-06	8.49E-06	0	0	0.03498	0.06617	-1.01E-05	-3.85E-07	-4.96E-06
p__Actinobacteria	g__Gleimia	4.83E-06	7.21E-06	4.25E-07	1.35E-06	0.04033	0.07366	-9.38E-06	-7.41E-07	-4.40E-06
p__Firmicutes	g__Peribacillus	0.003898	0.002351	0.0003868	4.99E-05	0.000183	0.001654	-0.00501	-0.00216	-0.00351
p__Firmicutes	g__Propionispora	0.003466	0.00198	0.0005626	0.000253	0.000183	0.001654	-0.0041	-0.00184	-0.0029
p__Firmicutes	g__Kurthia	0.00394	0.001062	0.001255	0.000305	0.000183	0.001654	-0.00331	-0.00204	-0.00269
p__Firmicutes	g__Bacillus_f__Bacillaceae	0.03177	0.002682	0.02919	0.002673	0.04515	0.08009	-0.00473	-0.00032	-0.00259
p__Firmicutes	g__Domibacillus	0.001928	0.0005166	0.0003173	9.06E-05	0.000183	0.001654	-0.00191	-0.00129	-0.00161
p__Firmicutes	g__Desulfotomaculum	0.003762	0.001545	0.002626	0.00025	0.007285	0.01777	-0.0021	-0.00031	-0.00114
p__Firmicutes	g__Staphylococcus	0.002906	0.0003949	0.001898	0.000276	0.00033	0.002032	-0.00129	-0.00072	-0.00101
p__Firmicutes	g__Carboxydothermus	0.001128	0.0005124	0.0002273	6.83E-05	0.000183	0.001654	-0.00122	-0.00059	-0.0009
p__Firmicutes	g__Sulfobacillus	0.002174	0.00094	0.0013	0.000387	0.01133	0.02551	-0.00153	-0.00028	-0.00087
p__Firmicutes	g__Thermoanaerosceptrum	0.001155	0.0004551	0.0004123	0.000127	0.000183	0.001654	-0.00104	-0.00049	-0.00074
p__Firmicutes	g__Intestinimonas	0.001371	0.0001825	0.0007134	0.000358	0.001315	0.004744	-0.00087	-0.00041	-0.00066
p__Firmicutes	g__Dehalobacter	0.0009591	0.0003232	0.0004061	8.39E-05	0.00044	0.002332	-0.00074	-0.00034	-0.00055
p__Firmicutes	g__Desnuesiella	0.0008396	0.0004127	0.0003873	0.000129	0.001706	0.00577	-0.00073	-0.00022	-0.00045
p__Firmicutes	g__Cytobacillus	0.001166	0.0002756	0.0007654	0.000131	0.003611	0.01003	-0.00059	-0.00022	-0.0004
p__Firmicutes	g__unclassified_f__Veillonellaceae	0.0009143	0.0002282	0.0006491	0.000204	0.03121	0.05963	-0.00044	-8.08E-05	-0.00027
p__Firmicutes	g__Megasphaera	0.0004938	0.0001466	0.0002312	7.17E-05	0.001008	0.003952	-0.00035	-0.00016	-0.00026
p__Firmicutes	g__Dethiobacter	0.0005335	0.0001284	0.0002949	0.000259	0.005795	0.01483	-0.00039	-6.31E-05	-0.00024
p__Firmicutes	g__unclassified_o__Bacillales	0.0004387	0.0002135	0.0002007	6.38E-05	0.004586	0.01226	-0.00038	-0.00011	-0.00024
p__Firmicutes	g__Pelosinus	0.0007995	0.0001998	0.0005641	0.000216	0.03764	0.06884	-0.0004	-7.30E-05	-0.00024
p__Firmicutes	g__Propionispira	0.0002627	0.0001393	3.12E-05	1.33E-05	0.000183	0.001654	-0.00031	-0.00015	-0.00023
p__Firmicutes	g__Lysinibacillus	0.0007908	0.0001714	0.0005639	0.000148	0.009108	0.0215	-0.00037	-8.97E-05	-0.00023
p__Firmicutes	g__unclassified_f__Clostridiales_Family_XVII._Incertae_S	0.0004484	0.0001703	0.0002397	0.000111	0.002827	0.00826	-0.00033	-9.13E-05	-0.00021
p__Firmicutes	g__Exiguobacterium	0.0004457	0.0002017	0.000241	4.29E-05	0.002827	0.00826	-0.00034	-9.20E-05	-0.0002
p__Firmicutes	g__Symbiobacterium	0.0008617	0.0002147	0.0006631	0.000151	0.03764	0.06884	-0.00036	-4.07E-05	-0.0002
p__Firmicutes	g__Numidum	0.0004907	0.0001404	0.0003229	0.000103	0.01133	0.02551	-0.00027	-6.98E-05	-0.00017
p__Firmicutes	g__Enterococcus	0.000586	0.0001922	0.0004241	0.000151	0.02575	0.05065	-0.0003	-1.49E-05	-0.00016
p__Firmicutes	g__Desulfurispora	0.0003521	0.0002108	0.0001916	6.80E-05	0.02113	0.04273	-0.0003	-3.58E-05	-0.00016
p__Firmicutes	g__Hydrogenibacillus	0.0003635	0.0001706	0.0002186	7.52E-05	0.03764	0.06884	-0.00026	-4.22E-05	-0.00014
p__Firmicutes	g__Halothermothrix	0.0002405	0.0001719	0.0001258	2.25E-05	0.02113	0.04273	-0.00023	-3.21E-05	-0.00011
p__Firmicutes	g__Chengkuizengella	0.000132	7.85E-05	4.89E-05	4.72E-05	0.02113	0.04273	-0.00014	-2.77E-05	-8.32E-05
p__Firmicutes	g__Dehalobacterium	0.0001167	7.20E-05	3.95E-05	3.26E-05	0.003611	0.01003	-0.00012	-3.15E-05	-7.72E-05
p__Firmicutes	g__Chryseomicrobium	7.61E-05	4.11E-05	1.12E-05	1.46E-05	0.000964	0.003952	-9.28E-05	-4.06E-05	-6.50E-05
p__Firmicutes	g__Paenisporosarcina	9.67E-05	4.82E-05	3.21E-05	2.68E-05	0.003611	0.01003	-9.81E-05	-3.18E-05	-6.46E-05
p__Firmicutes	g__Leuconostoc	8.85E-05	7.83E-05	2.46E-05	2.13E-05	0.007285	0.01777	-0.00011	-2.19E-05	-6.39E-05
p__Firmicutes	g__Gelria	7.34E-05	4.41E-05	9.51E-06	1.05E-05	0.000323	0.002032	-9.35E-05	-3.73E-05	-6.39E-05
p__Firmicutes	g__Hazenella	0.000113	5.24E-05	5.26E-05	4.00E-05	0.004586	0.01226	-9.89E-05	-2.22E-05	-6.04E-05
p__Firmicutes	g__Candidatus_Carbobacillus	1.00E-04	6.43E-05	4.06E-05	1.93E-05	0.007285	0.01777	-9.93E-05	-2.40E-05	-5.93E-05
p__Firmicutes	g__Desulfitibacter	9.74E-05	6.48E-05	4.21E-05	2.12E-05	0.009108	0.0215	-9.80E-05	-2.06E-05	-5.53E-05
p__Firmicutes	g__Caldicoprobacter	0.0001476	6.25E-05	9.27E-05	4.77E-05	0.03121	0.05963	-0.0001	-8.89E-06	-5.49E-05
p__Firmicutes	g__Aquibacillus	9.31E-05	3.61E-05	3.94E-05	2.65E-05	0.002827	0.00826	-8.08E-05	-2.73E-05	-5.37E-05
p__Firmicutes	g__Thermodesulfobium	6.37E-05	3.49E-05	1.13E-05	1.05E-05	0.000572	0.002674	-7.45E-05	-3.08E-05	-5.25E-05
p__Firmicutes	g__Metasolibacillus	7.89E-05	2.83E-05	2.89E-05	1.53E-05	0.001008	0.003952	-6.77E-05	-2.87E-05	-5.00E-05
p__Firmicutes	g__unclassified_f__Oscillospiraceae	6.05E-05	4.66E-05	1.41E-05	1.99E-05	0.006852	0.0174	-7.58E-05	-1.70E-05	-4.64E-05

p__Firmicutes	g__Abyssisolibacter	5.19E-05	3.86E-05	1.01E-05	1.19E-05	0.001638	0.00577	-6.52E-05	-1.93E-05	-4.19E-05
p__Firmicutes	g__Inediibacterium	4.10E-05	1.72E-05	5.44E-06	1.10E-05	0.000397	0.002332	-4.68E-05	-2.29E-05	-3.56E-05
p__Firmicutes	g__Oxobacter	6.99E-05	3.53E-05	3.49E-05	2.55E-05	0.02575	0.05065	-6.11E-05	-8.80E-06	-3.50E-05
p__Firmicutes	g__Hydrogenoanaerobacterium	3.33E-05	3.70E-05	0	0	0.002213	0.00693	-5.32E-05	-1.36E-05	-3.33E-05
p__Firmicutes	g__Thermoclostridium	6.16E-05	2.46E-05	2.83E-05	1.91E-05	0.005795	0.01483	-5.15E-05	-1.33E-05	-3.33E-05
p__Firmicutes	g__Anaeroarcus	6.29E-05	4.97E-05	2.99E-05	3.58E-05	0.04507	0.08009	-7.02E-05	8.96E-07	-3.30E-05
p__Firmicutes	g__Alkalibaculum	4.86E-05	3.46E-05	1.87E-05	1.99E-05	0.03764	0.06884	-5.57E-05	-7.38E-06	-2.99E-05
p__Firmicutes	g__Faecalicatena	3.45E-05	2.85E-05	6.86E-06	6.41E-06	0.000964	0.003952	-4.53E-05	-1.26E-05	-2.77E-05
p__Firmicutes	g__Pseudogracilibacillus	2.74E-05	3.06E-05	9.98E-07	3.16E-06	0.001235	0.004744	-4.60E-05	-9.82E-06	-2.64E-05
p__Firmicutes	g__Caproiciproducens	3.13E-05	1.91E-05	6.04E-06	7.45E-06	0.000556	0.002674	-3.79E-05	-1.49E-05	-2.52E-05
p__Firmicutes	g__Petrocella	2.61E-05	2.40E-05	9.04E-07	1.92E-06	0.003198	0.009319	-4.04E-05	-1.24E-05	-2.52E-05
p__Firmicutes	g__Acetoanaerobium	2.65E-05	1.59E-05	4.27E-06	6.28E-06	0.002811	0.00826	-3.20E-05	-1.13E-05	-2.23E-05
p__Firmicutes	g__Marinilactibacillus	3.06E-05	1.78E-05	9.16E-06	9.61E-06	0.004525	0.01226	-3.35E-05	-8.50E-06	-2.15E-05
p__Firmicutes	g__Clostridiisalibacter	5.45E-05	1.45E-05	3.42E-05	2.70E-05	0.02575	0.05065	-3.73E-05	-4.39E-07	-2.03E-05
p__Firmicutes	g__Tenuibacillus	2.03E-05	1.67E-05	4.99E-07	1.58E-06	0.000167	0.001654	-2.99E-05	-1.04E-05	-1.98E-05
p__Firmicutes	g__Mitsuokella	3.86E-05	1.60E-05	1.93E-05	2.05E-05	0.02113	0.04273	-3.28E-05	-3.12E-06	-1.93E-05
p__Firmicutes	g__Mobilibacterium	1.85E-05	1.71E-05	4.05E-07	1.28E-06	0.001444	0.005199	-2.85E-05	-8.59E-06	-1.81E-05
p__Firmicutes	g__Andreesenia	1.35E-05	9.25E-06	0	0	0.000231	0.001813	-1.87E-05	-8.19E-06	-1.35E-05
p__Firmicutes	g__Lottiidibacillus	1.16E-05	1.25E-05	8.10E-07	2.56E-06	0.01875	0.03907	-1.89E-05	-3.99E-06	-1.08E-05
p__Firmicutes	g__Thermosyntropha	9.28E-06	1.31E-05	0	0	0.01493	0.03223	-1.73E-05	-2.44E-06	-9.28E-06
p__Firmicutes	g__Colibacter	9.64E-06	7.55E-06	4.69E-07	1.48E-06	0.001916	0.006453	-1.42E-05	-4.97E-06	-9.17E-06
p__Firmicutes	g__unclassified_o__Tissierellales	8.36E-06	7.88E-06	0	0	0.000751	0.003257	-1.36E-05	-4.22E-06	-8.36E-06
p__Firmicutes	g__Centipeda_f__Selenomonadaceae	7.73E-06	9.61E-06	0	0	0.01493	0.03223	-1.32E-05	-2.65E-06	-7.73E-06
p__Firmicutes	g__Robinsoniella	8.39E-06	9.75E-06	7.64E-07	2.42E-06	0.00817	0.01988	-1.40E-05	-2.30E-06	-7.63E-06
p__Firmicutes	g__Massilimaliae	7.18E-06	8.56E-06	0	0	0.01493	0.03223	-1.21E-05	-2.26E-06	-7.18E-06
p__Firmicutes	g__Eggerthia	7.08E-06	1.35E-05	0	0	0.03498	0.06617	-1.57E-05	-7.69E-07	-7.08E-06
p__Firmicutes	g__Oenococcus	1.08E-05	7.35E-06	4.08E-06	5.94E-06	0.03367	0.06426	-1.22E-05	-9.97E-07	-6.72E-06
p__Firmicutes	g__Bavariicoccus	6.33E-06	1.11E-05	0	0	0.03498	0.06617	-1.36E-05	-8.62E-07	-6.33E-06
p__Firmicutes	g__Atopococcus	6.73E-06	1.19E-05	5.15E-07	1.63E-06	0.04033	0.07366	-1.47E-05	-7.48E-07	-6.21E-06
p__Chloroflexi	g__unclassified_o__Thermomicrobiales	0.03855	0.02469	0.005639	0.002696	0.000246	0.001813	-0.04759	-0.01811	-0.03291
p__Chloroflexi	g__Sphaerobacter	0.04051	0.02417	0.01057	0.002219	0.00044	0.002332	-0.04442	-0.01602	-0.02994
p__Chloroflexi	g__unclassified_f__Anaerolineaceae	0.05314	0.02204	0.02354	0.002698	0.000183	0.001654	-0.04324	-0.01714	-0.0296
p__Chloroflexi	g__Thermorudis	0.01879	0.008735	0.008249	0.00102	0.000769	0.003257	-0.01605	-0.00564	-0.01054
p__Chloroflexi	g__unclassified_c__Candidatus_Thermofonsia	0.01346	0.006662	0.003097	0.000788	0.000246	0.001813	-0.01457	-0.00681	-0.01036
p__Chloroflexi	g__Nitrolancea	0.01676	0.007647	0.006641	0.001333	0.000769	0.003257	-0.01479	-0.00573	-0.01012
p__Chloroflexi	g__Ktedonobacter	0.01747	0.003197	0.01313	0.001088	0.000769	0.003257	-0.00655	-0.00237	-0.00434
p__Chloroflexi	g__Kallotenue	0.01442	0.003194	0.01012	0.000961	0.001315	0.004744	-0.00624	-0.00229	-0.0043
p__Chloroflexi	g__unclassified_f__Sphaerobacteraceae	0.006928	0.003547	0.002677	0.000646	0.002202	0.00693	-0.00645	-0.00213	-0.00425
p__Chloroflexi	g__Thermomicrobium	0.004808	0.002673	0.001343	0.000471	0.001008	0.003952	-0.00514	-0.00189	-0.00347
p__Chloroflexi	g__unclassified_f__Chloroflexaceae	0.01471	0.001896	0.01158	0.000946	0.001008	0.003952	-0.0044	-0.00195	-0.00313
p__Chloroflexi	g__Dehalococcoides	0.001508	0.00174	0.0005925	0.000168	0.007285	0.01777	-0.00206	-0.00015	-0.00092
p__Chloroflexi	g__Leptolinea	0.0006863	0.000147	0.0005185	0.000138	0.01402	0.03047	-0.00029	-4.28E-05	-0.00017
p__Chloroflexi	g__Pelolinea	0.0003994	7.71E-05	0.0002456	6.52E-05	0.001315	0.004744	-0.00021	-9.17E-05	-0.00015
p__Verrucomicrobia	g__unclassified_o__Methylacidiphilales	0.003367	0.0003194	0.001987	0.000407	0.000183	0.001654	-0.00171	-0.00108	-0.00138
p__Aquificae	g__Phorcysia	0.001491	0.0009247	0.0002499	0.000113	0.000183	0.001654	-0.00181	-0.00076	-0.00124
p__Aquificae	g__Aquifex	0.0002666	0.0002261	7.25E-05	5.03E-05	0.007285	0.01777	-0.00035	-7.40E-05	-0.00019
p__Aquificae	g__Thermovibrio	0.0002075	0.000188	0.0001002	8.68E-05	0.03121	0.05963	-0.00023	7.88E-06	-0.00011
p__Balneolaeota	g__Balneola	0.001874	0.0006292	0.001331	0.000465	0.01402	0.03047	-0.00103	-7.93E-05	-0.00054

p__Balneolaeota	g__Candidatus_Cyclonatronum	0.0002561	0.0001547	9.00E-05	6.64E-05	0.01133	0.02551	-0.00026	-6.91E-05	-0.00017
p__candidate_division	g__Candidatus_Methylomirabilis	0.0141	0.001974	0.0105	0.001683	0.002202	0.00693	-0.00498	-0.00201	-0.0036
p__Candidatus_Azam	g__unclassified_p__Candidatus_Azambacteria	0.001718	0.0003795	0.001065	0.000142	0.00044	0.002332	-0.00091	-0.00042	-0.00065
p__Candidatus_Beckw	g__unclassified_p__Candidatus_Beckwithbacteria	0.0004144	0.0001566	0.000214	6.62E-05	0.001315	0.004744	-0.00031	-0.0001	-0.0002
p__Candidatus_Dadab	g__unclassified_p__Candidatus_Dadabacteria	1.653	0.7481	1.022	0.292	0.01726	0.03608	-1.117	-0.1904	-0.6313
p__Candidatus_Dormi	g__Candidatus_Dormibacter	0.0007017	0.0002894	0.0003508	0.000106	0.001706	0.00577	-0.00056	-0.00019	-0.00035
p__Candidatus_Falkov	g__unclassified_p__Candidatus_Falkowbacteria	0.002968	0.00089	0.001867	0.000316	0.000183	0.001654	-0.00171	-0.0006	-0.0011
p__Candidatus_Jorger	g__unclassified_p__Candidatus_Jorgensenbacteria	0.001361	0.0005842	0.0006947	0.000159	0.002202	0.00693	-0.00103	-0.00034	-0.00067
p__Candidatus_Melai	g__unclassified_o__Candidatus_Caenarcaniphilales	0.0003354	0.0001572	0.0001366	6.45E-05	0.001706	0.00577	-0.00031	-0.00011	-0.0002
p__Candidatus_Sacch	g__unclassified_p__Candidatus_Saccharibacteria	0.002756	0.0006164	0.001581	0.000248	0.000183	0.001654	-0.00158	-0.00079	-0.00118
p__Candidatus_Sungb	g__unclassified_p__Candidatus_Sungbacteria	0.0005181	0.0001153	0.0003151	0.000159	0.01133	0.02551	-0.00031	-8.40E-05	-0.0002
p__Candidatus_Terryl	g__unclassified_p__Candidatus_Terrybacteria	0.0005219	0.0001965	0.0002614	5.68E-05	0.002202	0.00693	-0.00038	-0.00013	-0.00026
p__Candidatus_Uhrba	g__unclassified_p__Candidatus_Uhrbacteria	0.008004	0.008654	0.002326	0.000545	0.004586	0.01226	-0.01198	-0.0016	-0.00568
p__Candidatus_Wallb	g__unclassified_p__Candidatus_Wallbacteria	0.0005195	0.0001216	0.0003235	0.000104	0.002827	0.00826	-0.00029	-0.0001	-0.0002
p__Candidatus_Woese	g__unclassified_p__Candidatus_Woesebacteria	0.003813	0.001052	0.002913	0.000523	0.04515	0.08009	-0.00162	-0.00023	-0.0009
p__Candidatus_Zambr	g__unclassified_p__Candidatus_Zambryskibacteria	0.00334	0.001386	0.002449	0.001471	0.03121	0.05963	-0.00204	0.00044	-0.00089
p__Chlamydiae	g__Parachlamydia	0.02584	0.01419	0.00125	0.000445	0.000183	0.001654	-0.03441	-0.01707	-0.02459
p__Chlamydiae	g__unclassified_p__Chlamydiae	0.01983	0.008572	0.005152	0.001212	0.000183	0.001654	-0.02009	-0.00985	-0.01468
p__Chlamydiae	g__unclassified_o__Chlamydiales	0.01463	0.008068	0.0006917	0.000171	0.000183	0.001654	-0.01929	-0.00974	-0.01394
p__Chlamydiae	g__Candidatus_Protochlamydia	0.008437	0.004437	0.0005539	0.00014	0.000183	0.001654	-0.0108	-0.00564	-0.00788
p__Chlamydiae	g__Neochlamydia	0.005029	0.002433	0.000552	7.16E-05	0.000183	0.001654	-0.00581	-0.00313	-0.00448
p__Chlamydiae	g__Waddlia	0.002408	0.001264	0.000155	0.000106	0.000183	0.001654	-0.00302	-0.00154	-0.00225
p__Chlamydiae	g__unclassified_f__Parachlamydiaceae	0.00201	0.001073	0.0001896	0.000123	0.000183	0.001654	-0.00252	-0.00123	-0.00182
p__Chlamydiae	g__Chlamydia	0.002608	0.0008167	0.0008912	0.000192	0.000183	0.001654	-0.00225	-0.00126	-0.00172
p__Chlamydiae	g__Criblamydia	0.001805	0.0008636	0.0001164	5.27E-05	0.000183	0.001654	-0.00226	-0.00123	-0.00169
p__Chlamydiae	g__Candidatus_Rubidus	0.001185	0.0006096	6.12E-05	2.72E-05	0.000183	0.001654	-0.0015	-0.00078	-0.00112
p__Chlamydiae	g__Estrella	0.001205	0.0005766	9.51E-05	6.70E-05	0.000183	0.001654	-0.00148	-0.00079	-0.00111
p__Chlamydiae	g__unclassified_f__Waddliaceae	0.00143	0.0007381	0.0003367	8.21E-05	0.000183	0.001654	-0.00159	-0.00069	-0.00109
p__Chlamydiae	g__Simkania	0.0004245	0.0001305	8.68E-05	3.92E-05	0.000183	0.001654	-0.00042	-0.00026	-0.00034
p__Chlamydiae	g__Candidatus_Rhabdochlamydia	0.000263	8.78E-05	2.48E-05	2.69E-05	0.000182	0.001654	-0.00029	-0.00018	-0.00024
p__Chlamydiae	g__unclassified_o__Anoxychlamydiales	0.0003666	0.0001481	0.0001576	9.81E-05	0.001315	0.004744	-0.00032	-0.00011	-0.00021
p__Chlamydiae	g__unclassified_o__Parachlamydiales	0.0002128	5.06E-05	8.15E-05	3.33E-05	0.000183	0.001654	-0.00017	-9.65E-05	-0.00013
p__Chlamydiae	g__unclassified_c__Chlamydiia	0.0001258	5.23E-05	1.45E-05	1.25E-05	0.000179	0.001654	-0.00014	-7.88E-05	-0.00011
p__Chlamydiae	g__unclassified_f__Simkaniaceae	0.0001913	6.52E-05	8.31E-05	3.63E-05	0.001706	0.00577	-0.00015	-6.33E-05	-0.00011
p__Chlamydiae	g__Candidatus_Similichlamydia	2.36E-05	1.51E-05	7.43E-06	1.48E-05	0.01278	0.02874	-2.90E-05	-3.31E-06	-1.62E-05
p__Chlorobi	g__Pelodictyon	0.002227	0.0005619	0.001439	0.000884	0.004586	0.01226	-0.00135	-7.72E-05	-0.00079
p__Chlorobi	g__Prosthecochloris	0.001217	0.0002442	0.0008754	0.00012	0.000183	0.001654	-0.00051	-0.0002	-0.00034
p__Coprothermobacte	g__unclassified_f__Coprothermobacteraceae	2.04E-05	2.02E-05	0	0	0.002213	0.00693	-3.31E-05	-8.21E-06	-2.04E-05
p__Deferribacteres	g__unclassified_c__Deferribacteres	0.001332	0.0003676	0.0008563	0.000142	0.001706	0.00577	-0.00073	-0.00026	-0.00048
p__Deinococcus-Ther	g__Oceanithermus	0.001544	0.0002708	0.001013	0.00025	0.001008	0.003952	-0.00075	-0.00031	-0.00053
p__Fusobacteria	g__Sebaldella	5.00E-05	1.69E-05	2.46E-05	1.55E-05	0.002827	0.00826	-3.76E-05	-1.19E-05	-2.54E-05
p__Synergistetes	g__unclassified_o__Synergistales	0.0002496	8.64E-05	0.0001092	4.47E-05	0.001315	0.004744	-0.00019	-8.45E-05	-0.00014
p__Synergistetes	g__Acetomicrobium	0.0001274	7.95E-05	3.67E-05	2.20E-05	0.001706	0.00577	-0.00014	-4.40E-05	-9.07E-05
p__Synergistetes	g__Jonquetella	2.99E-05	2.84E-05	3.44E-06	8.25E-06	0.01465	0.03181	-4.46E-05	-8.25E-06	-2.64E-05
p__Thermodesulfobac	g__unclassified_o__Thermodesulfobacteriales	0.1225	0.05433	0.06848	0.02079	0.005795	0.01483	-0.09162	-0.0208	-0.05402
p__Thermotogae	g__Petrotoga	0.001599	0.0005786	0.0006385	0.000182	0.00033	0.002032	-0.00136	-0.00064	-0.00096
p__Thermotogae	g__Kosmotoga	0.0004554	0.0002044	0.0002321	0.000223	0.02113	0.04273	-0.00039	-2.51E-05	-0.00022
p__unclassified_d__B	g__Thermobaculum	0.00672	0.002786	0.004194	0.000686	0.04515	0.08009	-0.00427	-0.00097	-0.00253

p__unclassified_d__B	g__Candidatus_Babela	0.0005785	0.0004089	5.35E-05	4.29E-05	0.000246	0.001813	-0.00077	-0.00031	-0.00053
p__unclassified_d__B	g__Vermiphilus	0.0002988	0.000299	2.82E-05	1.89E-05	0.000183	0.001654	-0.00047	-0.00013	-0.00027
p__unclassified_d__B	g__Candidatus_Chazhemtobacterium	7.60E-05	9.28E-05	2.31E-06	3.89E-06	0.000244	0.001813	-0.00014	-2.64E-05	-7.37E-05

Taxa abundances significantlly decreased under green light when compared to white light

Phylum	Genus	White-Mean (%	White-Sd (%	Green-Mean (%	Green-Sd (%	P value	Corrected pvalu	Lower ci	Upper ci	Effect size
p__Cyanobacteria	g__unclassified_p__Cyanobacteria	10.56	2.995	5.085	2.206	0.000769	0.00392	-7.675	-3.236	-5.479
p__Cyanobacteria	g__Leptolyngbya	1.944	0.5866	1.107	0.431	0.004586	0.0134	-1.245	-0.4157	-0.8371
p__Cyanobacteria	g__unclassified_f__Leptolyngbyace	1.527	0.496	0.7819	0.2909	0.001706	0.006643	-1.078	-0.4118	-0.7454
p__Cyanobacteria	g__Nostoc	0.6852	0.1874	0.4284	0.1507	0.005795	0.01601	-0.3977	-0.1126	-0.2568
p__Cyanobacteria	g__Scytonema	0.4236	0.1233	0.2137	0.08776	0.001008	0.004664	-0.296	-0.1172	-0.2099
p__Cyanobacteria	g__Microcoleus	0.4045	0.1183	0.2002	0.08534	0.000769	0.00392	-0.2916	-0.1214	-0.2043
p__Cyanobacteria	g__unclassified_o__Oscillatoriales	0.4552	0.1466	0.2891	0.1021	0.02575	0.05115	-0.278	-0.06101	-0.1661
p__Cyanobacteria	g__Fischerella	0.2788	0.07572	0.1382	0.05805	0.000769	0.00392	-0.1984	-0.08251	-0.1406
p__Cyanobacteria	g__Calothrix	0.2989	0.09166	0.1626	0.0621	0.002202	0.008006	-0.2021	-0.06844	-0.1363
p__Cyanobacteria	g__Phormidesmis	0.2137	0.06961	0.1051	0.04532	0.001008	0.004664	-0.1598	-0.0623	-0.1086
p__Cyanobacteria	g__Tolypothrix	0.1712	0.0473	0.08859	0.03201	0.000769	0.00392	-0.1205	-0.0508	-0.08258
p__Cyanobacteria	g__Myxacorys	0.08901	0.02301	0.01822	0.007727	0.000183	0.002076	-0.08672	-0.05786	-0.07079
p__Cyanobacteria	g__unclassified_o__Nostocales	0.114	0.03297	0.04949	0.01925	0.000246	0.0022	-0.08805	-0.04414	-0.06456
p__Cyanobacteria	g__Symploca	0.159	0.04573	0.1011	0.04093	0.01726	0.03681	-0.0931	-0.02288	-0.05787
p__Cyanobacteria	g__Hydrococcus_f__Hydrococcace	0.1009	0.02829	0.04689	0.01971	0.000583	0.003312	-0.07555	-0.03175	-0.05406
p__Cyanobacteria	g__Brasilonema	0.1083	0.03576	0.05704	0.02344	0.003611	0.01118	-0.07895	-0.02768	-0.05123
p__Cyanobacteria	g__Cyanothece	0.1515	0.04315	0.1045	0.03202	0.02113	0.04338	-0.07669	-0.0167	-0.04701
p__Cyanobacteria	g__Pseudanabaena	0.1174	0.03413	0.0715	0.02581	0.007285	0.01916	-0.0716	-0.02052	-0.04596
p__Cyanobacteria	g__Moorea	0.09695	0.03166	0.05316	0.01933	0.004586	0.0134	-0.0662	-0.02374	-0.04381
p__Cyanobacteria	g__Chlorogloeopsis	0.0832	0.02523	0.03958	0.01667	0.000769	0.00392	-0.06245	-0.02545	-0.04362
p__Cyanobacteria	g__Desertifilum	0.07024	0.02174	0.03595	0.01379	0.001706	0.006643	-0.05001	-0.01879	-0.0343
p__Cyanobacteria	g__Microcystis	0.08457	0.02619	0.05328	0.01791	0.01402	0.03183	-0.04967	-0.01189	-0.03129
p__Cyanobacteria	g__Pleurocapsa	0.06731	0.02171	0.04012	0.01483	0.01133	0.02695	-0.04174	-0.01116	-0.02719
p__Cyanobacteria	g__Kamptonema	0.05832	0.01667	0.03114	0.0115	0.001008	0.004664	-0.03976	-0.01555	-0.02719
p__Cyanobacteria	g__Nodosilinea	0.05356	0.01398	0.02651	0.01052	0.000583	0.003312	-0.03748	-0.01701	-0.02705
p__Cyanobacteria	g__Thermoleptolyngbya	0.06966	0.02007	0.04282	0.01784	0.01402	0.03183	-0.04209	-0.01132	-0.02684
p__Cyanobacteria	g__Rippkaea	0.02975	0.01239	0.003201	0.0009187	0.000183	0.002076	-0.03403	-0.01936	-0.02654
p__Cyanobacteria	g__Anabaena	0.08383	0.02263	0.05832	0.01794	0.01726	0.03681	-0.0418	-0.00862	-0.0255
p__Cyanobacteria	g__Gloeothece	0.05105	0.02065	0.02605	0.01157	0.007285	0.01916	-0.03962	-0.01097	-0.02501
p__Cyanobacteria	g__Mastigocladopsis	0.04721	0.01707	0.0258	0.009874	0.009108	0.02275	-0.03357	-0.0101	-0.02141
p__Cyanobacteria	g__Gloeocapsopsis	0.04835	0.01254	0.02846	0.01191	0.004586	0.0134	-0.03001	-0.00997	-0.01989
p__Cyanobacteria	g__Cyanosarcina	0.04045	0.01247	0.02112	0.008952	0.002202	0.008006	-0.02848	-0.01062	-0.01933
p__Cyanobacteria	g__Chlorogloea	0.02792	0.007361	0.009985	0.004567	0.000183	0.002076	-0.02327	-0.01284	-0.01794
p__Cyanobacteria	g__Planktothrix	0.0452	0.01629	0.02769	0.0118	0.03121	0.05952	-0.03056	-0.00551	-0.01751
p__Cyanobacteria	g__Chroogloeocystis	0.02197	0.006029	0.006658	0.002809	0.000183	0.002076	-0.01941	-0.01142	-0.01531
p__Cyanobacteria	g__Cylindrospermum	0.03067	0.007173	0.01542	0.005361	0.00033	0.002387	-0.02101	-0.00973	-0.01525
p__Cyanobacteria	g__unclassified_o__Synechococcal	0.02888	0.007991	0.01379	0.005501	0.00044	0.00292	-0.02101	-0.00971	-0.01509
p__Cyanobacteria	g__Stanieria	0.03745	0.01099	0.02239	0.01015	0.01402	0.03183	-0.02393	-0.00557	-0.01506
p__Cyanobacteria	g__unclassified_o__Chroococcales	0.03838	0.01261	0.02383	0.009443	0.01402	0.03183	-0.02379	-0.00455	-0.01455
p__Cyanobacteria	g__Synechococcus	0.04956	0.01043	0.0358	0.006221	0.001706	0.006643	-0.02131	-0.00651	-0.01376
p__Cyanobacteria	g__Synechocystis	0.03005	0.007564	0.01757	0.006442	0.001706	0.006643	-0.01802	-0.00652	-0.01248
p__Cyanobacteria	g__Gloeocapsa	0.03249	0.009597	0.0202	0.007816	0.01726	0.03681	-0.01964	-0.00496	-0.01229
p__Cyanobacteria	g__Coleofasciculus	0.02193	0.004748	0.009716	0.003143	0.000183	0.002076	-0.01554	-0.0086	-0.01221
p__Cyanobacteria	g__Gloeomargarita	0.01803	0.004517	0.006777	0.002798	0.000183	0.002076	-0.01462	-0.00835	-0.01125
p__Cyanobacteria	g__Sphaerospermopsis	0.01997	0.006428	0.008728	0.003331	0.00033	0.002387	-0.01548	-0.0067	-0.01124
p__Cyanobacteria	g__Merismopedia	0.02651	0.009234	0.01543	0.006657	0.01133	0.02695	-0.01721	-0.00484	-0.01108

p__Cyanobacteria	g__Jacksonvillea	0.01779	0.004333	0.006839	0.002436	0.000183	0.002076	-0.01377	-0.00818	-0.01096
p__Cyanobacteria	g__Hyella	0.01806	0.003893	0.007736	0.003048	0.000246	0.0022	-0.01317	-0.00766	-0.01032
p__Cyanobacteria	g__Westiellopsis	0.01196	0.003428	0.001848	0.0007724	0.000183	0.002076	-0.01224	-0.00824	-0.01012
p__Cyanobacteria	g__Rubidibacter	0.01544	0.004694	0.005504	0.002268	0.000183	0.002076	-0.01312	-0.00686	-0.00993
p__Cyanobacteria	g__Nodularia_f__Aphanizomenona	0.02236	0.005748	0.0129	0.00499	0.001315	0.005574	-0.01402	-0.00515	-0.00947
p__Cyanobacteria	g__Fortiea	0.01617	0.004442	0.006741	0.002537	0.000183	0.002076	-0.01272	-0.00657	-0.00943
p__Cyanobacteria	g__Neosynechococcus	0.02557	0.007746	0.01643	0.007139	0.03121	0.05952	-0.01555	-0.00287	-0.00914
p__Cyanobacteria	g__unclassified_f__Acaryochlorida	0.02143	0.006436	0.01301	0.006544	0.01726	0.03681	-0.01413	-0.00319	-0.00842
p__Cyanobacteria	g__unclassified_f__Hapalosiphona	0.01806	0.005301	0.01068	0.004205	0.005795	0.01601	-0.01169	-0.0034	-0.00739
p__Cyanobacteria	g__unclassified_f__Oscillatoriacea	0.007656	0.002195	0.0003943	8.35E-05	0.000183	0.002076	-0.00852	-0.00595	-0.00726
p__Cyanobacteria	g__Crinalium	0.01758	0.005346	0.0108	0.004305	0.01133	0.02695	-0.01083	-0.00299	-0.00678
p__Cyanobacteria	g__Chamaesiphon	0.01713	0.00463	0.01112	0.003696	0.01726	0.03681	-0.00973	-0.00243	-0.00602
p__Cyanobacteria	g__Dolichospermum	0.01038	0.001847	0.004436	0.001206	0.000183	0.002076	-0.00727	-0.00447	-0.00595
p__Cyanobacteria	g__Prochlorothrix	0.00868	0.004497	0.00415	0.002088	0.01402	0.03183	-0.00778	-0.00164	-0.00453
p__Cyanobacteria	g__unclassified_f__Nostocaceae	0.01039	0.004036	0.005944	0.002224	0.02113	0.04338	-0.00725	-0.00171	-0.00445
p__Cyanobacteria	g__Mastigocoleus	0.009812	0.002951	0.005393	0.002056	0.001706	0.006643	-0.00666	-0.00242	-0.00442
p__Cyanobacteria	g__Hapalosiphon	0.01006	0.003254	0.006039	0.002803	0.01726	0.03681	-0.00677	-0.00159	-0.00403
p__Cyanobacteria	g__Anabaenopsis	0.009634	0.004253	0.005781	0.002389	0.02575	0.05115	-0.00652	-0.00098	-0.00386
p__Cyanobacteria	g__unclassified_f__Hyellaceae	0.004989	0.00191	0.001267	0.0006133	0.000183	0.002076	-0.00487	-0.00261	-0.00372
p__Cyanobacteria	g__Roseofilum	0.006524	0.002532	0.002895	0.001271	0.000769	0.00392	-0.00533	-0.00204	-0.00363
p__Cyanobacteria	g__unclassified_f__Microcoleacea	0.00611	0.001532	0.002591	0.001091	0.000246	0.0022	-0.00465	-0.00243	-0.00352
p__Cyanobacteria	g__Myxosarcina	0.007337	0.002002	0.00414	0.001862	0.004586	0.0134	-0.00476	-0.00148	-0.0032
p__Cyanobacteria	g__unclassified_f__Synechococcac	0.007155	0.002444	0.003963	0.0009867	0.001706	0.006643	-0.00491	-0.00149	-0.00319
p__Cyanobacteria	g__Rivularia_f__Rivulariaceae	0.008344	0.002402	0.005175	0.001862	0.007285	0.01916	-0.00493	-0.00142	-0.00317
p__Cyanobacteria	g__Arthrospira	0.006397	0.0024	0.003386	0.001303	0.003611	0.01118	-0.00456	-0.00143	-0.00301
p__Cyanobacteria	g__Geminocystis	0.004217	0.001239	0.001229	0.0003751	0.000183	0.002076	-0.00377	-0.0023	-0.00299
p__Cyanobacteria	g__Cyanobium	0.009043	0.001835	0.006974	0.001284	0.007285	0.01916	-0.00343	-0.00068	-0.00207
p__Cyanobacteria	g__Euhalothece	0.002336	0.0005445	0.0007279	0.0002924	0.000183	0.002076	-0.00199	-0.00125	-0.00161
p__Cyanobacteria	g__Limnothrix	0.005518	0.0009868	0.003913	0.001398	0.01402	0.03183	-0.00254	-0.00048	-0.00161
p__Cyanobacteria	g__Hydrocoleum	0.002585	0.000448	0.001055	0.0003427	0.000183	0.002076	-0.00186	-0.00121	-0.00153
p__Cyanobacteria	g__Snowella	0.001565	0.001034	0.0004717	0.0002639	0.004586	0.0134	-0.00174	-0.00051	-0.00109
p__Cyanobacteria	g__Aphanocapsa	0.001943	0.0004508	0.001078	0.0003376	0.001315	0.005574	-0.00119	-0.00054	-0.00087
p__Cyanobacteria	g__unclassified_f__Spirulinaceae	0.001514	0.0004102	0.0007499	0.0001612	0.000183	0.002076	-0.00104	-0.00052	-0.00076
p__Cyanobacteria	g__Dactylococcopsis	0.001132	0.0003781	0.0006845	0.0002498	0.007285	0.01916	-0.00073	-0.0002	-0.00045
p__Cyanobacteria	g__Microchaete	0.001314	0.0002138	0.0008785	0.0003007	0.002827	0.00937	-0.00065	-0.00021	-0.00044
p__Cyanobacteria	g__unclassified_f__Aphanizomeno	0.0003858	0.0001464	5.23E-06	6.74E-06	0.000163	0.002076	-0.00046	-0.00029	-0.00038
p__Cyanobacteria	g__Cuspidothrix	0.0002446	0.0001525	0.000121	5.47E-05	0.009108	0.02275	-0.00023	-3.81E-05	-0.00012
p__Cyanobacteria	g__Candidatus_Atelocyanobacteriu	9.88E-05	3.90E-05	1.57E-06	3.68E-06	0.000208	0.0022	-0.00012	-7.07E-05	-9.72E-05
p__Cyanobacteria	g__unclassified_f__Chroococcacea	5.33E-05	4.17E-05	0	0	6.39E-05	0.002076	-7.98E-05	-2.95E-05	-5.33E-05
p__Cyanobacteria	g__Chrysosporum	3.01E-05	1.98E-05	2.13E-06	3.03E-06	0.001349	0.005712	-3.95E-05	-1.58E-05	-2.79E-05
p__Cyanobacteria	g__Roholtiella	6.75E-06	1.14E-05	0	0	0.01493	0.03351	-1.37E-05	-1.25E-06	-6.75E-06
p__Proteobacteria	g__Sphingomonas	3.913	0.71	1.086	0.6578	0.000183	0.002076	-3.386	-2.228	-2.826
p__Proteobacteria	g__unclassified_f__Rhodospirillace	5.821	0.939	3.004	1.218	0.000583	0.003312	-3.705	-1.841	-2.817
p__Proteobacteria	g__unclassified_c__Betaproteobact	1.369	0.2263	0.6498	0.1289	0.000183	0.002076	-0.8632	-0.5625	-0.7193
p__Proteobacteria	g__Hyphomicrobium	1.329	0.3437	0.7583	0.184	0.000583	0.003312	-0.8008	-0.3251	-0.5708
p__Proteobacteria	g__unclassified_f__Phyllobacteriac	0.7923	0.2451	0.4503	0.352	0.01402	0.03183	-0.5777	-0.09089	-0.3419
p__Proteobacteria	g__unclassified_c__Alphaproteoba	2.371	0.2236	2.054	0.2837	0.03121	0.05952	-0.5294	-0.08194	-0.3171
p__Proteobacteria	g__Parvularcula	0.4003	0.2418	0.1145	0.05772	0.00033	0.002387	-0.4388	-0.1529	-0.2858

p__Proteobacteria	g__unclassified_f__Comamonadac	0.3602	0.1297	0.08837	0.05452	0.000183	0.002076	-0.3578	-0.198	-0.2718
p__Proteobacteria	g__unclassified_f__Hyphomicrobia	0.5193	0.123	0.2712	0.0918	0.001008	0.004664	-0.3355	-0.1531	-0.2481
p__Proteobacteria	g__Rubrivivax	0.3105	0.1054	0.06922	0.05037	0.000183	0.002076	-0.3122	-0.1749	-0.2413
p__Proteobacteria	g__Aquincola	0.2786	0.1277	0.04116	0.03909	0.000183	0.002076	-0.3225	-0.1643	-0.2375
p__Proteobacteria	g__Sphingopyxis	0.3798	0.05006	0.1746	0.03023	0.000183	0.002076	-0.2389	-0.1716	-0.2051
p__Proteobacteria	g__unclassified_o__Burkholderiale	0.3688	0.09154	0.193	0.104	0.002202	0.008006	-0.2518	-0.08598	-0.1758
p__Proteobacteria	g__Stella	0.2506	0.08668	0.0755	0.01507	0.000183	0.002076	-0.2248	-0.1226	-0.1751
p__Proteobacteria	g__unclassified_o__Rhizobiales	1.206	0.08215	1.036	0.1228	0.003611	0.01118	-0.2582	-0.07824	-0.1707
p__Proteobacteria	g__Piscinibacter	0.2267	0.07613	0.06252	0.04237	0.000246	0.0022	-0.2181	-0.1111	-0.1642
p__Proteobacteria	g__Ideonella	0.218	0.06468	0.0552	0.04099	0.000246	0.0022	-0.2075	-0.1177	-0.1628
p__Proteobacteria	g__Novosphingobium	0.2616	0.04393	0.1063	0.01352	0.000183	0.002076	-0.1826	-0.1276	-0.1553
p__Proteobacteria	g__Azohydromonas	0.1651	0.06505	0.04293	0.03563	0.000583	0.003312	-0.1679	-0.08025	-0.1222
p__Proteobacteria	g__Sphingobium	0.1998	0.02959	0.084	0.0138	0.000183	0.002076	-0.1354	-0.09729	-0.1159
p__Proteobacteria	g__unclassified_o__Sphingomonad	0.2274	0.0272	0.1195	0.03338	0.000246	0.0022	-0.1323	-0.08301	-0.108
p__Proteobacteria	g__Panacagrimonas	0.2002	0.07216	0.09819	0.02817	0.002202	0.008006	-0.1491	-0.05908	-0.102
p__Proteobacteria	g__Arenimonas	0.2047	0.1123	0.1056	0.03879	0.007285	0.01916	-0.1753	-0.03708	-0.09908
p__Proteobacteria	g__Rhizobacter	0.1793	0.05602	0.08468	0.0856	0.003611	0.01118	-0.1492	-0.02928	-0.09466
p__Proteobacteria	g__Sphingorhabdus	0.1456	0.02964	0.05311	0.02127	0.000246	0.0022	-0.1136	-0.07074	-0.09248
p__Proteobacteria	g__Methylibium	0.6906	0.1858	0.5994	0.7912	0.003611	0.01118	-0.45	0.4643	-0.09135
p__Proteobacteria	g__Chakrabartia	0.1211	0.03332	0.03422	0.01126	0.000183	0.002076	-0.1078	-0.06649	-0.08685
p__Proteobacteria	g__unclassified_f__Sphingomonad	0.2537	0.05503	0.1738	0.04356	0.004586	0.0134	-0.1223	-0.03749	-0.07989
p__Proteobacteria	g__Pseudorivibacter	0.1226	0.03789	0.04809	0.04077	0.001706	0.006643	-0.1081	-0.04174	-0.0745
p__Proteobacteria	g__Aquabacterium	0.1096	0.03046	0.04156	0.03351	0.001315	0.005574	-0.09394	-0.04262	-0.06806
p__Proteobacteria	g__Aestuariisphingobium	0.09195	0.02952	0.02981	0.01009	0.000183	0.002076	-0.08147	-0.04425	-0.06214
p__Proteobacteria	g__Bauldia	0.1688	0.02187	0.112	0.04254	0.009108	0.02275	-0.08233	-0.02771	-0.05684
p__Proteobacteria	g__Nordella	0.1452	0.02864	0.09041	0.0323	0.005795	0.01601	-0.07969	-0.02595	-0.05481
p__Proteobacteria	g__Dongia	0.1362	0.0161	0.08268	0.0271	0.001008	0.004664	-0.0706	-0.03533	-0.05354
p__Proteobacteria	g__Altererythrobacter	0.09315	0.01491	0.04136	0.01242	0.000183	0.002076	-0.06193	-0.0399	-0.05179
p__Proteobacteria	g__Filomicrobium	0.07029	0.03025	0.01917	0.004865	0.000183	0.002076	-0.06972	-0.03288	-0.05112
p__Proteobacteria	g__Rivibacter	0.09552	0.02206	0.04823	0.05481	0.002827	0.00937	-0.07513	-0.01022	-0.04728
p__Proteobacteria	g__Ramlibacter	0.09593	0.02766	0.05502	0.03433	0.002827	0.00937	-0.06537	-0.01451	-0.0409
p__Proteobacteria	g__Polaromonas	0.08773	0.02542	0.04795	0.02825	0.002827	0.00937	-0.06155	-0.01484	-0.03978
p__Proteobacteria	g__Hydrogenophaga	0.09053	0.01841	0.05245	0.0209	0.003611	0.01118	-0.05432	-0.01945	-0.03808
p__Proteobacteria	g__Paucibacter	0.05654	0.01883	0.01859	0.01325	0.000769	0.00392	-0.0525	-0.02487	-0.03795
p__Proteobacteria	g__Pelomonas	0.06984	0.02063	0.03256	0.02156	0.005795	0.01601	-0.05582	-0.01944	-0.03728
p__Proteobacteria	g__Erythrobacter	0.07671	0.00967	0.04003	0.01974	0.002202	0.008006	-0.04778	-0.02252	-0.03668
p__Proteobacteria	g__unclassified_f__Burkholderiace	0.1257	0.03581	0.08966	0.07522	0.02575	0.05115	-0.08123	0.02122	-0.03607
p__Proteobacteria	g__Blastomonas	0.04658	0.00698	0.01297	0.002662	0.000183	0.002076	-0.03843	-0.0293	-0.03361
p__Proteobacteria	g__Variovorax	0.2123	0.04163	0.1793	0.1268	0.03121	0.05952	-0.09692	0.04852	-0.03299
p__Proteobacteria	g__Tardibacter	0.04709	0.0219	0.01723	0.005188	0.000246	0.0022	-0.04495	-0.01858	-0.02987
p__Proteobacteria	g__Zhizhongheella	0.0396	0.01242	0.01186	0.01311	0.001315	0.005574	-0.03842	-0.01683	-0.02774
p__Proteobacteria	g__'Geomonas'_Khan_et_al._2020	0.05601	0.02009	0.02847	0.0389	0.003611	0.01118	-0.04949	-0.00082	-0.02756
p__Proteobacteria	g__Hypericibacter	0.1182	0.01154	0.09178	0.01716	0.007285	0.01916	-0.03775	-0.01396	-0.02637
p__Proteobacteria	g__unclassified_f__Sinobacteracea	0.0465	0.03317	0.02379	0.002228	0.001706	0.006643	-0.04514	-0.00596	-0.02271
p__Proteobacteria	g__Porphyrobacter	0.03089	0.004664	0.008833	0.001515	0.000183	0.002076	-0.02514	-0.0194	-0.02206
p__Proteobacteria	g__Leptothrix_r__root	0.03378	0.009285	0.01185	0.0104	0.001315	0.005574	-0.02997	-0.01288	-0.02193
p__Proteobacteria	g__Sandarakinorhabdus	0.03103	0.00531	0.01024	0.001314	0.000183	0.002076	-0.02416	-0.01745	-0.02079
p__Proteobacteria	g__Caenimonas	0.03661	0.009864	0.01596	0.00994	0.001315	0.005574	-0.02886	-0.01299	-0.02065

p__Proteobacteria	g__Parasphingopyxis	0.02435	0.00469	0.003964	0.0005073	0.000183	0.002076	-0.02346	-0.01778	-0.02038
p__Proteobacteria	g__Schlegelella	0.03335	0.009843	0.01305	0.01185	0.001706	0.006643	-0.02895	-0.01078	-0.0203
p__Proteobacteria	g__Rhodoplanes	0.0874	0.01019	0.06942	0.005695	0.000583	0.003312	-0.0251	-0.0113	-0.01798
p__Proteobacteria	g__Nevskia	0.04313	0.02339	0.02589	0.003529	0.009108	0.02275	-0.03301	-0.00563	-0.01724
p__Proteobacteria	g__Rhodomicrobium	0.047	0.01021	0.0299	0.004216	0.000769	0.00392	-0.02322	-0.00999	-0.0171
p__Proteobacteria	g__Acidovorax	0.04408	0.007037	0.02861	0.01473	0.002827	0.00937	-0.02393	-0.00546	-0.01547
p__Proteobacteria	g__Parasphingorhabdus	0.0198	0.003512	0.005274	0.0008284	0.000183	0.002076	-0.01664	-0.01248	-0.01452
p__Proteobacteria	g__Simplicispira	0.01666	0.007179	0.003055	0.002165	0.000183	0.002076	-0.01803	-0.0097	-0.01361
p__Proteobacteria	g__Caldimonas	0.02123	0.007086	0.007977	0.005323	0.001315	0.005574	-0.01853	-0.00776	-0.01325
p__Proteobacteria	g__Pseudorhodoferax	0.02473	0.005979	0.01187	0.006121	0.001706	0.006643	-0.01738	-0.00755	-0.01286
p__Proteobacteria	g__Tatlockia	0.01401	0.006246	0.001302	0.000526	0.000183	0.002076	-0.01597	-0.00878	-0.01271
p__Proteobacteria	g__Curvibacter	0.0185	0.003792	0.008167	0.00367	0.000583	0.003312	-0.01337	-0.00754	-0.01033
p__Proteobacteria	g__unclassified_f__Hyphomonadac	0.04628	0.005268	0.03602	0.008531	0.005795	0.01601	-0.01594	-0.0042	-0.01027
p__Proteobacteria	g__Candidatus_Accumulibacter	0.03614	0.008175	0.02592	0.003625	0.003611	0.01118	-0.01558	-0.00491	-0.01022
p__Proteobacteria	g__Silvanigrella	0.009192	0.005861	0.0001844	8.12E-05	0.000183	0.002076	-0.01275	-0.00599	-0.00901
p__Proteobacteria	g__Aromatoleum	0.02705	0.00336	0.01856	0.00452	0.002827	0.00937	-0.01183	-0.00494	-0.00849
p__Proteobacteria	g__Polycyclovorans	0.01904	0.00638	0.01125	0.002639	0.007285	0.01916	-0.01213	-0.0034	-0.0078
p__Proteobacteria	g__Mitsuaria	0.01484	0.005097	0.007366	0.003254	0.004586	0.0134	-0.01106	-0.00395	-0.00748
p__Proteobacteria	g__unclassified_f__Ectothiorhodos	0.01839	0.007839	0.01108	0.004495	0.03121	0.05952	-0.01259	-0.00226	-0.00731
p__Proteobacteria	g__Phreatobacter	0.03647	0.00369	0.02918	0.002687	0.000583	0.003312	-0.01035	-0.00449	-0.00729
p__Proteobacteria	g__Oceanibaculum	0.03674	0.005047	0.02951	0.004942	0.005795	0.01601	-0.01142	-0.003	-0.00723
p__Proteobacteria	g__Rhizorhabdus	0.01342	0.002383	0.006249	0.001576	0.000183	0.002076	-0.00884	-0.00555	-0.00717
p__Proteobacteria	g__Sphaerotilus	0.01155	0.003047	0.004417	0.00337	0.001315	0.005574	-0.00962	-0.00421	-0.00713
p__Proteobacteria	g__Novosphingopyxis	0.007942	0.001586	0.0009928	0.0002503	0.000183	0.002076	-0.00799	-0.00606	-0.00695
p__Proteobacteria	g__Methylocystis	0.02448	0.002144	0.01784	0.001348	0.000183	0.002076	-0.00821	-0.0051	-0.00664
p__Proteobacteria	g__Ferrovibrio	0.02363	0.003977	0.01702	0.002856	0.001315	0.005574	-0.00949	-0.00388	-0.00661
p__Proteobacteria	g__Magnetospirillum	0.04121	0.00538	0.03487	0.004848	0.02575	0.05115	-0.01081	-0.00165	-0.00635
p__Proteobacteria	g__Ottowia	0.01642	0.005123	0.01021	0.004374	0.003611	0.01118	-0.01019	-0.00232	-0.00622
p__Proteobacteria	g__Azoarcus	0.02725	0.004746	0.02129	0.006657	0.005795	0.01601	-0.01014	-0.00087	-0.00595
p__Proteobacteria	g__unclassified_f__Kiloniellaceae	0.03595	0.0061	0.03	0.004196	0.02113	0.04338	-0.01027	-0.00196	-0.00595
p__Proteobacteria	g__Roseateles	0.01255	0.002514	0.006818	0.005061	0.005795	0.01601	-0.00863	-0.00224	-0.00573
p__Proteobacteria	g__Thiomonas	0.01132	0.002184	0.006008	0.002175	0.00044	0.00292	-0.00702	-0.00353	-0.00531
p__Proteobacteria	g__Eilatimonas	0.011	0.003567	0.005708	0.001094	0.000183	0.002076	-0.00767	-0.00339	-0.00529
p__Proteobacteria	g__Ahniella	0.01418	0.006647	0.008915	0.002864	0.003611	0.01118	-0.01029	-0.0017	-0.00527
p__Proteobacteria	g__Janthinobacterium	0.02055	0.005533	0.01539	0.002169	0.02113	0.04338	-0.00883	-0.00183	-0.00516
p__Proteobacteria	g__Hephaestia	0.01166	0.001482	0.006545	0.001924	0.000583	0.003312	-0.00648	-0.00364	-0.00511
p__Proteobacteria	g__Pacificimonas	0.007048	0.001407	0.002133	0.0008674	0.000183	0.002076	-0.00589	-0.00392	-0.00492
p__Proteobacteria	g__Stakelama	0.006273	0.00112	0.001417	0.0004166	0.000183	0.002076	-0.00554	-0.00416	-0.00486
p__Proteobacteria	g__unclassified_f__Xanthobacterac	0.02571	0.002706	0.02105	0.003385	0.007285	0.01916	-0.00712	-0.00207	-0.00466
p__Proteobacteria	g__Methyloversatilis	0.01194	0.002376	0.007532	0.002433	0.001706	0.006643	-0.00642	-0.00237	-0.00441
p__Proteobacteria	g__Kinneretia	0.00726	0.002294	0.002909	0.002486	0.007285	0.01916	-0.00639	-0.00235	-0.00435
p__Proteobacteria	g__Vogesella	0.00626	0.0004638	0.001999	0.0004391	0.000183	0.002076	-0.00462	-0.00389	-0.00426
p__Proteobacteria	g__Polymorphobacter	0.008027	0.0008523	0.003769	0.0004949	0.000183	0.002076	-0.00482	-0.00365	-0.00426
p__Proteobacteria	g__Wenzhouxiangella	0.02392	0.004732	0.01984	0.003251	0.03764	0.06883	-0.00759	-0.00066	-0.00408
p__Proteobacteria	g__Vitreoscilla	0.005503	0.00176	0.001469	0.001231	0.00033	0.002387	-0.00526	-0.00276	-0.00403
p__Proteobacteria	g__Comamonas	0.01322	0.001825	0.009208	0.003427	0.002827	0.00937	-0.006	-0.00145	-0.00401
p__Proteobacteria	g__unclassified_o__Silvanigrellales	0.003994	0.002622	0.0001185	5.39E-05	0.000183	0.002076	-0.00545	-0.00246	-0.00388
p__Proteobacteria	g__Defluviicoccus	0.02358	0.003635	0.01975	0.003782	0.04515	0.07991	-0.00688	-0.0004	-0.00382

p__Proteobacteria	g__Limnohabitans	0.01167	0.002046	0.007895	0.003515	0.003611	0.01118	-0.0058	-0.00105	-0.00377
p__Proteobacteria	g__Fluviispira	0.003581	0.002261	5.01E-05	4.03E-05	0.000183	0.002076	-0.00499	-0.00227	-0.00353
p__Proteobacteria	g__Sandaracinobacter	0.00595	0.001019	0.00252	0.0005633	0.000183	0.002076	-0.00411	-0.00281	-0.00343
p__Proteobacteria	g__Xenophilus	0.01111	0.001581	0.007717	0.001945	0.001315	0.005574	-0.00505	-0.00192	-0.0034
p__Proteobacteria	g__unclassified_o__Hydrogenophil	0.02168	0.002612	0.0183	0.006173	0.02113	0.04338	-0.00682	0.000931	-0.00339
p__Proteobacteria	g__Thauera	0.01968	0.001952	0.01632	0.002967	0.01402	0.03183	-0.00537	-0.00113	-0.00337
p__Proteobacteria	g__Ensifer	0.03984	0.002739	0.03655	0.004317	0.04515	0.07991	-0.00628	-0.00047	-0.00329
p__Proteobacteria	g__Croceicoccus	0.006806	0.001355	0.003586	0.001089	0.000583	0.003312	-0.0042	-0.00217	-0.00322
p__Proteobacteria	g__Oceanibacterium	0.01472	0.002053	0.01153	0.001805	0.004586	0.0134	-0.00473	-0.00142	-0.0032
p__Proteobacteria	g__Ruegeria	0.01978	0.001503	0.0166	0.001677	0.001315	0.005574	-0.00441	-0.00187	-0.00318
p__Proteobacteria	g__Paracandidimonas	0.003693	0.001546	0.000802	0.0003852	0.000183	0.002076	-0.0039	-0.00197	-0.00289
p__Proteobacteria	g__Rhodoferax	0.03483	0.006934	0.03199	0.03565	0.01726	0.03681	-0.01854	0.02192	-0.00284
p__Proteobacteria	g__Colwellia	0.01005	0.002343	0.007309	0.001002	0.009108	0.02275	-0.00431	-0.00135	-0.00274
p__Proteobacteria	g__Citromicrobium	0.007982	0.00104	0.005253	0.001227	0.000583	0.003312	-0.00367	-0.00177	-0.00273
p__Proteobacteria	g__Methylococcus	0.01069	0.002386	0.007973	0.001785	0.03121	0.05952	-0.00449	-0.00108	-0.00272
p__Proteobacteria	g__Sphingosinithalassobacter	0.00567	0.0008371	0.002977	0.0005498	0.000183	0.002076	-0.00325	-0.0021	-0.00269
p__Proteobacteria	g__Marinicauda	0.008806	0.001298	0.006182	0.001437	0.002202	0.008006	-0.0037	-0.00149	-0.00263
p__Proteobacteria	g__Dechloromonas	0.01201	0.001609	0.00942	0.002063	0.005795	0.01601	-0.00415	-0.00091	-0.00259
p__Proteobacteria	g__Sneathiella	0.01015	0.001184	0.007561	0.0006773	0.000246	0.0022	-0.00346	-0.00181	-0.00259
p__Proteobacteria	g__Rugamonas	0.005311	0.001288	0.002724	0.0006013	0.000183	0.002076	-0.00345	-0.00178	-0.00259
p__Proteobacteria	g__Legionella	0.01409	0.002819	0.01152	0.00182	0.03764	0.06883	-0.00467	-0.00061	-0.00256
p__Proteobacteria	g__Pseudorhodobacter	0.005505	0.001429	0.002969	0.0006377	0.000183	0.002076	-0.00351	-0.00169	-0.00254
p__Proteobacteria	g__Sulfitobacter	0.009395	0.001086	0.006883	0.001068	0.00044	0.00292	-0.00347	-0.00157	-0.00251
p__Proteobacteria	g__Methylomicrobium	0.008514	0.00099	0.006023	0.001453	0.001008	0.004664	-0.00347	-0.00145	-0.00249
p__Proteobacteria	g__Methylosarcina	0.004797	0.0004004	0.002483	0.0005949	0.000183	0.002076	-0.00271	-0.00184	-0.00231
p__Proteobacteria	g__Desulfobacterium	0.005465	0.001521	0.003188	0.0007329	0.00044	0.00292	-0.00338	-0.00134	-0.00228
p__Proteobacteria	g__unclassified_f__Beijerinckiacea	0.01351	0.001275	0.01127	0.001416	0.003611	0.01118	-0.00334	-0.00105	-0.00224
p__Proteobacteria	g__Pusillimonas	0.01112	0.0006489	0.00889	0.00186	0.02113	0.04338	-0.0034	-0.00101	-0.00223
p__Proteobacteria	g__Zoogloea	0.009205	0.0013	0.007176	0.002296	0.007285	0.01916	-0.00346	-0.00028	-0.00203
p__Proteobacteria	g__Xylella	0.00343	0.001631	0.001429	0.0004033	0.002202	0.008006	-0.00308	-0.00102	-0.002
p__Proteobacteria	g__Pseudacidovorax	0.004121	0.0008288	0.002128	0.001114	0.001315	0.005574	-0.00273	-0.00115	-0.00199
p__Proteobacteria	g__Magnetofaba	0.003649	0.0004566	0.001661	0.0008134	0.000583	0.003312	-0.0025	-0.0014	-0.00199
p__Proteobacteria	g__Tepidimonas	0.005551	0.001157	0.003564	0.001104	0.009108	0.02275	-0.00296	-0.00099	-0.00199
p__Proteobacteria	g__Nitrosococcus	0.0149	0.002135	0.01295	0.001356	0.04515	0.07991	-0.00345	-0.00044	-0.00195
p__Proteobacteria	g__Pseudobacteriovorax	0.002866	0.001503	0.0009433	0.0003351	0.00033	0.002387	-0.00284	-0.00114	-0.00192
p__Proteobacteria	g__Aquicella	0.002385	0.001435	0.0004791	0.0003154	0.000246	0.0022	-0.00287	-0.00117	-0.00191
p__Proteobacteria	g__Siccirubricoccus	0.007594	0.0008315	0.005698	0.0008247	0.000246	0.0022	-0.00259	-0.00125	-0.0019
p__Proteobacteria	g__Thiobacillus	0.01461	0.001419	0.01272	0.001998	0.01402	0.03183	-0.00325	-0.00043	-0.00189
p__Proteobacteria	g__Gluconobacter	0.002678	0.0003598	0.0008715	0.0002194	0.000183	0.002076	-0.00209	-0.00156	-0.00181
p__Proteobacteria	g__Glycocaulis	0.006113	0.0009062	0.004333	0.0009851	0.002202	0.008006	-0.00255	-0.00099	-0.00178
p__Proteobacteria	g__Qipengyuania	0.002825	0.0009584	0.001046	0.0006017	0.000583	0.003312	-0.00244	-0.00111	-0.00178
p__Proteobacteria	g__Thiomargarita	0.003887	0.0006842	0.002121	0.0003054	0.000183	0.002076	-0.00225	-0.00132	-0.00177
p__Proteobacteria	g__Methyloceanibacter	0.01301	0.001864	0.01126	0.0006769	0.01726	0.03681	-0.00294	-0.00066	-0.00175
p__Proteobacteria	g__Plasticicumulans	0.01403	0.000939	0.01233	0.001878	0.03121	0.05952	-0.00298	-0.00048	-0.0017
p__Proteobacteria	g__Malikia	0.002642	0.000817	0.000945	0.0003533	0.000183	0.002076	-0.00221	-0.00123	-0.0017
p__Proteobacteria	g__Hylemonella	0.005122	0.001369	0.003455	0.00131	0.009108	0.02275	-0.00278	-0.00053	-0.00167
p__Proteobacteria	g__Croceibacterium	0.002637	0.0005726	0.0009805	0.0002902	0.000183	0.002076	-0.00202	-0.00125	-0.00166
p__Proteobacteria	g__Thiohalobacter	0.004395	0.0005039	0.002772	0.0007455	0.000769	0.00392	-0.00216	-0.00101	-0.00162

p__Proteobacteria	g__unclassified_f__Bacteriovoracac	0.001924	0.0006198	0.0003586	0.0001016	0.000183	0.002076	-0.00196	-0.0012	-0.00157
p__Proteobacteria	g__unclassified_o__Rhodocyclales	0.009911	0.001098	0.008352	0.0008354	0.007285	0.01916	-0.00234	-0.00069	-0.00156
p__Proteobacteria	g__Leisingera	0.003371	0.0004572	0.001875	0.0003412	0.000183	0.002076	-0.00183	-0.00114	-0.0015
p__Proteobacteria	g__Chitinibacter	0.002271	0.0005085	0.0007759	0.000399	0.000183	0.002076	-0.00188	-0.00111	-0.0015
p__Proteobacteria	g__Undibacterium	0.005131	0.0004762	0.00364	0.0005343	0.00044	0.00292	-0.00192	-0.00105	-0.00149
p__Proteobacteria	g__Thalassomonas	0.001978	0.000673	0.0005018	0.0001861	0.000183	0.002076	-0.00192	-0.00106	-0.00148
p__Proteobacteria	g__Acidiphilium	0.006142	0.001247	0.004681	0.001028	0.01726	0.03681	-0.00248	-0.00054	-0.00146
p__Proteobacteria	g__Cohaesibacter	0.003007	0.0005635	0.00155	0.0004768	0.000583	0.003312	-0.00189	-0.001	-0.00146
p__Proteobacteria	g__Zhengella	0.00397	0.001457	0.002514	0.0007027	0.02575	0.05115	-0.00239	-0.00052	-0.00146
p__Proteobacteria	g__Roseibaca	0.001621	0.0003391	0.0001848	7.21E-05	0.000183	0.002076	-0.00163	-0.00124	-0.00144
p__Proteobacteria	g__Boseongicola	0.004039	0.0006711	0.002632	0.0004152	0.000246	0.0022	-0.0019	-0.00096	-0.00141
p__Proteobacteria	g__unclassified_f__Desulfobulbace	0.008793	0.0007407	0.007401	0.0005503	0.000583	0.003312	-0.00193	-0.00087	-0.00139
p__Proteobacteria	g__Alicycliphilus	0.004969	0.00113	0.00358	0.001373	0.02575	0.05115	-0.00238	-0.00033	-0.00139
p__Proteobacteria	g__Propionivibrio	0.005083	0.0004623	0.003733	0.001005	0.01402	0.03183	-0.00197	-0.00063	-0.00135
p__Proteobacteria	g__unclassified_o__Thiotrichales	0.008888	0.001034	0.007554	0.0006723	0.002827	0.00937	-0.00204	-0.00061	-0.00134
p__Proteobacteria	g__Derxia	0.003248	0.0003698	0.001923	0.000279	0.000183	0.002076	-0.00159	-0.00105	-0.00133
p__Proteobacteria	g__Candidatus_Tenderia	0.00389	0.0005985	0.002565	0.0006331	0.001008	0.004664	-0.00182	-0.00078	-0.00133
p__Proteobacteria	g__Cysteiniphilum	0.002763	0.0006758	0.001449	0.0007672	0.002827	0.00937	-0.00191	-0.00071	-0.00131
p__Proteobacteria	g__Oceanicola	0.003486	0.0004859	0.002198	0.0002566	0.000183	0.002076	-0.00161	-0.00097	-0.00129
p__Proteobacteria	g__Chromohalobacter	0.001548	0.0004397	0.0002656	6.00E-05	0.000183	0.002076	-0.00154	-0.00103	-0.00128
p__Proteobacteria	g__unclassified_f__Methylotherma	0.002408	0.0003465	0.001145	0.0002077	0.000183	0.002076	-0.00151	-0.00103	-0.00126
p__Proteobacteria	g__Acidibrevibacterium	0.004517	0.0007998	0.003256	0.0007726	0.003611	0.01118	-0.00183	-0.00056	-0.00126
p__Proteobacteria	g__Escherichia	0.004985	0.000647	0.00375	0.0005852	0.001008	0.004664	-0.00176	-0.00073	-0.00124
p__Proteobacteria	g__unclassified_f__Methylococcac	0.01078	0.001151	0.009576	0.001014	0.04515	0.07991	-0.00211	-0.00024	-0.00121
p__Proteobacteria	g__Oligoflexus	0.004029	0.001574	0.002832	0.0008775	0.03764	0.06883	-0.0023	-0.00016	-0.0012
p__Proteobacteria	g__Spirobacillus	0.001283	0.0006953	0.0001129	5.96E-05	0.000183	0.002076	-0.00161	-0.00079	-0.00117
p__Proteobacteria	g__Oleiphilus	0.003726	0.0005228	0.002562	0.0007724	0.004586	0.0134	-0.00173	-0.00061	-0.00116
p__Proteobacteria	g__Gallionella	0.003967	0.0005604	0.002809	0.0005095	0.001008	0.004664	-0.00156	-0.0007	-0.00116
p__Proteobacteria	g__Granulibacter	0.002356	0.001139	0.001235	0.0004571	0.03121	0.05952	-0.0019	-0.00041	-0.00112
p__Proteobacteria	g__Terasakiella	0.003417	0.0003118	0.002306	0.0003881	0.00033	0.002387	-0.00139	-0.0008	-0.00111
p__Proteobacteria	g__Immundisolibacter	0.005705	0.0006425	0.004598	0.0007738	0.007285	0.01916	-0.0017	-0.00044	-0.00111
p__Proteobacteria	g__Salmonella	0.007998	0.0007567	0.006928	0.0008494	0.007285	0.01916	-0.0018	-0.00039	-0.00107
p__Proteobacteria	g__Shewanella	0.007094	0.0006237	0.006025	0.0007619	0.004586	0.0134	-0.00165	-0.00052	-0.00107
p__Proteobacteria	g__Magnetospira	0.002937	0.000276	0.001905	0.000413	0.00044	0.00292	-0.00131	-0.00074	-0.00103
p__Proteobacteria	g__Pseudomethylobacillus	0.001625	0.0002785	0.0006003	0.0001965	0.000183	0.002076	-0.00123	-0.00081	-0.00103
p__Proteobacteria	g__Zavarzinia	0.01108	0.0009389	0.01006	0.0009231	0.03764	0.06883	-0.00187	-0.00028	-0.00102
p__Proteobacteria	g__Acinetobacter	0.007936	0.0007351	0.006927	0.003448	0.004586	0.0134	-0.00249	0.001727	-0.00101
p__Proteobacteria	g__Marinicella	0.002576	0.0007866	0.001601	0.0005764	0.002827	0.00937	-0.00154	-0.00036	-0.00097
p__Proteobacteria	g__Ahrensia	0.00476	0.0009051	0.003813	0.0007903	0.01133	0.02695	-0.00161	-0.00016	-0.00095
p__Proteobacteria	g__Corallincola	0.001399	0.0002793	0.0004569	0.0002265	0.000183	0.002076	-0.00114	-0.00073	-0.00094
p__Proteobacteria	g__Kofleria	0.001329	0.001026	0.0004062	0.0001031	0.00033	0.002387	-0.00167	-0.00041	-0.00092
p__Proteobacteria	g__Thiocystis	0.002106	0.0008235	0.001201	0.0002102	0.001315	0.005574	-0.00142	-0.0004	-0.00091
p__Proteobacteria	g__Tepidicella	0.001416	0.0003019	0.0005128	0.0002501	0.00033	0.002387	-0.00114	-0.00068	-0.0009
p__Proteobacteria	g__Candidatus_Thiosymbion	0.00346	0.0006386	0.002562	0.0007772	0.02575	0.05115	-0.00146	-0.00023	-0.0009
p__Proteobacteria	g__Methylophaga	0.005936	0.0006528	0.005039	0.0008994	0.02575	0.05115	-0.00158	-0.0002	-0.0009
p__Proteobacteria	g__unclassified_o__Sneathiellales	0.001665	0.0003214	0.0007698	0.0002125	0.000183	0.002076	-0.00112	-0.00068	-0.00089
p__Proteobacteria	g__Maritalea	0.001959	0.0002571	0.001066	0.0002756	0.000246	0.0022	-0.0011	-0.00067	-0.00089
p__Proteobacteria	g__Algiphilus	0.00292	0.001293	0.002034	0.0003263	0.03121	0.05952	-0.00177	-0.00017	-0.00089

p__Proteobacteria	g__Melaminivora	0.001133	0.0003108	0.000267	0.0002058	0.000183	0.002076	-0.00107	-0.00064	-0.00087
p__Proteobacteria	g__unclassified_f__Methylocystace	0.00506	0.0009113	0.004209	0.0003237	0.02575	0.05115	-0.0014	-0.00022	-0.00085
p__Proteobacteria	g__Quisquiliibacterium	0.003816	0.0007769	0.002971	0.001906	0.03764	0.06883	-0.00191	0.000464	-0.00085
p__Proteobacteria	g__Pantoea	0.002199	0.0006147	0.001357	0.0004171	0.002827	0.00937	-0.00126	-0.00038	-0.00084
p__Proteobacteria	g__unclassified_c__Candidatus_La	0.001738	0.0004173	0.0009129	0.0002464	0.000183	0.002076	-0.0011	-0.00056	-0.00083
p__Proteobacteria	g__Crenothrix	0.002541	0.0003646	0.00172	0.0004764	0.003611	0.01118	-0.0012	-0.00046	-0.00082
p__Proteobacteria	g__Methylogaea	0.00193	0.0002646	0.00111	0.000403	0.001315	0.005574	-0.0011	-0.00053	-0.00082
p__Proteobacteria	g__Desulfotignum	0.001941	0.000585	0.001122	0.0004044	0.002827	0.00937	-0.00125	-0.00041	-0.00082
p__Proteobacteria	g__Dissulfurirhabdus	0.001916	0.000402	0.001105	0.000171	0.00033	0.002387	-0.00107	-0.00056	-0.00081
p__Proteobacteria	g__Thioflavicoccus	0.003857	0.0005672	0.003054	0.000722	0.01726	0.03681	-0.00129	-0.00022	-0.0008
p__Proteobacteria	g__Ectothiorhodospira	0.004143	0.0003202	0.003343	0.0005291	0.002827	0.00937	-0.00116	-0.00042	-0.0008
p__Proteobacteria	g__Alkalispirillum	0.001056	0.0004825	0.0002575	0.0001015	0.000183	0.002076	-0.00112	-0.00053	-0.0008
p__Proteobacteria	g__Marinovum	0.001766	0.0004361	0.0009759	0.0002818	0.000583	0.003312	-0.0011	-0.0005	-0.00079
p__Proteobacteria	g__Oceanimonas	0.001164	0.0004023	0.0003776	7.32E-05	0.000183	0.002076	-0.00105	-0.00058	-0.00079
p__Proteobacteria	g__unclassified_f__Chromatiaceae	0.00405	0.0007739	0.003279	0.0006203	0.03121	0.05952	-0.00134	-0.00016	-0.00077
p__Proteobacteria	g__Azovibrio	0.00215	0.0003396	0.001399	0.0002909	0.00044	0.00292	-0.00103	-0.0005	-0.00075
p__Proteobacteria	g__Denitratisoma	0.002919	0.0005951	0.002175	0.0005726	0.02575	0.05115	-0.00124	-0.00022	-0.00074
p__Proteobacteria	g__Rickettsia	0.001279	0.000275	0.0005424	0.0001168	0.000183	0.002076	-0.00091	-0.00057	-0.00074
p__Proteobacteria	g__Candidatus_Methylospira	0.001391	0.0002447	0.0006632	0.0001044	0.000183	0.002076	-0.00089	-0.00058	-0.00073
p__Proteobacteria	g__Rhodospira	0.001873	0.0001839	0.001146	0.0002186	0.000183	0.002076	-0.0009	-0.00057	-0.00073
p__Proteobacteria	g__unclassified_f__Pseudomonada	0.001761	0.0003418	0.001043	0.0003525	0.001706	0.006643	-0.00101	-0.00043	-0.00072
p__Proteobacteria	g__Rugosibacter	0.002886	0.0006747	0.002175	0.000392	0.01402	0.03183	-0.00122	-0.00028	-0.00071
p__Proteobacteria	g__Minwuia	0.002722	0.0002567	0.002057	0.0003462	0.00033	0.002387	-0.00096	-0.00043	-0.00067
p__Proteobacteria	g__Candidatus_Marithrix	0.001309	0.0004201	0.0006575	0.0003059	0.002202	0.008006	-0.00098	-0.00036	-0.00065
p__Proteobacteria	g__Roseospira	0.004113	0.0003976	0.00347	0.0006468	0.03121	0.05952	-0.00109	-0.00015	-0.00064
p__Proteobacteria	g__Casimicrobium	0.002778	0.000489	0.002137	0.0003122	0.003611	0.01118	-0.00098	-0.00032	-0.00064
p__Proteobacteria	g__Thiolapillus	0.002062	0.000514	0.001426	0.0002557	0.004586	0.0134	-0.00102	-0.0003	-0.00064
p__Proteobacteria	g__Xylophilus	0.003601	0.0008305	0.002966	0.002643	0.01133	0.02695	-0.00196	0.001107	-0.00063
p__Proteobacteria	g__Hankyongella	0.001338	0.0002774	0.0007041	8.22E-05	0.000183	0.002076	-0.00081	-0.00046	-0.00063
p__Proteobacteria	g__Oligotropha	0.002299	0.0002175	0.001666	0.0002729	0.00033	0.002387	-0.00086	-0.00042	-0.00063
p__Proteobacteria	g__Luminiphilus	0.001948	0.0004185	0.001316	0.0004523	0.009108	0.02275	-0.001	-0.0003	-0.00063
p__Proteobacteria	g__Cucumibacter	0.001711	0.0001399	0.00108	0.00018	0.000183	0.002076	-0.00076	-0.00051	-0.00063
p__Proteobacteria	g__Sulfurirhabdus	0.002391	0.0003268	0.001767	0.0002485	0.000769	0.00392	-0.00089	-0.00037	-0.00062
p__Proteobacteria	g__Granulosicoccus	0.001284	0.0004327	0.0006703	9.84E-05	0.000183	0.002076	-0.00091	-0.00039	-0.00061
p__Proteobacteria	g__Candidatus_Magnetomorum	0.001756	0.0005097	0.001156	0.0003423	0.009108	0.02275	-0.001	-0.00026	-0.0006
p__Proteobacteria	g__Planktomarina	0.0008089	0.0003634	0.0002168	0.0001292	0.000246	0.0022	-0.00085	-0.00038	-0.00059
p__Proteobacteria	g__Iodobacter	0.000999	0.0003299	0.0004124	0.0001578	0.000583	0.003312	-0.0008	-0.00038	-0.00059
p__Proteobacteria	g__Smithella	0.002799	0.000398	0.002214	0.0006452	0.02113	0.04338	-0.00103	-0.00012	-0.00059
p__Proteobacteria	g__unclassified_f__Acidiferrobacte	0.004034	0.0005515	0.00345	0.0004443	0.03121	0.05952	-0.00103	-0.00016	-0.00058
p__Proteobacteria	g__Photorhabdus	0.001516	0.0004315	0.0009363	0.000227	0.002827	0.00937	-0.00089	-0.00031	-0.00058
p__Proteobacteria	g__Aquidulcibacter	0.001577	0.0003634	0.001002	0.0003054	0.004586	0.0134	-0.00087	-0.0003	-0.00058
p__Proteobacteria	g__Celeribacter	0.002063	0.0003168	0.001492	0.0002837	0.002827	0.00937	-0.00079	-0.00029	-0.00057
p__Proteobacteria	g__unclassified_o__Oligoflexales	0.0007139	0.0002277	0.0001429	4.12E-05	0.000183	0.002076	-0.00072	-0.00044	-0.00057
p__Proteobacteria	g__Arhodomonas	0.002313	0.000337	0.001764	0.0003238	0.005795	0.01601	-0.00081	-0.00025	-0.00055
p__Proteobacteria	g__Maribius	0.001361	0.0004057	0.000814	0.0002546	0.002827	0.00937	-0.00086	-0.00027	-0.00055
p__Proteobacteria	g__unclassified_o__Legionellales	0.001135	0.0002957	0.0005897	0.0001524	0.000183	0.002076	-0.00076	-0.00037	-0.00055
p__Proteobacteria	g__Mariprofundus	0.001734	0.0007075	0.001189	0.0001491	0.04515	0.07991	-0.00103	-0.00015	-0.00055
p__Proteobacteria	g__Amylibacter	0.00157	0.0003338	0.00103	0.0002459	0.002202	0.008006	-0.00078	-0.0003	-0.00054

p__Proteobacteria	g__Alcaligenes	0.001428	0.0003091	0.0008938	0.0003313	0.009108	0.02275	-0.00079	-0.00027	-0.00053
p__Proteobacteria	g__Serpentinomonas	0.0007988	0.0002155	0.0002673	0.0001718	0.00044	0.00292	-0.00069	-0.00035	-0.00053
p__Proteobacteria	g__Halobacteriovorax	0.001432	0.0005811	0.0009028	0.0002275	0.02113	0.04338	-0.0009	-0.00019	-0.00053
p__Proteobacteria	g__Endozoicomonas	0.001921	0.0003426	0.001392	0.0001309	0.001315	0.005574	-0.00074	-0.00029	-0.00053
p__Proteobacteria	g__Lonsdalea	0.0006992	0.0003911	0.0001704	9.63E-05	0.00044	0.00292	-0.00077	-0.0003	-0.00053
p__Proteobacteria	g__Chitinivorax	0.002336	0.0004655	0.001827	0.0002491	0.01726	0.03681	-0.00079	-0.00019	-0.00051
p__Proteobacteria	g__Maritimibacter	0.003915	0.0002314	0.003415	0.0005296	0.04515	0.07991	-0.00086	-0.00019	-0.0005
p__Proteobacteria	g__Cronobacter	0.001019	0.0005762	0.0005315	0.0001705	0.03121	0.05952	-0.00087	-0.00014	-0.00049
p__Proteobacteria	g__Yoonia	0.001707	0.0002959	0.001226	0.0003146	0.003611	0.01118	-0.00075	-0.00023	-0.00048
p__Proteobacteria	g__Erythromicrobium	0.002447	0.0003489	0.001971	0.0005373	0.03764	0.06883	-0.00088	-9.27E-05	-0.00048
p__Proteobacteria	g__Salaquimonas	0.001888	0.0005601	0.001412	0.001111	0.01402	0.03183	-0.00116	0.000407	-0.00048
p__Proteobacteria	g__Pelagicola	0.0009261	0.0003635	0.0004511	9.89E-05	0.000583	0.003312	-0.00068	-0.00026	-0.00048
p__Proteobacteria	g__Thermochromatium	0.0008389	0.000267	0.0003646	0.0001212	0.000183	0.002076	-0.00066	-0.00031	-0.00047
p__Proteobacteria	g__Sulfurovum	0.0006296	0.0001503	0.0001587	5.87E-05	0.000183	0.002076	-0.00057	-0.00037	-0.00047
p__Proteobacteria	g__Hahella	0.003519	0.0004055	0.00305	0.0004035	0.03121	0.05952	-0.0008	-0.00013	-0.00047
p__Proteobacteria	g__Dichotomicrobium	0.001558	0.000398	0.001105	0.0001511	0.002202	0.008006	-0.00071	-0.00021	-0.00045
p__Proteobacteria	g__Candidatus_Thioglobus	0.0009392	0.0001907	0.0004921	0.0001294	0.00033	0.002387	-0.00058	-0.00031	-0.00045
p__Proteobacteria	g__Kangiella	0.001804	0.0003581	0.001359	0.0003105	0.01726	0.03681	-0.00072	-0.00017	-0.00044
p__Proteobacteria	g__Epibacterium	0.001279	0.0004327	0.0008486	0.0002389	0.02113	0.04338	-0.00072	-0.00014	-0.00043
p__Proteobacteria	g__Candidatus_Magnetaquicoccus	0.0005895	0.0004083	0.0001629	4.79E-05	0.000183	0.002076	-0.00069	-0.0002	-0.00043
p__Proteobacteria	g__Herminiimonas	0.002346	0.0003649	0.001921	0.0008343	0.02575	0.05115	-0.00089	0.000218	-0.00043
p__Proteobacteria	g__Sulfurimonas	0.000785	0.0004623	0.0003668	0.0001197	0.005795	0.01601	-0.00071	-0.00017	-0.00042
p__Proteobacteria	g__Candidatus_Fonsibacter	0.0006632	0.0002109	0.0002672	0.000219	0.002202	0.008006	-0.00056	-0.00021	-0.0004
p__Proteobacteria	g__Brucella	0.003041	0.0002827	0.002656	0.0002965	0.01726	0.03681	-0.00066	-0.00016	-0.00039
p__Proteobacteria	g__Azonexus	0.00162	0.0002329	0.001235	0.0003393	0.01402	0.03183	-0.00061	-0.00012	-0.00039
p__Proteobacteria	g__Saezia	0.0005214	0.0001135	0.0001397	0.0001066	0.000246	0.0022	-0.00048	-0.00029	-0.00038
p__Proteobacteria	g__Pseudodesulfovibrio	0.001405	0.0002307	0.001024	0.0001916	0.002202	0.008006	-0.00055	-0.0002	-0.00038
p__Proteobacteria	g__Pelagibaca	0.0007533	0.0002044	0.0003768	7.78E-05	0.000183	0.002076	-0.0005	-0.00024	-0.00038
p__Proteobacteria	g__Chitinilyticum	0.0009274	0.0002442	0.0005624	0.0001852	0.004586	0.0134	-0.00054	-0.00018	-0.00037
p__Proteobacteria	g__Candidatus_Electronema	0.0007416	0.0003823	0.0003805	0.000136	0.005795	0.01601	-0.00061	-0.00013	-0.00036
p__Proteobacteria	g__Catenovulum	0.000604	0.0001845	0.0002489	0.0001035	0.00044	0.00292	-0.00048	-0.00022	-0.00036
p__Proteobacteria	g__unclassified_o__Nevskiales	0.001045	0.0003845	0.0006938	0.0002051	0.02113	0.04338	-0.00062	-0.00012	-0.00035
p__Proteobacteria	g__Acetobacter	0.00454	0.0004055	0.0042	0.0003134	0.03121	0.05952	-0.00065	-5.35E-05	-0.00034
p__Proteobacteria	g__Guyparkeria	0.0003916	9.45E-05	5.48E-05	3.52E-05	0.000183	0.002076	-0.0004	-0.00028	-0.00034
p__Proteobacteria	g__unclassified_f__Neisseriaceae	0.0009627	0.0003056	0.0006291	0.0001994	0.01402	0.03183	-0.00056	-0.00012	-0.00033
p__Proteobacteria	g__Magnetococcus	0.0008886	0.0001771	0.0005661	0.000187	0.002827	0.00937	-0.00047	-0.00017	-0.00032
p__Proteobacteria	g__Geopsychrobacter	0.0007502	0.0003344	0.0004304	0.0001579	0.02575	0.05115	-0.00054	-0.00013	-0.00032
p__Proteobacteria	g__Gulbenkiania	0.001094	0.0002052	0.0007795	0.0002876	0.01726	0.03681	-0.0005	-9.82E-05	-0.00031
p__Proteobacteria	g__unclassified_f__Fastidiosibacter	0.0004818	0.0003502	0.0001709	8.19E-05	0.004586	0.0134	-0.00054	-0.0001	-0.00031
p__Proteobacteria	g__Desulfomicrobium	0.001351	0.0001381	0.001046	0.0001906	0.001706	0.006643	-0.00045	-0.00018	-0.00031
p__Proteobacteria	g__Coxiella_f__Coxiellaceae	0.000879	0.0002915	0.0005792	0.0001762	0.005795	0.01601	-0.00051	-0.0001	-0.0003
p__Proteobacteria	g__Aerosticca	0.001041	0.0002785	0.0007416	0.0002426	0.01133	0.02695	-0.00051	-7.53E-05	-0.0003
p__Proteobacteria	g__unclassified_f__Cellvibrionacea	0.0007436	0.0003402	0.0004486	8.63E-05	0.009108	0.02275	-0.00052	-0.00012	-0.0003
p__Proteobacteria	g__Hydrogenovibrio	0.0005471	0.0002345	0.0002534	6.16E-05	0.005795	0.01601	-0.00043	-0.00015	-0.00029
p__Proteobacteria	g__Novispirillum	0.001485	0.0001766	0.001192	0.0001709	0.004586	0.0134	-0.00045	-0.00015	-0.00029
p__Proteobacteria	g__Aidingimonas	0.00101	0.000274	0.000725	0.0001199	0.02113	0.04338	-0.00047	-0.00012	-0.00028
p__Proteobacteria	g__Ferriphaselus	0.0008846	9.09E-05	0.000603	0.0001617	0.000583	0.003312	-0.00039	-0.00018	-0.00028
p__Proteobacteria	g__Candidatus_Magnetoglobus	0.0003865	0.0002452	0.0001085	4.40E-05	0.00044	0.00292	-0.00043	-0.00014	-0.00028

p__Proteobacteria	g__Fastidiosibacter	0.0003873	8.95E-05	0.0001156	8.30E-05	0.00033	0.002387	-0.00034	-0.0002	-0.00027
p__Proteobacteria	g__Tolumonas	0.0003956	0.0001673	0.0001244	4.51E-05	0.000583	0.003312	-0.00038	-0.00018	-0.00027
p__Proteobacteria	g__Nioella	0.000893	0.0002094	0.0006251	0.0001989	0.01726	0.03681	-0.00043	-8.25E-05	-0.00027
p__Proteobacteria	g__Brachymonas	0.001301	0.0002883	0.001034	0.0001819	0.03121	0.05952	-0.00048	-6.56E-05	-0.00027
p__Proteobacteria	g__Bartonella	0.0006438	0.0002149	0.0003798	0.0001378	0.004586	0.0134	-0.00041	-0.00011	-0.00026
p__Proteobacteria	g__Desulfohalovibrio	0.0008968	0.0002297	0.0006351	0.0001336	0.02113	0.04338	-0.00042	-0.0001	-0.00026
p__Proteobacteria	g__Cycloclasticus	0.0006413	0.0001425	0.0003959	9.38E-05	0.002827	0.00937	-0.00034	-0.00014	-0.00025
p__Proteobacteria	g__Peredibacter	0.0002747	0.0001461	4.26E-05	4.21E-05	0.000246	0.0022	-0.00032	-0.00014	-0.00023
p__Proteobacteria	g__Amphritea	0.0008549	0.0001642	0.0006235	0.0002005	0.01133	0.02695	-0.0004	-7.50E-05	-0.00023
p__Proteobacteria	g__Candidatus_Symbiobacter	0.0003256	0.0001465	0.0001034	5.18E-05	0.00033	0.002387	-0.00032	-0.00013	-0.00022
p__Proteobacteria	g__Thalassococcus	0.0009562	0.0001329	0.0007349	0.0001818	0.02575	0.05115	-0.00034	-9.11E-05	-0.00022
p__Proteobacteria	g__Hydrogenophilus	0.0004162	0.0001438	0.0001963	7.57E-05	0.001315	0.005574	-0.00031	-0.00013	-0.00022
p__Proteobacteria	g__Neomegalonema	0.0006007	0.0001864	0.0003812	0.0001553	0.02113	0.04338	-0.00036	-8.92E-05	-0.00022
p__Proteobacteria	g__Desulfonauticus	0.0004738	0.0001601	0.0002567	9.39E-05	0.002827	0.00937	-0.00034	-0.00011	-0.00022
p__Proteobacteria	g__unclassified_f__Pasteurellaceae	0.0002811	0.0001463	6.79E-05	3.83E-05	0.000246	0.0022	-0.00031	-0.00013	-0.00021
p__Proteobacteria	g__Providencia	0.0006133	0.0001075	0.0004015	0.0001265	0.002827	0.00937	-0.00032	-0.00012	-0.00021
p__Proteobacteria	g__Salinihabitans	0.000772	0.0001625	0.0005616	0.0001513	0.01402	0.03183	-0.00034	-7.90E-05	-0.00021
p__Proteobacteria	g__Methylocucumis	0.0008391	0.0001076	0.000631	9.83E-05	0.002202	0.008006	-0.0003	-0.00012	-0.00021
p__Proteobacteria	g__Microvirgula	0.0004635	0.0001746	0.000267	0.0001152	0.02575	0.05115	-0.00032	-8.34E-05	-0.0002
p__Proteobacteria	g__Sinobacterium	0.0006601	0.000148	0.0004759	0.0001634	0.01402	0.03183	-0.00032	-5.26E-05	-0.00018
p__Proteobacteria	g__unclassified_o__Enterobacterale	0.0008177	0.0001666	0.0006351	0.0001775	0.03121	0.05952	-0.00032	-2.86E-05	-0.00018
p__Proteobacteria	g__Desulfocapsa	0.0005172	0.0001957	0.000337	0.0001287	0.01402	0.03183	-0.00032	-4.85E-05	-0.00018
p__Proteobacteria	g__Profundibacterium	0.0002175	9.52E-05	4.22E-05	3.55E-05	0.000246	0.0022	-0.00024	-0.00012	-0.00018
p__Proteobacteria	g__Stenoxybacter	0.0002692	1.00E-04	9.53E-05	3.99E-05	0.00033	0.002387	-0.00024	-0.00011	-0.00017
p__Proteobacteria	g__Chitinolyticbacter	0.0006684	0.0001825	0.0005043	0.000155	0.03764	0.06883	-0.0003	-1.54E-05	-0.00016
p__Proteobacteria	g__Desulfotalea	0.0002527	0.0001972	8.97E-05	5.93E-05	0.03764	0.06883	-0.00028	-4.48E-05	-0.00016
p__Proteobacteria	g__Wolbachia	0.0002702	0.0001057	0.0001095	5.03E-05	0.001008	0.004664	-0.00023	-9.33E-05	-0.00016
p__Proteobacteria	g__Dissulfuribacter	0.000296	0.0001819	0.0001359	6.41E-05	0.005795	0.01601	-0.00028	-4.86E-05	-0.00016
p__Proteobacteria	g__Fluoribacter	0.0003295	9.78E-05	0.0001706	6.72E-05	0.001706	0.006643	-0.00023	-8.74E-05	-0.00016
p__Proteobacteria	g__Biostraticola	0.0002704	9.08E-05	0.0001125	7.72E-05	0.002827	0.00937	-0.00023	-9.18E-05	-0.00016
p__Proteobacteria	g__unclassified_f__Brucellaceae	0.0002158	8.55E-05	6.28E-05	3.68E-05	0.000583	0.003312	-0.00021	-0.0001	-0.00015
p__Proteobacteria	g__Arsukibacterium	0.0002195	0.0002053	6.84E-05	5.75E-05	0.03764	0.06883	-0.0003	-3.52E-05	-0.00015
p__Proteobacteria	g__Sutterella	0.0002635	8.68E-05	0.0001182	4.53E-05	0.000769	0.00392	-0.0002	-8.70E-05	-0.00015
p__Proteobacteria	g__Oryzisolibacter	0.0002064	8.67E-05	7.00E-05	7.40E-05	0.003611	0.01118	-0.0002	-6.84E-05	-0.00014
p__Proteobacteria	g__Grimontia	0.0005552	0.0001777	0.0004214	0.0001721	0.04515	0.07991	-0.0003	1.26E-05	-0.00013
p__Proteobacteria	g__Lawsonia_f__Desulfovibrionac	0.0001639	9.84E-05	3.23E-05	2.13E-05	0.00033	0.002387	-0.0002	-7.73E-05	-0.00013
p__Proteobacteria	g__Aliishimia	0.0001507	8.65E-05	2.23E-05	2.12E-05	0.000246	0.0022	-0.00018	-8.02E-05	-0.00013
p__Proteobacteria	g__Tritonibacter	0.0002269	0.0001163	0.0001041	3.97E-05	0.009108	0.02275	-0.0002	-5.48E-05	-0.00012
p__Proteobacteria	g__Lentibacter	0.0001788	0.0002057	5.70E-05	3.19E-05	0.02113	0.04338	-0.00025	-2.92E-05	-0.00012
p__Proteobacteria	g__Candidatus_Methylopumilus	0.0002257	0.000133	0.0001058	2.81E-05	0.001008	0.004664	-0.00021	-5.14E-05	-0.00012
p__Proteobacteria	g__Kingella	0.0002855	9.00E-05	0.0001683	6.90E-05	0.01133	0.02695	-0.00018	-4.83E-05	-0.00012
p__Proteobacteria	g__Extensimonas	0.0008833	0.0002015	0.0007684	0.0005795	0.03121	0.05952	-0.0004	0.000315	-0.00012
p__Proteobacteria	g__Salinimonas	0.0001913	0.000105	8.02E-05	4.97E-05	0.01726	0.03681	-0.00018	-3.83E-05	-0.00011
p__Proteobacteria	g__Parvibium	0.000198	0.0001025	9.96E-05	3.08E-05	0.002827	0.00937	-0.00017	-4.06E-05	-9.85E-05
p__Proteobacteria	g__unclassified_f__Sutterellaceae	0.0001565	9.68E-05	6.04E-05	2.44E-05	0.003611	0.01118	-0.00016	-4.22E-05	-9.61E-05
p__Proteobacteria	g__unclassified_f__Desulfarculacea	0.0001211	0.0001068	2.81E-05	2.88E-05	0.03121	0.05952	-0.00015	-3.02E-05	-9.30E-05
p__Proteobacteria	g__Rodentibacter	0.0001102	3.38E-05	2.22E-05	1.50E-05	0.000183	0.002076	-0.00011	-6.68E-05	-8.80E-05
p__Proteobacteria	g__Bergeriella_f__Neisseriaceae	9.42E-05	8.18E-05	7.11E-06	6.23E-06	0.003934	0.01214	-0.00014	-4.31E-05	-8.71E-05

p__Proteobacteria	g__Corticimicrobacter	0.0001384	5.33E-05	5.18E-05	3.89E-05	0.001315	0.005574	-0.00013	-4.83E-05	-8.66E-05
p__Proteobacteria	g__Frischella	8.93E-05	4.25E-05	4.22E-06	6.09E-06	0.000149	0.002076	-0.00011	-6.12E-05	-8.51E-05
p__Proteobacteria	g__Gallibacterium	0.000105	8.18E-05	2.75E-05	2.54E-05	0.005795	0.01601	-0.00013	-3.28E-05	-7.75E-05
p__Proteobacteria	g__unclassified_f__Erythrobacterac	0.0008345	9.65E-05	0.00076	0.0007957	0.005795	0.01601	-0.00039	0.00044	-7.45E-05
p__Proteobacteria	g__Neptunicoccus	0.0001285	5.22E-05	5.56E-05	1.97E-05	0.000183	0.002076	-0.00011	-4.26E-05	-7.29E-05
p__Proteobacteria	g__Pulveribacter	0.0001445	5.39E-05	7.34E-05	4.04E-05	0.01133	0.02695	-0.00011	-3.14E-05	-7.11E-05
p__Proteobacteria	g__Candidatus_Hamiltonella	7.09E-05	9.98E-05	0	0	0.000231	0.0022	-0.00013	-1.72E-05	-7.09E-05
p__Proteobacteria	g__Nitratiruptor	9.97E-05	5.51E-05	3.06E-05	1.72E-05	0.003611	0.01118	-0.0001	-3.49E-05	-6.91E-05
p__Proteobacteria	g__Youngimonas	0.0001893	8.87E-05	0.0001208	5.48E-05	0.03121	0.05952	-0.00013	-1.01E-05	-6.85E-05
p__Proteobacteria	g__Thiofilum	0.0002092	5.65E-05	0.000141	5.51E-05	0.02575	0.05115	-0.00012	-2.17E-05	-6.82E-05
p__Proteobacteria	g__Caedimonas	0.0001018	3.20E-05	3.50E-05	3.28E-05	0.001706	0.006643	-9.04E-05	-3.96E-05	-6.67E-05
p__Proteobacteria	g__Ignatzschineria	7.05E-05	5.72E-05	4.00E-06	1.14E-05	0.00038	0.002739	-9.94E-05	-3.39E-05	-6.65E-05
p__Proteobacteria	g__Thalassorhabdomicrobium	0.0001055	5.02E-05	4.36E-05	3.06E-05	0.005795	0.01601	-9.66E-05	-2.91E-05	-6.19E-05
p__Proteobacteria	g__Modicisalibacter	0.0001237	4.08E-05	6.36E-05	2.67E-05	0.003611	0.01118	-8.88E-05	-3.20E-05	-6.02E-05
p__Proteobacteria	g__Thiovulum	0.0001093	7.95E-05	5.34E-05	3.82E-05	0.03121	0.05952	-0.00011	-9.14E-06	-5.59E-05
p__Proteobacteria	g__Atlantibacter	8.48E-05	3.33E-05	3.10E-05	2.40E-05	0.001315	0.005574	-7.80E-05	-2.88E-05	-5.38E-05
p__Proteobacteria	g__unclassified_f__Chromobacteria	5.22E-05	3.38E-05	5.30E-06	6.25E-06	0.000233	0.0022	-6.94E-05	-3.09E-05	-4.69E-05
p__Proteobacteria	g__Suttonella	5.80E-05	4.02E-05	1.22E-05	8.27E-06	0.004586	0.0134	-6.96E-05	-2.13E-05	-4.58E-05
p__Proteobacteria	g__Aliivibrio	0.0001264	4.63E-05	8.14E-05	1.68E-05	0.009108	0.02275	-7.44E-05	-1.65E-05	-4.50E-05
p__Proteobacteria	g__Gilliamella	0.000147	2.94E-05	0.0001028	0.0001322	0.01402	0.03183	-0.00011	4.41E-05	-4.42E-05
p__Proteobacteria	g__Desulfohalobium	7.08E-05	3.74E-05	2.67E-05	2.54E-05	0.007263	0.01916	-7.22E-05	-1.91E-05	-4.41E-05
p__Proteobacteria	g__Thaumasiovibrio	0.000145	4.33E-05	0.0001015	7.24E-05	0.01726	0.03681	-8.97E-05	1.08E-05	-4.36E-05
p__Proteobacteria	g__Bibersteinia	4.24E-05	3.98E-05	0	0	6.39E-05	0.002076	-6.80E-05	-2.02E-05	-4.24E-05
p__Proteobacteria	g__Taylorella	6.65E-05	5.99E-05	2.73E-05	1.57E-05	0.02575	0.05115	-7.72E-05	-5.60E-06	-3.91E-05
p__Proteobacteria	g__Nissabacter	7.74E-05	3.61E-05	3.96E-05	1.98E-05	0.01133	0.02695	-6.34E-05	-1.41E-05	-3.78E-05
p__Proteobacteria	g__unclassified_f__Coxiellaceae	5.17E-05	2.43E-05	1.53E-05	1.50E-05	0.001315	0.005574	-5.27E-05	-2.00E-05	-3.64E-05
p__Proteobacteria	g__Entomomonas	4.81E-05	4.51E-05	1.28E-05	1.17E-05	0.02329	0.04778	-6.48E-05	-1.06E-05	-3.53E-05
p__Proteobacteria	g__Desulfonatronospira	5.99E-05	2.22E-05	2.49E-05	2.42E-05	0.007285	0.01916	-5.29E-05	-1.52E-05	-3.50E-05
p__Proteobacteria	g__Ventosimonas	5.72E-05	2.73E-05	2.25E-05	1.85E-05	0.004571	0.0134	-5.50E-05	-1.60E-05	-3.47E-05
p__Proteobacteria	g__Candidatus_Finniella	7.80E-05	2.94E-05	4.49E-05	2.50E-05	0.03121	0.05952	-5.82E-05	-9.57E-06	-3.31E-05
p__Proteobacteria	g__Hydrogenimonas	5.47E-05	5.15E-05	2.28E-05	1.28E-05	0.03764	0.06883	-6.70E-05	-5.52E-06	-3.19E-05
p__Proteobacteria	g__unclassified_f__Holosporaceae	3.39E-05	3.32E-05	3.01E-06	8.14E-06	0.00051	0.003312	-5.24E-05	-1.23E-05	-3.09E-05
p__Proteobacteria	g__Nitratifractor	3.85E-05	2.72E-05	1.01E-05	6.93E-06	0.002827	0.00937	-4.78E-05	-1.27E-05	-2.83E-05
p__Proteobacteria	g__Aggregatibacter	0.0001032	2.82E-05	7.52E-05	4.32E-05	0.04515	0.07991	-5.80E-05	4.78E-06	-2.81E-05
p__Proteobacteria	g__Sulfurivirga	4.54E-05	2.81E-05	1.76E-05	1.62E-05	0.01402	0.03183	-4.74E-05	-9.14E-06	-2.78E-05
p__Proteobacteria	g__Pseudospirillum	3.08E-05	1.45E-05	5.97E-06	8.45E-06	0.001207	0.005564	-3.47E-05	-1.47E-05	-2.48E-05
p__Proteobacteria	g__Formivibrio	0.0003315	0.000129	0.0003119	0.0005716	0.007285	0.01916	-0.00025	0.000383	-1.96E-05
p__Proteobacteria	g__unclassified_f__Mariprofundac	3.43E-05	2.20E-05	1.66E-05	1.82E-05	0.01133	0.02695	-3.48E-05	-6.45E-07	-1.77E-05
p__Proteobacteria	g__Nereida	1.80E-05	1.50E-05	3.69E-06	1.03E-05	0.004901	0.0143	-2.47E-05	-2.91E-06	-1.43E-05
p__Proteobacteria	g__Halarcobacter	1.85E-05	1.79E-05	4.35E-06	5.32E-06	0.02631	0.05223	-2.68E-05	-3.81E-06	-1.42E-05
p__Proteobacteria	g__Wolinella	1.42E-05	1.10E-05	8.72E-07	1.84E-06	0.002455	0.008855	-1.95E-05	-6.31E-06	-1.33E-05
p__Proteobacteria	g__Pseudoarcobacter	1.83E-05	1.58E-05	5.54E-06	1.09E-05	0.01913	0.04073	-2.40E-05	-1.64E-06	-1.28E-05
p__Proteobacteria	g__Necropsobacter	1.24E-05	1.82E-05	0	0	0.002213	0.008006	-2.38E-05	-4.03E-06	-1.24E-05
p__Proteobacteria	g__Aliiarcobacter	1.24E-05	1.39E-05	0	0	0.002213	0.008006	-2.09E-05	-4.85E-06	-1.24E-05
p__Proteobacteria	g__Candidatus_Phycorickettsia	1.20E-05	1.60E-05	4.84E-07	1.53E-06	0.01472	0.03332	-2.16E-05	-3.61E-06	-1.15E-05
p__Proteobacteria	g__Thorsellia	1.13E-05	1.81E-05	0	0	0.002213	0.008006	-2.40E-05	-3.17E-06	-1.13E-05
p__Proteobacteria	g__Halopeptonella	1.71E-05	8.76E-06	7.04E-06	1.36E-05	0.01278	0.03032	-1.91E-05	8.57E-07	-1.01E-05
p__Proteobacteria	g__Celerinatantimonas	1.12E-05	1.23E-05	1.14E-06	2.55E-06	0.01161	0.02758	-1.76E-05	-3.13E-06	-1.00E-05

p__Proteobacteria	g__Caedibacter	1.16E-05	7.58E-06	1.77E-06	3.24E-06	0.001784	0.006938	-1.49E-05	-5.27E-06	-9.84E-06
p__Proteobacteria	g__Frederiksenia	9.51E-06	1.13E-05	4.08E-07	1.29E-06	0.002526	0.009092	-1.67E-05	-3.53E-06	-9.10E-06
p__Proteobacteria	g__Candidatus_Aquarickettsia	9.08E-06	1.09E-05	0	0	0.01493	0.03351	-1.55E-05	-2.61E-06	-9.08E-06
p__Proteobacteria	g__unclassified_f__Endozoicomon	1.06E-05	1.02E-05	1.84E-06	2.40E-06	0.03571	0.06723	-1.47E-05	-2.87E-06	-8.81E-06
p__Proteobacteria	g__Trabulsiella	1.06E-05	7.62E-06	2.31E-06	7.31E-06	0.003641	0.01126	-1.39E-05	-2.08E-06	-8.26E-06
p__Proteobacteria	g__Phocoenobacter	7.32E-06	1.18E-05	0	0	0.01493	0.03351	-1.48E-05	-1.73E-06	-7.32E-06
p__Proteobacteria	g__Candidatus_Neoehrlichia	9.37E-06	6.73E-06	2.66E-06	5.63E-06	0.01939	0.04123	-1.18E-05	-1.18E-06	-6.70E-06
p__Proteobacteria	g__Vespertiliibacter	5.84E-06	1.22E-05	0	0	0.03498	0.06597	-1.37E-05	-4.35E-07	-5.84E-06
p__Proteobacteria	g__Yokenella	5.48E-06	1.06E-05	0	0	0.03498	0.06597	-1.24E-05	-8.62E-07	-5.48E-06
p__Proteobacteria	g__Chelonobacter	3.70E-06	6.05E-06	0	0	0.03498	0.06597	-7.74E-06	-3.74E-07	-3.70E-06
p__Planctomycetes	g__unclassified_f__Lacipirellulacea	0.2902	0.04607	0.1127	0.04597	0.000183	0.002076	-0.2137	-0.1378	-0.1774
p__Planctomycetes	g__Lacipirellula	0.1994	0.03116	0.08155	0.03084	0.000183	0.002076	-0.1417	-0.08973	-0.1179
p__Planctomycetes	g__Pirellula	0.1572	0.03552	0.04583	0.02029	0.000183	0.002076	-0.1347	-0.08643	-0.1114
p__Planctomycetes	g__unclassified_f__Planctomycetac	0.4877	0.05455	0.3967	0.06802	0.003611	0.01118	-0.1427	-0.03792	-0.09099
p__Planctomycetes	g__unclassified_c__Planctomycetia	0.1492	0.02575	0.1064	0.02369	0.003611	0.01118	-0.06371	-0.02269	-0.04279
p__Planctomycetes	g__Rhodopirellula	0.08513	0.01138	0.04506	0.00631	0.000183	0.002076	-0.04756	-0.03287	-0.04006
p__Planctomycetes	g__Schlesneria	0.04174	0.007889	0.02153	0.001983	0.000183	0.002076	-0.02498	-0.01565	-0.02021
p__Planctomycetes	g__Planctomicrobium	0.02625	0.008425	0.008762	0.0009882	0.000183	0.002076	-0.02253	-0.01306	-0.01749
p__Planctomycetes	g__unclassified_f__Pirellulaceae	0.03189	0.00591	0.01836	0.003796	0.000246	0.0022	-0.01785	-0.00921	-0.01353
p__Planctomycetes	g__Candidatus_Anammoximicrobi	0.02947	0.004713	0.01745	0.002402	0.000183	0.002076	-0.01528	-0.00905	-0.01202
p__Planctomycetes	g__Blastopirellula	0.05017	0.007296	0.03819	0.007512	0.001706	0.006643	-0.01831	-0.0065	-0.01198
p__Planctomycetes	g__Rubinisphaera	0.01563	0.002134	0.01057	0.001305	0.000183	0.002076	-0.0066	-0.00374	-0.00506
p__Planctomycetes	g__Candidatus_Brocadia	0.01854	0.003506	0.01357	0.002419	0.002827	0.00937	-0.00737	-0.00241	-0.00497
p__Planctomycetes	g__Roseimaritima	0.01202	0.001565	0.008239	0.0008471	0.000183	0.002076	-0.0048	-0.0028	-0.00379
p__Planctomycetes	g__Gimesia	0.009531	0.001013	0.006209	0.0008024	0.000183	0.002076	-0.00415	-0.00257	-0.00332
p__Planctomycetes	g__Fuerstia_f__Planctomycetaceae	0.01154	0.00171	0.008304	0.001236	0.000583	0.003312	-0.0045	-0.00201	-0.00323
p__Planctomycetes	g__Bremerella	0.005935	0.0007176	0.003458	0.0008359	0.000183	0.002076	-0.00316	-0.00181	-0.00248
p__Planctomycetes	g__Planctopirus	0.006766	0.001111	0.004694	0.000706	0.000183	0.002076	-0.00297	-0.00142	-0.00207
p__Planctomycetes	g__Thermogutta	0.005111	0.0007142	0.003443	0.0006331	0.000183	0.002076	-0.0022	-0.00113	-0.00167
p__Planctomycetes	g__Crateriforma	0.00132	0.0003664	0.0005433	8.93E-05	0.000183	0.002076	-0.00101	-0.00056	-0.00078
p__Planctomycetes	g__Alienimonas	0.003453	0.0006028	0.00272	0.0003609	0.009108	0.02275	-0.00113	-0.00033	-0.00073
p__Planctomycetes	g__Mariniblastus	0.001616	0.0001804	0.001249	0.0002054	0.001315	0.005574	-0.00052	-0.00021	-0.00037
p__Bacteroidetes	g__unclassified_f__Flavobacteriace	0.08324	0.06161	0.01846	0.02806	0.001315	0.005574	-0.1043	-0.02641	-0.06478
p__Bacteroidetes	g__Flavobacterium	0.1638	0.0436	0.09911	0.02794	0.004586	0.0134	-0.09366	-0.03113	-0.06467
p__Bacteroidetes	g__Chryseotalea	0.03056	0.01836	0.01502	0.008792	0.02113	0.04338	-0.02768	-0.00364	-0.01554
p__Bacteroidetes	g__Muricauda	0.01571	0.01034	0.005744	0.0017	0.002827	0.00937	-0.01699	-0.00417	-0.00996
p__Bacteroidetes	g__Fulvivirga	0.02423	0.007891	0.01505	0.00801	0.01402	0.03183	-0.01579	-0.00272	-0.00918
p__Bacteroidetes	g__Maribacter	0.007204	0.00493	0.002348	0.001141	0.002827	0.00937	-0.00808	-0.00224	-0.00486
p__Bacteroidetes	g__Arenibacter	0.005673	0.003859	0.00172	0.0007168	0.001706	0.006643	-0.00639	-0.00197	-0.00395
p__Bacteroidetes	g__Imperialibacter	0.007927	0.003589	0.004845	0.002267	0.04515	0.07991	-0.0056	-0.0007	-0.00308
p__Bacteroidetes	g__Pricia	0.003383	0.002018	0.0006398	0.0001932	0.000183	0.002076	-0.00398	-0.00165	-0.00274
p__Bacteroidetes	g__Flagellimonas	0.002776	0.001648	0.0004073	0.0001481	0.000183	0.002076	-0.00334	-0.00143	-0.00237
p__Bacteroidetes	g__Zeaxanthinibacter	0.002198	0.00167	0.0001162	7.72E-05	0.000183	0.002076	-0.0031	-0.00121	-0.00208
p__Bacteroidetes	g__Eudoraea	0.002173	0.001369	0.0004452	0.0002214	0.00044	0.00292	-0.00266	-0.00089	-0.00173
p__Bacteroidetes	g__Robiginitalea	0.001704	0.001333	0.0001397	8.53E-05	0.000183	0.002076	-0.00238	-0.00086	-0.00157
p__Bacteroidetes	g__Kriegella	0.001524	0.0009031	0.0001566	9.07E-05	0.000183	0.002076	-0.00195	-0.00089	-0.00137
p__Bacteroidetes	g__Ulvibacterium	0.002392	0.001498	0.001106	0.0005186	0.01133	0.02695	-0.00225	-0.00043	-0.00129
p__Bacteroidetes	g__Muriicola	0.001437	0.0008162	0.0001846	0.0001286	0.000183	0.002076	-0.00173	-0.00083	-0.00125

p__Bacteroidetes	g__Paludibacter	0.002437	0.000779	0.001233	0.0006451	0.003611	0.01118	-0.00184	-0.00063	-0.00121
p__Bacteroidetes	g__Aquiflexum	0.001826	0.0007388	0.0009426	0.0005881	0.003611	0.01118	-0.00146	-0.00034	-0.00088
p__Bacteroidetes	g__Winogradskyella	0.001958	0.000496	0.001203	0.0005376	0.007285	0.01916	-0.00119	-0.00033	-0.00075
p__Bacteroidetes	g__Salegentibacter	0.000978	0.0005513	0.0003795	7.97E-05	0.001008	0.004664	-0.00094	-0.0003	-0.0006
p__Bacteroidetes	g__Saonia	0.0007133	0.0005106	0.0001163	5.32E-05	0.000183	0.002076	-0.00093	-0.00032	-0.0006
p__Bacteroidetes	g__Pseudozobellia	0.0005962	0.0004262	4.61E-05	2.68E-05	0.000183	0.002076	-0.00082	-0.00032	-0.00055
p__Bacteroidetes	g__Proteiniphilum	0.0009726	0.000503	0.0004654	0.0002176	0.01726	0.03681	-0.00084	-0.0002	-0.00051
p__Bacteroidetes	g__Algibacter	0.001061	0.0003594	0.0005597	0.00016	0.001008	0.004664	-0.00074	-0.00028	-0.0005
p__Bacteroidetes	g__Salinimicrobium	0.0006551	0.000395	0.0001732	6.17E-05	0.00033	0.002387	-0.00075	-0.00027	-0.00048
p__Bacteroidetes	g__Breznakibacter	0.0005641	0.0002442	0.0001195	2.79E-05	0.000183	0.002076	-0.00059	-0.0003	-0.00044
p__Bacteroidetes	g__Antarcticibacterium	0.0006355	0.0001749	0.0002156	0.0001259	0.00044	0.00292	-0.00054	-0.0003	-0.00042
p__Bacteroidetes	g__Sediminicola	0.0005031	0.0003777	0.0001007	5.47E-05	0.001706	0.006643	-0.00065	-0.0002	-0.0004
p__Bacteroidetes	g__Croceitalea	0.0004212	0.0002933	2.20E-05	2.33E-05	0.000183	0.002076	-0.00059	-0.00023	-0.0004
p__Bacteroidetes	g__unclassified_f__Microscillaceae	0.001175	0.0003573	0.0007818	0.0002865	0.01726	0.03681	-0.00065	-9.85E-05	-0.00039
p__Bacteroidetes	g__Robertkochia	0.0004775	0.0003372	0.0001235	5.74E-05	0.001315	0.005574	-0.00056	-0.00017	-0.00035
p__Bacteroidetes	g__Belliella	0.0004855	0.0002734	0.0001376	6.73E-05	0.000246	0.0022	-0.00054	-0.0002	-0.00035
p__Bacteroidetes	g__Seonamhaeicola	0.0006376	0.000457	0.0003155	0.0002009	0.03121	0.05952	-0.00065	-4.85E-05	-0.00032
p__Bacteroidetes	g__Anditalea	0.0004013	0.0002201	0.0001298	6.75E-05	0.000769	0.00392	-0.00041	-0.00015	-0.00027
p__Bacteroidetes	g__Flavivirga	0.0006564	0.0002364	0.0003879	0.0002126	0.01726	0.03681	-0.00047	-8.64E-05	-0.00027
p__Bacteroidetes	g__Crocinitomix	0.0004433	0.0004103	0.000186	0.0001668	0.02575	0.05115	-0.00053	-2.17E-05	-0.00026
p__Bacteroidetes	g__Euzebyella	0.0002843	0.0002396	3.30E-05	2.29E-05	0.00033	0.002387	-0.00041	-0.00012	-0.00025
p__Bacteroidetes	g__Marixanthomonas	0.0002624	9.92E-05	2.45E-05	2.01E-05	0.000183	0.002076	-0.00029	-0.00018	-0.00024
p__Bacteroidetes	g__Prolixibacter	0.0005359	0.0002836	0.000337	0.0001151	0.04515	0.07991	-0.00039	-3.26E-05	-0.0002
p__Bacteroidetes	g__Salisaeta	0.0004601	0.0001549	0.0002726	0.0001313	0.009108	0.02275	-0.00031	-6.52E-05	-0.00019
p__Bacteroidetes	g__Acetobacteroides	0.0002661	0.0002215	7.88E-05	3.69E-05	0.01133	0.02695	-0.00033	-6.62E-05	-0.00019
p__Bacteroidetes	g__Tangfeifania	0.0002424	0.0001835	5.63E-05	2.70E-05	0.000583	0.003312	-0.00029	-8.37E-05	-0.00019
p__Bacteroidetes	g__Capnocytophaga	0.0004121	0.0001782	0.0002458	9.55E-05	0.02575	0.05115	-0.0003	-4.22E-05	-0.00017
p__Bacteroidetes	g__Mesonia	0.0002328	0.0001502	8.75E-05	3.99E-05	0.01402	0.03183	-0.00025	-5.78E-05	-0.00015
p__Bacteroidetes	g__Lunatimonas	0.0002431	0.0001024	9.78E-05	5.80E-05	0.002827	0.00937	-0.00021	-7.98E-05	-0.00015
p__Bacteroidetes	g__Persicobacter	0.0002502	0.0001808	0.0001052	6.38E-05	0.01133	0.02695	-0.00026	-4.58E-05	-0.00015
p__Bacteroidetes	g__Altibacter	0.0001515	5.86E-05	2.24E-05	1.17E-05	0.000183	0.002076	-0.00016	-9.40E-05	-0.00013
p__Bacteroidetes	g__Owenweeksia	0.0002562	0.0001542	0.0001292	7.64E-05	0.009108	0.02275	-0.00025	-2.32E-05	-0.00013
p__Bacteroidetes	g__Urechidicola	0.0001362	7.10E-05	1.22E-05	1.11E-05	0.000179	0.002076	-0.00017	-8.40E-05	-0.00012
p__Bacteroidetes	g__Arenitalea	0.000357	0.000141	0.0002381	0.0001246	0.03764	0.06883	-0.00023	-2.73E-06	-0.00012
p__Bacteroidetes	g__Schleiferia	0.0001286	9.69E-05	1.43E-05	1.50E-05	0.000583	0.003312	-0.00018	-5.97E-05	-0.00011
p__Bacteroidetes	g__Yeosuana	0.0001238	8.19E-05	1.11E-05	1.03E-05	0.000241	0.0022	-0.00017	-6.97E-05	-0.00011
p__Bacteroidetes	g__Williamwhitmania	0.0001666	5.00E-05	5.88E-05	3.37E-05	0.00033	0.002387	-0.00014	-7.47E-05	-0.00011
p__Bacteroidetes	g__Imtechella	0.0001041	8.01E-05	0	0	6.39E-05	0.002076	-0.00016	-6.42E-05	-0.0001
p__Bacteroidetes	g__Vicingus	0.0001634	9.28E-05	6.47E-05	8.55E-05	0.01133	0.02695	-0.00017	-2.43E-05	-9.86E-05
p__Bacteroidetes	g__Galbibacter	0.0001066	7.11E-05	1.25E-05	1.61E-05	0.000323	0.002387	-0.00014	-5.36E-05	-9.41E-05
p__Bacteroidetes	g__Litoribacter	0.0002089	5.12E-05	0.000117	4.31E-05	0.001706	0.006643	-0.00013	-5.48E-05	-9.19E-05
p__Bacteroidetes	g__Croceibacter	0.0001472	9.02E-05	5.56E-05	3.11E-05	0.009108	0.02275	-0.00015	-3.80E-05	-9.16E-05
p__Bacteroidetes	g__Algoriella	9.52E-05	6.34E-05	3.93E-06	5.79E-06	0.001029	0.004747	-0.00013	-5.49E-05	-9.13E-05
p__Bacteroidetes	g__Bernardetia	0.0001034	0.0001007	1.54E-05	1.89E-05	0.003485	0.01118	-0.00015	-3.34E-05	-8.80E-05
p__Bacteroidetes	g__Oceanihabitans	0.0001007	4.58E-05	2.26E-05	2.23E-05	0.000583	0.003312	-0.00011	-5.05E-05	-7.81E-05
p__Bacteroidetes	g__Hyunsoonleella	0.0001185	3.81E-05	4.25E-05	3.33E-05	0.000583	0.003312	-0.00011	-4.45E-05	-7.60E-05
p__Bacteroidetes	g__Zhouia	9.24E-05	5.02E-05	1.76E-05	1.27E-05	0.000183	0.002076	-0.00011	-4.51E-05	-7.49E-05
p__Bacteroidetes	g__Aquirufa	0.0001195	6.54E-05	4.73E-05	3.15E-05	0.004586	0.0134	-0.00012	-3.03E-05	-7.23E-05

p__Bacteroidetes	g__Croceivirga	0.0001105	8.89E-05	4.17E-05	2.22E-05	0.02575	0.05115	-0.00013	-1.96E-05	-6.88E-05
p__Bacteroidetes	g__Indibacter	0.0001005	9.15E-05	4.44E-05	5.43E-05	0.02575	0.05115	-0.00012	4.26E-06	-5.62E-05
p__Bacteroidetes	g__Spongiivirga	6.85E-05	4.76E-05	1.35E-05	1.53E-05	0.000765	0.00392	-8.68E-05	-2.94E-05	-5.50E-05
p__Bacteroidetes	g__Hugenholtzia	9.16E-05	3.09E-05	3.74E-05	1.83E-05	0.001008	0.004664	-7.50E-05	-3.39E-05	-5.43E-05
p__Bacteroidetes	g__Alkaliflexus	6.78E-05	3.91E-05	1.91E-05	1.93E-05	0.004525	0.0134	-7.76E-05	-2.44E-05	-4.88E-05
p__Bacteroidetes	g__Natronoflexus	6.72E-05	2.52E-05	1.96E-05	1.38E-05	0.000769	0.00392	-6.45E-05	-3.03E-05	-4.75E-05
p__Bacteroidetes	g__Subsaximicrobium	7.00E-05	2.41E-05	2.52E-05	1.76E-05	0.001008	0.004664	-6.15E-05	-2.72E-05	-4.49E-05
p__Bacteroidetes	g__Mucinivorans	4.21E-05	4.27E-05	0	0	6.39E-05	0.002076	-6.98E-05	-2.09E-05	-4.21E-05
p__Bacteroidetes	g__Thermophagus	4.55E-05	2.17E-05	9.46E-06	9.34E-06	0.00058	0.003312	-4.95E-05	-2.34E-05	-3.61E-05
p__Bacteroidetes	g__Microbacter	3.75E-05	2.73E-05	6.31E-06	1.00E-05	0.003184	0.0105	-4.75E-05	-1.44E-05	-3.12E-05
p__Bacteroidetes	g__unclassified_f__Lentimicrobiac	4.77E-05	3.58E-05	1.72E-05	1.78E-05	0.03115	0.05952	-5.25E-05	-8.37E-06	-3.05E-05
p__Bacteroidetes	g__Pustulibacterium	3.71E-05	3.04E-05	7.95E-06	1.20E-05	0.005434	0.01582	-5.02E-05	-1.12E-05	-2.92E-05
p__Bacteroidetes	g__Mesoflavibacter	6.75E-05	4.12E-05	4.34E-05	1.68E-05	0.03764	0.06883	-5.27E-05	-2.60E-06	-2.41E-05
p__Bacteroidetes	g__Ochrovirga	2.40E-05	1.90E-05	1.52E-06	4.82E-06	0.000693	0.003919	-3.39E-05	-1.21E-05	-2.25E-05
p__Bacteroidetes	g__Cruoricaptor	2.09E-05	1.78E-05	0	0	0.000751	0.00392	-3.19E-05	-1.11E-05	-2.09E-05
p__Bacteroidetes	g__Rikenella	2.31E-05	2.35E-05	2.85E-06	9.02E-06	0.001005	0.004664	-3.71E-05	-6.54E-06	-2.02E-05
p__Bacteroidetes	g__Jejuia	2.60E-05	2.04E-05	7.93E-06	9.14E-06	0.02995	0.05925	-3.11E-05	-6.13E-06	-1.81E-05
p__Bacteroidetes	g__Cloacibacterium	1.85E-05	1.84E-05	1.69E-06	2.85E-06	0.001364	0.005769	-2.87E-05	-7.42E-06	-1.68E-05
p__Bacteroidetes	g__Sanguibacteroides	1.80E-05	5.96E-06	1.51E-06	4.76E-06	0.000229	0.0022	-2.07E-05	-1.18E-05	-1.65E-05
p__Bacteroidetes	g__unclassified_f__Prevotellaceae	1.93E-05	2.92E-05	3.58E-06	7.45E-06	0.009958	0.02482	-3.52E-05	-2.25E-06	-1.57E-05
p__Bacteroidetes	g__Bergeyella	1.47E-05	1.82E-05	1.86E-06	3.94E-06	0.04496	0.07991	-2.45E-05	-2.89E-06	-1.28E-05
p__Bacteroidetes	g__Luteibaculum	3.01E-05	2.91E-05	1.80E-05	3.16E-05	0.03096	0.05952	-3.70E-05	1.41E-05	-1.21E-05
p__Bacteroidetes	g__unclassified_f__Dysgonomonad	8.42E-06	7.17E-06	3.05E-06	9.64E-06	0.01161	0.02758	-1.17E-05	2.27E-06	-5.38E-06
p__Bacteroidetes	g__Blattabacterium	4.69E-06	6.20E-06	0	0	0.01493	0.03351	-8.87E-06	-1.44E-06	-4.69E-06
p__Acidobacteria	g__unclassified_f__Bryobacteracea	0.2188	0.1102	0.1121	0.0325	0.007285	0.01916	-0.1793	-0.03953	-0.1067
p__Acidobacteria	g__unclassified_c__Thermoanaerol	0.1082	0.04274	0.06532	0.0439	0.002827	0.00937	-0.07584	-0.00564	-0.0429
p__Acidobacteria	g__unclassified_o__Holophagales	0.009742	0.003129	0.005895	0.001161	0.000583	0.003312	-0.00589	-0.0021	-0.00385
p__Acidobacteria	g__Holophaga	0.001563	0.0003598	0.0007866	0.0002971	0.000583	0.003312	-0.00108	-0.0005	-0.00078
p__Acidobacteria	g__Thermoanaerobaculum	0.001513	0.0004811	0.0008765	0.0003115	0.007285	0.01916	-0.00097	-0.00031	-0.00064
p__Actinobacteria	g__Ilumatobacter	0.1428	0.04044	0.04656	0.01595	0.000183	0.002076	-0.1253	-0.07083	-0.09624
p__Actinobacteria	g__Agromyces	0.07906	0.1094	0.01132	0.002235	0.002827	0.00937	-0.1376	-0.01171	-0.06773
p__Actinobacteria	g__unclassified_f__Acidimicrobiac	0.09608	0.02163	0.05393	0.00732	0.00033	0.002387	-0.0555	-0.02827	-0.04214
p__Actinobacteria	g__Humibacillus	0.002262	0.0004976	0.00134	0.0003159	0.001008	0.004664	-0.00128	-0.00059	-0.00092
p__Actinobacteria	g__Agrococcus	0.002156	0.0006694	0.001385	0.0003542	0.002827	0.00937	-0.00125	-0.00037	-0.00077
p__Actinobacteria	g__Mycetocola	0.001528	0.0004689	0.0009565	0.0003723	0.007285	0.01916	-0.00093	-0.00022	-0.00057
p__Actinobacteria	g__Actinomyces	0.004447	0.0004882	0.004046	0.001707	0.02575	0.05115	-0.00132	0.00085	-0.0004
p__Actinobacteria	g__Collinsella	0.0009171	0.0001736	0.0006627	0.0001095	0.005795	0.01601	-0.00037	-0.00013	-0.00025
p__Actinobacteria	g__Acidithrix	0.0004477	0.0001151	0.0002701	0.0001143	0.004586	0.0134	-0.00027	-8.06E-05	-0.00018
p__Actinobacteria	g__Citricoccus	0.0003164	5.02E-05	0.000151	4.91E-05	0.000183	0.002076	-0.00021	-0.00012	-0.00017
p__Actinobacteria	g__Ornithinibacter	0.0003447	0.0001033	0.0002057	7.52E-05	0.009108	0.02275	-0.00022	-6.68E-05	-0.00014
p__Actinobacteria	g__Sediminihabitans	0.000292	0.0001151	0.0001541	5.78E-05	0.01726	0.03681	-0.00022	-6.79E-05	-0.00014
p__Actinobacteria	g__Barrientosiimonas	0.0002879	0.0001426	0.0001621	4.31E-05	0.02113	0.04338	-0.00022	-4.38E-05	-0.00013
p__Actinobacteria	g__unclassified_f__Kineosporiacea	0.0003933	0.0001182	0.0002678	0.0001331	0.03121	0.05952	-0.00023	-1.59E-05	-0.00013
p__Actinobacteria	g__Millisia	0.0002306	3.71E-05	0.0001411	3.59E-05	0.00033	0.002387	-0.00012	-6.04E-05	-8.95E-05
p__Actinobacteria	g__unclassified_f__Coriobacteriace	0.0002031	7.27E-05	0.0001237	4.54E-05	0.005795	0.01601	-0.00013	-2.83E-05	-7.95E-05
p__Actinobacteria	g__unclassified_o__Candidatus_Na	9.82E-05	3.02E-05	2.12E-05	1.55E-05	0.000183	0.002076	-9.69E-05	-5.75E-05	-7.70E-05
p__Actinobacteria	g__Rothia_f__Micrococcaceae	0.0001401	9.27E-05	7.52E-05	2.85E-05	0.01402	0.03183	-0.00013	-1.58E-05	-6.50E-05
p__Actinobacteria	g__Xylanimicrobium	8.71E-05	3.61E-05	2.81E-05	1.85E-05	0.000769	0.00392	-8.34E-05	-3.64E-05	-5.90E-05

p__Actinobacteria	g__Propionimicrobium	7.65E-05	4.60E-05	2.01E-05	2.06E-05	0.002194	0.008006	-8.63E-05	-2.63E-05	-5.63E-05
p__Actinobacteria	g__Yonghaparkia	9.08E-05	4.99E-05	4.08E-05	2.02E-05	0.04515	0.07991	-8.39E-05	-1.91E-05	-5.00E-05
p__Actinobacteria	g__Trueperella	5.05E-05	5.08E-05	1.11E-05	1.90E-05	0.01643	0.0368	-7.05E-05	-9.58E-06	-3.94E-05
p__Actinobacteria	g__Arcanobacterium	6.58E-05	3.66E-05	2.70E-05	1.81E-05	0.02113	0.04338	-6.22E-05	-1.56E-05	-3.88E-05
p__Actinobacteria	g__Parvibacter	2.73E-05	4.41E-05	5.08E-07	1.61E-06	0.002526	0.009092	-5.72E-05	-6.33E-06	-2.67E-05
p__Actinobacteria	g__Adlercreutzia	2.05E-05	1.48E-05	6.58E-06	1.35E-05	0.01207	0.02864	-2.46E-05	-1.35E-06	-1.39E-05
p__Actinobacteria	g__Ancrocorticia	4.96E-06	8.49E-06	0	0	0.03498	0.06597	-1.02E-05	-7.69E-07	-4.96E-06
p__Actinobacteria	g__Gleimia	4.83E-06	7.21E-06	0	0	0.01493	0.03351	-9.76E-06	-1.44E-06	-4.83E-06
p__Nitrospinae	g__unclassified_f__Nitrospinaceae	0.0007067	0.0001814	0.0004396	9.99E-05	0.009108	0.02275	-0.00039	-0.00015	-0.00027
p__Nitrospirae	g__unclassified_f__Nitrospiraceae	0.09813	0.02548	0.04726	0.02013	0.001008	0.004664	-0.06951	-0.03109	-0.05087
p__Nitrospirae	g__unclassified_o__Nitrospirales	0.003378	0.0006784	0.002503	0.0003704	0.007285	0.01916	-0.00133	-0.00045	-0.00087
p__Nitrospirae	g__Candidatus_Magnetobacterium	0.001345	0.0004265	0.0009538	0.0001867	0.02575	0.05115	-0.00066	-9.78E-05	-0.00039
p__Firmicutes	g__Peribacillus	0.003898	0.002351	0.0004492	0.0002205	0.000183	0.002076	-0.00484	-0.00218	-0.00345
p__Firmicutes	g__Propionispora	0.003466	0.00198	0.0001625	4.95E-05	0.000183	0.002076	-0.00455	-0.0022	-0.0033
p__Firmicutes	g__Kurthia	0.00394	0.001062	0.002243	0.001045	0.002827	0.00937	-0.00257	-0.00083	-0.0017
p__Firmicutes	g__Domibacillus	0.001928	0.0005166	0.0002342	8.74E-05	0.000183	0.002076	-0.002	-0.0014	-0.00169
p__Firmicutes	g__Desulfotomaculum	0.003762	0.001545	0.002077	0.0005023	0.001706	0.006643	-0.00274	-0.00081	-0.00169
p__Firmicutes	g__Sulfobacillus	0.002174	0.00094	0.001201	0.000117	0.01133	0.02695	-0.00159	-0.00043	-0.00097
p__Firmicutes	g__Fictibacillus	0.001892	0.0009827	0.0009764	0.0004068	0.03764	0.06883	-0.00157	-0.00027	-0.00092
p__Firmicutes	g__Thermoanaerosceptrum	0.001155	0.0004551	0.0002395	8.17E-05	0.000183	0.002076	-0.0012	-0.00064	-0.00092
p__Firmicutes	g__Intestinimonas	0.001371	0.0001825	0.000507	0.0002733	0.000183	0.002076	-0.00106	-0.00068	-0.00086
p__Firmicutes	g__Carboxydothermus	0.001128	0.0005124	0.0002662	9.66E-05	0.000183	0.002076	-0.00118	-0.00056	-0.00086
p__Firmicutes	g__Effusibacillus	0.002445	0.0007661	0.001591	0.0003871	0.01726	0.03681	-0.00137	-0.00036	-0.00085
p__Firmicutes	g__Limnochorda	0.002234	0.0004278	0.001513	0.0002916	0.001008	0.004664	-0.00104	-0.00042	-0.00072
p__Firmicutes	g__unclassified_f__Veillonellaceae	0.0009143	0.0002282	0.000399	0.0001675	0.00044	0.00292	-0.00067	-0.00034	-0.00052
p__Firmicutes	g__Desnuesiella	0.0008396	0.0004127	0.0003993	0.0002177	0.01402	0.03183	-0.00071	-0.00018	-0.00044
p__Firmicutes	g__Dethiobacter	0.0005335	0.0001284	0.0001184	4.14E-05	0.000183	0.002076	-0.0005	-0.00034	-0.00042
p__Firmicutes	g__Thermanaeromonas	0.0008575	0.0001932	0.0005293	0.0002225	0.002202	0.008006	-0.00048	-0.00013	-0.00033
p__Firmicutes	g__Vallitalea	0.0003794	0.0001807	9.65E-05	4.62E-05	0.00033	0.002387	-0.0004	-0.00017	-0.00028
p__Firmicutes	g__Kyrpidia	0.0008099	0.0001433	0.0005473	0.0002135	0.009108	0.02275	-0.00042	-0.00011	-0.00026
p__Firmicutes	g__Desulfurispora	0.0003521	0.0002108	9.62E-05	0.0001095	0.001315	0.005574	-0.0004	-0.00012	-0.00026
p__Firmicutes	g__Ruminococcus	0.0009461	0.0002667	0.000691	0.0001849	0.02575	0.05115	-0.00046	-7.21E-05	-0.00026
p__Firmicutes	g__Numidum	0.0004907	0.0001404	0.0002477	0.0001062	0.002202	0.008006	-0.00034	-0.00014	-0.00024
p__Firmicutes	g__unclassified_f__Clostridiales_F	0.0004484	0.0001703	0.0002073	9.48E-05	0.001706	0.006643	-0.00037	-0.00014	-0.00024
p__Firmicutes	g__Salicibibacter	0.0002707	0.0001626	4.43E-05	3.63E-05	0.00033	0.002387	-0.00033	-0.00014	-0.00023
p__Firmicutes	g__Baia	0.0005701	0.0002012	0.0003464	0.0001111	0.01402	0.03183	-0.00037	-9.23E-05	-0.00022
p__Firmicutes	g__Sporotomaculum	0.0003219	0.0003395	0.0001177	0.0001779	0.02113	0.04338	-0.00042	1.49E-05	-0.0002
p__Firmicutes	g__Blautia	0.0004299	0.0002176	0.0002353	3.06E-05	0.02113	0.04338	-0.00033	-7.33E-05	-0.00019
p__Firmicutes	g__Exiguobacterium	0.0004457	0.0002017	0.0002512	0.0001056	0.01726	0.03681	-0.00034	-6.96E-05	-0.00019
p__Firmicutes	g__Pseudoflavonifractor	0.0003662	6.37E-05	0.0001738	5.83E-05	0.00033	0.002387	-0.00024	-0.00013	-0.00019
p__Firmicutes	g__Desulfofarcimen	0.0003938	0.0001127	0.0002087	6.56E-05	0.001008	0.004664	-0.00026	-0.00011	-0.00019
p__Firmicutes	g__Thermobacillus	0.0005524	0.0001327	0.000371	0.0001578	0.01402	0.03183	-0.0003	-6.17E-05	-0.00018
p__Firmicutes	g__Anaerotruncus	0.0003628	0.000128	0.000182	0.0001151	0.002827	0.00937	-0.00027	-7.86E-05	-0.00018
p__Firmicutes	g__Propionispira	0.0002627	0.0001393	9.32E-05	3.94E-05	0.02113	0.04338	-0.00025	-8.04E-05	-0.00017
p__Firmicutes	g__Salipaludibacillus	0.0001832	0.0001184	2.26E-05	1.07E-05	0.000183	0.002076	-0.00023	-8.84E-05	-0.00016
p__Firmicutes	g__Hydrogenibacillus	0.0003635	0.0001706	0.0002097	7.57E-05	0.007285	0.01916	-0.00027	-4.69E-05	-0.00015
p__Firmicutes	g__Metabacillus	0.0003339	0.0001235	0.0001893	0.0001521	0.01726	0.03681	-0.00026	-2.03E-05	-0.00014
p__Firmicutes	g__Fontibacillus	0.0001695	0.0001015	2.66E-05	2.15E-05	0.00033	0.002387	-0.0002	-8.14E-05	-0.00014

p__Firmicutes	g__Caldanaerobius	0.0002052	0.0001176	7.42E-05	4.59E-05	0.007285	0.01916	-0.00021	-5.70E-05	-0.00013
p__Firmicutes	g__Acidaminobacter	0.0002592	0.0001388	0.0001319	6.89E-05	0.009108	0.02275	-0.00023	-4.33E-05	-0.00013
p__Firmicutes	g__unclassified_c__Erysipelotrichi	0.0002107	9.25E-05	8.37E-05	4.98E-05	0.001315	0.005574	-0.0002	-6.91E-05	-0.00013
p__Firmicutes	g__Desulfocucumis	0.0001871	0.0001452	6.08E-05	4.00E-05	0.001706	0.006643	-0.00024	-5.28E-05	-0.00013
p__Firmicutes	g__Desmospora	0.0003462	9.79E-05	0.0002232	6.09E-05	0.005795	0.01601	-0.00019	-5.59E-05	-0.00012
p__Firmicutes	g__Thermovenabulum	0.0001237	0.0001235	1.12E-05	8.52E-06	0.001706	0.006643	-0.00019	-4.50E-05	-0.00011
p__Firmicutes	g__Marininema	0.0002848	0.0001418	0.0001743	4.65E-05	0.04515	0.07991	-0.0002	-2.30E-05	-0.00011
p__Firmicutes	g__Heliophilum	0.0001421	0.0001494	3.24E-05	1.36E-05	0.02113	0.04338	-0.0002	-2.43E-05	-0.00011
p__Firmicutes	g__Sporobacter	0.0001245	0.0001167	1.91E-05	1.62E-05	0.007285	0.01916	-0.00019	-4.54E-05	-0.00011
p__Firmicutes	g__Acidibacillus	0.0002438	8.31E-05	0.0001455	2.65E-05	0.003611	0.01118	-0.00015	-5.00E-05	-9.82E-05
p__Firmicutes	g__Geosporobacter	0.0001358	9.12E-05	3.81E-05	1.53E-05	0.000583	0.003312	-0.00015	-4.53E-05	-9.77E-05
p__Firmicutes	g__Proteiniclasticum	0.0001378	9.50E-05	4.58E-05	6.46E-05	0.009108	0.02275	-0.00016	-2.03E-05	-9.20E-05
p__Firmicutes	g__Caldanaerobacter	0.0001653	8.32E-05	7.71E-05	3.40E-05	0.007285	0.01916	-0.00015	-4.05E-05	-8.82E-05
p__Firmicutes	g__Candidatus_Carbobacillus	1.00E-04	6.43E-05	1.52E-05	1.59E-05	0.000241	0.0022	-0.00013	-4.95E-05	-8.48E-05
p__Firmicutes	g__Dehalobacterium	0.0001167	7.20E-05	3.42E-05	1.64E-05	0.001315	0.005574	-0.00013	-4.22E-05	-8.25E-05
p__Firmicutes	g__Epulopiscium	0.0001376	6.98E-05	6.09E-05	3.99E-05	0.002202	0.008006	-0.00013	-3.18E-05	-7.67E-05
p__Firmicutes	g__Paenisporosarcina	9.67E-05	4.82E-05	2.08E-05	1.71E-05	0.000769	0.00392	-0.00011	-4.48E-05	-7.59E-05
p__Firmicutes	g__Lucifera	0.0001676	4.31E-05	9.21E-05	4.80E-05	0.005795	0.01601	-0.00011	-3.62E-05	-7.55E-05
p__Firmicutes	g__Metasolibacillus	7.89E-05	2.83E-05	9.86E-06	9.84E-06	0.000173	0.002076	-8.62E-05	-5.12E-05	-6.91E-05
p__Firmicutes	g__Clostridioides	0.0002994	8.23E-05	0.0002317	0.0001749	0.02575	0.05115	-0.00017	6.79E-05	-6.77E-05
p__Firmicutes	g__Gelria	7.34E-05	4.41E-05	6.24E-06	9.78E-06	0.000297	0.002387	-9.51E-05	-4.28E-05	-6.71E-05
p__Firmicutes	g__Gudongella	0.0001466	8.47E-05	7.95E-05	7.13E-05	0.04515	0.07991	-0.00013	-1.15E-06	-6.71E-05
p__Firmicutes	g__Caldicoprobacter	0.0001476	6.25E-05	8.39E-05	2.67E-05	0.03121	0.05952	-0.00011	-2.28E-05	-6.38E-05
p__Firmicutes	g__Hazenella	0.000113	5.24E-05	4.96E-05	3.13E-05	0.004586	0.0134	-0.00011	-2.99E-05	-6.34E-05
p__Firmicutes	g__Veillonella	9.36E-05	3.81E-05	3.10E-05	1.73E-05	0.000583	0.003312	-8.97E-05	-3.70E-05	-6.27E-05
p__Firmicutes	g__Chengkuizengella	0.000132	7.85E-05	7.04E-05	8.31E-05	0.03764	0.06883	-0.00013	1.04E-05	-6.16E-05
p__Firmicutes	g__Desulfitibacter	9.74E-05	6.48E-05	3.59E-05	2.50E-05	0.007285	0.01916	-0.0001	-2.10E-05	-6.15E-05
p__Firmicutes	g__Aquibacillus	9.31E-05	3.61E-05	3.19E-05	1.76E-05	0.000769	0.00392	-8.27E-05	-3.98E-05	-6.12E-05
p__Firmicutes	g__unclassified_f__Erysipelotricha	8.67E-05	3.99E-05	2.66E-05	1.21E-05	0.002827	0.00937	-8.16E-05	-3.33E-05	-6.00E-05
p__Firmicutes	g__Pontibacillus	0.0001842	5.65E-05	0.0001252	0.0001127	0.03121	0.05952	-0.00013	1.52E-05	-5.90E-05
p__Firmicutes	g__Chryseomicrobium	7.61E-05	4.11E-05	1.99E-05	1.65E-05	0.003611	0.01118	-8.15E-05	-3.14E-05	-5.63E-05
p__Firmicutes	g__Oxobacter	6.99E-05	3.53E-05	1.39E-05	1.59E-05	0.00099	0.004664	-7.93E-05	-3.35E-05	-5.61E-05
p__Firmicutes	g__Thermohalobacter	5.54E-05	7.13E-05	6.00E-06	6.41E-06	0.03514	0.06624	-9.44E-05	-1.01E-05	-4.93E-05
p__Firmicutes	g__Megamonas	5.94E-05	5.85E-05	1.10E-05	1.51E-05	0.004286	0.0132	-8.90E-05	-1.72E-05	-4.84E-05
p__Firmicutes	g__Clostridiisalibacter	5.45E-05	1.45E-05	6.78E-06	1.33E-05	0.000274	0.002387	-5.89E-05	-3.56E-05	-4.77E-05
p__Firmicutes	g__Abyssisolibacter	5.19E-05	3.86E-05	5.10E-06	1.46E-05	0.000681	0.003854	-7.34E-05	-2.28E-05	-4.68E-05
p__Firmicutes	g__Gallicola	4.18E-05	2.29E-05	0	0	6.39E-05	0.002076	-5.60E-05	-2.96E-05	-4.18E-05
p__Firmicutes	g__Filobacillus	5.61E-05	4.61E-05	1.48E-05	1.76E-05	0.004435	0.0134	-7.06E-05	-1.60E-05	-4.13E-05
p__Firmicutes	g__Lactococcus	4.48E-05	2.29E-05	6.11E-06	4.51E-06	0.000245	0.0022	-5.27E-05	-2.45E-05	-3.87E-05
p__Firmicutes	g__Anaeromicrobium	4.55E-05	3.06E-05	8.43E-06	8.87E-06	0.001008	0.004664	-5.78E-05	-1.97E-05	-3.70E-05
p__Firmicutes	g__unclassified_f__Clostridiales_F	4.32E-05	2.40E-05	6.63E-06	6.48E-06	0.000173	0.002076	-5.26E-05	-2.42E-05	-3.66E-05
p__Firmicutes	g__Proteiniborus	4.83E-05	2.60E-05	1.30E-05	1.24E-05	0.001293	0.005574	-5.28E-05	-1.94E-05	-3.54E-05
p__Firmicutes	g__Tuberibacillus	6.55E-05	2.05E-05	3.13E-05	1.69E-05	0.001008	0.004664	-4.93E-05	-1.85E-05	-3.42E-05
p__Firmicutes	g__Falsibacillus	5.30E-05	3.49E-05	2.10E-05	1.09E-05	0.02575	0.05115	-5.72E-05	-1.08E-05	-3.19E-05
p__Firmicutes	g__Inediibacterium	4.10E-05	1.72E-05	9.11E-06	8.37E-06	0.001309	0.005574	-4.39E-05	-2.05E-05	-3.19E-05
p__Firmicutes	g__Indiicoccus	5.35E-05	2.23E-05	2.30E-05	1.71E-05	0.004586	0.0134	-4.84E-05	-1.43E-05	-3.06E-05
p__Firmicutes	g__Aminipila	5.05E-05	2.83E-05	2.04E-05	2.10E-05	0.02575	0.05115	-5.10E-05	-8.65E-06	-3.01E-05
p__Firmicutes	g__Tepidibacter	4.69E-05	2.91E-05	1.79E-05	2.09E-05	0.01709	0.03681	-5.03E-05	-7.37E-06	-2.91E-05

p__Firmicutes	g__Alkalibacterium	3.34E-05	3.28E-05	6.39E-06	6.56E-06	0.008849	0.02275	-5.01E-05	-8.82E-06	-2.70E-05
p__Firmicutes	g__Paucisalibacillus	4.06E-05	3.44E-05	1.39E-05	1.64E-05	0.005795	0.01601	-5.38E-05	-7.09E-06	-2.68E-05
p__Firmicutes	g__Acetoanaerobium	2.65E-05	1.59E-05	0	0	0.000231	0.0022	-3.56E-05	-1.70E-05	-2.65E-05
p__Firmicutes	g__Massilibacillus	3.47E-05	2.67E-05	1.03E-05	1.16E-05	0.01866	0.03978	-4.33E-05	-8.14E-06	-2.43E-05
p__Firmicutes	g__Caproiciproducens	3.13E-05	1.91E-05	8.52E-06	2.11E-05	0.002282	0.00824	-3.85E-05	-4.59E-06	-2.28E-05
p__Firmicutes	g__Absiella	2.46E-05	2.67E-05	2.08E-06	3.46E-06	0.0292	0.05791	-3.97E-05	-8.86E-06	-2.25E-05
p__Firmicutes	g__Intestinibacter	3.10E-05	1.47E-05	1.01E-05	5.80E-06	0.001706	0.006643	-3.02E-05	-1.14E-05	-2.09E-05
p__Firmicutes	g__Tenuibacillus	2.03E-05	1.67E-05	0	0	6.39E-05	0.002076	-2.97E-05	-1.02E-05	-2.03E-05
p__Firmicutes	g__Tetragenococcus	2.44E-05	2.07E-05	4.55E-06	7.73E-06	0.00967	0.02414	-3.27E-05	-7.47E-06	-1.98E-05
p__Firmicutes	g__Salisediminibacterium	1.94E-05	1.98E-05	0	0	0.000751	0.00392	-3.16E-05	-8.54E-06	-1.94E-05
p__Firmicutes	g__Jeotgalicoccus	1.91E-05	2.20E-05	0	0	0.002213	0.008006	-3.32E-05	-7.10E-06	-1.91E-05
p__Firmicutes	g__Faecalicatena	3.45E-05	2.85E-05	1.57E-05	3.04E-05	0.01643	0.0368	-4.18E-05	6.74E-06	-1.88E-05
p__Firmicutes	g__Agathobaculum	2.49E-05	2.20E-05	6.90E-06	1.06E-05	0.04329	0.07899	-3.30E-05	-3.26E-06	-1.80E-05
p__Firmicutes	g__Mobilibacterium	1.85E-05	1.71E-05	1.18E-06	2.60E-06	0.003198	0.01054	-2.88E-05	-7.84E-06	-1.73E-05
p__Firmicutes	g__Candidatus_Arthromitus	1.65E-05	2.17E-05	5.08E-07	1.61E-06	0.01875	0.03993	-2.99E-05	-4.12E-06	-1.60E-05
p__Firmicutes	g__Andreesenia	1.35E-05	9.25E-06	0	0	0.000231	0.0022	-1.89E-05	-7.32E-06	-1.35E-05
p__Firmicutes	g__Caprobacter	1.37E-05	1.17E-05	5.17E-07	1.64E-06	0.000515	0.003312	-2.01E-05	-6.24E-06	-1.32E-05
p__Firmicutes	g__Colidextribacter	1.20E-05	1.22E-05	0	0	0.002213	0.008006	-2.01E-05	-5.92E-06	-1.20E-05
p__Firmicutes	g__Rummeliibacillus	1.39E-05	1.27E-05	2.13E-06	5.36E-06	0.01095	0.02695	-1.97E-05	-4.25E-06	-1.17E-05
p__Firmicutes	g__Marinilactibacillus	3.06E-05	1.78E-05	2.07E-05	3.83E-05	0.04483	0.07991	-3.17E-05	1.90E-05	-9.96E-06
p__Firmicutes	g__Colibacter	9.64E-06	7.55E-06	4.22E-07	1.33E-06	0.001444	0.006098	-1.39E-05	-4.54E-06	-9.21E-06
p__Firmicutes	g__Marasmitruncus	8.99E-06	6.28E-06	0	0	0.000751	0.00392	-1.28E-05	-5.17E-06	-8.99E-06
p__Firmicutes	g__Natranaerobius	3.13E-05	1.77E-05	2.24E-05	4.59E-05	0.02575	0.05115	-3.12E-05	2.22E-05	-8.93E-06
p__Firmicutes	g__Centipeda_f__Selenomonadace	7.73E-06	9.61E-06	0	0	0.01493	0.03351	-1.41E-05	-2.59E-06	-7.73E-06
p__Firmicutes	g__Massilimaliae	7.18E-06	8.56E-06	0	0	0.01493	0.03351	-1.26E-05	-2.26E-06	-7.18E-06
p__Firmicutes	g__Natribacillus	1.00E-05	1.35E-05	2.92E-06	7.99E-06	0.03486	0.06597	-1.69E-05	1.57E-06	-7.11E-06
p__Firmicutes	g__Eggerthia	7.08E-06	1.35E-05	0	0	0.03498	0.06597	-1.61E-05	-1.15E-06	-7.08E-06
p__Firmicutes	g__Atopococcus	6.73E-06	1.19E-05	0	0	0.01493	0.03351	-1.45E-05	-1.33E-06	-6.73E-06
p__Firmicutes	g__Ileibacterium	6.18E-06	6.22E-06	2.03E-06	6.42E-06	0.02653	0.05264	-9.05E-06	1.55E-06	-4.15E-06
p__Firmicutes	g__Faecalibaculum	3.85E-06	6.42E-06	0	0	0.03498	0.06597	-7.89E-06	-8.66E-07	-3.85E-06
p__Firmicutes	g__Drancourtella	2.89E-06	4.37E-06	0	0	0.03498	0.06597	-5.77E-06	-5.74E-07	-2.89E-06
p__Firmicutes	g__Kallipyga	2.57E-06	3.89E-06	0	0	0.03498	0.06597	-5.16E-06	-4.73E-07	-2.58E-06
p__Chloroflexi	g__unclassified_c__Candidatus_Th	0.01346	0.006662	0.007344	0.005286	0.02113	0.04338	-0.01143	-0.00108	-0.00612
p__Chloroflexi	g__Ktedonosporobacter	0.008018	0.0007933	0.006535	0.002103	0.04515	0.07991	-0.00273	-0.00019	-0.00148
p__Chloroflexi	g__Thermanaerothrix	0.0009715	0.0003864	0.0003029	9.47E-05	0.000183	0.002076	-0.00092	-0.00044	-0.00067
p__Chloroflexi	g__Longilinea	0.0009964	0.0003095	0.0006081	0.0001094	0.004586	0.0134	-0.00059	-0.00019	-0.00039
p__Chloroflexi	g__Ornatilinea	0.0008972	0.0001609	0.0006499	0.0002079	0.01402	0.03183	-0.00041	-9.43E-05	-0.00025
p__Chloroflexi	g__Brevefilum	6.12E-05	3.17E-05	2.45E-05	2.29E-05	0.009108	0.02275	-6.02E-05	-1.23E-05	-3.67E-05
p__Verrucomicrobia	g__Pedosphaera	0.1595	0.04666	0.06378	0.01483	0.000183	0.002076	-0.1242	-0.06541	-0.09578
p__Verrucomicrobia	g__unclassified_f__Verrucomicrob	0.1	0.02776	0.06157	0.01291	0.002202	0.008006	-0.05694	-0.01948	-0.03846
p__Verrucomicrobia	g__Haloferula	0.004614	0.0009874	0.003849	0.001143	0.04515	0.07991	-0.00169	0.0001	-0.00076
p__Verrucomicrobia	g__unclassified_o__Methylacidiphi	0.003367	0.0003194	0.002627	0.0004206	0.001008	0.004664	-0.00105	-0.0004	-0.00074
p__Verrucomicrobia	g__Limisphaera	0.00135	0.0003141	0.0007983	0.0001669	0.00044	0.00292	-0.00077	-0.00035	-0.00055
p__Verrucomicrobia	g__Candidatus_Xiphinematobacter	4.35E-05	1.95E-05	1.50E-05	1.59E-05	0.002827	0.00937	-4.22E-05	-1.41E-05	-2.85E-05
p__Aquificae	g__Phorcysia	0.001491	0.0009247	1.06E-05	1.59E-05	0.000179	0.002076	-0.00203	-0.00099	-0.00148
p__Aquificae	g__Aquifex	0.0002666	0.0002261	6.54E-05	7.27E-05	0.002202	0.008006	-0.00036	-7.78E-05	-0.0002
p__Aquificae	g__Thermosulfidibacter	0.0004064	9.40E-05	0.0002648	7.74E-05	0.002827	0.00937	-0.00021	-6.45E-05	-0.00014
p__Aquificae	g__Thermovibrio	0.0002075	0.000188	8.44E-05	6.79E-05	0.04515	0.07991	-0.00024	-2.10E-05	-0.00012

p__Aquificae	g__Desulfurobacterium	7.87E-05	3.08E-05	5.11E-05	4.07E-05	0.03121	0.05952	-5.48E-05	5.99E-06	-2.76E-05
p__Aquificae	g__Hydrogenivirga	3.38E-05	3.05E-05	1.05E-05	1.50E-05	0.0386	0.07056	-4.33E-05	-4.44E-06	-2.33E-05
p__Aquificae	g__Hydrogenobaculum	4.52E-06	7.75E-06	0	0	0.03498	0.06597	-9.86E-06	-4.73E-07	-4.52E-06
p__Caldiserica	g__unclassified_o__Caldisericales	0.0003669	8.62E-05	0.0001198	5.18E-05	0.000183	0.002076	-0.0003	-0.00019	-0.00025
p__Caldiserica	g__Caldisericum	0.0001259	8.15E-05	4.34E-05	4.44E-05	0.01398	0.03183	-0.00014	-3.18E-05	-8.24E-05
p__candidate_divisio	g__unclassified_p__candidate_divis	0.0007267	0.0003569	0.0003434	0.0004215	0.01133	0.02695	-0.0007	-4.70E-05	-0.00038
p__candidate_divisio	g__unclassified_p__candidate_divis	0.0002216	9.63E-05	0.0001379	6.32E-05	0.03121	0.05952	-0.00015	-1.64E-05	-8.37E-05
p__candidate_divisio	g__Candidatus_Methylomirabilis	0.0141	0.001974	0.0081	0.001226	0.000246	0.0022	-0.00733	-0.00466	-0.00601
p__Candidatus_Abys	g__unclassified_p__Candidatus_Ab	0.005318	0.0006663	0.004418	0.0008696	0.01726	0.03681	-0.00154	-0.0002	-0.0009
p__Candidatus_Aero	g__unclassified_p__Candidatus_Ae	0.001498	0.0002429	0.001184	0.0001881	0.004586	0.0134	-0.00049	-0.00015	-0.00031
p__Candidatus_Ame	g__unclassified_p__Candidatus_Ar	0.0006624	0.0001663	0.0003877	0.0001299	0.002202	0.008006	-0.0004	-0.00015	-0.00027
p__Candidatus_Bipo	g__Candidatus_Bipolaricaulis	0.0006384	0.0002097	0.0003434	0.0001061	0.001008	0.004664	-0.00043	-0.00017	-0.0003
p__Candidatus_Coat	g__unclassified_p__Candidatus_Co	0.0002948	7.59E-05	0.0001538	4.97E-05	0.001008	0.004664	-0.0002	-8.85E-05	-0.00014
p__Candidatus_Colw	g__unclassified_p__Candidatus_Co	0.00011	6.55E-05	2.97E-05	1.42E-05	0.001706	0.006643	-0.00012	-4.25E-05	-8.03E-05
p__Candidatus_Falko	g__unclassified_p__Candidatus_Fa	0.002968	0.00089	0.0009046	0.0001902	0.000183	0.002076	-0.00262	-0.00159	-0.00206
p__Candidatus_Ferm	g__unclassified_p__Candidatus_Fe	0.0009845	0.0002807	0.0006874	0.0001535	0.009108	0.02275	-0.00049	-0.00012	-0.0003
p__Candidatus_Ferm	g__Candidatus_Fermentibacter	0.0002356	9.50E-05	0.0001356	9.01E-05	0.03121	0.05952	-0.00017	-2.10E-05	-0.0001
p__Candidatus_Hydr	g__unclassified_p__Candidatus_Hy	0.02258	0.004481	0.01825	0.003033	0.04515	0.07991	-0.0074	-0.0009	-0.00433
p__Candidatus_Jorge	g__unclassified_p__Candidatus_Jo	0.001361	0.0005842	0.0005755	0.0003176	0.000769	0.00392	-0.00123	-0.0004	-0.00079
p__Candidatus_Kryp	g__Candidatus_Kryptonium	0.000709	0.0002921	0.0002262	0.0001182	0.00033	0.002387	-0.00068	-0.0003	-0.00048
p__Candidatus_Kryp	g__Candidatus_Chrysopegis	0.0003871	0.0002202	8.06E-05	2.56E-05	0.000183	0.002076	-0.00044	-0.00019	-0.00031
p__Candidatus_Kryp	g__Candidatus_Thermokryptus	0.0003491	0.0001309	0.0001602	6.73E-05	0.001315	0.005574	-0.00028	-0.00011	-0.00019
p__Candidatus_Kryp	g__Candidatus_Kryptobacter	0.0002897	9.59E-05	0.0001894	8.69E-05	0.03764	0.06883	-0.00018	-2.60E-05	-0.0001
p__Candidatus_Lipto	g__unclassified_p__Candidatus_Li	0.000287	0.0001875	0.0001166	6.88E-05	0.009108	0.02275	-0.0003	-5.94E-05	-0.00017
p__Candidatus_Lloy	g__unclassified_p__Candidatus_Ll	0.0004016	0.0001473	0.0002173	9.84E-05	0.01726	0.03681	-0.00029	-7.63E-05	-0.00018
p__Candidatus_Marg	g__Candidatus_Termititenax	5.03E-05	2.85E-05	1.62E-05	1.93E-05	0.002827	0.00937	-5.39E-05	-1.29E-05	-3.41E-05
p__Candidatus_Mari	g__unclassified_p__Candidatus_M	0.01065	0.001624	0.008651	0.00147	0.01726	0.03681	-0.00328	-0.00073	-0.002
p__Candidatus_Mela	g__unclassified_o__Candidatus_Ga	0.0002721	0.0001835	3.92E-05	2.44E-05	0.00033	0.002387	-0.00034	-0.00013	-0.00023
p__Candidatus_Mela	g__unclassified_o__Candidatus_Ca	0.0003354	0.0001572	0.0001939	5.44E-05	0.02575	0.05115	-0.00025	-4.98E-05	-0.00014
p__Candidatus_Micr	g__unclassified_p__Candidatus_M	0.0003575	0.0001182	0.0002082	0.0001046	0.01726	0.03681	-0.00024	-5.57E-05	-0.00015
p__Candidatus_Neals	g__unclassified_p__Candidatus_Ne	0.03035	0.006436	0.01473	0.003049	0.000183	0.002076	-0.01967	-0.01104	-0.01562
p__Candidatus_Niyo	g__unclassified_p__Candidatus_Ni	0.001033	0.0004841	0.0005183	0.0004607	0.02113	0.04338	-0.00091	-0.00011	-0.00051
p__Candidatus_Pereg	g__unclassified_o__Candidatus_Pe	0.0009436	0.0001952	0.0004082	0.0001643	0.00033	0.002387	-0.00069	-0.00038	-0.00054
p__Candidatus_Rifle	g__unclassified_p__Candidatus_Ri	0.00207	0.0004234	0.0011	0.0002314	0.000246	0.0022	-0.00127	-0.0007	-0.00097
p__Candidatus_Ryan	g__unclassified_p__Candidatus_Ry	0.0008413	0.0002198	0.0004895	0.0001123	0.00033	0.002387	-0.00051	-0.00022	-0.00035
p__Candidatus_Stask	g__unclassified_p__Candidatus_St	0.0004996	0.0003612	0.0001974	9.60E-05	0.03764	0.06883	-0.00054	-8.53E-05	-0.0003
p__Candidatus_Sung	g__unclassified_p__Candidatus_Su	0.0005181	0.0001153	0.0002934	0.0001054	0.001315	0.005574	-0.00031	-0.00013	-0.00022
p__Candidatus_Terry	g__unclassified_p__Candidatus_Te	0.0005219	0.0001965	0.0001927	6.07E-05	0.00044	0.00292	-0.00045	-0.00021	-0.00033
p__Candidatus_Uhrb	g__unclassified_p__Candidatus_Uh	0.008004	0.008654	0.001785	0.0003224	0.00033	0.002387	-0.01193	-0.00198	-0.00622
p__Candidatus_Vebl	g__unclassified_p__Candidatus_Ve	1.91E-05	1.20E-05	7.31E-06	1.54E-05	0.01498	0.03359	-2.19E-05	-4.65E-07	-1.18E-05
p__Candidatus_Wall	g__unclassified_p__Candidatus_W	0.0005195	0.0001216	0.0001859	7.44E-05	0.000183	0.002076	-0.00042	-0.00025	-0.00033
p__Candidatus_Yona	g__unclassified_p__Candidatus_Yo	0.0009811	0.0004049	0.0002833	0.000135	0.000183	0.002076	-0.00095	-0.00048	-0.0007
p__Candidatus_Zaml	g__unclassified_p__Candidatus_Za	0.00334	0.001386	0.001818	0.0005527	0.003611	0.01118	-0.00244	-0.00068	-0.00152
p__Chlamydiae	g__Parachlamydia	0.02584	0.01419	0.004481	0.001699	0.000183	0.002076	-0.03107	-0.01335	-0.02135
p__Chlamydiae	g__unclassified_p__Chlamydiae	0.01983	0.008572	0.006407	0.001327	0.000183	0.002076	-0.01903	-0.00887	-0.01343
p__Chlamydiae	g__unclassified_o__Chlamydiales	0.01463	0.008068	0.002255	0.0008191	0.000183	0.002076	-0.01754	-0.00823	-0.01238
p__Chlamydiae	g__Candidatus_Protochlamydia	0.008437	0.004437	0.00361	0.001414	0.001008	0.004664	-0.00752	-0.00248	-0.00483
p__Chlamydiae	g__Neochlamydia	0.005029	0.002433	0.001047	0.0003343	0.000183	0.002076	-0.00546	-0.00258	-0.00398

p__Chlamydiae	g__Waddlia	0.002408	0.001264	0.0006077	0.0001821	0.000183	0.002076	-0.00259	-0.00108	-0.0018
p__Chlamydiae	g__unclassified_f__Parachlamydiac	0.00201	0.001073	0.0005035	0.0001583	0.000183	0.002076	-0.00217	-0.00096	-0.00151
p__Chlamydiae	g__Chlamydia	0.002608	0.0008167	0.001201	0.0002704	0.000183	0.002076	-0.00195	-0.00091	-0.00141
p__Chlamydiae	g__Criblamydia	0.001805	0.0008636	0.0004384	0.0001849	0.000183	0.002076	-0.00195	-0.00085	-0.00137
p__Chlamydiae	g__Candidatus_Rubidus	0.001185	0.0006096	0.0002762	8.88E-05	0.000183	0.002076	-0.00131	-0.00056	-0.00091
p__Chlamydiae	g__unclassified_f__Waddliaceae	0.00143	0.0007381	0.0006049	0.000233	0.001706	0.006643	-0.00126	-0.0004	-0.00083
p__Chlamydiae	g__Estrella	0.001205	0.0005766	0.0004597	0.0001893	0.001008	0.004664	-0.00112	-0.00042	-0.00075
p__Chlamydiae	g__Simkania	0.0004245	0.0001305	0.0001349	7.51E-05	0.00033	0.002387	-0.00038	-0.00021	-0.00029
p__Chlamydiae	g__unclassified_o__Anoxychlamyd	0.0003666	0.0001481	0.0001266	5.96E-05	0.000183	0.002076	-0.00035	-0.00015	-0.00024
p__Chlamydiae	g__unclassified_c__Chlamydiia	0.0001258	5.23E-05	6.73E-05	3.15E-05	0.01402	0.03183	-9.61E-05	-2.00E-05	-5.85E-05
p__Chlorobi	g__Pelodictyon	0.002227	0.0005619	0.0005665	9.15E-05	0.000183	0.002076	-0.00197	-0.00131	-0.00166
p__Chlorobi	g__Chlorobaculum	0.001613	0.0002506	0.00101	0.0002958	0.000769	0.00392	-0.00083	-0.00038	-0.0006
p__Chlorobi	g__unclassified_f__Candidatus_Th	0.0006333	0.0002753	0.0003291	0.0001568	0.01726	0.03681	-0.00049	-0.00012	-0.0003
p__Chlorobi	g__Prosthecochloris	0.001217	0.0002442	0.0009861	0.0002695	0.03764	0.06883	-0.00044	-5.51E-06	-0.00023
p__Chrysiogenetes_d	g__unclassified_o__Chrysiogenales	0.0005154	0.0002929	0.0002037	6.89E-05	0.005795	0.01601	-0.0005	-0.00013	-0.00031
p__Coprothermobact	g__Coprothermobacter	7.57E-05	4.31E-05	3.16E-05	1.64E-05	0.01133	0.02695	-7.21E-05	-1.76E-05	-4.41E-05
p__Coprothermobact	g__unclassified_f__Coprothermoba	2.04E-05	2.02E-05	4.22E-07	1.33E-06	0.006328	0.01737	-3.18E-05	-8.25E-06	-1.99E-05
p__Deferribacteres	g__unclassified_c__Deferribacteres	0.001332	0.0003676	0.0007763	0.0002019	0.001008	0.004664	-0.00081	-0.00032	-0.00056
p__Deferribacteres	g__Denitrovibrio	0.0003114	0.0001606	0.0001575	6.43E-05	0.009108	0.02275	-0.00026	-5.50E-05	-0.00015
p__Deferribacteres	g__Mucispirillum	0.0001062	7.38E-05	4.97E-06	5.44E-06	0.000173	0.002076	-0.00015	-6.01E-05	-0.0001
p__Deferribacteres	g__Flexistipes	2.81E-05	2.18E-05	2.71E-06	3.84E-06	0.002943	0.009735	-3.80E-05	-1.33E-05	-2.54E-05
p__Deinococcus-The	g__Marinithermus	0.002068	0.000922	0.0007583	0.0001919	0.00033	0.002387	-0.00188	-0.0008	-0.00131
p__Deinococcus-The	g__Oceanithermus	0.001544	0.0002708	0.001235	0.0001561	0.009108	0.02275	-0.00049	-0.00013	-0.00031
p__Elusimicrobia	g__Elusimicrobium	4.55E-05	3.25E-05	1.11E-05	1.33E-05	0.002202	0.008006	-5.71E-05	-1.59E-05	-3.44E-05
p__Elusimicrobia	g__Endomicrobium	3.23E-05	3.13E-05	1.22E-06	3.87E-06	0.000311	0.002387	-5.10E-05	-1.48E-05	-3.11E-05
p__Fusobacteria	g__Leptotrichia	0.0001571	0.0001241	6.34E-05	4.48E-05	0.03764	0.06883	-0.00017	-1.70E-05	-9.37E-05
p__Fusobacteria	g__Psychrilyobacter	3.49E-05	3.83E-05	1.67E-06	2.18E-06	0.000274	0.002387	-5.86E-05	-1.35E-05	-3.32E-05
p__Ignavibacteriae	g__unclassified_f__Melioribacterac	0.0003063	0.0001624	5.75E-05	6.28E-05	0.000583	0.003312	-0.00035	-0.00015	-0.00025
p__Ignavibacteriae	g__unclassified_f__Ignavibacteriac	0.0005771	0.0001136	0.0003825	0.0002151	0.02113	0.04338	-0.00033	-3.88E-05	-0.00019
p__Lentisphaerae	g__unclassified_o__Lentisphaerales	0.0009843	0.0002037	0.0006244	0.0001881	0.002827	0.00937	-0.00053	-0.00019	-0.00036
p__Spirochaetes	g__unclassified_p__Spirochaetes	0.0148	0.002235	0.01097	0.00313	0.01133	0.02695	-0.00606	-0.00135	-0.00383
p__Spirochaetes	g__Treponema	0.0026	0.0003798	0.001236	0.0001864	0.000183	0.002076	-0.0016	-0.0011	-0.00136
p__Spirochaetes	g__unclassified_f__Leptospiraceae	0.00152	0.001035	0.0005252	0.001178	0.002827	0.00937	-0.00184	-2.69E-06	-0.00099
p__Spirochaetes	g__Spirochaeta	0.0008039	0.000244	0.0004293	0.0001072	0.000769	0.00392	-0.00054	-0.00022	-0.00037
p__Spirochaetes	g__Sphaerochaeta	0.0001523	0.0001069	4.38E-05	2.46E-05	0.001008	0.004664	-0.00018	-4.94E-05	-0.00011
p__Spirochaetes	g__Marispirochaeta	0.0002566	0.0001047	0.0001553	6.25E-05	0.03764	0.06883	-0.00017	-2.58E-05	-0.0001
p__Spirochaetes	g__Rectinema	7.06E-05	5.65E-05	2.84E-05	1.39E-05	0.02113	0.04338	-7.86E-05	-1.19E-05	-4.22E-05
p__Spirochaetes	g__Borreliella	4.29E-05	4.03E-05	1.28E-06	2.09E-06	0.003819	0.01179	-6.79E-05	-2.02E-05	-4.16E-05
p__Spirochaetes	g__Exilispira	7.69E-05	3.80E-05	3.93E-05	2.83E-05	0.04515	0.07991	-6.70E-05	-1.17E-05	-3.77E-05
p__Synergistetes	g__unclassified_o__Synergistales	0.0002496	8.64E-05	6.41E-05	4.75E-05	0.00033	0.002387	-0.00024	-0.00012	-0.00019
p__Synergistetes	g__Fretibacterium	7.58E-05	5.15E-05	2.51E-05	1.55E-05	0.009108	0.02275	-8.26E-05	-2.01E-05	-5.06E-05
p__Tenericutes	g__unclassified_p__Tenericutes	0.0005324	0.0003273	0.000433	0.0005891	0.03764	0.06883	-0.00043	0.000364	-9.95E-05
p__Thermodesulfoba	g__Thermodesulfatator	0.0004359	0.000211	0.0001514	5.08E-05	0.002202	0.008006	-0.00042	-0.00016	-0.00028
p__Thermotogae	g__Petrotoga	0.001599	0.0005786	0.0009217	0.0003507	0.004586	0.0134	-0.0011	-0.00032	-0.00068
p__Thermotogae	g__Thermosipho_f__Fervidobacter	0.0001571	8.01E-05	4.85E-05	3.59E-05	0.001315	0.005574	-0.00016	-5.57E-05	-0.00011
p__Thermotogae	g__unclassified_o__Thermotogales	3.47E-05	2.50E-05	8.18E-06	1.28E-05	0.001572	0.006624	-4.43E-05	-1.16E-05	-2.65E-05
p__unclassified_d__I	g__Candidatus_Babela	0.0005785	0.0004089	6.64E-05	9.80E-05	0.00044	0.00292	-0.00078	-0.00026	-0.00051
p__unclassified_d__I	g__Vermiphilus	0.0002988	0.000299	5.88E-05	5.13E-05	0.001008	0.004664	-0.00044	-7.91E-05	-0.00024

p__unclassified_d__I	g__Candidatus_Chazhemtobacteriu	7.60E-05	9.28E-05	4.98E-06	6.28E-06	0.0007	0.00392	-0.00013	-2.40E-05	-7.11E-05
p__unclassified_d__I	g__Isachenkonia	7.31E-06	7.63E-06	0	0	0.005972	0.01642	-1.20E-05	-2.97E-06	-7.31E-06

Taxa abundances significantlly decreased under red light when compared to white light

Phylum	Genus	White-Mean (%	White-Sd (%	Red-Mean (%	Red-Sd (%	P value	Corrected pvalu	Lower ci	Upper ci	Effect size
p__Cyanobacteria	g__Myxacorys	0.08901	0.02301	0.0677	0.007893	0.01402	0.03427	-0.03645	-0.00817	-0.02131
p__Cyanobacteria	g__Rippkaea	0.02975	0.01239	0.01255	0.001707	0.000583	0.003709	-0.02485	-0.00981	-0.0172
p__Cyanobacteria	g__Chlorogloea	0.02792	0.007361	0.01766	0.001764	0.00033	0.002769	-0.01522	-0.00633	-0.01027
p__Cyanobacteria	g__Crocosphaera	0.02446	0.01051	0.01498	0.002189	0.01726	0.0402	-0.01672	-0.00334	-0.00948
p__Cyanobacteria	g__Synechococcus	0.04956	0.01043	0.04133	0.00489	0.04515	0.08665	-0.01512	-0.00186	-0.00823
p__Cyanobacteria	g__Jacksonvillea	0.01779	0.004333	0.01025	0.00146	0.000183	0.002329	-0.01005	-0.00495	-0.00754
p__Cyanobacteria	g__unclassified_f__Oscillatoriaceae	0.007656	0.002195	0.003917	0.00069	0.000246	0.00248	-0.00504	-0.00249	-0.00374
p__Cyanobacteria	g__Dolichospermum	0.01038	0.001847	0.006656	0.000884	0.000183	0.002329	-0.00495	-0.00248	-0.00373
p__Cyanobacteria	g__Spirulina	0.01464	0.004223	0.01094	0.001475	0.02575	0.05574	-0.00638	-0.00107	-0.0037
p__Cyanobacteria	g__Westiellopsis	0.01196	0.003428	0.008514	0.00111	0.002202	0.008721	-0.00555	-0.00151	-0.00345
p__Cyanobacteria	g__unclassified_f__Microcoleaceae	0.00611	0.001532	0.00474	0.000564	0.01726	0.0402	-0.00235	-0.0005	-0.00137
p__Cyanobacteria	g__Snowella	0.001565	0.001034	0.0004934	0.000226	0.001008	0.00518	-0.00174	-0.00048	-0.00107
p__Cyanobacteria	g__Limnospira	0.001427	0.0008452	0.0005323	0.000158	0.000183	0.002329	-0.00145	-0.00049	-0.0009
p__Cyanobacteria	g__Dactylococcopsis	0.001132	0.0003781	0.0005874	0.00024	0.001706	0.007373	-0.00079	-0.00029	-0.00054
p__Cyanobacteria	g__Trichodesmium	0.001088	0.0003422	0.0006157	0.000142	0.002827	0.01032	-0.00069	-0.00025	-0.00047
p__Cyanobacteria	g__Prochloron	0.0004801	0.000639	8.48E-06	1.11E-05	0.000179	0.002329	-0.0009	-0.0002	-0.00047
p__Cyanobacteria	g__Cylindrospermopsis	0.0008509	0.0002942	0.0004165	6.63E-05	0.001008	0.00518	-0.00062	-0.00026	-0.00043
p__Cyanobacteria	g__unclassified_f__Aphanizomenonac	0.0003858	0.0001464	0.000152	5.71E-05	0.001706	0.007373	-0.00032	-0.00014	-0.00023
p__Cyanobacteria	g__Cuspidothrix	0.0002446	0.0001525	0.0001089	6.41E-05	0.009108	0.02457	-0.00024	-4.78E-05	-0.00014
p__Cyanobacteria	g__Vulcanococcus	0.0002281	0.0001185	9.66E-05	2.18E-05	0.003611	0.01236	-0.00021	-5.50E-05	-0.00013
p__Cyanobacteria	g__Candidatus_Atelocyanobacterium	9.88E-05	3.90E-05	1.68E-05	1.14E-05	0.001706	0.007373	-0.0001	-5.70E-05	-8.20E-05
p__Cyanobacteria	g__unclassified_f__Chroococcaceae	5.33E-05	4.17E-05	1.76E-06	3.71E-06	0.000208	0.00248	-7.92E-05	-2.88E-05	-5.16E-05
p__Cyanobacteria	g__Chrysosporum	3.01E-05	1.98E-05	8.23E-06	1.34E-05	0.01498	0.03635	-3.63E-05	-6.89E-06	-2.18E-05
p__Proteobacteria	g__Sphingomonas	3.913	0.71	1.494	0.535	0.000183	0.002329	-2.988	-1.903	-2.419
p__Proteobacteria	g__unclassified_c__Deltaproteobacter	2.629	0.71	1.565	0.2269	0.000769	0.004362	-1.478	-0.6072	-1.064
p__Proteobacteria	g__unclassified_c__Gammaproteobact	2.286	0.2672	1.592	0.1633	0.000183	0.002329	-0.8817	-0.5084	-0.6945
p__Proteobacteria	g__unclassified_c__Betaproteobacteri	1.369	0.2263	0.9177	0.3103	0.003611	0.01236	-0.6644	-0.2229	-0.4513
p__Proteobacteria	g__Hyphomicrobium	1.329	0.3437	0.9181	0.1986	0.009108	0.02457	-0.6342	-0.1795	-0.4109
p__Proteobacteria	g__Methylibium	0.6906	0.1858	0.292	0.2004	0.002202	0.008721	-0.5658	-0.2374	-0.3986
p__Proteobacteria	g__Mesorhizobium	1.399	0.3128	1.024	0.2099	0.007285	0.02069	-0.589	-0.1569	-0.3751
p__Proteobacteria	g__Parvularcula	0.4003	0.2418	0.04853	0.02983	0.000183	0.002329	-0.5283	-0.2252	-0.3518
p__Proteobacteria	g__unclassified_p__Proteobacteria	1.94	0.214	1.625	0.2836	0.01402	0.03427	-0.5141	-0.09644	-0.3143
p__Proteobacteria	g__unclassified_f__Phyllobacteriaceae	0.7923	0.2451	0.5268	0.0631	0.002202	0.008721	-0.4331	-0.1336	-0.2654
p__Proteobacteria	g__unclassified_o__Myxococcales	0.6182	0.1209	0.3763	0.0641	0.000583	0.003709	-0.3213	-0.1673	-0.2419
p__Proteobacteria	g__Amphiplicatus	0.221	0.1451	0.02802	0.0188	0.000183	0.002329	-0.2884	-0.1122	-0.193
p__Proteobacteria	g__Rubrivivax	0.3105	0.1054	0.1687	0.08534	0.004586	0.01474	-0.2212	-0.06399	-0.1418
p__Proteobacteria	g__Pseudomonas	0.3725	0.22	0.2328	0.04057	0.03121	0.06485	-0.2941	-0.02479	-0.1397
p__Proteobacteria	g__Novosphingobium	0.2616	0.04393	0.1255	0.03435	0.000183	0.002329	-0.1676	-0.1038	-0.1361
p__Proteobacteria	g__unclassified_o__Burkholderiales	0.3688	0.09154	0.2362	0.07278	0.002827	0.01032	-0.2099	-0.06094	-0.1326
p__Proteobacteria	g__Sphingopyxis	0.3798	0.05006	0.2505	0.08054	0.002827	0.01032	-0.1824	-0.07068	-0.1293
p__Proteobacteria	g__unclassified_f__Sphingomonadace	0.2537	0.05503	0.137	0.05957	0.001706	0.007373	-0.164	-0.07008	-0.1168
p__Proteobacteria	g__Ideonella	0.218	0.06468	0.1108	0.0573	0.002202	0.008721	-0.1557	-0.05919	-0.1072
p__Proteobacteria	g__Bauldia	0.1688	0.02187	0.0624	0.0236	0.000183	0.002329	-0.1232	-0.08674	-0.1065
p__Proteobacteria	g__Piscinibacter	0.2267	0.07613	0.1243	0.06657	0.004586	0.01474	-0.164	-0.04323	-0.1024
p__Proteobacteria	g__Sphingobium	0.1998	0.02959	0.1072	0.03612	0.000583	0.003709	-0.1197	-0.06337	-0.09266
p__Proteobacteria	g__Sphingosinicella	0.223	0.03469	0.131	0.03549	0.00033	0.002769	-0.122	-0.06288	-0.09201

p__Proteobacteria	g__Rhizobacter	0.1793	0.05602	0.09504	0.04853	0.002202	0.008721	-0.1272	-0.03938	-0.08429
p__Proteobacteria	g__Sphingorhabdus	0.1456	0.02964	0.0724	0.03201	0.001008	0.00518	-0.1013	-0.04587	-0.07319
p__Proteobacteria	g__Phenylobacterium	0.2074	0.0599	0.1349	0.03093	0.009108	0.02457	-0.1096	-0.03038	-0.07253
p__Proteobacteria	g__Azohydromonas	0.1651	0.06505	0.09581	0.05078	0.009108	0.02457	-0.119	-0.02247	-0.06929
p__Proteobacteria	g__Chakrabartia	0.1211	0.03332	0.05718	0.0321	0.002827	0.01032	-0.09183	-0.03577	-0.06388
p__Proteobacteria	g__Pseudorivibacter	0.1226	0.03789	0.06146	0.0351	0.001315	0.006216	-0.09263	-0.03168	-0.06112
p__Proteobacteria	g__unclassified_f__Burkholderiaceae	0.1257	0.03581	0.07271	0.03139	0.002827	0.01032	-0.08127	-0.02393	-0.05302
p__Proteobacteria	g__Rivibacter	0.09552	0.02206	0.04307	0.02584	0.001008	0.00518	-0.07113	-0.03049	-0.05245
p__Proteobacteria	g__Aquabacterium	0.1096	0.03046	0.05895	0.0279	0.002202	0.008721	-0.07555	-0.02635	-0.05068
p__Proteobacteria	g__unclassified_f__Polyangiaceae	0.08464	0.02057	0.03455	0.005059	0.000183	0.002329	-0.06375	-0.03877	-0.05009
p__Proteobacteria	g__Variovorax	0.2123	0.04163	0.1663	0.03441	0.01726	0.0402	-0.0774	-0.01145	-0.04599
p__Proteobacteria	g__Aestuariisphingobium	0.09195	0.02952	0.04629	0.02673	0.009108	0.02457	-0.07021	-0.0232	-0.04566
p__Proteobacteria	g__Nordella	0.1452	0.02864	0.1025	0.01495	0.002202	0.008721	-0.06051	-0.02221	-0.04271
p__Proteobacteria	g__Altererythrobacter	0.09315	0.01491	0.05229	0.01295	0.000246	0.00248	-0.05232	-0.02896	-0.04086
p__Proteobacteria	g__Erythrobacter	0.07671	0.00967	0.03712	0.007509	0.000183	0.002329	-0.04703	-0.03206	-0.03958
p__Proteobacteria	g__unclassified_f__Kofleriaceae	0.05773	0.04849	0.01896	0.00687	0.02575	0.05574	-0.06785	-0.01252	-0.03878
p__Proteobacteria	g__Filomicrobium	0.07029	0.03025	0.03409	0.01107	0.004586	0.01474	-0.05515	-0.01758	-0.0362
p__Proteobacteria	g__Brevundimonas	0.09028	0.0123	0.06008	0.009644	0.000246	0.00248	-0.03949	-0.02079	-0.0302
p__Proteobacteria	g__Pseudorhodoplanes	0.1933	0.02321	0.1633	0.03413	0.02113	0.04755	-0.05542	-0.00668	-0.03
p__Proteobacteria	g__Marinicaulis	0.04025	0.02124	0.01042	0.00173	0.000183	0.002329	-0.0428	-0.01853	-0.02983
p__Proteobacteria	g__Haliangium	0.042	0.03053	0.01247	0.002222	0.000769	0.004362	-0.04987	-0.01329	-0.02953
p__Proteobacteria	g__Polyangium	0.06294	0.0155	0.03593	0.01121	0.001008	0.00518	-0.03896	-0.01581	-0.027
p__Proteobacteria	g__'Geomonas'_Khan_et_al._2020	0.05601	0.02009	0.03019	0.0164	0.004586	0.01474	-0.04218	-0.01087	-0.02582
p__Proteobacteria	g__unclassified_o__Chromatiales	0.07426	0.01993	0.0486	0.003786	0.000183	0.002329	-0.03798	-0.01463	-0.02566
p__Proteobacteria	g__unclassified_f__Rhizobiaceae	0.05694	0.0186	0.03255	0.004964	0.00044	0.003169	-0.03613	-0.01363	-0.02439
p__Proteobacteria	g__Paucibacter	0.05654	0.01883	0.03216	0.01579	0.007285	0.02069	-0.03914	-0.01048	-0.02438
p__Proteobacteria	g__Polaromonas	0.08773	0.02542	0.06363	0.01912	0.02113	0.04755	-0.04289	-0.00615	-0.0241
p__Proteobacteria	g__Pelomonas	0.06984	0.02063	0.04599	0.01675	0.009108	0.02457	-0.04089	-0.00669	-0.02386
p__Proteobacteria	g__Blastomonas	0.04658	0.00698	0.02313	0.009529	0.00044	0.003169	-0.03015	-0.01598	-0.02345
p__Proteobacteria	g__Nitratireductor	0.06972	0.02048	0.04783	0.006617	0.01133	0.02909	-0.03535	-0.00927	-0.02189
p__Proteobacteria	g__unclassified_f__Sinobacteraceae	0.0465	0.03317	0.02567	0.0106	0.01726	0.0402	-0.04267	-0.00245	-0.02083
p__Proteobacteria	g__Zhizhongheella	0.0396	0.01242	0.0202	0.01122	0.002202	0.008721	-0.02975	-0.01	-0.0194
p__Proteobacteria	g__Corallococcus	0.06969	0.01763	0.05087	0.004389	0.004586	0.01474	-0.03005	-0.00818	-0.01881
p__Proteobacteria	g__Aquamicrobium	0.0369	0.01154	0.01814	0.003812	0.00033	0.002769	-0.02628	-0.01141	-0.01876
p__Proteobacteria	g__Minicystis	0.04402	0.009737	0.02569	0.008695	0.001315	0.006216	-0.0254	-0.01037	-0.01833
p__Proteobacteria	g__Leptothrix_r__root	0.03378	0.009285	0.01592	0.009208	0.001315	0.006216	-0.02614	-0.01001	-0.01785
p__Proteobacteria	g__Porphyrobacter	0.03089	0.004664	0.01308	0.003213	0.000183	0.002329	-0.02118	-0.01435	-0.01781
p__Proteobacteria	g__Tardibacter	0.04709	0.0219	0.02977	0.01531	0.02113	0.04755	-0.03603	-0.00246	-0.01733
p__Proteobacteria	g__Schlegelella	0.03335	0.009843	0.01623	0.008481	0.001315	0.006216	-0.02464	-0.00949	-0.01711
p__Proteobacteria	g__Parasphingopyxis	0.02435	0.00469	0.007335	0.002483	0.000183	0.002329	-0.02012	-0.01396	-0.01701
p__Proteobacteria	g__Aminobacter	0.04331	0.01234	0.02654	0.004889	0.001706	0.007373	-0.02547	-0.00864	-0.01677
p__Proteobacteria	g__Sandarakinorhabdus	0.03103	0.00531	0.01459	0.004135	0.000183	0.002329	-0.0205	-0.01231	-0.01644
p__Proteobacteria	g__Rhodobacter	0.02633	0.01854	0.01027	0.002003	0.000246	0.00248	-0.02856	-0.00708	-0.01606
p__Proteobacteria	g__Pseudaminobacter	0.0373	0.008439	0.02272	0.002823	0.000246	0.00248	-0.01988	-0.00948	-0.01458
p__Proteobacteria	g__Parasphingorhabdus	0.0198	0.003512	0.005245	0.001379	0.000183	0.002329	-0.01674	-0.01251	-0.01455
p__Proteobacteria	g__Caulobacter	0.162	0.01129	0.1479	0.01254	0.03121	0.06485	-0.02383	-0.00492	-0.01407
p__Proteobacteria	g__unclassified_f__Rhodobacteraceae	0.05609	0.005875	0.04239	0.004047	0.000183	0.002329	-0.01792	-0.00974	-0.0137
p__Proteobacteria	g__Halioglobus	0.02295	0.01867	0.009573	0.001834	0.007285	0.02069	-0.02677	-0.00369	-0.01338

p__Proteobacteria	g__Caenimonas	0.03661	0.009864	0.02342	0.00785	0.005795	0.0177	-0.02053	-0.00614	-0.01319
p__Proteobacteria	g__Tistlia	0.03691	0.006878	0.02463	0.00331	0.000583	0.003709	-0.0169	-0.0078	-0.01229
p__Proteobacteria	g__Myxococcus	0.04178	0.01321	0.03003	0.002227	0.03764	0.07496	-0.0206	-0.00456	-0.01175
p__Proteobacteria	g__Acidovorax	0.04408	0.007037	0.03236	0.006096	0.002202	0.008721	-0.01766	-0.0064	-0.01172
p__Proteobacteria	g__Labilithrix	0.04032	0.008806	0.02907	0.008223	0.01133	0.02909	-0.01815	-0.0034	-0.01124
p__Proteobacteria	g__unclassified_f__Myxococcaceae	0.03132	0.008841	0.0207	0.002249	0.007285	0.02069	-0.01644	-0.00539	-0.01062
p__Proteobacteria	g__Chelativorans	0.0345	0.005961	0.02397	0.002066	0.000246	0.00248	-0.01419	-0.00691	-0.01053
p__Proteobacteria	g__Rhodoferax	0.03483	0.006934	0.02433	0.007025	0.003611	0.01236	-0.01638	-0.00411	-0.01051
p__Proteobacteria	g__Tatlockia	0.01401	0.006246	0.003746	0.00186	0.00044	0.003169	-0.01392	-0.00637	-0.01027
p__Proteobacteria	g__Fontimonas	0.01732	0.0172	0.007207	0.004989	0.02575	0.05574	-0.02197	-0.001	-0.01011
p__Proteobacteria	g__Pseudorhodoferax	0.02473	0.005979	0.01477	0.004884	0.001706	0.007373	-0.01475	-0.00545	-0.00996
p__Proteobacteria	g__Candidatus_Accumulibacter	0.03614	0.008175	0.02628	0.005046	0.007285	0.02069	-0.01605	-0.00444	-0.00986
p__Proteobacteria	g__Caldimonas	0.02123	0.007086	0.01142	0.005927	0.001706	0.007373	-0.01538	-0.00452	-0.00981
p__Proteobacteria	g__Chondromyces	0.03107	0.007596	0.02132	0.007153	0.005795	0.0177	-0.01602	-0.00369	-0.00975
p__Proteobacteria	g__Starkeya	0.04406	0.007942	0.03459	0.00672	0.01402	0.03427	-0.01585	-0.00294	-0.00947
p__Proteobacteria	g__Hyphomonas	0.03222	0.005205	0.02346	0.002734	0.000769	0.004362	-0.01206	-0.00554	-0.00876
p__Proteobacteria	g__Silvanigrella	0.009192	0.005861	0.0008757	0.000842	0.000183	0.002329	-0.01215	-0.00509	-0.00832
p__Proteobacteria	g__Wenzhouxiangella	0.02392	0.004732	0.01592	0.00242	0.00044	0.003169	-0.01132	-0.005	-0.008
p__Proteobacteria	g__Aliihoeflea	0.01174	0.004694	0.00401	0.001393	0.000183	0.002329	-0.01049	-0.00475	-0.00773
p__Proteobacteria	g__Aureimonas	0.02164	0.002384	0.01457	0.001438	0.000183	0.002329	-0.00867	-0.00543	-0.00707
p__Proteobacteria	g__Aestuariivirga	0.01587	0.007234	0.008973	0.001413	0.000583	0.003709	-0.01135	-0.00335	-0.0069
p__Proteobacteria	g__unclassified_f__Ectothiorhodospir	0.01839	0.007839	0.01155	0.004161	0.01726	0.0402	-0.01273	-0.00158	-0.00684
p__Proteobacteria	g__Curvibacter	0.0185	0.003792	0.01166	0.003364	0.002202	0.008721	-0.00989	-0.004	-0.00683
p__Proteobacteria	g__Xanthobacter	0.02802	0.005578	0.02176	0.00327	0.01726	0.0402	-0.01018	-0.00217	-0.00626
p__Proteobacteria	g__unclassified_f__Kiloniellaceae	0.03595	0.0061	0.02979	0.003171	0.01133	0.02909	-0.01059	-0.00225	-0.00616
p__Proteobacteria	g__Agrobacterium	0.02119	0.001165	0.01516	0.001133	0.000183	0.002329	-0.00698	-0.00508	-0.00604
p__Proteobacteria	g__Ancylobacter	0.01631	0.003146	0.01036	0.002324	0.001706	0.007373	-0.00856	-0.00357	-0.00595
p__Proteobacteria	g__Paracoccus_f__Rhodobacteraceae	0.0287	0.002496	0.02275	0.002565	0.000583	0.003709	-0.0083	-0.00372	-0.00596
p__Proteobacteria	g__unclassified_o__Caulobacterales	0.01528	0.002649	0.009374	0.001761	0.00044	0.003169	-0.00791	-0.00414	-0.00591
p__Proteobacteria	g__Novosphingopyxis	0.007942	0.001586	0.002061	0.000903	0.000183	0.002329	-0.00702	-0.00477	-0.00588
p__Proteobacteria	g__Sphaerotilus	0.01155	0.003047	0.005811	0.003141	0.001706	0.007373	-0.00833	-0.00304	-0.00574
p__Proteobacteria	g__Azoarcus	0.02725	0.004746	0.02156	0.00404	0.009108	0.02457	-0.00922	-0.00218	-0.00569
p__Proteobacteria	g__Ruegeria	0.01978	0.001503	0.0141	0.002991	0.000769	0.004362	-0.00762	-0.00359	-0.00568
p__Proteobacteria	g__unclassified_f__Sandaracinaceae	0.01475	0.003925	0.009199	0.00223	0.009108	0.02457	-0.0081	-0.00254	-0.00555
p__Proteobacteria	g__Noviherbaspirillum	0.02931	0.00205	0.02383	0.002322	0.00044	0.003169	-0.00735	-0.00358	-0.00548
p__Proteobacteria	g__Ensifer	0.03984	0.002739	0.03437	0.003629	0.003611	0.01236	-0.00819	-0.00262	-0.00547
p__Proteobacteria	g__Kaistia	0.03073	0.002552	0.02532	0.001724	0.001008	0.00518	-0.00708	-0.00366	-0.00541
p__Proteobacteria	g__Pacificimonas	0.007048	0.001407	0.001717	0.000639	0.000183	0.002329	-0.00619	-0.00432	-0.00533
p__Proteobacteria	g__Rhizorhabdus	0.01342	0.002383	0.008158	0.004329	0.005795	0.0177	-0.00799	-0.00214	-0.00526
p__Proteobacteria	g__Thiomonas	0.01132	0.002184	0.006155	0.001587	0.00033	0.002769	-0.0069	-0.00348	-0.00517
p__Proteobacteria	g__Inhella	0.01135	0.002753	0.006203	0.002719	0.001706	0.007373	-0.00741	-0.00262	-0.00515
p__Proteobacteria	g__Massilia	0.05075	0.00376	0.0457	0.003517	0.009108	0.02457	-0.00797	-0.00194	-0.00505
p__Proteobacteria	g__Defluviicoccus	0.02358	0.003635	0.01855	0.004304	0.01133	0.02909	-0.00851	-0.00156	-0.00503
p__Proteobacteria	g__Limnohabitans	0.01167	0.002046	0.006796	0.001591	0.000769	0.004362	-0.00637	-0.00319	-0.00487
p__Proteobacteria	g__Stenotrophobium	0.01145	0.007791	0.006587	0.00264	0.03121	0.06485	-0.01006	-0.00067	-0.00486
p__Proteobacteria	g__unclassified_f__Geminicoccaceae	0.01324	0.003896	0.008418	0.003628	0.009108	0.02457	-0.00798	-0.00174	-0.00482
p__Proteobacteria	g__Mitsuaria	0.01484	0.005097	0.01008	0.004074	0.02113	0.04755	-0.00858	-0.00079	-0.00477
p__Proteobacteria	g__Aromatoleum	0.02705	0.00336	0.02236	0.003051	0.004586	0.01474	-0.00744	-0.00201	-0.0047

p__Proteobacteria	g__Legionella	0.01409	0.002819	0.009404	0.001291	0.001008	0.00518	-0.00669	-0.00286	-0.00468
p__Proteobacteria	g__Hoeflea	0.01819	0.003391	0.0136	0.001716	0.002827	0.01032	-0.00693	-0.00238	-0.00459
p__Proteobacteria	g__Labrenzia	0.01236	0.0007802	0.007783	0.001062	0.000183	0.002329	-0.00531	-0.00378	-0.00457
p__Proteobacteria	g__Azorhizobium	0.01596	0.003719	0.0114	0.001927	0.004586	0.01474	-0.00714	-0.00224	-0.00456
p__Proteobacteria	g__Stigmatella	0.0154	0.004885	0.01086	0.002055	0.01402	0.03427	-0.00784	-0.00145	-0.00454
p__Proteobacteria	g__Hephaestia	0.01166	0.001482	0.00716	0.002527	0.002827	0.01032	-0.00619	-0.00275	-0.0045
p__Proteobacteria	g__Sandaracinobacter	0.00595	0.001019	0.001601	0.000465	0.000183	0.002329	-0.00505	-0.00371	-0.00435
p__Proteobacteria	g__Rhodovulum	0.01369	0.001947	0.009365	0.00109	0.000183	0.002329	-0.00568	-0.00298	-0.00432
p__Proteobacteria	g__Rhodoligotrophos	0.02858	0.002584	0.02433	0.002399	0.003611	0.01236	-0.00631	-0.0022	-0.00424
p__Proteobacteria	g__Citromicrobium	0.007982	0.00104	0.003841	0.000768	0.000183	0.002329	-0.00487	-0.00338	-0.00414
p__Proteobacteria	g__Denitrobaculum	0.02025	0.003837	0.01615	0.001935	0.01726	0.0402	-0.00681	-0.00174	-0.00411
p__Proteobacteria	g__Stakelama	0.006273	0.00112	0.002204	0.000725	0.000183	0.002329	-0.00482	-0.00326	-0.00407
p__Proteobacteria	g__unclassified_f__Desulfobacteracea	0.03034	0.002286	0.02637	0.001561	0.000246	0.00248	-0.00573	-0.00243	-0.00397
p__Proteobacteria	g__Roseateles	0.01255	0.002514	0.008623	0.002263	0.002827	0.01032	-0.00605	-0.00194	-0.00393
p__Proteobacteria	g__Thiobacillus	0.01461	0.001419	0.0107	0.0009	0.000183	0.002329	-0.00497	-0.00293	-0.00391
p__Proteobacteria	g__Tepidamorphus	0.01094	0.0007807	0.007038	0.000884	0.000183	0.002329	-0.00463	-0.0032	-0.0039
p__Proteobacteria	g__unclassified_o__Rhodocyclales	0.009911	0.001098	0.006028	0.000974	0.000183	0.002329	-0.00473	-0.00304	-0.00388
p__Proteobacteria	g__Ottowia	0.01642	0.005123	0.01255	0.002804	0.01726	0.0402	-0.00781	-0.00058	-0.00388
p__Proteobacteria	g__unclassified_o__Hydrogenophilale	0.02168	0.002612	0.01797	0.00173	0.001706	0.007373	-0.00578	-0.00197	-0.00372
p__Proteobacteria	g__Methylopila	0.009765	0.0008067	0.006103	0.00127	0.000246	0.00248	-0.00452	-0.00271	-0.00366
p__Proteobacteria	g__Croceicoccus	0.006806	0.001355	0.003184	0.001097	0.00044	0.003169	-0.00464	-0.00251	-0.00362
p__Proteobacteria	g__unclassified_o__Silvanigrellales	0.003994	0.002622	0.0003727	0.000428	0.000246	0.00248	-0.00526	-0.0022	-0.00362
p__Proteobacteria	g__Anaeromyxobacter	0.02084	0.004513	0.01722	0.001654	0.03764	0.07496	-0.00619	-0.00079	-0.00362
p__Proteobacteria	g__Pelagibius	0.02299	0.002864	0.0195	0.002849	0.02575	0.05574	-0.00619	-0.00113	-0.0035
p__Proteobacteria	g__Fodinicurvata	0.007767	0.002325	0.004329	0.000865	0.000769	0.004362	-0.00503	-0.00198	-0.00344
p__Proteobacteria	g__Hirschia	0.007233	0.001114	0.003803	0.001372	0.000246	0.00248	-0.00444	-0.0024	-0.00343
p__Proteobacteria	g__Thauera	0.01968	0.001952	0.01626	0.00211	0.002827	0.01032	-0.00521	-0.0018	-0.00342
p__Proteobacteria	g__Parahaliea	0.0112	0.002378	0.007784	0.001402	0.004586	0.01474	-0.00493	-0.00173	-0.00342
p__Proteobacteria	g__Thiocapsa	0.0164	0.00301	0.01304	0.002303	0.01402	0.03427	-0.00568	-0.0012	-0.00336
p__Proteobacteria	g__Comamonas	0.01322	0.001825	0.009868	0.001172	0.000583	0.003709	-0.00452	-0.00204	-0.00335
p__Proteobacteria	g__Kinneretia	0.00726	0.002294	0.00402	0.001846	0.002202	0.008721	-0.00498	-0.00171	-0.00324
p__Proteobacteria	g__Fluviispira	0.003581	0.002261	0.0003805	0.000328	0.000183	0.002329	-0.00461	-0.00199	-0.0032
p__Proteobacteria	g__unclassified_f__Methylococcaceae	0.01078	0.001151	0.00763	0.00162	0.000583	0.003709	-0.00435	-0.00199	-0.00315
p__Proteobacteria	g__unclassified_f__Halieaceae	0.009738	0.001807	0.00665	0.00094	0.00044	0.003169	-0.0044	-0.0019	-0.00309
p__Proteobacteria	g__Andreprevotia	0.007603	0.001047	0.004533	0.000672	0.000183	0.002329	-0.00384	-0.0023	-0.00307
p__Proteobacteria	g__Bradymonas	0.005926	0.00171	0.002972	0.000433	0.00044	0.003169	-0.00403	-0.00195	-0.00295
p__Proteobacteria	g__Methylocystis	0.02448	0.002144	0.02156	0.002458	0.01402	0.03427	-0.00491	-0.00102	-0.00292
p__Proteobacteria	g__Vitreoscilla	0.005503	0.00176	0.002644	0.001484	0.002202	0.008721	-0.00414	-0.00158	-0.00286
p__Proteobacteria	g__unclassified_o__Desulfurellales	0.01094	0.002083	0.008108	0.001466	0.004586	0.01474	-0.00422	-0.00136	-0.00284
p__Proteobacteria	g__Ochrobactrum	0.00929	0.0007543	0.006553	0.000495	0.000183	0.002329	-0.00327	-0.00219	-0.00274
p__Proteobacteria	g__Methyloversatilis	0.01194	0.002376	0.009271	0.001836	0.01133	0.02909	-0.00447	-0.0008	-0.00267
p__Proteobacteria	g__Ferruginivarius	0.01241	0.001885	0.009754	0.001139	0.004586	0.01474	-0.00393	-0.00138	-0.00265
p__Proteobacteria	g__Labrys_f__Xanthobacteraceae	0.01447	0.001715	0.01183	0.00146	0.002827	0.01032	-0.00394	-0.00128	-0.00264
p__Proteobacteria	g__Marinicauda	0.008806	0.001298	0.006181	0.000719	0.000246	0.00248	-0.00349	-0.00181	-0.00263
p__Proteobacteria	g__Polymorphobacter	0.008027	0.0008523	0.00544	0.002677	0.004586	0.01474	-0.00408	-0.00068	-0.00259
p__Proteobacteria	g__Xenophilus	0.01111	0.001581	0.008581	0.001552	0.005795	0.0177	-0.0038	-0.00119	-0.00253
p__Proteobacteria	g__Pararhizobium	0.005994	0.001482	0.003472	0.000877	0.000769	0.004362	-0.00364	-0.00159	-0.00252
p__Proteobacteria	g__Rhodovastum	0.009016	0.001432	0.006501	0.000973	0.000769	0.004362	-0.00358	-0.00153	-0.00252

p__Proteobacteria	g__Vulgatibacter	0.0059	0.002089	0.003393	0.000392	0.002202	0.008721	-0.00371	-0.00126	-0.00251
p__Proteobacteria	g__unclassified_f__Porticoccaceae	0.007539	0.0007301	0.005055	0.00082	0.000183	0.002329	-0.00311	-0.00184	-0.00248
p__Proteobacteria	g__Achromobacter	0.02708	0.001688	0.02461	0.002646	0.03764	0.07496	-0.00432	-0.00043	-0.00246
p__Proteobacteria	g__Micavibrio	0.007516	0.001156	0.005083	0.000906	0.000583	0.003709	-0.00335	-0.00158	-0.00243
p__Proteobacteria	g__unclassified_f__Steroidobacterace	0.01066	0.001317	0.00823	0.00153	0.002827	0.01032	-0.00356	-0.0012	-0.00243
p__Proteobacteria	g__Robiginitomaculum	0.007046	0.001395	0.004695	0.000408	0.000246	0.00248	-0.00331	-0.00159	-0.00235
p__Proteobacteria	g__Desulfuromonas	0.01173	0.002188	0.009411	0.001065	0.01402	0.03427	-0.00382	-0.00091	-0.00232
p__Proteobacteria	g__Henriciella	0.0102	0.001675	0.007895	0.000706	0.001008	0.00518	-0.00341	-0.00128	-0.0023
p__Proteobacteria	g__Afifella	0.006215	0.0007258	0.00393	0.000543	0.000183	0.002329	-0.0028	-0.00178	-0.00229
p__Proteobacteria	g__Aurantimonas	0.008602	0.0006921	0.006337	0.001393	0.002202	0.008721	-0.00319	-0.00135	-0.00227
p__Proteobacteria	g__Acinetobacter	0.007936	0.0007351	0.005677	0.000896	0.000583	0.003709	-0.00286	-0.00149	-0.00226
p__Proteobacteria	g__Plasticicumulans	0.01403	0.000939	0.01179	0.001461	0.001008	0.00518	-0.00344	-0.00119	-0.00224
p__Proteobacteria	g__Pseudobacteriovorax	0.002866	0.001503	0.0006647	0.000222	0.000183	0.002329	-0.00321	-0.00139	-0.0022
p__Proteobacteria	g__unclassified_f__Rhodocyclaceae	0.02392	0.001905	0.02174	0.001127	0.01402	0.03427	-0.00343	-0.00077	-0.00219
p__Proteobacteria	g__unclassified_o__Pseudomonadales	0.01266	0.001739	0.01048	0.002323	0.01402	0.03427	-0.00385	-0.00037	-0.00217
p__Proteobacteria	g__Roseovarius	0.009693	0.0006177	0.007515	0.000988	0.00044	0.003169	-0.00288	-0.00151	-0.00218
p__Proteobacteria	g__Glycocaulis	0.006113	0.0009062	0.003977	0.000387	0.000246	0.00248	-0.00268	-0.00158	-0.00214
p__Proteobacteria	g__Zhengella	0.00397	0.001457	0.001854	0.00046	0.000769	0.004362	-0.003	-0.00122	-0.00212
p__Proteobacteria	g__unclassified_f__Parvularculaceae	0.00271	0.001495	0.0006281	0.000271	0.000183	0.002329	-0.00313	-0.00131	-0.00208
p__Proteobacteria	g__Oceanicoccus	0.004123	0.00108	0.002051	0.00058	0.000769	0.004362	-0.00278	-0.00141	-0.00207
p__Proteobacteria	g__Lautropia	0.007325	0.001059	0.005287	0.000633	0.001008	0.00518	-0.00274	-0.00135	-0.00204
p__Proteobacteria	g__Cohaesibacter	0.003007	0.0005635	0.000998	0.000177	0.000183	0.002329	-0.00237	-0.00165	-0.00201
p__Proteobacteria	g__unclassified_f__Woeseiaceae	0.02058	0.00218	0.01858	0.002288	0.03121	0.06485	-0.00392	-0.00018	-0.002
p__Proteobacteria	g__Gallionella	0.003967	0.0005604	0.001977	0.000473	0.000246	0.00248	-0.00242	-0.00155	-0.00199
p__Proteobacteria	g__Amaricoccus	0.006753	0.001449	0.00477	0.001812	0.03764	0.07496	-0.00331	-0.00055	-0.00198
p__Proteobacteria	g__Sulfitobacter	0.009395	0.001086	0.00742	0.00144	0.01402	0.03427	-0.00298	-0.00092	-0.00198
p__Proteobacteria	g__Methyloceanibacter	0.01301	0.001864	0.01104	0.001279	0.02575	0.05574	-0.0033	-0.00062	-0.00197
p__Proteobacteria	g__Pseudorhodobacter	0.005505	0.001429	0.003559	0.000949	0.003611	0.01236	-0.00303	-0.00095	-0.00195
p__Proteobacteria	g__Thalassospira	0.01365	0.001267	0.01172	0.002001	0.02113	0.04755	-0.0033	-0.00042	-0.00193
p__Proteobacteria	g__Desulfovibrio	0.01353	0.001093	0.0116	0.001002	0.002202	0.008721	-0.0028	-0.00113	-0.00193
p__Proteobacteria	g__Marinobacter	0.02071	0.002038	0.01878	0.00127	0.03121	0.06485	-0.00368	-0.00069	-0.00193
p__Proteobacteria	g__Haliea	0.01046	0.002206	0.008533	0.000784	0.01726	0.0402	-0.00351	-0.00068	-0.00193
p__Proteobacteria	g__Plesiocystis	0.004686	0.001782	0.00277	0.000736	0.009108	0.02457	-0.00302	-0.00086	-0.00192
p__Proteobacteria	g__Oceanicola	0.003486	0.0004859	0.001616	0.000281	0.000183	0.002329	-0.00221	-0.00153	-0.00187
p__Proteobacteria	g__Croceibacterium	0.002637	0.0005726	0.0007796	0.00029	0.000183	0.002329	-0.00226	-0.00145	-0.00186
p__Proteobacteria	g__Parvibaculum	0.01471	0.0006916	0.01287	0.00058	0.000246	0.00248	-0.00235	-0.00132	-0.00184
p__Proteobacteria	g__Limnobacter	0.006848	0.001283	0.005021	0.000895	0.007285	0.02069	-0.00277	-0.00092	-0.00183
p__Proteobacteria	g__Thiohalocapsa	0.005979	0.0009861	0.004158	0.000562	0.000183	0.002329	-0.00258	-0.00126	-0.00182
p__Proteobacteria	g__Methylosinus	0.007406	0.001011	0.005591	0.00071	0.000769	0.004362	-0.00255	-0.00107	-0.00182
p__Proteobacteria	g__Ahrensia	0.00476	0.0009051	0.002949	0.000372	0.000183	0.002329	-0.00243	-0.00129	-0.00181
p__Proteobacteria	g__Pseudacidovorax	0.004121	0.0008288	0.002352	0.000948	0.001706	0.007373	-0.00249	-0.00099	-0.00177
p__Proteobacteria	g__Oceanicaulis	0.003912	0.001059	0.002157	0.000365	0.00044	0.003169	-0.00243	-0.00106	-0.00176
p__Proteobacteria	g__Candidatus_Contendobacter	0.008299	0.001166	0.00656	0.000973	0.002827	0.01032	-0.00264	-0.00087	-0.00174
p__Proteobacteria	g__Gluconobacter	0.002678	0.0003598	0.001005	0.00017	0.000183	0.002329	-0.00192	-0.00145	-0.00167
p__Proteobacteria	g__unclassified_o__Bradymonadales	0.003721	0.001172	0.002076	0.000346	0.001008	0.00518	-0.00239	-0.00093	-0.00165
p__Proteobacteria	g__Kordiimonas	0.005758	0.0006458	0.004116	0.000677	0.000583	0.003709	-0.0022	-0.0011	-0.00164
p__Proteobacteria	g__Crenobacter	0.003455	0.0005434	0.001821	0.000171	0.000183	0.002329	-0.00196	-0.0013	-0.00163
p__Proteobacteria	g__Pseudooceanicola	0.005099	0.0007181	0.00347	0.000752	0.000769	0.004362	-0.00227	-0.00103	-0.00163

p__Proteobacteria	g__Rubellimicrobium	0.005136	0.0004169	0.003521	0.000486	0.000183	0.002329	-0.002	-0.00124	-0.00161
p__Proteobacteria	g__Oligoflexus	0.004029	0.001574	0.00242	0.000374	0.003611	0.01236	-0.00272	-0.0007	-0.00161
p__Proteobacteria	g__Neorhizobium	0.01204	0.0007965	0.01044	0.000596	0.00044	0.003169	-0.00216	-0.001	-0.0016
p__Proteobacteria	g__Pseudenhygromyxa	0.004329	0.001907	0.002736	0.000658	0.04515	0.08665	-0.00285	-0.00048	-0.00159
p__Proteobacteria	g__Belnapia	0.008869	0.001182	0.007279	0.001176	0.01402	0.03427	-0.00258	-0.00053	-0.00159
p__Proteobacteria	g__Desulfobacterium	0.005465	0.001521	0.003907	0.001482	0.03764	0.07496	-0.00286	-0.00032	-0.00156
p__Proteobacteria	g__Roseiarcus	0.005958	0.0006607	0.004411	0.000686	0.001008	0.00518	-0.00209	-0.00099	-0.00155
p__Proteobacteria	g__Gemmobacter	0.005062	0.0009837	0.003515	0.000585	0.001315	0.006216	-0.00224	-0.00086	-0.00155
p__Proteobacteria	g__Thalassomonas	0.001978	0.000673	0.0004633	9.29E-05	0.000183	0.002329	-0.00194	-0.00112	-0.00151
p__Proteobacteria	g__unclassified_c__Zetaproteobacteria	0.007283	0.001258	0.005772	0.000757	0.002827	0.01032	-0.00244	-0.00072	-0.00151
p__Proteobacteria	g__'Geomonas'_Xu_et_al._2019	0.002375	0.0005347	0.000865	0.000149	0.000183	0.002329	-0.00183	-0.00118	-0.00151
p__Proteobacteria	g__Siccirubricoccus	0.007594	0.0008315	0.006102	0.000521	0.000769	0.004362	-0.00207	-0.00094	-0.00149
p__Proteobacteria	g__Acidihalobacter	0.003948	0.0007271	0.002471	0.000489	0.000246	0.00248	-0.00201	-0.00102	-0.00148
p__Proteobacteria	g__Pseudohaliea	0.002974	0.0008225	0.001521	0.000519	0.000769	0.004362	-0.00199	-0.00084	-0.00145
p__Proteobacteria	g__Silicimonas	0.002738	0.0003513	0.001338	8.19E-05	0.000183	0.002329	-0.00161	-0.00119	-0.0014
p__Proteobacteria	g__Desulfopila	0.002824	0.000335	0.001426	0.000344	0.000183	0.002329	-0.00169	-0.00111	-0.0014
p__Proteobacteria	g__Methylotenera	0.004304	0.0007676	0.002944	0.000581	0.001008	0.00518	-0.00192	-0.00079	-0.00136
p__Proteobacteria	g__Pseudogulbenkiania	0.004647	0.000702	0.003321	0.000428	0.001008	0.00518	-0.0018	-0.0008	-0.00133
p__Proteobacteria	g__Crenothrix	0.002541	0.0003646	0.001242	0.000479	0.00044	0.003169	-0.00165	-0.00092	-0.0013
p__Proteobacteria	g__unclassified_o__Rickettsiales	0.004208	0.0007868	0.002923	0.00054	0.001315	0.006216	-0.00186	-0.00072	-0.00128
p__Proteobacteria	g__Thiocystis	0.002106	0.0008235	0.0008225	0.000218	0.000246	0.00248	-0.0018	-0.00079	-0.00128
p__Proteobacteria	g__Inmirania	0.005299	0.0009251	0.004041	0.000459	0.007285	0.02069	-0.00187	-0.00062	-0.00126
p__Proteobacteria	g__Boseongicola	0.004039	0.0006711	0.002789	0.000743	0.002827	0.01032	-0.00186	-0.00069	-0.00125
p__Proteobacteria	g__Thioflavicoccus	0.003857	0.0005672	0.002609	0.000462	0.00033	0.002769	-0.00169	-0.00082	-0.00125
p__Proteobacteria	g__Lutibaculum	0.003647	0.0003424	0.002401	0.000355	0.000183	0.002329	-0.00153	-0.00095	-0.00125
p__Proteobacteria	g__unclassified_f__Methylothermacea	0.002408	0.0003465	0.001174	0.000414	0.000246	0.00248	-0.00159	-0.0009	-0.00123
p__Proteobacteria	g__Thiomargarita	0.003887	0.0006842	0.002657	0.000484	0.00044	0.003169	-0.00175	-0.00078	-0.00123
p__Proteobacteria	g__Maricaulis	0.004557	0.0008161	0.003333	0.001154	0.007285	0.02069	-0.00199	-0.00034	-0.00123
p__Proteobacteria	g__unclassified_f__Geobacteraceae	0.006082	0.001154	0.004861	0.000583	0.03764	0.07496	-0.002	-0.00048	-0.00122
p__Proteobacteria	g__Candidatus_Kentron	0.007919	0.0005592	0.006718	0.000859	0.007285	0.02069	-0.00186	-0.00059	-0.0012
p__Proteobacteria	g__Derxia	0.003248	0.0003698	0.00205	0.000409	0.000183	0.002329	-0.00153	-0.00087	-0.0012
p__Proteobacteria	g__Denitratisoma	0.002919	0.0005951	0.001729	0.000221	0.000183	0.002329	-0.00156	-0.00082	-0.00119
p__Proteobacteria	g__unclassified_o__Thiotrichales	0.008888	0.001034	0.007701	0.000781	0.01133	0.02909	-0.002	-0.00041	-0.00119
p__Proteobacteria	g__Thiohalobacter	0.004395	0.0005039	0.003218	0.000706	0.000769	0.004362	-0.00171	-0.00063	-0.00118
p__Proteobacteria	g__Defluviimonas	0.004568	0.0003589	0.003392	0.001122	0.02113	0.04755	-0.00184	-0.00042	-0.00118
p__Proteobacteria	g__Salinisphaera	0.003853	0.001341	0.002695	0.000415	0.007285	0.02069	-0.00202	-0.00039	-0.00116
p__Proteobacteria	g__Chitinimonas	0.009673	0.000831	0.008522	0.000695	0.009108	0.02457	-0.00182	-0.00051	-0.00115
p__Proteobacteria	g__Spirobacillus	0.001283	0.0006953	0.0001543	0.000127	0.000183	0.002329	-0.00158	-0.00073	-0.00113
p__Proteobacteria	g__unclassified_f__Bacteriovoracacea	0.001924	0.0006198	0.0008015	0.000224	0.00033	0.002769	-0.00152	-0.00074	-0.00112
p__Proteobacteria	g__Roseibaca	0.001621	0.0003391	0.0005196	0.000278	0.000183	0.002329	-0.00134	-0.00084	-0.0011
p__Proteobacteria	g__Oceanococcus	0.003812	0.001089	0.002732	0.000701	0.01133	0.02909	-0.00181	-0.00031	-0.00108
p__Proteobacteria	g__Malikia	0.002642	0.000817	0.001564	0.000666	0.01133	0.02909	-0.00174	-0.00044	-0.00108
p__Proteobacteria	g__unclassified_o__Syntrophobacteral	0.002917	0.000442	0.001846	0.000195	0.000183	0.002329	-0.00137	-0.00078	-0.00107
p__Proteobacteria	g__Sulfurirhabdus	0.002391	0.0003268	0.001324	0.000441	0.000246	0.00248	-0.00142	-0.00074	-0.00107
p__Proteobacteria	g__Abyssibacter	0.001838	0.001515	0.0007927	0.000569	0.02113	0.04755	-0.00216	-0.0002	-0.00105
p__Proteobacteria	g__Salaquimonas	0.001888	0.0005601	0.000843	0.000112	0.000183	0.002329	-0.00137	-0.00072	-0.00105
p__Proteobacteria	g__Marinicella	0.002576	0.0007866	0.001532	0.00072	0.005795	0.0177	-0.00168	-0.00034	-0.00104
p__Proteobacteria	g__unclassified_f__Pseudomonadacea	0.001761	0.0003418	0.0007173	0.000167	0.000183	0.002329	-0.00127	-0.00082	-0.00104

p__Proteobacteria	g__Alicycliphilus	0.004969	0.00113	0.003945	0.001002	0.03764	0.07496	-0.00195	-0.00015	-0.00102
p__Proteobacteria	g__unclassified_f__Sterolibacteriaceae	0.002166	0.0004878	0.001143	0.000139	0.000183	0.002329	-0.0013	-0.00071	-0.00102
p__Proteobacteria	g__Tepidiphilus	0.002913	0.0005363	0.001892	0.000241	0.001008	0.00518	-0.00135	-0.00065	-0.00102
p__Proteobacteria	g__Mariprofundus	0.001734	0.0007075	0.0007237	0.000177	0.00044	0.003169	-0.00149	-0.00061	-0.00101
p__Proteobacteria	g__Rhodobium_f__Rhodobiaceae	0.003051	0.0003507	0.002051	0.000436	0.00044	0.003169	-0.00135	-0.00069	-0.001
p__Proteobacteria	g__Stappia	0.01011	0.000917	0.009111	0.000859	0.02575	0.05574	-0.0017	-0.00026	-0.001
p__Proteobacteria	g__Thiothrix	0.003133	0.0002964	0.002139	0.000278	0.000183	0.002329	-0.00122	-0.00076	-0.00099
p__Proteobacteria	g__Dethiosulfatarculus	0.002067	0.0006987	0.001078	0.000306	0.007285	0.02069	-0.00145	-0.00055	-0.00099
p__Proteobacteria	g__Oharaeibacter	0.003372	0.0005121	0.002402	0.000744	0.009108	0.02457	-0.00149	-0.00044	-0.00097
p__Proteobacteria	g__Hansschlegelia	0.005445	0.000606	0.00448	0.00128	0.009108	0.02457	-0.00175	-0.00014	-0.00097
p__Proteobacteria	g__unclassified_o__Sneathiellales	0.001665	0.0003214	0.0007033	0.000201	0.000183	0.002329	-0.00119	-0.00074	-0.00096
p__Proteobacteria	g__Methylovorus	0.002135	0.0003207	0.001198	0.000411	0.001008	0.00518	-0.00123	-0.00061	-0.00094
p__Proteobacteria	g__Hahella	0.003519	0.0004055	0.002591	0.000378	0.00044	0.003169	-0.00125	-0.00057	-0.00093
p__Proteobacteria	g__Dissulfurirhabdus	0.001916	0.000402	0.001007	0.000213	0.000183	0.002329	-0.00118	-0.00066	-0.00091
p__Proteobacteria	g__Pseudovibrio	0.002212	0.0002437	0.001305	0.000153	0.000183	0.002329	-0.00108	-0.00073	-0.00091
p__Proteobacteria	g__Undibacterium	0.005131	0.0004762	0.004237	0.000557	0.002827	0.01032	-0.00134	-0.00046	-0.00089
p__Proteobacteria	g__Aeromonas	0.004135	0.0006056	0.003258	0.000452	0.004586	0.01474	-0.00134	-0.0004	-0.00088
p__Proteobacteria	g__Methyloligella	0.002724	0.0003695	0.001852	0.000432	0.001315	0.006216	-0.0012	-0.00056	-0.00087
p__Proteobacteria	g__Silanimonas	0.001573	0.0009449	0.0007039	0.000547	0.01402	0.03427	-0.0016	-0.00026	-0.00087
p__Proteobacteria	g__Roseospirillum	0.002794	0.0003807	0.001928	0.000244	0.00033	0.002769	-0.00114	-0.0006	-0.00087
p__Proteobacteria	g__Desulfotignum	0.001941	0.000585	0.001079	0.00045	0.002827	0.01032	-0.00133	-0.00042	-0.00086
p__Proteobacteria	g__Roseibium	0.003629	0.0003007	0.002769	0.000607	0.003611	0.01236	-0.00127	-0.00046	-0.00086
p__Proteobacteria	g__Maribius	0.001361	0.0004057	0.0005023	0.000215	0.000183	0.002329	-0.00112	-0.00061	-0.00086
p__Proteobacteria	g__Methyloprofundus	0.001529	0.000335	0.0006816	0.000142	0.000183	0.002329	-0.00107	-0.00065	-0.00085
p__Proteobacteria	g__Fulvimarina	0.002992	0.000542	0.002149	0.00033	0.002202	0.008721	-0.00124	-0.00047	-0.00084
p__Proteobacteria	g__Arsenicitalea	0.002171	0.0008193	0.001329	0.000364	0.007285	0.02069	-0.00137	-0.00034	-0.00084
p__Proteobacteria	g__unclassified_f__Gallionellaceae	0.00507	0.0005272	0.004232	0.000543	0.01133	0.02909	-0.00132	-0.0004	-0.00084
p__Proteobacteria	g__Aquidulcibacter	0.001577	0.0003634	0.0007568	0.000197	0.00033	0.002769	-0.00106	-0.00057	-0.00082
p__Proteobacteria	g__Thiolapillus	0.002062	0.000514	0.001248	0.000489	0.004586	0.01474	-0.00123	-0.00044	-0.00081
p__Proteobacteria	g__Ponticaulis	0.002595	0.0004413	0.001786	0.000428	0.001706	0.007373	-0.0012	-0.00044	-0.00081
p__Proteobacteria	g__Chromohalobacter	0.001548	0.0004397	0.00074	0.000176	0.00033	0.002769	-0.00109	-0.00054	-0.00081
p__Proteobacteria	g__unclassified_o__Holosporales	0.001844	0.0006655	0.001044	0.000371	0.001008	0.00518	-0.00126	-0.00037	-0.0008
p__Proteobacteria	g__Alteromonas	0.005589	0.0004173	0.004811	0.000389	0.002827	0.01032	-0.00114	-0.00044	-0.00078
p__Proteobacteria	g__Salinicola	0.001966	0.0003392	0.001189	0.00028	0.000769	0.004362	-0.00103	-0.0005	-0.00078
p__Proteobacteria	g__Uliginosibacterium	0.002338	0.0004411	0.001563	0.000497	0.004586	0.01474	-0.00117	-0.00038	-0.00078
p__Proteobacteria	g__Kofleria	0.001329	0.001026	0.0005628	0.000188	0.007285	0.02069	-0.00148	-0.00025	-0.00077
p__Proteobacteria	g__Casimicrobium	0.002778	0.000489	0.002019	0.000575	0.01133	0.02909	-0.00123	-0.00034	-0.00076
p__Proteobacteria	g__Oleiphilus	0.003726	0.0005228	0.002973	0.000802	0.01402	0.03427	-0.00131	-0.00018	-0.00075
p__Proteobacteria	g__Marinovum	0.001766	0.0004361	0.001036	0.000319	0.001706	0.007373	-0.00104	-0.00044	-0.00073
p__Proteobacteria	g__Breoghania	0.003114	0.0003903	0.002403	0.000459	0.007285	0.02069	-0.00106	-0.00035	-0.00071
p__Proteobacteria	g__Teredinibacter	0.0008553	0.0007697	0.0001465	4.31E-05	0.000583	0.003709	-0.0012	-0.00029	-0.00071
p__Proteobacteria	g__Dichotomicrobium	0.001558	0.000398	0.0008539	0.000264	0.002202	0.008721	-0.00097	-0.00043	-0.0007
p__Proteobacteria	g__Albimonas	0.002831	0.000532	0.002136	0.000491	0.009108	0.02457	-0.00109	-0.00027	-0.0007
p__Proteobacteria	g__Azovibrio	0.00215	0.0003396	0.001455	0.000239	0.001008	0.00518	-0.00092	-0.00045	-0.00069
p__Proteobacteria	g__unclassified_f__Neisseriaceae	0.0009627	0.0003056	0.0002703	8.07E-05	0.000183	0.002329	-0.00089	-0.00052	-0.00069
p__Proteobacteria	g__Arenicella	0.0008302	0.0001839	0.0001415	4.91E-05	0.000183	0.002329	-0.0008	-0.00057	-0.00069
p__Proteobacteria	g__Methylobrevis	0.002082	0.0003009	0.001399	0.000181	0.00033	0.002769	-0.00089	-0.00048	-0.00068
p__Proteobacteria	g__Delftia	0.003993	0.0004417	0.003311	0.000391	0.002827	0.01032	-0.00104	-0.00036	-0.00068

p__Proteobacteria	g__Rhodovibrio	0.002505	0.0005871	0.001823	0.000219	0.009108	0.02457	-0.00104	-0.0003	-0.00068
p__Proteobacteria	g__Orrella	0.002182	0.0004599	0.001501	0.000273	0.005795	0.0177	-0.00099	-0.00037	-0.00068
p__Proteobacteria	g__Thalassotalea	0.001964	0.0004972	0.001291	0.000453	0.009108	0.02457	-0.00107	-0.00026	-0.00067
p__Proteobacteria	g__Photorhabdus	0.001516	0.0004315	0.000843	0.00035	0.003611	0.01236	-0.00102	-0.00036	-0.00067
p__Proteobacteria	g__Pannonibacter	0.003148	0.0002221	0.002489	0.000386	0.001008	0.00518	-0.00092	-0.0004	-0.00066
p__Proteobacteria	g__Oceanicella	0.002376	0.0002686	0.001717	0.000363	0.00044	0.003169	-0.00091	-0.00038	-0.00066
p__Proteobacteria	g__Ferrovum	0.00218	0.0003468	0.001522	0.000332	0.002202	0.008721	-0.00097	-0.00037	-0.00066
p__Proteobacteria	g__Desulfospira	0.0008539	0.0006495	0.000203	0.000132	0.001315	0.006216	-0.00107	-0.00033	-0.00065
p__Proteobacteria	g__Melaminivora	0.001133	0.0003108	0.0004867	0.000264	0.000769	0.004362	-0.00087	-0.00038	-0.00065
p__Proteobacteria	g__Diaphorobacter	0.002368	0.0002598	0.001726	0.000475	0.002827	0.01032	-0.00095	-0.00028	-0.00064
p__Proteobacteria	g__Agarilytica	0.001042	0.0003451	0.0004003	0.000153	0.000246	0.00248	-0.00088	-0.00044	-0.00064
p__Proteobacteria	g__Oceanimonas	0.001164	0.0004023	0.0005418	0.000192	0.000769	0.004362	-0.00091	-0.00038	-0.00062
p__Proteobacteria	g__Roseinatronobacter	0.001185	0.0004116	0.0005699	0.000229	0.001706	0.007373	-0.00089	-0.00033	-0.00062
p__Proteobacteria	g__Verminephrobacter	0.001578	0.0003678	0.0009649	0.000262	0.001315	0.006216	-0.00088	-0.00034	-0.00061
p__Proteobacteria	g__Thiobaca	0.001384	0.000237	0.0007711	0.000185	0.000183	0.002329	-0.0008	-0.00044	-0.00061
p__Proteobacteria	g__Amylibacter	0.00157	0.0003338	0.0009578	0.000153	0.000583	0.003709	-0.00082	-0.0004	-0.00061
p__Proteobacteria	g__Tepidicella	0.001416	0.0003019	0.0008091	0.000324	0.001315	0.006216	-0.00087	-0.00036	-0.00061
p__Proteobacteria	g__Mangrovicoccus	0.001539	0.0003075	0.0009338	0.000173	0.00033	0.002769	-0.0008	-0.0004	-0.00061
p__Proteobacteria	g__Rhodocyclus	0.002138	0.0004311	0.001542	0.000272	0.003611	0.01236	-0.0009	-0.00029	-0.0006
p__Proteobacteria	g__Chitiniphilus	0.001785	0.0003613	0.001199	0.000297	0.003611	0.01236	-0.00087	-0.00032	-0.00059
p__Proteobacteria	g__Pleomorphomonas	0.005656	0.0007815	0.005085	0.000563	0.03121	0.06485	-0.00114	3.79E-05	-0.00057
p__Proteobacteria	g__Cronobacter	0.001019	0.0005762	0.0004482	0.000206	0.007285	0.02069	-0.00096	-0.00025	-0.00057
p__Proteobacteria	g__Chitinivorax	0.002336	0.0004655	0.001766	0.000282	0.007285	0.02069	-0.00088	-0.00025	-0.00057
p__Proteobacteria	g__Endozoicomonas	0.001921	0.0003426	0.001357	0.000142	0.001315	0.006216	-0.00077	-0.00034	-0.00056
p__Proteobacteria	g__Candidatus_Electronema	0.0007416	0.0003823	0.00019	6.34E-05	0.00033	0.002769	-0.00079	-0.00033	-0.00055
p__Proteobacteria	g__Amorphus	0.001897	0.0002038	0.001352	0.00022	0.00033	0.002769	-0.00072	-0.00035	-0.00055
p__Proteobacteria	g__Oceanibium	0.00102	0.0002948	0.0004809	0.000344	0.002202	0.008721	-0.00081	-0.00026	-0.00054
p__Proteobacteria	g__unclassified_o__Acidithiobacillale	0.00331	0.0004208	0.002776	0.00052	0.03121	0.06485	-0.00091	-0.00014	-0.00053
p__Proteobacteria	g__unclassified_o__Oligoflexales	0.0007139	0.0002277	0.0001802	7.31E-05	0.000183	0.002329	-0.00068	-0.00039	-0.00053
p__Proteobacteria	g__Oricola	0.001436	0.000231	0.0009028	0.000185	0.000246	0.00248	-0.00072	-0.00036	-0.00053
p__Proteobacteria	g__Byssovorax	0.001067	0.000196	0.0005343	0.000142	0.000246	0.00248	-0.00067	-0.00039	-0.00053
p__Proteobacteria	g__Halospina	0.001225	0.0003465	0.0006931	0.00015	0.000769	0.004362	-0.00075	-0.0003	-0.00053
p__Proteobacteria	g__Celeribacter	0.002063	0.0003168	0.001536	0.000297	0.003611	0.01236	-0.00077	-0.00026	-0.00053
p__Proteobacteria	g__Limimaricola	0.001245	0.0003771	0.00072	0.000122	0.000583	0.003709	-0.00078	-0.0003	-0.00053
p__Proteobacteria	g__unclassified_o__Nevskiales	0.001045	0.0003845	0.0005291	0.000192	0.001315	0.006216	-0.00081	-0.00027	-0.00052
p__Proteobacteria	g__Lonsdalea	0.0006992	0.0003911	0.0001836	7.92E-05	0.00033	0.002769	-0.00075	-0.00029	-0.00052
p__Proteobacteria	g__Candidatus_Dactylopiibacterium	0.0007289	0.0002597	0.0002149	7.03E-05	0.000183	0.002329	-0.00068	-0.00036	-0.00051
p__Proteobacteria	g__unclassified_o__Alteromonadales	0.0006955	0.0006989	0.0001917	6.10E-05	0.000769	0.004362	-0.00097	-0.0002	-0.0005
p__Proteobacteria	g__Rhodothalassium	0.00115	0.0001341	0.0006501	0.000184	0.000246	0.00248	-0.00064	-0.00036	-0.0005
p__Proteobacteria	g__Thermithiobacillus	0.007447	0.0004973	0.006948	0.000624	0.02113	0.04755	-0.00097	-1.89E-05	-0.0005
p__Proteobacteria	g__Youhaiella	0.001421	0.0005329	0.0009273	0.000176	0.03121	0.06485	-0.00084	-0.00017	-0.00049
p__Proteobacteria	g__Sulfurimonas	0.000785	0.0004623	0.0002939	6.41E-05	0.001315	0.006216	-0.00078	-0.00024	-0.00049
p__Proteobacteria	g__Serpentinomonas	0.0007988	0.0002155	0.0003099	0.000142	0.00044	0.003169	-0.00064	-0.00033	-0.00049
p__Proteobacteria	g__Methylocucumis	0.0008391	0.0001076	0.0003537	7.40E-05	0.000183	0.002329	-0.00057	-0.0004	-0.00049
p__Proteobacteria	g__Minwuia	0.002722	0.0002567	0.002238	0.000584	0.03121	0.06485	-0.00084	-0.00013	-0.00048
p__Proteobacteria	g__Halobacteriovorax	0.001432	0.0005811	0.0009521	0.000396	0.01133	0.02909	-0.00088	-8.79E-05	-0.00048
p__Proteobacteria	g__Limimonas	0.001915	0.0002722	0.001443	0.000316	0.007285	0.02069	-0.00071	-0.00024	-0.00047
p__Proteobacteria	g__Paucimonas	0.001441	0.0003254	0.0009702	0.000184	0.001315	0.006216	-0.0007	-0.00025	-0.00047

p__Proteobacteria	g__Hankyongella	0.001338	0.0002774	0.0008705	0.000211	0.001706	0.007373	-0.00067	-0.00027	-0.00047
p__Proteobacteria	g__Palleronia	0.0009341	0.0001925	0.0004684	0.000169	0.00033	0.002769	-0.00062	-0.00031	-0.00047
p__Proteobacteria	g__Aerosticca	0.001041	0.0002785	0.0005755	0.000243	0.001008	0.00518	-0.00069	-0.00026	-0.00047
p__Proteobacteria	g__Brucella	0.003041	0.0002827	0.002577	0.000254	0.001008	0.00518	-0.0007	-0.00023	-0.00046
p__Proteobacteria	g__Thermochromatium	0.0008389	0.000267	0.0003765	0.00012	0.00033	0.002769	-0.00065	-0.0003	-0.00046
p__Proteobacteria	g__Desulforhopalus	0.001645	0.0002843	0.001184	0.000252	0.003611	0.01236	-0.0007	-0.00022	-0.00046
p__Proteobacteria	g__Epibacterium	0.001279	0.0004327	0.0008215	0.000376	0.01133	0.02909	-0.00078	-0.00011	-0.00046
p__Proteobacteria	g__Tepidicaulis	0.002779	0.000404	0.002327	0.000197	0.003611	0.01236	-0.00073	-0.00019	-0.00045
p__Proteobacteria	g__Desulfobacter	0.001171	0.0001362	0.0007248	0.000169	0.00044	0.003169	-0.00058	-0.00032	-0.00045
p__Proteobacteria	g__Kerstersia	0.001061	0.0002512	0.000616	0.000232	0.002202	0.008721	-0.00063	-0.00023	-0.00044
p__Proteobacteria	g__Sulfuritortus	0.0009841	0.0001207	0.0005419	0.000184	0.00033	0.002769	-0.00057	-0.0003	-0.00044
p__Proteobacteria	g__Denitromonas	0.001332	0.0002821	0.0008923	0.000304	0.009108	0.02457	-0.00068	-0.0002	-0.00044
p__Proteobacteria	g__Granulosicoccus	0.001284	0.0004327	0.0008577	0.000186	0.009108	0.02457	-0.00072	-0.00016	-0.00043
p__Proteobacteria	g__Woodsholea	0.0004894	0.0002801	7.23E-05	2.72E-05	0.000183	0.002329	-0.00059	-0.00026	-0.00042
p__Proteobacteria	g__Loktanella	0.001244	0.000203	0.0008317	0.000121	0.00033	0.002769	-0.00056	-0.00027	-0.00041
p__Proteobacteria	g__Puniceibacterium	0.001257	0.0004562	0.0008485	0.000408	0.02113	0.04755	-0.00075	-1.61E-05	-0.00041
p__Proteobacteria	g__Sulfurisoma	0.0009009	0.0003548	0.0004928	0.000138	0.001315	0.006216	-0.00065	-0.0002	-0.00041
p__Proteobacteria	g__Sansalvadorimonas	0.0008288	0.0002899	0.0004212	0.000175	0.004586	0.01474	-0.00061	-0.0002	-0.00041
p__Proteobacteria	g__unclassified_f__Fastidiosibacterac	0.0004818	0.0003502	7.56E-05	2.61E-05	0.000183	0.002329	-0.00063	-0.00022	-0.00041
p__Proteobacteria	g__Kangiella	0.001804	0.0003581	0.001403	0.00027	0.01726	0.0402	-0.00065	-0.00013	-0.0004
p__Proteobacteria	g__Hwanghaeicola	0.0006467	0.000366	0.000247	9.13E-05	0.000769	0.004362	-0.00063	-0.0002	-0.0004
p__Proteobacteria	g__Marinibacterium	0.0005041	0.0005559	0.0001093	9.71E-05	0.001008	0.00518	-0.00078	-0.00014	-0.00039
p__Proteobacteria	g__Sideroxydans	0.0009867	0.000155	0.0005931	0.000264	0.005795	0.0177	-0.00056	-0.00021	-0.00039
p__Proteobacteria	g__Psychrobacter	0.0006531	0.0001802	0.0002604	6.76E-05	0.000183	0.002329	-0.0005	-0.00028	-0.00039
p__Proteobacteria	g__Providencia	0.0006133	0.0001075	0.0002257	6.34E-05	0.000183	0.002329	-0.00046	-0.00032	-0.00039
p__Proteobacteria	g__Monaibacterium	0.0004929	5.65E-05	0.0001088	4.62E-05	0.000183	0.002329	-0.00042	-0.00034	-0.00038
p__Proteobacteria	g__Actibacterium	0.0005879	0.0001714	0.0002049	3.31E-05	0.000183	0.002329	-0.00048	-0.00028	-0.00038
p__Proteobacteria	g__Neomegalonema	0.0006007	0.0001864	0.0002177	7.89E-05	0.000183	0.002329	-0.00052	-0.00027	-0.00038
p__Proteobacteria	g__Deferrisoma	0.001129	0.0002738	0.0007466	0.000192	0.004586	0.01474	-0.00057	-0.0002	-0.00038
p__Proteobacteria	g__Amphritea	0.0008549	0.0001642	0.0004737	0.000113	0.000183	0.002329	-0.00051	-0.00026	-0.00038
p__Proteobacteria	g__Salinihabitans	0.000772	0.0001625	0.0003932	0.000143	0.00044	0.003169	-0.00049	-0.00024	-0.00038
p__Proteobacteria	g__Catenovulum	0.000604	0.0001845	0.0002265	3.02E-05	0.000183	0.002329	-0.00049	-0.00027	-0.00038
p__Proteobacteria	g__Yangia	0.001184	0.0001984	0.0008094	0.00017	0.002827	0.01032	-0.00052	-0.00021	-0.00037
p__Proteobacteria	g__Saccharospirillum	0.0008279	0.0003687	0.0004546	9.24E-05	0.000583	0.003709	-0.00063	-0.00019	-0.00037
p__Proteobacteria	g__unclassified_o__Legionellales	0.001135	0.0002957	0.0007647	0.000283	0.003611	0.01236	-0.00063	-0.00013	-0.00037
p__Proteobacteria	g__Candidatus_Methylospira	0.001391	0.0002447	0.001021	0.000361	0.02113	0.04755	-0.00065	-9.92E-05	-0.00037
p__Proteobacteria	g__Halovulum	0.002337	0.0001678	0.001968	0.000326	0.01726	0.0402	-0.00056	-0.00015	-0.00037
p__Proteobacteria	g__Pelagibaca	0.0007533	0.0002044	0.000389	0.000166	0.002202	0.008721	-0.00053	-0.0002	-0.00036
p__Proteobacteria	g__unclassified_f__Rickettsiaceae	0.000398	0.0003251	3.37E-05	2.17E-05	0.000182	0.002329	-0.00057	-0.00019	-0.00036
p__Proteobacteria	g__Castellaniella	0.0007211	0.0001447	0.0003571	7.28E-05	0.000183	0.002329	-0.00047	-0.00027	-0.00036
p__Proteobacteria	g__Desulfoglaeba	0.0007727	0.0002088	0.0004094	0.000159	0.001315	0.006216	-0.00051	-0.00021	-0.00036
p__Proteobacteria	g__Haematobacter	0.0006941	0.0001574	0.0003361	8.09E-05	0.000183	0.002329	-0.00047	-0.00025	-0.00036
p__Proteobacteria	g__unclassified_f__Wenzhouxiangella	0.0007492	0.0003614	0.0003915	0.000157	0.01402	0.03427	-0.00059	-0.00013	-0.00036
p__Proteobacteria	g__Rickettsia	0.001279	0.000275	0.0009223	0.000267	0.01726	0.0402	-0.00059	-0.00014	-0.00036
p__Proteobacteria	g__Halothiobacillus	0.001246	0.0001979	0.0008968	0.000225	0.004586	0.01474	-0.00052	-0.00017	-0.00035
p__Proteobacteria	g__Candidatus_Magnetaquicoccus	0.0005895	0.0004083	0.0002411	6.63E-05	0.002202	0.008721	-0.00062	-0.00013	-0.00035
p__Proteobacteria	g__Hellea	0.001077	0.0003223	0.0007307	0.000275	0.04515	0.08665	-0.00059	-0.0001	-0.00035
p__Proteobacteria	g__Guyparkeria	0.0003916	9.45E-05	4.75E-05	2.63E-05	0.000183	0.002329	-0.0004	-0.00029	-0.00034

p__Proteobacteria	g__Allochromatium	0.001494	0.0002805	0.001158	0.000181	0.01726	0.0402	-0.00053	-0.00013	-0.00034
p__Proteobacteria	g__Thalassococcus	0.0009562	0.0001329	0.0006227	0.000129	0.00044	0.003169	-0.00044	-0.00022	-0.00033
p__Proteobacteria	g__unclassified_f__Oceanospirillaceae	0.001138	0.0002263	0.0008065	0.000164	0.007285	0.02069	-0.00049	-0.00017	-0.00033
p__Proteobacteria	g__Lacimicrobium	0.0004059	0.0001271	7.90E-05	2.94E-05	0.000183	0.002329	-0.0004	-0.00025	-0.00033
p__Proteobacteria	g__Sulfurovum	0.0006296	0.0001503	0.0003032	0.000133	0.000769	0.004362	-0.00044	-0.00021	-0.00033
p__Proteobacteria	g__Extensimonas	0.0008833	0.0002015	0.0005602	0.000333	0.007285	0.02069	-0.00053	-7.59E-05	-0.00032
p__Proteobacteria	g__Ponticoccus_f__Rhodobacteraceae	0.000654	0.0001277	0.0003336	0.00011	0.00044	0.003169	-0.00041	-0.00022	-0.00032
p__Proteobacteria	g__Franconibacter	0.0007644	0.0002287	0.0004449	0.000151	0.004586	0.01474	-0.00046	-0.00015	-0.00032
p__Proteobacteria	g__Umboniibacter	0.0004592	0.0001727	0.0001415	8.03E-05	0.00033	0.002769	-0.00043	-0.00021	-0.00032
p__Proteobacteria	g__Rhabdaerophilum	0.002056	0.0002096	0.001739	0.000254	0.01402	0.03427	-0.0005	-0.00011	-0.00032
p__Proteobacteria	g__Sinobacterium	0.0006601	0.000148	0.000351	4.94E-05	0.000183	0.002329	-0.00041	-0.00023	-0.00031
p__Proteobacteria	g__Moraxella	0.0004741	0.0001712	0.0001685	6.09E-05	0.000246	0.00248	-0.00041	-0.0002	-0.00031
p__Proteobacteria	g__Martelella	0.001683	0.0002795	0.001379	0.000138	0.009108	0.02457	-0.00048	-0.00012	-0.0003
p__Proteobacteria	g__Tolumonas	0.0003956	0.0001673	9.16E-05	4.64E-05	0.00033	0.002769	-0.00042	-0.00022	-0.0003
p__Proteobacteria	g__Iodobacter	0.000999	0.0003299	0.0006996	0.000209	0.02113	0.04755	-0.00055	-7.00E-05	-0.0003
p__Proteobacteria	g__Bartonella	0.0006438	0.0002149	0.0003466	0.000237	0.01726	0.0402	-0.00049	-0.0001	-0.0003
p__Proteobacteria	g__Geopsychrobacter	0.0007502	0.0003344	0.0004542	0.000428	0.02113	0.04755	-0.0006	6.12E-05	-0.0003
p__Proteobacteria	g__unclassified_c__Epsilonproteobact	0.001075	0.0002831	0.0007801	0.000344	0.04515	0.08665	-0.00054	-4.07E-05	-0.00029
p__Proteobacteria	g__Pontibaca	0.0005257	0.0002203	0.0002372	9.43E-05	0.001008	0.00518	-0.00044	-0.00016	-0.00029
p__Proteobacteria	g__Octadecabacter	0.00102	0.0001532	0.0007385	0.000358	0.01133	0.02909	-0.00048	-1.89E-05	-0.00028
p__Proteobacteria	g__Halorhodospira	0.0005457	0.0002375	0.0002644	0.000101	0.001008	0.00518	-0.00046	-0.00014	-0.00028
p__Proteobacteria	g__Rubritepida	0.002009	0.0002274	0.001732	0.000188	0.02575	0.05574	-0.00045	-8.95E-05	-0.00028
p__Proteobacteria	g__Candidatus_Magnetoglobus	0.0003865	0.0002452	0.0001123	4.17E-05	0.00033	0.002769	-0.00042	-0.00014	-0.00027
p__Proteobacteria	g__Candidatus_Fonsibacter	0.0006632	0.0002109	0.0003947	0.000115	0.005795	0.0177	-0.00039	-0.00013	-0.00027
p__Proteobacteria	g__Desulfocurvus	0.000584	0.0001896	0.0003177	8.91E-05	0.003611	0.01236	-0.0004	-0.00015	-0.00027
p__Proteobacteria	g__Litorimicrobium	0.0005707	0.0001648	0.0003092	0.000136	0.003611	0.01236	-0.00038	-0.00013	-0.00026
p__Proteobacteria	g__Mameliella	0.001019	0.0001299	0.0007606	0.000155	0.002202	0.008721	-0.00037	-0.00014	-0.00026
p__Proteobacteria	g__Haemophilus	0.0005471	0.0002068	0.0002897	7.99E-05	0.000583	0.003709	-0.0004	-0.00015	-0.00026
p__Proteobacteria	g__unclassified_o__Enterobacterales	0.0008177	0.0001666	0.0005614	0.000188	0.01133	0.02909	-0.0004	-0.00011	-0.00026
p__Proteobacteria	g__Aquaspirillum	0.0006027	0.0001711	0.0003535	0.000195	0.02575	0.05574	-0.00041	-0.0001	-0.00025
p__Proteobacteria	g__Albidovulum	0.0005694	0.0001121	0.0003222	0.000131	0.001706	0.007373	-0.00035	-0.00014	-0.00025
p__Proteobacteria	g__Ephemeropotericola	0.0004086	0.0001708	0.0001619	0.000134	0.003611	0.01236	-0.00038	-0.00012	-0.00025
p__Proteobacteria	g__Peredibacter	0.0002747	0.0001461	2.80E-05	3.95E-05	0.000245	0.00248	-0.00034	-0.00015	-0.00025
p__Proteobacteria	g__Chitinolyticbacter	0.0006684	0.0001825	0.0004225	0.000149	0.007285	0.02069	-0.00038	-0.00011	-0.00025
p__Proteobacteria	g__unclassified_f__Pasteurellaceae	0.0002811	0.0001463	3.58E-05	3.06E-05	0.000183	0.002329	-0.00034	-0.00016	-0.00025
p__Proteobacteria	g__Thiomicrorhabdus	0.000417	7.69E-05	0.0001741	5.18E-05	0.000183	0.002329	-0.0003	-0.00019	-0.00024
p__Proteobacteria	g__Rhodospira	0.001873	0.0001839	0.001632	0.000233	0.01726	0.0402	-0.0004	-5.71E-05	-0.00024
p__Proteobacteria	g__Solirhodobacter	0.0007234	0.0001447	0.000483	7.71E-05	0.001315	0.006216	-0.00034	-0.00014	-0.00024
p__Proteobacteria	g__Salipiger	0.001621	0.0001716	0.001381	0.000386	0.04515	0.08665	-0.00046	1.05E-05	-0.00024
p__Proteobacteria	g__Gulbenkiania	0.001094	0.0002052	0.0008588	0.000178	0.04515	0.08665	-0.00039	-7.27E-05	-0.00024
p__Proteobacteria	g__Rhodophyticola	0.0005556	0.0001492	0.0003207	0.0001	0.001706	0.007373	-0.00034	-0.00013	-0.00023
p__Proteobacteria	g__Francisella	0.0003468	9.85E-05	0.0001126	4.71E-05	0.000183	0.002329	-0.0003	-0.00018	-0.00023
p__Proteobacteria	g__Alkanindiges	0.0003146	0.0001023	8.64E-05	9.91E-05	0.001008	0.00518	-0.00031	-0.00014	-0.00023
p__Proteobacteria	g__Desulfovermiculus	0.0003443	0.0001106	0.0001196	4.44E-05	0.000183	0.002329	-0.0003	-0.00016	-0.00022
p__Proteobacteria	g__Nitrincola	0.000847	0.0001658	0.0006249	0.000223	0.01402	0.03427	-0.00039	-6.48E-05	-0.00022
p__Proteobacteria	g__Varunaivibrio	0.001135	0.0001686	0.0009132	0.000168	0.01402	0.03427	-0.00036	-8.07E-05	-0.00022
p__Proteobacteria	g__Bowmanella	0.0002792	0.000216	6.53E-05	2.99E-05	0.00044	0.003169	-0.00036	-0.00011	-0.00021
p__Proteobacteria	g__Saezia	0.0005214	0.0001135	0.0003093	4.97E-05	0.000183	0.002329	-0.0003	-0.00014	-0.00021

p__Proteobacteria	g__Ferrimonas	0.0008062	0.0002204	0.0005953	0.000299	0.03121	0.06485	-0.00042	2.49E-05	-0.00021
p__Proteobacteria	g__Thiohalorhabdus	0.0006163	0.0002904	0.000409	0.000135	0.03764	0.07496	-0.00039	-2.71E-05	-0.00021
p__Proteobacteria	g__Desulfotalea	0.0002527	0.0001972	4.77E-05	3.24E-05	0.001706	0.007373	-0.00032	-9.60E-05	-0.00021
p__Proteobacteria	g__Desulfoluna	0.0004139	0.0001361	0.0002096	4.74E-05	0.001706	0.007373	-0.00029	-0.00012	-0.0002
p__Proteobacteria	g__Arsukibacterium	0.0002195	0.0002053	1.56E-05	1.55E-05	0.000233	0.00248	-0.00032	-9.14E-05	-0.0002
p__Proteobacteria	g__Reinekea	0.0003389	0.0001448	0.0001397	4.29E-05	0.000769	0.004362	-0.00029	-0.00011	-0.0002
p__Proteobacteria	g__Tateyamaria	0.000512	0.0001287	0.0003128	7.57E-05	0.002202	0.008721	-0.00029	-0.00011	-0.0002
p__Proteobacteria	g__Wolbachia	0.0002702	0.0001057	7.36E-05	2.60E-05	0.00033	0.002769	-0.00026	-0.00013	-0.0002
p__Proteobacteria	g__Neptuniibacter	0.0002865	0.0001141	9.07E-05	4.87E-05	0.000183	0.002329	-0.00028	-0.00013	-0.0002
p__Proteobacteria	g__Desulfonauticus	0.0004738	0.0001601	0.0002781	7.75E-05	0.004586	0.01474	-0.0003	-9.49E-05	-0.0002
p__Proteobacteria	g__Candidatus_Symbiobacter	0.0003256	0.0001465	0.0001303	5.00E-05	0.000769	0.004362	-0.00029	-0.00011	-0.0002
p__Proteobacteria	g__Grimontia	0.0005552	0.0001777	0.0003685	0.000133	0.01726	0.0402	-0.00032	-6.36E-05	-0.00019
p__Proteobacteria	g__Sulfuriflexus	0.0004207	0.0002136	0.0002378	9.82E-05	0.03764	0.07496	-0.00033	-5.14E-05	-0.00018
p__Proteobacteria	g__Lentilitoribacter	0.0004957	0.0001736	0.0003135	0.000114	0.01726	0.0402	-0.0003	-5.68E-05	-0.00018
p__Proteobacteria	g__Sinirhodobacter	0.001479	0.0002226	0.001298	0.000186	0.04515	0.08665	-0.00037	5.08E-06	-0.00018
p__Proteobacteria	g__Jahnella	0.0003575	0.0001368	0.0001768	5.98E-05	0.002827	0.01032	-0.00027	-8.95E-05	-0.00018
p__Proteobacteria	g__Roseisalinus	0.0003712	0.0001224	0.0001909	4.55E-05	0.001008	0.00518	-0.00026	-0.00011	-0.00018
p__Proteobacteria	g__Thioflexothrix	0.0006533	0.0001601	0.0004746	9.85E-05	0.01133	0.02909	-0.00029	-7.22E-05	-0.00018
p__Proteobacteria	g__unclassified_f__Piscirickettsiaceae	0.0006936	0.0001845	0.0005163	0.000126	0.03121	0.06485	-0.00031	-4.34E-05	-0.00018
p__Proteobacteria	g__Marinomonas	0.001521	0.0001925	0.001344	0.000132	0.01726	0.0402	-0.00031	-1.49E-05	-0.00018
p__Proteobacteria	g__Pseudoroseicyclus	0.0005457	0.0001068	0.0003693	0.000106	0.007285	0.02069	-0.00026	-9.02E-05	-0.00018
p__Proteobacteria	g__Pseudophaeobacter	0.0003263	0.0001782	0.0001505	7.89E-05	0.01726	0.0402	-0.00029	-6.88E-05	-0.00018
p__Proteobacteria	g__Phaselicystis	0.000229	0.0001421	5.35E-05	3.57E-05	0.000246	0.00248	-0.00027	-9.95E-05	-0.00018
p__Proteobacteria	g__unclassified_f__Crenotrichaceae	0.0004404	0.0001152	0.0002663	4.69E-05	0.000583	0.003709	-0.00025	-0.00011	-0.00017
p__Proteobacteria	g__Desulfocarbo	0.0003633	0.0001317	0.0001896	5.71E-05	0.005795	0.0177	-0.00026	-9.00E-05	-0.00017
p__Proteobacteria	g__Sedimentitalea	0.000399	0.0001337	0.000226	8.10E-05	0.002202	0.008721	-0.00027	-8.40E-05	-0.00017
p__Proteobacteria	g__unclassified_f__Halobacteriovorac	0.0007657	0.0001464	0.0005982	0.000432	0.03121	0.06485	-0.0004	0.000121	-0.00017
p__Proteobacteria	g__Zobellella	0.0006769	0.0001414	0.0005098	8.06E-05	0.009108	0.02457	-0.00027	-7.79E-05	-0.00017
p__Proteobacteria	g__Methylarcula	0.0003455	9.14E-05	0.0001786	8.29E-05	0.002827	0.01032	-0.00024	-9.71E-05	-0.00017
p__Proteobacteria	g__Marinagarivorans	0.0002296	0.0001749	6.33E-05	2.06E-05	0.000769	0.004362	-0.00028	-7.84E-05	-0.00017
p__Proteobacteria	g__Formivibrio	0.0003315	0.000129	0.0001705	7.04E-05	0.001008	0.00518	-0.00025	-7.25E-05	-0.00016
p__Proteobacteria	g__Amantichitinum	0.0002547	9.29E-05	9.40E-05	4.97E-05	0.001315	0.006216	-0.00022	-9.70E-05	-0.00016
p__Proteobacteria	g__Wenxinia	0.0006753	0.0001204	0.0005149	8.68E-05	0.007285	0.02069	-0.00025	-6.33E-05	-0.00016
p__Proteobacteria	g__unclassified_f__Brucellaceae	0.0002158	8.55E-05	6.40E-05	5.06E-05	0.000769	0.004362	-0.00021	-9.45E-05	-0.00015
p__Proteobacteria	g__unclassified_f__Alcanivoracaceae	0.0001988	0.0001343	4.81E-05	1.27E-05	0.004586	0.01474	-0.00023	-7.75E-05	-0.00015
p__Proteobacteria	g__Desulfoplanes	0.0002846	6.65E-05	0.0001362	7.50E-05	0.001315	0.006216	-0.00021	-9.04E-05	-0.00015
p__Proteobacteria	g__Advenella	0.0007762	6.57E-05	0.0006282	0.000169	0.03764	0.07496	-0.00025	-4.17E-05	-0.00015
p__Proteobacteria	g__Cereibacter	0.0003098	0.0001255	0.0001637	3.92E-05	0.002827	0.01032	-0.00023	-7.28E-05	-0.00015
p__Proteobacteria	g__Salinimonas	0.0001913	0.000105	4.97E-05	3.53E-05	0.001706	0.007373	-0.0002	-8.08E-05	-0.00014
p__Proteobacteria	g__Lawsonia_f__Desulfovibrionaceae	0.0001639	9.84E-05	2.65E-05	2.84E-05	0.00044	0.003169	-0.0002	-7.98E-05	-0.00014
p__Proteobacteria	g__Pseudorhizobium	0.0005224	0.0001721	0.0003859	7.18E-05	0.04515	0.08665	-0.00024	-3.22E-05	-0.00014
p__Proteobacteria	g__Pseudogemmobacter	0.0003456	8.38E-05	0.0002093	8.35E-05	0.005795	0.0177	-0.0002	-6.58E-05	-0.00014
p__Proteobacteria	g__Tropicibacter	0.0002797	5.96E-05	0.0001447	4.62E-05	0.00044	0.003169	-0.00018	-9.20E-05	-0.00014
p__Proteobacteria	g__Deefgea	0.0002055	0.0001256	7.05E-05	3.93E-05	0.005795	0.0177	-0.00022	-6.05E-05	-0.00014
p__Proteobacteria	g__Profundibacterium	0.0002175	9.52E-05	8.75E-05	5.40E-05	0.001706	0.007373	-0.00019	-6.84E-05	-0.00013
p__Proteobacteria	g__Pelagivirga	0.000277	9.62E-05	0.0001493	6.97E-05	0.009108	0.02457	-0.00019	-5.62E-05	-0.00013
p__Proteobacteria	g__Candidatus_Methylopumilus	0.0002257	0.000133	0.0001004	4.97E-05	0.004586	0.01474	-0.00022	-5.02E-05	-0.00013
p__Proteobacteria	g__Hippea	0.0001616	0.0001315	4.16E-05	2.48E-05	0.004586	0.01474	-0.00021	-4.60E-05	-0.00012

p__Proteobacteria	g__Oceaniovalibus	0.0004917	0.000124	0.0003727	9.23E-05	0.04515	0.08665	-0.00021	-2.44E-05	-0.00012
p__Proteobacteria	g__Zymomonas	0.0004369	0.0001541	0.0003198	0.000175	0.03764	0.07496	-0.00025	2.49E-05	-0.00012
p__Proteobacteria	g__Tritonibacter	0.0002269	0.0001163	0.00011	3.50E-05	0.005795	0.0177	-0.00019	-5.12E-05	-0.00012
p__Proteobacteria	g__Simulacricoccus	0.0002592	0.0001079	0.0001432	6.23E-05	0.01402	0.03427	-0.00019	-3.90E-05	-0.00012
p__Proteobacteria	g__Histidinibacterium	0.0003553	0.0001272	0.0002424	7.27E-05	0.02575	0.05574	-0.0002	-3.73E-05	-0.00011
p__Proteobacteria	g__Sodalis	0.0002709	6.26E-05	0.0001593	4.73E-05	0.001315	0.006216	-0.00016	-6.54E-05	-0.00011
p__Proteobacteria	g__Moritella	0.000163	5.26E-05	5.41E-05	2.62E-05	0.00044	0.003169	-0.00014	-7.67E-05	-0.00011
p__Proteobacteria	g__Cognatiyoonia	0.0001406	5.88E-05	3.20E-05	2.40E-05	0.000583	0.003709	-0.00015	-7.21E-05	-0.00011
p__Proteobacteria	g__Bilophila	0.0002035	8.10E-05	9.54E-05	5.56E-05	0.005795	0.0177	-0.00016	-4.92E-05	-0.00011
p__Proteobacteria	g__Tamilnaduibacter	0.0001561	0.0001141	4.81E-05	1.99E-05	0.007285	0.02069	-0.00018	-4.13E-05	-0.00011
p__Proteobacteria	g__Rouxiella	0.0001961	0.0001194	9.24E-05	4.88E-05	0.009108	0.02457	-0.00019	-3.18E-05	-0.0001
p__Proteobacteria	g__Oligella	0.0001368	0.0001041	3.41E-05	2.43E-05	0.001315	0.006216	-0.00017	-4.62E-05	-0.0001
p__Proteobacteria	g__Corticimicrobacter	0.0001384	5.33E-05	3.57E-05	2.90E-05	0.000246	0.00248	-0.00014	-6.90E-05	-0.0001
p__Proteobacteria	g__Rhodosalinus	0.0002901	8.86E-05	0.0001878	6.15E-05	0.01133	0.02909	-0.00016	-3.80E-05	-0.0001
p__Proteobacteria	g__Aestuariivita	0.0001769	2.99E-05	7.48E-05	2.50E-05	0.000183	0.002329	-0.00013	-7.88E-05	-0.0001
p__Proteobacteria	g__Parasedimentitalea	0.0002398	7.23E-05	0.0001381	7.53E-05	0.009108	0.02457	-0.00016	-3.90E-05	-0.0001
p__Proteobacteria	g__Shigella	0.0002186	5.68E-05	0.0001175	3.71E-05	0.00044	0.003169	-0.00015	-6.29E-05	-0.0001
p__Proteobacteria	g__Bombella	0.0001416	0.0001292	4.08E-05	2.36E-05	0.01402	0.03427	-0.00018	-2.95E-05	-0.0001
p__Proteobacteria	g__Thalassolituus	0.0004723	9.47E-05	0.0003729	5.90E-05	0.04515	0.08665	-0.00017	-3.34E-05	-9.94E-05
p__Proteobacteria	g__Acidomonas	0.0002623	0.0001319	0.0001629	7.88E-05	0.03764	0.07496	-0.0002	-1.75E-05	-9.94E-05
p__Proteobacteria	g__Marisediminitalea	0.0001677	9.84E-05	7.10E-05	3.52E-05	0.009108	0.02457	-0.00016	-3.66E-05	-9.68E-05
p__Proteobacteria	g__Sutterella	0.0002635	8.68E-05	0.0001696	6.04E-05	0.009108	0.02457	-0.00016	-3.49E-05	-9.39E-05
p__Proteobacteria	g__unclassified_o__Aeromonadales	0.0004206	8.11E-05	0.0003268	8.84E-05	0.02575	0.05574	-0.00016	-2.05E-05	-9.39E-05
p__Proteobacteria	g__Bergeriella_f__Neisseriaceae	9.42E-05	8.18E-05	8.82E-07	1.86E-06	0.000566	0.003709	-0.00015	-4.84E-05	-9.33E-05
p__Proteobacteria	g__Rivicola	0.0001677	8.24E-05	7.81E-05	4.18E-05	0.01133	0.02909	-0.00015	-3.25E-05	-8.97E-05
p__Proteobacteria	g__Aliishimia	0.0001507	8.65E-05	6.22E-05	3.20E-05	0.002827	0.01032	-0.00014	-3.94E-05	-8.85E-05
p__Proteobacteria	g__Acidimangrovimonas	0.0002618	6.10E-05	0.0001758	5.92E-05	0.007285	0.02069	-0.00014	-3.44E-05	-8.60E-05
p__Proteobacteria	g__Desulfatirhabdium	0.0002669	6.84E-05	0.0001817	6.56E-05	0.01133	0.02909	-0.00014	-2.65E-05	-8.52E-05
p__Proteobacteria	g__Leclercia	0.0001136	0.000107	2.88E-05	1.23E-05	0.01726	0.0402	-0.00016	-2.61E-05	-8.48E-05
p__Proteobacteria	g__unclassified_f__Pelagibacteraceae	0.0002132	5.05E-05	0.000133	4.76E-05	0.003611	0.01236	-0.00012	-4.02E-05	-8.02E-05
p__Proteobacteria	g__Roseibacterium	0.0003339	8.64E-05	0.0002544	7.38E-05	0.03764	0.07496	-0.00014	-1.65E-05	-7.95E-05
p__Proteobacteria	g__Desulfamplus	0.0002249	7.69E-05	0.0001457	5.48E-05	0.01402	0.03427	-0.00014	-2.64E-05	-7.92E-05
p__Proteobacteria	g__Nitratiruptor	9.97E-05	5.51E-05	2.75E-05	3.13E-05	0.001706	0.007373	-0.00011	-3.75E-05	-7.22E-05
p__Proteobacteria	g__Ignatzschineria	7.05E-05	5.72E-05	0	0	6.39E-05	0.002329	-0.0001	-3.94E-05	-7.05E-05
p__Proteobacteria	g__Thalassobacter	0.0001569	7.04E-05	8.71E-05	4.96E-05	0.03121	0.06485	-0.00012	-1.56E-05	-6.98E-05
p__Proteobacteria	g__Chachezhania	0.0001091	2.91E-05	4.42E-05	2.18E-05	0.00044	0.003169	-8.60E-05	-4.35E-05	-6.49E-05
p__Proteobacteria	g__Aggregatibacter	0.0001032	2.82E-05	3.84E-05	3.42E-05	0.001706	0.007373	-8.85E-05	-3.87E-05	-6.48E-05
p__Proteobacteria	g__Ectothiorhodosinus	0.000114	6.10E-05	4.97E-05	3.55E-05	0.002202	0.008721	-0.00011	-2.72E-05	-6.43E-05
p__Proteobacteria	g__Natronohydrobacter	9.94E-05	5.47E-05	3.53E-05	1.86E-05	0.002827	0.01032	-9.95E-05	-3.12E-05	-6.41E-05
p__Proteobacteria	g__Achromatium	0.0001578	5.82E-05	9.61E-05	4.48E-05	0.02113	0.04755	-0.00011	-1.96E-05	-6.17E-05
p__Proteobacteria	g__Cardiobacterium	0.0001066	5.14E-05	4.53E-05	2.61E-05	0.01402	0.03427	-9.13E-05	-2.94E-05	-6.13E-05
p__Proteobacteria	g__Laribacter	0.0001713	3.96E-05	0.0001102	5.03E-05	0.01402	0.03427	-9.95E-05	-1.91E-05	-6.12E-05
p__Proteobacteria	g__Piscirickettsia	0.0001059	5.88E-05	4.51E-05	4.89E-05	0.01402	0.03427	-0.0001	-1.82E-05	-6.08E-05
p__Proteobacteria	g__Nissabacter	7.74E-05	3.61E-05	1.75E-05	1.23E-05	0.000183	0.002329	-8.40E-05	-3.80E-05	-5.99E-05
p__Proteobacteria	g__Neiella	7.62E-05	3.88E-05	1.63E-05	1.99E-05	0.001008	0.00518	-8.77E-05	-3.36E-05	-5.99E-05
p__Proteobacteria	g__Budvicia	9.77E-05	6.06E-05	3.88E-05	1.31E-05	0.005795	0.0177	-0.0001	-2.60E-05	-5.88E-05
p__Proteobacteria	g__Falsirhodobacter	8.24E-05	3.55E-05	2.41E-05	9.61E-06	0.002202	0.008721	-7.89E-05	-3.47E-05	-5.83E-05
p__Proteobacteria	g__Modicisalibacter	0.0001237	4.08E-05	6.63E-05	3.31E-05	0.007285	0.02069	-8.63E-05	-2.49E-05	-5.75E-05

p__Proteobacteria	g__Candidatus_Puniceispirillum	0.0001307	6.14E-05	7.44E-05	2.96E-05	0.01402	0.03427	-9.80E-05	-1.55E-05	-5.62E-05
p__Proteobacteria	g__Taylorella	6.65E-05	5.99E-05	1.20E-05	1.18E-05	0.002827	0.01032	-9.47E-05	-2.30E-05	-5.44E-05
p__Proteobacteria	g__Amnimonas	0.0001389	5.71E-05	8.67E-05	3.36E-05	0.04515	0.08665	-9.08E-05	-1.24E-05	-5.22E-05
p__Proteobacteria	g__unclassified_f__Colwelliaceae	6.69E-05	3.96E-05	1.56E-05	1.34E-05	0.000769	0.004362	-7.83E-05	-2.93E-05	-5.13E-05
p__Proteobacteria	g__Snodgrassella	0.0001325	3.73E-05	8.13E-05	3.64E-05	0.009108	0.02457	-8.01E-05	-1.87E-05	-5.12E-05
p__Proteobacteria	g__Pajaroellobacter	0.0001284	5.67E-05	8.17E-05	7.88E-05	0.01726	0.0402	-9.62E-05	1.93E-05	-4.67E-05
p__Proteobacteria	g__Pasteurella	7.62E-05	3.30E-05	2.98E-05	2.00E-05	0.002827	0.01032	-7.23E-05	-2.27E-05	-4.64E-05
p__Proteobacteria	g__unclassified_f__Chromobacteriace	5.22E-05	3.38E-05	6.41E-06	6.51E-06	0.000233	0.00248	-6.80E-05	-2.81E-05	-4.58E-05
p__Proteobacteria	g__Actinobacillus	9.37E-05	3.94E-05	4.85E-05	2.78E-05	0.009108	0.02457	-7.49E-05	-1.75E-05	-4.52E-05
p__Proteobacteria	g__Bibersteinia	4.24E-05	3.98E-05	4.52E-07	1.43E-06	0.000121	0.002329	-6.53E-05	-1.93E-05	-4.19E-05
p__Proteobacteria	g__Suttonella	5.80E-05	4.02E-05	1.93E-05	1.42E-05	0.03121	0.06485	-6.34E-05	-1.43E-05	-3.87E-05
p__Proteobacteria	g__Hydrogenimonas	5.47E-05	5.15E-05	1.71E-05	1.58E-05	0.009108	0.02457	-7.29E-05	-1.03E-05	-3.76E-05
p__Proteobacteria	g__Roseicitreum	0.0001151	3.97E-05	7.83E-05	2.51E-05	0.03121	0.06485	-6.61E-05	-8.74E-06	-3.68E-05
p__Proteobacteria	g__Neoasaia	6.80E-05	3.53E-05	3.17E-05	1.15E-05	0.01402	0.03427	-5.92E-05	-1.63E-05	-3.63E-05
p__Proteobacteria	g__Duodenibacillus	4.00E-05	4.27E-05	3.91E-06	9.61E-06	0.00232	0.009123	-6.73E-05	-1.41E-05	-3.61E-05
p__Proteobacteria	g__Fangia	4.31E-05	2.71E-05	7.10E-06	6.29E-06	0.00099	0.00518	-5.29E-05	-1.94E-05	-3.60E-05
p__Proteobacteria	g__Arsenophonus	5.60E-05	3.89E-05	2.23E-05	2.33E-05	0.009108	0.02457	-6.09E-05	-5.10E-06	-3.37E-05
p__Proteobacteria	g__Saliniradius	5.79E-05	3.21E-05	2.44E-05	2.21E-05	0.02113	0.04755	-5.75E-05	-1.03E-05	-3.35E-05
p__Proteobacteria	g__Aquicoccus	5.40E-05	2.98E-05	2.19E-05	1.19E-05	0.03121	0.06485	-5.03E-05	-1.29E-05	-3.21E-05
p__Proteobacteria	g__Ventosimonas	5.72E-05	2.73E-05	2.54E-05	1.27E-05	0.001706	0.007373	-5.21E-05	-1.65E-05	-3.19E-05
p__Proteobacteria	g__Saccharibacter	4.72E-05	3.52E-05	1.56E-05	1.76E-05	0.03096	0.06485	-5.50E-05	-9.77E-06	-3.16E-05
p__Proteobacteria	g__Nitratifractor	3.85E-05	2.72E-05	7.08E-06	9.60E-06	0.002119	0.008721	-4.99E-05	-1.54E-05	-3.14E-05
p__Proteobacteria	g__Candidatus_Micropelagos	4.27E-05	4.47E-05	1.31E-05	7.98E-06	0.04515	0.08665	-6.08E-05	-7.17E-06	-2.96E-05
p__Proteobacteria	g__Pontivivens	3.79E-05	2.24E-05	1.03E-05	7.56E-06	0.004571	0.01474	-4.13E-05	-1.29E-05	-2.76E-05
p__Proteobacteria	g__Desulfonatronospira	5.99E-05	2.22E-05	3.31E-05	2.77E-05	0.01726	0.0402	-4.67E-05	-3.69E-06	-2.68E-05
p__Proteobacteria	g__Pseudooctadecabacter	3.70E-05	2.89E-05	1.16E-05	8.77E-06	0.02113	0.04755	-4.39E-05	-9.17E-06	-2.54E-05
p__Proteobacteria	g__unclassified_f__Coxiellaceae	5.17E-05	2.43E-05	2.82E-05	4.37E-05	0.02113	0.04755	-4.83E-05	8.96E-06	-2.34E-05
p__Proteobacteria	g__Alginatibacterium	2.24E-05	2.56E-05	0	0	0.000751	0.004362	-3.80E-05	-9.00E-06	-2.24E-05
p__Proteobacteria	g__unclassified_f__Holosporaceae	3.39E-05	3.32E-05	1.16E-05	1.02E-05	0.03115	0.06485	-4.43E-05	-3.10E-06	-2.24E-05
p__Proteobacteria	g__Izhakiella	2.60E-05	3.25E-05	6.85E-06	1.10E-05	0.01331	0.03401	-4.11E-05	-2.98E-06	-1.92E-05
p__Proteobacteria	g__Occidentia	1.84E-05	2.09E-05	3.91E-07	1.24E-06	0.004869	0.01561	-3.11E-05	-6.33E-06	-1.80E-05
p__Proteobacteria	g__Pseudoarcobacter	1.83E-05	1.58E-05	1.78E-06	3.10E-06	0.00232	0.009123	-2.67E-05	-8.00E-06	-1.65E-05
p__Proteobacteria	g__Morococcus	1.83E-05	1.21E-05	4.37E-06	6.58E-06	0.005171	0.01656	-2.25E-05	-6.09E-06	-1.39E-05
p__Proteobacteria	g__Candidatus_Jidaibacter	1.76E-05	1.40E-05	4.67E-06	5.17E-06	0.03621	0.07449	-2.16E-05	-4.03E-06	-1.29E-05
p__Proteobacteria	g__Halopeptonella	1.71E-05	8.76E-06	4.63E-06	4.29E-06	0.002169	0.008721	-1.82E-05	-6.89E-06	-1.25E-05
p__Proteobacteria	g__Necropsobacter	1.24E-05	1.82E-05	0	0	0.002213	0.008721	-2.44E-05	-4.17E-06	-1.24E-05
p__Proteobacteria	g__Candidatus_Adiutrix	1.18E-05	1.56E-05	0	0	0.002213	0.008721	-2.21E-05	-3.70E-06	-1.18E-05
p__Proteobacteria	g__Thorsellia	1.13E-05	1.81E-05	0	0	0.002213	0.008721	-2.36E-05	-3.26E-06	-1.13E-05
p__Proteobacteria	g__Aquimixticola	1.19E-05	1.35E-05	8.65E-07	1.82E-06	0.02958	0.06374	-2.01E-05	-3.60E-06	-1.11E-05
p__Proteobacteria	g__unclassified_f__Kangiellaceae	1.35E-05	1.21E-05	2.71E-06	7.28E-06	0.02288	0.0514	-1.86E-05	-2.18E-06	-1.08E-05
p__Proteobacteria	g__Wolinella	1.42E-05	1.10E-05	3.88E-06	3.76E-06	0.03514	0.07239	-1.75E-05	-3.67E-06	-1.03E-05
p__Proteobacteria	g__Caedibacter	1.16E-05	7.58E-06	2.05E-06	3.52E-06	0.003	0.01092	-1.50E-05	-4.57E-06	-9.56E-06
p__Proteobacteria	g__Candidatus_Neoehrlichia	9.37E-06	6.73E-06	4.52E-07	1.43E-06	0.001444	0.006807	-1.32E-05	-4.89E-06	-8.91E-06
p__Proteobacteria	g__Candidatus_Aquarickettsia	9.08E-06	1.09E-05	4.01E-07	1.27E-06	0.04033	0.08009	-1.60E-05	-1.90E-06	-8.68E-06
p__Proteobacteria	g__Ehrlichia	7.91E-06	7.70E-06	0	0	0.005972	0.01818	-1.23E-05	-3.71E-06	-7.91E-06
p__Proteobacteria	g__Trabulsiella	1.06E-05	7.62E-06	3.91E-06	8.13E-06	0.01498	0.03635	-1.31E-05	2.65E-07	-6.66E-06
p__Proteobacteria	g__Orbus	5.83E-06	5.95E-06	0	0	0.005972	0.01818	-9.54E-06	-2.24E-06	-5.83E-06
p__Proteobacteria	g__unclassified_f__Erwiniaceae	5.72E-06	6.98E-06	8.84E-07	2.80E-06	0.03712	0.07496	-9.51E-06	-5.16E-07	-4.84E-06

p__Proteobacteria	g__Chelonobacter	3.70E-06	6.05E-06	0	0	0.03498	0.07215	-7.74E-06	-4.35E-07	-3.70E-06
p__Proteobacteria	g__unclassified_f__Bradymonadaceae	2.98E-06	3.33E-06	0	0	0.01493	0.03629	-4.81E-06	-1.01E-06	-2.98E-06
p__Planctomycetes	g__unclassified_c__Phycisphaerae	0.2742	0.1214	0.1092	0.01705	0.001008	0.00518	-0.2399	-0.09942	-0.165
p__Planctomycetes	g__unclassified_o__Phycisphaerales	0.2178	0.1242	0.1131	0.05673	0.01726	0.0402	-0.1876	-0.03043	-0.1047
p__Planctomycetes	g__unclassified_f__Lacipirellulaceae	0.2902	0.04607	0.1873	0.05187	0.001315	0.006216	-0.1401	-0.05675	-0.1028
p__Planctomycetes	g__unclassified_f__Planctomycetacea	0.4877	0.05455	0.4027	0.09952	0.02575	0.05574	-0.1511	-0.01417	-0.08504
p__Planctomycetes	g__Lacipirellula	0.1994	0.03116	0.1279	0.03616	0.001315	0.006216	-0.09814	-0.04305	-0.07145
p__Planctomycetes	g__Phycisphaera	0.04528	0.07946	0.003243	0.002078	0.000769	0.004362	-0.09785	-0.00913	-0.04204
p__Planctomycetes	g__unclassified_c__Planctomycetia	0.1492	0.02575	0.1167	0.02671	0.02575	0.05574	-0.05302	-0.01125	-0.03252
p__Planctomycetes	g__Rhodopirellula	0.08513	0.01138	0.05373	0.006322	0.000183	0.002329	-0.03875	-0.02307	-0.0314
p__Planctomycetes	g__Gemmata	0.08749	0.02298	0.06852	0.0148	0.03764	0.07496	-0.03555	-0.00428	-0.01897
p__Planctomycetes	g__Blastopirellula	0.05017	0.007296	0.03492	0.004671	0.000246	0.00248	-0.0211	-0.01023	-0.01526
p__Planctomycetes	g__unclassified_f__Phycisphaeraceae	0.05575	0.01429	0.04347	0.02115	0.04515	0.08665	-0.02708	0.003544	-0.01228
p__Planctomycetes	g__unclassified_f__Pirellulaceae	0.03189	0.00591	0.02006	0.002014	0.000246	0.00248	-0.01523	-0.00813	-0.01183
p__Planctomycetes	g__Schlesneria	0.04174	0.007889	0.03094	0.007497	0.007285	0.02069	-0.01746	-0.00464	-0.0108
p__Planctomycetes	g__Planctomyces	0.04141	0.009091	0.0333	0.005325	0.03764	0.07496	-0.01439	-0.00225	-0.00811
p__Planctomycetes	g__Planctomicrobium	0.02625	0.008425	0.01879	0.007647	0.04515	0.08665	-0.01401	-0.00059	-0.00746
p__Planctomycetes	g__Bythopirellula	0.02212	0.0032	0.01514	0.003053	0.000769	0.004362	-0.0096	-0.00431	-0.00698
p__Planctomycetes	g__Aquisphaera	0.04507	0.004536	0.03967	0.002537	0.007285	0.02069	-0.00841	-0.00204	-0.0054
p__Planctomycetes	g__Candidatus_Anammoximicrobium	0.02947	0.004713	0.02407	0.004797	0.03121	0.06485	-0.00933	-0.00133	-0.0054
p__Planctomycetes	g__Rubinisphaera	0.01563	0.002134	0.01083	0.001817	0.00033	0.002769	-0.00657	-0.00311	-0.00481
p__Planctomycetes	g__Roseimaritima	0.01202	0.001565	0.008412	0.001373	0.000183	0.002329	-0.00491	-0.0024	-0.00361
p__Planctomycetes	g__Gimesia	0.009531	0.001013	0.006897	0.001012	0.00033	0.002769	-0.00348	-0.00185	-0.00263
p__Planctomycetes	g__Tautonia	0.02284	0.002266	0.02024	0.001941	0.01402	0.03427	-0.00436	-0.00081	-0.0026
p__Planctomycetes	g__Fuerstia_f__Planctomycetaceae	0.01154	0.00171	0.009199	0.001876	0.01402	0.03427	-0.00382	-0.00081	-0.00234
p__Planctomycetes	g__Thermogutta	0.005111	0.0007142	0.003069	0.000528	0.000183	0.002329	-0.00258	-0.0015	-0.00204
p__Planctomycetes	g__Bremerella	0.005935	0.0007176	0.003922	0.000837	0.000246	0.00248	-0.00271	-0.00135	-0.00201
p__Planctomycetes	g__Planctopirus	0.006766	0.001111	0.005007	0.000752	0.000769	0.004362	-0.00254	-0.00103	-0.00176
p__Planctomycetes	g__Isosphaera	0.004331	0.000543	0.002869	0.000506	0.000183	0.002329	-0.00191	-0.00099	-0.00146
p__Planctomycetes	g__Rubripirellula	0.005624	0.0008041	0.004588	0.000754	0.01402	0.03427	-0.00171	-0.00039	-0.00104
p__Planctomycetes	g__Crateriforma	0.00132	0.0003664	0.0005622	0.000179	0.000246	0.00248	-0.001	-0.00053	-0.00076
p__Planctomycetes	g__Mariniblastus	0.001616	0.0001804	0.0009973	0.000288	0.000246	0.00248	-0.00081	-0.00041	-0.00062
p__Planctomycetes	g__Alienimonas	0.003453	0.0006028	0.002866	0.000359	0.02575	0.05574	-0.00104	-0.00016	-0.00059
p__Planctomycetes	g__Limihaloglobus	0.0003424	0.000133	9.17E-05	7.09E-05	0.000583	0.003709	-0.00034	-0.00016	-0.00025
p__Planctomycetes	g__unclassified_f__Isosphaeraceae	3.29E-05	3.60E-05	9.44E-06	8.34E-06	0.04049	0.08036	-4.89E-05	-4.92E-06	-2.35E-05
p__Bacteroidetes	g__unclassified_f__Flavobacteriaceae	0.08324	0.06161	0.008938	0.00458	0.000183	0.002329	-0.1155	-0.04012	-0.0743
p__Bacteroidetes	g__Muricauda	0.01571	0.01034	0.004322	0.001488	0.00033	0.002769	-0.01757	-0.00573	-0.01139
p__Bacteroidetes	g__Maribacter	0.007204	0.00493	0.002005	0.00088	0.001315	0.006216	-0.00848	-0.00254	-0.0052
p__Bacteroidetes	g__Arenibacter	0.005673	0.003859	0.001357	0.000591	0.000583	0.003709	-0.00666	-0.00234	-0.00432
p__Bacteroidetes	g__Pricia	0.003383	0.002018	0.0007473	0.000237	0.000183	0.002329	-0.00386	-0.00151	-0.00264
p__Bacteroidetes	g__Zeaxanthinibacter	0.002198	0.00167	0.0001557	0.000106	0.000183	0.002329	-0.0031	-0.00111	-0.00204
p__Bacteroidetes	g__Eudoraea	0.002173	0.001369	0.0002635	0.000135	0.000183	0.002329	-0.00282	-0.00117	-0.00191
p__Bacteroidetes	g__Flagellimonas	0.002776	0.001648	0.001016	0.000208	0.001706	0.007373	-0.00284	-0.0009	-0.00176
p__Bacteroidetes	g__Ulvibacterium	0.002392	0.001498	0.0006546	0.000144	0.000183	0.002329	-0.00273	-0.00091	-0.00174
p__Bacteroidetes	g__Robiginitalea	0.001704	0.001333	0.000178	0.000102	0.000246	0.00248	-0.00237	-0.00078	-0.00153
p__Bacteroidetes	g__Ekhidna	0.002269	0.000419	0.001179	0.000265	0.000183	0.002329	-0.00137	-0.00078	-0.00109
p__Bacteroidetes	g__Muriicola	0.001437	0.0008162	0.0003776	0.000108	0.000183	0.002329	-0.00158	-0.00063	-0.00106
p__Bacteroidetes	g__Kriegella	0.001524	0.0009031	0.0004857	0.000185	0.00044	0.003169	-0.00161	-0.00056	-0.00104

p__Bacteroidetes	g__Zobellia	0.001098	0.0008892	0.0002056	0.000125	0.00044	0.003169	-0.00147	-0.00044	-0.00089
p__Bacteroidetes	g__Paludibacter	0.002437	0.000779	0.00165	0.000547	0.01726	0.0402	-0.00137	-0.00022	-0.00079
p__Bacteroidetes	g__Flammeovirga	0.001868	0.0006137	0.001083	0.000269	0.001315	0.006216	-0.00118	-0.0004	-0.00078
p__Bacteroidetes	g__Winogradskyella	0.001958	0.000496	0.001235	0.000228	0.000583	0.003709	-0.00107	-0.00041	-0.00072
p__Bacteroidetes	g__Catalinimonas	0.004984	0.0004753	0.00434	0.000723	0.04515	0.08665	-0.00122	-0.00013	-0.00064
p__Bacteroidetes	g__Proteiniphilum	0.0009726	0.000503	0.0003929	0.000315	0.005795	0.0177	-0.00092	-0.00023	-0.00058
p__Bacteroidetes	g__Parabacteroides	0.001303	0.0003308	0.0007252	0.000185	0.001008	0.00518	-0.00082	-0.00033	-0.00058
p__Bacteroidetes	g__Sinomicrobium	0.001373	0.0007266	0.0008101	0.000576	0.02575	0.05574	-0.00116	-5.39E-05	-0.00056
p__Bacteroidetes	g__Aequorivita	0.002097	0.0005913	0.001535	0.000474	0.04515	0.08665	-0.00101	-0.00013	-0.00056
p__Bacteroidetes	g__Saonia	0.0007133	0.0005106	0.0001649	0.00012	0.002202	0.008721	-0.0009	-0.00027	-0.00055
p__Bacteroidetes	g__Lewinella	0.001437	0.0004205	0.0009041	0.000206	0.001008	0.00518	-0.00082	-0.00027	-0.00053
p__Bacteroidetes	g__Pseudozobellia	0.0005962	0.0004262	9.51E-05	5.44E-05	0.000246	0.00248	-0.00077	-0.00027	-0.0005
p__Bacteroidetes	g__Breznakibacter	0.0005641	0.0002442	6.89E-05	4.02E-05	0.000183	0.002329	-0.00064	-0.00036	-0.0005
p__Bacteroidetes	g__Leeuwenhoekiella	0.0009134	0.0004684	0.0004204	0.000134	0.009108	0.02457	-0.00079	-0.00022	-0.00049
p__Bacteroidetes	g__Sediminicola	0.0005031	0.0003777	9.33E-05	5.47E-05	0.001706	0.007373	-0.00065	-0.00021	-0.00041
p__Bacteroidetes	g__Salinimicrobium	0.0006551	0.000395	0.0002489	9.82E-05	0.003611	0.01236	-0.00066	-0.00018	-0.00041
p__Bacteroidetes	g__Seonamhaeicola	0.0006376	0.000457	0.0002375	8.11E-05	0.001706	0.007373	-0.00069	-0.00017	-0.0004
p__Bacteroidetes	g__unclassified_f__Microscillaceae	0.001175	0.0003573	0.0007838	0.000164	0.02113	0.04755	-0.00062	-0.00017	-0.00039
p__Bacteroidetes	g__unclassified_f__Rikenellaceae	0.000433	0.0004245	8.42E-05	4.16E-05	0.01133	0.02909	-0.0006	-0.00012	-0.00035
p__Bacteroidetes	g__Algibacter	0.001061	0.0003594	0.0007214	0.000128	0.009108	0.02457	-0.00059	-0.00014	-0.00034
p__Bacteroidetes	g__Robertkochia	0.0004775	0.0003372	0.000145	0.00011	0.005795	0.0177	-0.00056	-0.00013	-0.00033
p__Bacteroidetes	g__Gillisia	0.000738	0.0002931	0.0004196	0.00019	0.04515	0.08665	-0.00054	-0.00011	-0.00032
p__Bacteroidetes	g__Gelidibacter	0.0006877	0.000412	0.0003834	0.000106	0.007285	0.02069	-0.00059	-9.85E-05	-0.0003
p__Bacteroidetes	g__Croceitalea	0.0004212	0.0002933	0.0001386	3.70E-05	0.004586	0.01474	-0.00049	-0.00012	-0.00028
p__Bacteroidetes	g__Longimonas	0.0003814	0.0001974	0.0001034	5.02E-05	0.000183	0.002329	-0.00042	-0.00018	-0.00028
p__Bacteroidetes	g__Siccationidurans	0.0005822	0.0001295	0.000308	9.95E-05	0.000583	0.003709	-0.00037	-0.00017	-0.00027
p__Bacteroidetes	g__Euzebyella	0.0002843	0.0002396	2.65E-05	3.58E-05	0.000572	0.003709	-0.00041	-0.00013	-0.00026
p__Bacteroidetes	g__Phaeodactylibacter	0.0007629	0.0001381	0.0005236	0.000149	0.009108	0.02457	-0.00035	-0.00013	-0.00024
p__Bacteroidetes	g__Salisaeta	0.0004601	0.0001549	0.0002494	0.000104	0.002827	0.01032	-0.00032	-0.00011	-0.00021
p__Bacteroidetes	g__Anditalea	0.0004013	0.0002201	0.0001922	0.000141	0.01402	0.03427	-0.00037	-5.89E-05	-0.00021
p__Bacteroidetes	g__Jiulongibacter	0.000296	0.0001204	0.0001038	6.97E-05	0.000583	0.003709	-0.00028	-0.00011	-0.00019
p__Bacteroidetes	g__Arenitalea	0.000357	0.000141	0.0001809	0.000143	0.01726	0.0402	-0.00029	-4.40E-05	-0.00018
p__Bacteroidetes	g__Tangfeifania	0.0002424	0.0001835	6.80E-05	4.19E-05	0.005795	0.0177	-0.0003	-6.85E-05	-0.00017
p__Bacteroidetes	g__Acetobacteroides	0.0002661	0.0002215	0.0001024	8.30E-05	0.03764	0.07496	-0.00032	-2.25E-05	-0.00016
p__Bacteroidetes	g__Marixanthomonas	0.0002624	9.92E-05	0.0001159	8.48E-05	0.004586	0.01474	-0.00022	-6.71E-05	-0.00015
p__Bacteroidetes	g__Salinivirga	0.0003718	0.0001176	0.0002355	8.10E-05	0.007285	0.02069	-0.00022	-4.33E-05	-0.00014
p__Bacteroidetes	g__Moheibacter	0.0001752	0.0001229	6.89E-05	5.37E-05	0.02575	0.05574	-0.00018	-3.04E-05	-0.00011
p__Bacteroidetes	g__Altibacter	0.0001515	5.86E-05	4.68E-05	2.35E-05	0.001008	0.00518	-0.00014	-6.73E-05	-0.0001
p__Bacteroidetes	g__Joostella	0.0001752	0.0001417	7.14E-05	9.93E-05	0.04515	0.08665	-0.0002	2.06E-06	-0.0001
p__Bacteroidetes	g__unclassified_f__Marinifilaceae	0.0002382	7.87E-05	0.0001349	4.80E-05	0.004586	0.01474	-0.00016	-4.75E-05	-0.0001
p__Bacteroidetes	g__Galbibacter	0.0001066	7.11E-05	9.62E-06	1.09E-05	0.000418	0.003169	-0.00014	-5.89E-05	-9.70E-05
p__Bacteroidetes	g__Algoriella	9.52E-05	6.34E-05	1.86E-05	1.25E-05	0.006482	0.01964	-0.00011	-3.93E-05	-7.67E-05
p__Bacteroidetes	g__Bernardetia	0.0001034	0.0001007	3.30E-05	3.22E-05	0.03756	0.07496	-0.00014	-1.21E-05	-7.05E-05
p__Bacteroidetes	g__Meridianimaribacter	8.01E-05	8.28E-05	1.71E-05	1.39E-05	0.003611	0.01236	-0.00012	-1.92E-05	-6.31E-05
p__Bacteroidetes	g__Aquirufa	0.0001195	6.54E-05	5.86E-05	3.13E-05	0.01133	0.02909	-0.00011	-1.95E-05	-6.10E-05
p__Bacteroidetes	g__unclassified_f__Muribaculaceae	5.46E-05	3.66E-05	5.28E-06	1.19E-05	0.000681	0.004322	-7.22E-05	-2.82E-05	-4.93E-05
p__Bacteroidetes	g__Ichthyenterobacterium	4.65E-05	7.74E-05	0	0	0.002213	0.008721	-9.96E-05	-1.34E-05	-4.65E-05
p__Bacteroidetes	g__Mesoflavibacter	6.75E-05	4.12E-05	2.31E-05	2.77E-05	0.002827	0.01032	-7.65E-05	-1.90E-05	-4.44E-05

p__Bacteroidetes	g__Mucinivorans	4.21E-05	4.27E-05	1.28E-06	2.93E-06	0.000208	0.00248	-6.72E-05	-1.85E-05	-4.08E-05
p__Bacteroidetes	g__Microbacter	3.75E-05	2.73E-05	0	0	6.39E-05	0.002329	-5.46E-05	-2.22E-05	-3.75E-05
p__Bacteroidetes	g__Pustulibacterium	3.71E-05	3.04E-05	0	0	6.39E-05	0.002329	-5.47E-05	-2.07E-05	-3.71E-05
p__Bacteroidetes	g__Marinirhabdus	3.41E-05	1.36E-05	3.51E-06	4.53E-06	0.000149	0.002329	-3.93E-05	-2.27E-05	-3.06E-05
p__Bacteroidetes	g__Cruoricaptor	2.09E-05	1.78E-05	8.84E-07	2.80E-06	0.001916	0.008236	-3.05E-05	-9.74E-06	-2.01E-05
p__Bacteroidetes	g__unclassified_f__Prevotellaceae	1.93E-05	2.92E-05	3.40E-06	5.21E-06	0.02772	0.05985	-3.73E-05	-2.36E-06	-1.59E-05
p__Bacteroidetes	g__Sanguibacteroides	1.80E-05	5.96E-06	2.92E-06	4.28E-06	0.000368	0.003079	-1.92E-05	-1.09E-05	-1.50E-05
p__Bacteroidetes	g__unclassified_f__Odoribacteraceae	2.09E-06	2.73E-06	0	0	0.03498	0.07215	-3.81E-06	-5.29E-07	-2.09E-06
p__Acidobacteria	g__unclassified_c__Thermoanaerobac	0.1082	0.04274	0.01426	0.002717	0.000183	0.002329	-0.1202	-0.06987	-0.09396
p__Acidobacteria	g__unclassified_o__Holophagales	0.009742	0.003129	0.003306	0.000285	0.000183	0.002329	-0.0084	-0.00469	-0.00644
p__Acidobacteria	g__unclassified_c__Holophagae	0.01055	0.003054	0.004273	0.000432	0.000183	0.002329	-0.00823	-0.00466	-0.00628
p__Acidobacteria	g__Geothrix	0.002856	0.0006409	0.001254	0.00028	0.000183	0.002329	-0.002	-0.00121	-0.0016
p__Acidobacteria	g__Holophaga	0.001563	0.0003598	0.0005008	0.000107	0.000183	0.002329	-0.00128	-0.00085	-0.00106
p__Acidobacteria	g__Thermoanaerobaculum	0.001513	0.0004811	0.0004967	0.000236	0.000183	0.002329	-0.00135	-0.00071	-0.00102
p__Acidobacteria	g__unclassified_f__Thermoanaerobac	4.79E-05	2.07E-05	2.56E-05	2.49E-05	0.03115	0.06485	-4.00E-05	-2.12E-06	-2.23E-05
p__Actinobacteria	g__Pseudonocardia	1.164	0.4846	0.6	0.2812	0.005795	0.0177	-0.9057	-0.2421	-0.5643
p__Actinobacteria	g__unclassified_c__Actinobacteria	0.2663	0.04055	0.1797	0.03366	0.000769	0.004362	-0.1174	-0.05713	-0.08658
p__Actinobacteria	g__Streptomyces	0.1855	0.06204	0.124	0.02082	0.003611	0.01236	-0.1015	-0.02536	-0.06149
p__Actinobacteria	g__Ilumatobacter	0.1428	0.04044	0.08796	0.02997	0.007285	0.02069	-0.08601	-0.02304	-0.05484
p__Actinobacteria	g__Amycolatopsis	0.09831	0.05166	0.0516	0.01667	0.01133	0.02909	-0.08149	-0.01657	-0.0467
p__Actinobacteria	g__unclassified_f__Acidimicrobiaceae	0.09608	0.02163	0.06458	0.02065	0.007285	0.02069	-0.04867	-0.01431	-0.03149
p__Actinobacteria	g__Micromonospora	0.06318	0.01945	0.04629	0.009229	0.02575	0.05574	-0.02897	-0.00456	-0.01689
p__Actinobacteria	g__Kribbella	0.03778	0.01168	0.02298	0.003926	0.003611	0.01236	-0.02208	-0.00757	-0.01481
p__Actinobacteria	g__Nocardia	0.03871	0.01139	0.02419	0.004155	0.002202	0.008721	-0.02244	-0.00776	-0.01452
p__Actinobacteria	g__Frankia	0.03516	0.01151	0.02174	0.005756	0.002827	0.01032	-0.021	-0.00633	-0.01342
p__Actinobacteria	g__Mycobacterium	0.07486	0.01138	0.06166	0.004488	0.007285	0.02069	-0.02114	-0.00635	-0.0132
p__Actinobacteria	g__unclassified_o__Acidimicrobiales	0.04119	0.007985	0.03063	0.00673	0.002827	0.01032	-0.0165	-0.00469	-0.01057
p__Actinobacteria	g__Rhodococcus_f__Nocardiaceae	0.03322	0.008049	0.02432	0.00728	0.03121	0.06485	-0.01518	-0.00283	-0.00891
p__Actinobacteria	g__Actinomadura	0.04206	0.009943	0.0332	0.00588	0.01726	0.0402	-0.01591	-0.00227	-0.00887
p__Actinobacteria	g__Prauserella	0.01669	0.00879	0.008367	0.002596	0.01402	0.03427	-0.01376	-0.00334	-0.00832
p__Actinobacteria	g__Mycolicibacterium	0.03613	0.008677	0.02834	0.003499	0.009108	0.02457	-0.01392	-0.00305	-0.00779
p__Actinobacteria	g__Nocardioides	0.03452	0.005765	0.02712	0.004795	0.005795	0.0177	-0.01169	-0.00296	-0.0074
p__Actinobacteria	g__Nonomuraea	0.02376	0.005197	0.01691	0.002835	0.002827	0.01032	-0.01041	-0.00332	-0.00685
p__Actinobacteria	g__unclassified_p__Actinobacteria	0.0193	0.008054	0.01297	0.001489	0.003611	0.01236	-0.01174	-0.00232	-0.00633
p__Actinobacteria	g__Geodermatophilus	0.02096	0.006042	0.01478	0.003302	0.01726	0.0402	-0.01	-0.00231	-0.00618
p__Actinobacteria	g__Kibdelosporangium	0.01167	0.007334	0.00601	0.001654	0.02113	0.04755	-0.01045	-0.0017	-0.00566
p__Actinobacteria	g__Saccharopolyspora	0.01324	0.007172	0.007808	0.003114	0.01726	0.0402	-0.00992	-0.00116	-0.00543
p__Actinobacteria	g__Kutzneria	0.011	0.0062	0.005681	0.002135	0.03764	0.07496	-0.00967	-0.00162	-0.00532
p__Actinobacteria	g__Actinoplanes	0.01575	0.00446	0.01045	0.001745	0.001706	0.007373	-0.00831	-0.00241	-0.0053
p__Actinobacteria	g__Cellulomonas	0.01296	0.00289	0.008042	0.001812	0.002827	0.01032	-0.0069	-0.00279	-0.00492
p__Actinobacteria	g__Actinomycetospora	0.009574	0.003281	0.005182	0.001926	0.005795	0.0177	-0.00648	-0.00198	-0.00439
p__Actinobacteria	g__Saccharomonospora	0.007813	0.003949	0.00379	0.001357	0.007285	0.02069	-0.00643	-0.00171	-0.00402
p__Actinobacteria	g__Thermocrispum	0.007053	0.00553	0.003255	0.001697	0.02575	0.05574	-0.00763	-0.00068	-0.0038
p__Actinobacteria	g__Modestobacter	0.008463	0.002543	0.004678	0.001165	0.002827	0.01032	-0.00545	-0.00212	-0.00379
p__Actinobacteria	g__Streptosporangium	0.00899	0.003801	0.005432	0.001357	0.004586	0.01474	-0.0061	-0.0013	-0.00356
p__Actinobacteria	g__unclassified_o__Streptosporangial	0.009756	0.002666	0.00641	0.001778	0.004586	0.01474	-0.0054	-0.0015	-0.00335
p__Actinobacteria	g__Kineosporia	0.005928	0.001833	0.002845	0.000984	0.001315	0.006216	-0.00429	-0.00189	-0.00308
p__Actinobacteria	g__Arthrobacter	0.01023	0.002714	0.007291	0.00068	0.005795	0.0177	-0.00473	-0.00137	-0.00294

p__Actinobacteria	g__Nocardiopsis	0.008097	0.002881	0.00516	0.000907	0.01133	0.02909	-0.00472	-0.00123	-0.00294
p__Actinobacteria	g__unclassified_f__Micromonosporac	0.007229	0.002685	0.004311	0.001118	0.002202	0.008721	-0.00499	-0.00138	-0.00292
p__Actinobacteria	g__unclassified_f__Ilumatobacteracea	0.006271	0.002849	0.003402	0.001019	0.005795	0.0177	-0.0048	-0.00128	-0.00287
p__Actinobacteria	g__Allokutzneria	0.005466	0.002965	0.002623	0.000978	0.007285	0.02069	-0.00463	-0.00123	-0.00284
p__Actinobacteria	g__Kitasatospora	0.01023	0.002382	0.007483	0.00125	0.004586	0.01474	-0.00459	-0.00116	-0.00275
p__Actinobacteria	g__Actinoalloteichus	0.005501	0.003495	0.002793	0.000868	0.01402	0.03427	-0.00513	-0.00073	-0.00271
p__Actinobacteria	g__Microbispora	0.008075	0.002807	0.005497	0.001134	0.03121	0.06485	-0.00445	-0.00074	-0.00258
p__Actinobacteria	g__Solirubrobacter	0.008491	0.002276	0.006027	0.001758	0.02575	0.05574	-0.00413	-0.00072	-0.00246
p__Actinobacteria	g__Salinispora	0.00437	0.003093	0.002011	0.000578	0.004586	0.01474	-0.0045	-0.00091	-0.00236
p__Actinobacteria	g__unclassified_f__Iamiaceae	0.007865	0.001356	0.005645	0.001428	0.007285	0.02069	-0.00331	-0.00105	-0.00222
p__Actinobacteria	g__Actinocrispum	0.00472	0.002886	0.002585	0.00061	0.009108	0.02457	-0.00407	-0.00068	-0.00213
p__Actinobacteria	g__unclassified_o__Actinomycetales	0.004185	0.001245	0.002057	0.000453	0.00044	0.003169	-0.00296	-0.00137	-0.00213
p__Actinobacteria	g__Planosporangium	0.004874	0.00182	0.003086	0.000943	0.007285	0.02069	-0.00306	-0.0006	-0.00179
p__Actinobacteria	g__Goodfellowiella	0.00387	0.002844	0.002231	0.001187	0.04515	0.08665	-0.00367	-0.00018	-0.00164
p__Actinobacteria	g__Cryptosporangium	0.004656	0.00117	0.003047	0.000671	0.003611	0.01236	-0.00238	-0.0008	-0.00161
p__Actinobacteria	g__Cryobacterium	0.003755	0.001219	0.002147	0.000347	0.00033	0.002769	-0.00246	-0.00091	-0.00161
p__Actinobacteria	g__Marmoricola	0.003403	0.00083	0.001873	0.000325	0.000183	0.002329	-0.00209	-0.00101	-0.00153
p__Actinobacteria	g__Phycicoccus	0.002865	0.0005918	0.001425	0.000224	0.000183	0.002329	-0.00178	-0.00104	-0.00144
p__Actinobacteria	g__Mycobacteroides	0.003882	0.000976	0.002527	0.000288	0.000183	0.002329	-0.00199	-0.00082	-0.00136
p__Actinobacteria	g__Ornithinimicrobium	0.003117	0.0004738	0.001767	0.000343	0.000246	0.00248	-0.0017	-0.00099	-0.00135
p__Actinobacteria	g__Actinomyces	0.004447	0.0004882	0.003128	0.000669	0.00044	0.003169	-0.00182	-0.00082	-0.00132
p__Actinobacteria	g__Humibacillus	0.002262	0.0004976	0.0009747	0.000231	0.000183	0.002329	-0.00163	-0.00097	-0.00129
p__Actinobacteria	g__Verrucosispora	0.003625	0.001177	0.002347	0.000792	0.01133	0.02909	-0.00213	-0.00048	-0.00128
p__Actinobacteria	g__Euzebya	0.002386	0.0006263	0.001205	0.000318	0.00044	0.003169	-0.00158	-0.00075	-0.00118
p__Actinobacteria	g__Nitriliruptor	0.003865	0.0007041	0.002716	0.000706	0.007285	0.02069	-0.00169	-0.00057	-0.00115
p__Actinobacteria	g__Haloactinobacterium	0.001928	0.0006368	0.000819	0.000221	0.000246	0.00248	-0.00152	-0.0007	-0.00111
p__Actinobacteria	g__Corynebacterium	0.003073	0.0008978	0.001975	0.000331	0.004586	0.01474	-0.00167	-0.00055	-0.0011
p__Actinobacteria	g__Janibacter	0.001667	0.0005654	0.0006136	0.000196	0.000183	0.002329	-0.00144	-0.0007	-0.00105
p__Actinobacteria	g__Aeromicrobium	0.003582	0.00094	0.002535	0.000431	0.009108	0.02457	-0.00165	-0.00042	-0.00105
p__Actinobacteria	g__Dactylosporangium	0.00238	0.001263	0.001349	0.000305	0.01402	0.03427	-0.00186	-0.00034	-0.00103
p__Actinobacteria	g__unclassified_o__Propionibacteriale	0.001976	0.0008007	0.0009536	0.000283	0.001706	0.007373	-0.00151	-0.00055	-0.00102
p__Actinobacteria	g__unclassified_f__Mycobacteriaceae	0.002283	0.0008139	0.001319	0.000343	0.001315	0.006216	-0.00155	-0.0005	-0.00096
p__Actinobacteria	g__Hamadaea	0.001897	0.0003999	0.0009528	0.00029	0.000583	0.003709	-0.00122	-0.00065	-0.00094
p__Actinobacteria	g__Actinopolyspora	0.001897	0.001008	0.0009547	0.000392	0.005795	0.0177	-0.00162	-0.00038	-0.00094
p__Actinobacteria	g__Patulibacter	0.003686	0.0007467	0.00276	0.000519	0.007285	0.02069	-0.00152	-0.0004	-0.00093
p__Actinobacteria	g__Motilibacter	0.003404	0.0008047	0.002482	0.000396	0.002827	0.01032	-0.0015	-0.0004	-0.00092
p__Actinobacteria	g__Isoptericola	0.001893	0.0002564	0.0009876	0.000276	0.000183	0.002329	-0.00113	-0.00068	-0.00091
p__Actinobacteria	g__Tetrasphaera	0.001675	0.0005055	0.0008328	0.000166	0.001315	0.006216	-0.00115	-0.00048	-0.00084
p__Actinobacteria	g__Leifsonia	0.002888	0.0009283	0.002065	0.000449	0.005795	0.0177	-0.00146	-0.00024	-0.00082
p__Actinobacteria	g__Couchioplanes	0.001494	0.0006152	0.0006783	0.000261	0.001706	0.007373	-0.0012	-0.00046	-0.00082
p__Actinobacteria	g__unclassified_f__Microbacteriaceae	0.001421	0.0003504	0.0006828	0.000132	0.000183	0.002329	-0.00095	-0.00052	-0.00074
p__Actinobacteria	g__unclassified_f__Nocardiaceae	0.001336	0.0004356	0.0006311	0.00018	0.001008	0.00518	-0.00099	-0.00042	-0.0007
p__Actinobacteria	g__Sphaerisporangium	0.001863	0.000685	0.001164	0.000386	0.005795	0.0177	-0.00116	-0.00022	-0.0007
p__Actinobacteria	g__Glycomyces	0.00229	0.0005662	0.001638	0.000168	0.002827	0.01032	-0.00103	-0.00033	-0.00065
p__Actinobacteria	g__Haloechinothrix	0.001198	0.0005173	0.0005862	0.000253	0.003611	0.01236	-0.00093	-0.00026	-0.00061
p__Actinobacteria	g__Thermomonospora	0.001479	0.0006127	0.0008708	0.000286	0.005795	0.0177	-0.00104	-0.00027	-0.00061
p__Actinobacteria	g__Luteipulveratus	0.001071	0.0003269	0.0004637	0.000114	0.00033	0.002769	-0.0008	-0.00039	-0.00061
p__Actinobacteria	g__Kocuria	0.001692	0.000482	0.001085	0.000317	0.01133	0.02909	-0.00093	-0.00028	-0.00061

p__Actinobacteria	g__Actinoallomurus	0.001527	0.0007274	0.0009236	0.000343	0.02575	0.05574	-0.00108	-0.00017	-0.0006
p__Actinobacteria	g__Krasilnikovia	0.001312	0.0005014	0.0007123	0.000258	0.003611	0.01236	-0.00094	-0.0003	-0.0006
p__Actinobacteria	g__Knoellia	0.001643	0.0004544	0.001049	0.000195	0.002202	0.008721	-0.0009	-0.00029	-0.00059
p__Actinobacteria	g__Stackebrandtia	0.0009923	0.0002858	0.0004165	0.000147	0.00033	0.002769	-0.00077	-0.00039	-0.00058
p__Actinobacteria	g__Allosalinactinospora	0.001123	0.0005686	0.0005512	0.000138	0.000769	0.004362	-0.00097	-0.00029	-0.00057
p__Actinobacteria	g__Longispora_f__Micromonosporace	0.001195	0.0004626	0.0006401	0.000144	0.001706	0.007373	-0.00086	-0.00027	-0.00056
p__Actinobacteria	g__Diaminobutyricimonas	0.0008555	0.0008367	0.0003015	5.77E-05	0.005795	0.0177	-0.00114	-0.00011	-0.00055
p__Actinobacteria	g__unclassified_f__Geodermatophilac	0.001322	0.0004291	0.0008108	0.000187	0.01133	0.02909	-0.00078	-0.00022	-0.00051
p__Actinobacteria	g__Cellulosimicrobium	0.001418	0.0004216	0.0009167	0.000202	0.002202	0.008721	-0.0008	-0.00024	-0.0005
p__Actinobacteria	g__Bifidobacterium	0.0009679	0.000287	0.0004693	9.61E-05	0.000183	0.002329	-0.0007	-0.00034	-0.0005
p__Actinobacteria	g__Terrabacter	0.001426	0.0002951	0.0009275	0.00011	0.00044	0.003169	-0.00068	-0.00032	-0.0005
p__Actinobacteria	g__unclassified_o__Micrococcales	0.001404	0.0004008	0.000907	0.000327	0.01402	0.03427	-0.00079	-0.00019	-0.0005
p__Actinobacteria	g__Embleya	0.002241	0.0005045	0.001745	0.000347	0.03121	0.06485	-0.00089	-0.00014	-0.0005
p__Actinobacteria	g__Quadrisphaera	0.0008898	0.0003468	0.0004068	0.000181	0.002202	0.008721	-0.00071	-0.00026	-0.00048
p__Actinobacteria	g__Klenkia	0.001283	0.0004476	0.0008059	0.000304	0.01133	0.02909	-0.0008	-0.00014	-0.00048
p__Actinobacteria	g__Pseudarthrobacter	0.001235	0.0002183	0.0007752	0.000114	0.00033	0.002769	-0.00061	-0.00031	-0.00046
p__Actinobacteria	g__Skermania	0.0006817	0.000216	0.0002286	5.96E-05	0.000183	0.002329	-0.00059	-0.00032	-0.00045
p__Actinobacteria	g__Salinibacterium	0.001736	0.0005988	0.001297	0.000181	0.02575	0.05574	-0.00082	-0.00012	-0.00044
p__Actinobacteria	g__Allonocardiopsis	0.0009518	0.0004227	0.0005402	0.000135	0.005795	0.0177	-0.0007	-0.00015	-0.00041
p__Actinobacteria	g__Thermopolyspora	0.001205	0.0004326	0.0007968	0.000324	0.02575	0.05574	-0.00072	-7.91E-05	-0.00041
p__Actinobacteria	g__Streptomonospora	0.0009167	0.0003156	0.0005089	0.000247	0.005795	0.0177	-0.00064	-0.00017	-0.00041
p__Actinobacteria	g__Mycolicibacter	0.001325	0.0002314	0.0009323	0.000211	0.003611	0.01236	-0.00058	-0.00021	-0.00039
p__Actinobacteria	g__Thermostaphylospora	0.0009292	0.0004447	0.0005595	0.000351	0.007285	0.02069	-0.00073	-3.42E-05	-0.00037
p__Actinobacteria	g__Auraticoccus	0.0007501	0.0002665	0.0003828	0.000146	0.004586	0.01474	-0.00054	-0.00019	-0.00037
p__Actinobacteria	g__Planobispora	0.001429	0.0003259	0.001062	0.00025	0.01133	0.02909	-0.00061	-0.00012	-0.00037
p__Actinobacteria	g__Actinospica	0.0006292	0.0003171	0.000265	9.38E-05	0.009108	0.02457	-0.00055	-0.00017	-0.00036
p__Actinobacteria	g__Acidiferrimicrobium	0.001012	0.0003051	0.0006612	0.000188	0.01726	0.0402	-0.00056	-0.00016	-0.00035
p__Actinobacteria	g__Leekyejoonella	0.0007222	0.000159	0.0003847	0.000137	0.00044	0.003169	-0.00045	-0.00022	-0.00034
p__Actinobacteria	g__Sinosporangium	0.0007656	0.0003498	0.0004428	0.000167	0.01402	0.03427	-0.00056	-0.00011	-0.00032
p__Actinobacteria	g__Oryzihumus	0.00118	0.0001709	0.0008622	0.000296	0.02113	0.04755	-0.00052	-0.00012	-0.00032
p__Actinobacteria	g__Collinsella	0.0009171	0.0001736	0.0006005	0.000131	0.002202	0.008721	-0.00045	-0.00019	-0.00032
p__Actinobacteria	g__Acidothermus	0.0004938	0.0002318	0.0001799	5.95E-05	0.000246	0.00248	-0.00046	-0.00019	-0.00031
p__Actinobacteria	g__Murinocardiopsis	0.0007054	0.0002414	0.0003982	0.000138	0.003611	0.01236	-0.00046	-0.00014	-0.00031
p__Actinobacteria	g__Actinocorallia	0.001167	0.0002414	0.0008642	0.000152	0.003611	0.01236	-0.00048	-0.00013	-0.0003
p__Actinobacteria	g__Serinicoccus	0.0005537	0.000285	0.0002727	7.28E-05	0.01726	0.0402	-0.00047	-0.00012	-0.00028
p__Actinobacteria	g__Friedmanniella	0.0008199	0.0001355	0.000546	0.000122	0.000769	0.004362	-0.00039	-0.00017	-0.00027
p__Actinobacteria	g__Sciscionella	0.000918	0.0003034	0.0006457	0.000187	0.02575	0.05574	-0.00049	-7.29E-05	-0.00027
p__Actinobacteria	g__unclassified_f__Nocardiopsaceae	0.0004946	0.0001944	0.0002272	0.000137	0.004586	0.01474	-0.0004	-0.00014	-0.00027
p__Actinobacteria	g__Nesterenkonia	0.0004822	0.0001806	0.0002171	5.61E-05	0.000183	0.002329	-0.00039	-0.00016	-0.00027
p__Actinobacteria	g__Oerskovia	0.000763	0.000257	0.0005035	0.000158	0.02113	0.04755	-0.00044	-8.43E-05	-0.00026
p__Actinobacteria	g__Schaalia	0.0004437	0.0001935	0.0001985	5.87E-05	0.002202	0.008721	-0.00036	-0.00013	-0.00025
p__Actinobacteria	g__Candidatus_Lumbricidophila	0.0002848	0.0003721	4.21E-05	3.29E-05	0.001315	0.006216	-0.00049	-5.98E-05	-0.00024
p__Actinobacteria	g__Diaminobutyricibacter	0.0002902	8.84E-05	5.32E-05	2.57E-05	0.000183	0.002329	-0.00029	-0.00018	-0.00024
p__Actinobacteria	g__Homoserinimonas	0.0003008	0.000261	6.44E-05	2.57E-05	0.000183	0.002329	-0.00042	-9.92E-05	-0.00024
p__Actinobacteria	g__Desertiactinospora	0.0004985	0.0002332	0.0002643	0.000118	0.007285	0.02069	-0.00038	-9.20E-05	-0.00023
p__Actinobacteria	g__Smaragdicoccus	0.0005068	0.0002021	0.0002762	0.000122	0.009108	0.02457	-0.00037	-8.78E-05	-0.00023
p__Actinobacteria	g__Ornithinicoccus	0.0005123	0.0001305	0.0002913	9.99E-05	0.001706	0.007373	-0.00032	-0.00013	-0.00022
p__Actinobacteria	g__Brachybacterium	0.0004548	0.0001602	0.0002347	6.36E-05	0.001008	0.00518	-0.00033	-0.00013	-0.00022

p__Actinobacteria	g__Actinotalea	0.000503	0.0001202	0.0002841	7.54E-05	0.000769	0.004362	-0.0003	-0.00013	-0.00022
p__Actinobacteria	g__Leucobacter	0.0006889	0.0002534	0.0004878	0.000132	0.03764	0.07496	-0.00039	-4.01E-05	-0.0002
p__Actinobacteria	g__Thermasporomyces	0.0006391	0.0002168	0.0004429	0.000122	0.03764	0.07496	-0.00034	-5.30E-05	-0.0002
p__Actinobacteria	g__Sediminihabitans	0.000292	0.0001151	0.0001027	6.51E-05	0.001008	0.00518	-0.00027	-0.00011	-0.00019
p__Actinobacteria	g__Citricoccus	0.0003164	5.02E-05	0.0001281	6.27E-05	0.000183	0.002329	-0.00024	-0.00014	-0.00019
p__Actinobacteria	g__Angustibacter	0.0003998	0.0001564	0.0002163	6.09E-05	0.007285	0.02069	-0.00029	-7.85E-05	-0.00018
p__Actinobacteria	g__Acidithrix	0.0004477	0.0001151	0.0002674	0.000105	0.004586	0.01474	-0.00027	-8.07E-05	-0.00018
p__Actinobacteria	g__Pseudopropionibacterium	0.0002531	0.0001563	8.91E-05	2.38E-05	0.00044	0.003169	-0.00027	-7.39E-05	-0.00016
p__Actinobacteria	g__Sanguibacter	0.0006139	0.000154	0.0004531	8.97E-05	0.01402	0.03427	-0.00027	-5.73E-05	-0.00016
p__Actinobacteria	g__Lapillicoccus	0.0004809	0.0001837	0.0003242	0.000127	0.03764	0.07496	-0.0003	-2.27E-05	-0.00016
p__Actinobacteria	g__Actinorugispora	0.0002815	0.0002192	0.0001254	5.51E-05	0.03764	0.07496	-0.00032	-4.05E-05	-0.00016
p__Actinobacteria	g__Catenuloplanes	0.000394	0.0001174	0.0002419	6.75E-05	0.005795	0.0177	-0.00024	-7.19E-05	-0.00015
p__Actinobacteria	g__Spirillospora	0.0003214	0.0001544	0.0001705	9.88E-05	0.03764	0.07496	-0.00027	-3.72E-05	-0.00015
p__Actinobacteria	g__Hoyosella	0.0002482	4.98E-05	0.0001044	3.76E-05	0.000183	0.002329	-0.00018	-0.00011	-0.00014
p__Actinobacteria	g__Millisia	0.0002306	3.71E-05	9.25E-05	5.52E-05	0.00044	0.003169	-0.00018	-9.99E-05	-0.00014
p__Actinobacteria	g__Barrientosiimonas	0.0002879	0.0001426	0.0001532	7.57E-05	0.02113	0.04755	-0.00023	-4.08E-05	-0.00013
p__Actinobacteria	g__unclassified_f__Coriobacteriaceae	0.0002031	7.27E-05	6.91E-05	3.21E-05	0.001706	0.007373	-0.00018	-8.77E-05	-0.00013
p__Actinobacteria	g__Marisediminicola	0.0001971	6.84E-05	6.81E-05	3.21E-05	0.000183	0.002329	-0.00018	-8.89E-05	-0.00013
p__Actinobacteria	g__Acidimicrobium	0.0003484	0.0001047	0.0002196	9.33E-05	0.007285	0.02069	-0.00021	-4.69E-05	-0.00013
p__Actinobacteria	g__Egicoccus	0.0003173	0.0001077	0.0001887	6.26E-05	0.007285	0.02069	-0.00021	-5.67E-05	-0.00013
p__Actinobacteria	g__Flavimobilis	0.0001657	7.28E-05	4.47E-05	2.82E-05	0.000769	0.004362	-0.00017	-7.62E-05	-0.00012
p__Actinobacteria	g__Amnibacterium	0.0001824	6.81E-05	6.23E-05	3.48E-05	0.00033	0.002769	-0.00017	-7.38E-05	-0.00012
p__Actinobacteria	g__Propionicicella	0.000152	0.0001007	3.52E-05	2.39E-05	0.002202	0.008721	-0.00018	-5.58E-05	-0.00012
p__Actinobacteria	g__Cutibacterium	0.0002368	0.0001095	0.0001232	7.26E-05	0.02575	0.05574	-0.00019	-3.28E-05	-0.00011
p__Actinobacteria	g__Humibacter	0.0001851	0.0001545	7.65E-05	2.64E-05	0.01726	0.0402	-0.00022	-2.55E-05	-0.00011
p__Actinobacteria	g__Subtercola	0.0003009	9.94E-05	0.0001925	5.91E-05	0.03121	0.06485	-0.00018	-3.84E-05	-0.00011
p__Actinobacteria	g__Paenarthrobacter	0.0002401	0.0001159	0.0001393	4.80E-05	0.04515	0.08665	-0.00018	-2.99E-05	-0.0001
p__Actinobacteria	g__Xylanimonas	0.0001773	6.37E-05	8.26E-05	4.94E-05	0.001706	0.007373	-0.00014	-4.70E-05	-9.47E-05
p__Actinobacteria	g__Luteimicrobium	0.0001741	5.27E-05	8.11E-05	4.78E-05	0.002202	0.008721	-0.00014	-4.67E-05	-9.29E-05
p__Actinobacteria	g__Microcella	0.0001787	0.0001164	8.58E-05	4.15E-05	0.01133	0.02909	-0.00017	-2.71E-05	-9.29E-05
p__Actinobacteria	g__Olsenella	0.0001579	9.15E-05	6.52E-05	4.78E-05	0.01402	0.03427	-0.00016	-3.60E-05	-9.28E-05
p__Actinobacteria	g__unclassified_f__Actinomycetaceae	0.0001239	0.000122	3.49E-05	3.11E-05	0.01726	0.0402	-0.00017	-1.90E-05	-8.90E-05
p__Actinobacteria	g__Myceligenerans	0.0001364	6.57E-05	4.89E-05	2.73E-05	0.001008	0.00518	-0.00013	-4.65E-05	-8.76E-05
p__Actinobacteria	g__Marinactinospora	0.0002286	8.67E-05	0.0001421	6.52E-05	0.02113	0.04755	-0.00015	-2.74E-05	-8.66E-05
p__Actinobacteria	g__Dermacoccus	0.0001791	4.73E-05	9.32E-05	5.42E-05	0.003611	0.01236	-0.00013	-4.38E-05	-8.59E-05
p__Actinobacteria	g__Rothia_f__Micrococcaceae	0.0001401	9.27E-05	6.85E-05	3.92E-05	0.02113	0.04755	-0.00014	-2.04E-05	-7.17E-05
p__Actinobacteria	g__Miniimonas	8.20E-05	2.93E-05	1.31E-05	7.22E-06	0.000183	0.002329	-8.71E-05	-5.19E-05	-6.90E-05
p__Actinobacteria	g__unclassified_o__Candidatus_Nano	9.82E-05	3.02E-05	3.09E-05	1.78E-05	0.000246	0.00248	-8.94E-05	-4.77E-05	-6.73E-05
p__Actinobacteria	g__Yonghaparkia	9.08E-05	4.99E-05	2.47E-05	3.55E-05	0.001706	0.007373	-0.0001	-2.78E-05	-6.62E-05
p__Actinobacteria	g__Granulicoccus	7.79E-05	4.89E-05	1.40E-05	8.05E-06	0.000583	0.003709	-9.22E-05	-3.30E-05	-6.39E-05
p__Actinobacteria	g__Actinobaculum	7.24E-05	0.0001207	1.02E-05	1.10E-05	0.007285	0.02069	-0.00015	-9.67E-06	-6.22E-05
p__Actinobacteria	g__Kribbia	8.96E-05	4.02E-05	3.16E-05	2.67E-05	0.003611	0.01236	-8.71E-05	-2.88E-05	-5.80E-05
p__Actinobacteria	g__Tersicoccus	9.76E-05	3.70E-05	4.12E-05	2.52E-05	0.002827	0.01032	-8.35E-05	-3.00E-05	-5.64E-05
p__Actinobacteria	g__Homoserinibacter	6.64E-05	5.68E-05	1.00E-05	1.02E-05	0.01121	0.02909	-9.13E-05	-2.30E-05	-5.64E-05
p__Actinobacteria	g__Xylanimicrobium	8.71E-05	3.61E-05	3.18E-05	2.58E-05	0.001706	0.007373	-8.26E-05	-2.84E-05	-5.53E-05
p__Actinobacteria	g__Segeticoccus	8.63E-05	4.91E-05	3.36E-05	1.33E-05	0.004586	0.01474	-8.45E-05	-2.39E-05	-5.27E-05
p__Actinobacteria	g__Propionimicrobium	7.65E-05	4.60E-05	2.42E-05	2.20E-05	0.003611	0.01236	-8.25E-05	-2.40E-05	-5.23E-05
p__Actinobacteria	g__Epidermidibacterium	0.0001047	3.45E-05	5.37E-05	2.69E-05	0.004586	0.01474	-7.55E-05	-2.60E-05	-5.10E-05

p__Actinobacteria	g__Mycolicibacillus	0.0001862	3.99E-05	0.0001373	0.000123	0.02575	0.05574	-0.00012	3.18E-05	-4.90E-05
p__Actinobacteria	g__Raineyella	0.0001321	4.91E-05	8.73E-05	2.69E-05	0.02575	0.05574	-7.81E-05	-1.30E-05	-4.49E-05
p__Actinobacteria	g__Trueperella	5.05E-05	5.08E-05	6.61E-06	1.16E-05	0.006109	0.01857	-7.58E-05	-1.44E-05	-4.39E-05
p__Actinobacteria	g__Zhihengliuella	5.00E-05	3.38E-05	7.06E-06	5.84E-06	0.001293	0.006216	-6.47E-05	-2.36E-05	-4.30E-05
p__Actinobacteria	g__Naumannella	7.83E-05	4.06E-05	3.98E-05	2.42E-05	0.02575	0.05574	-6.55E-05	-8.16E-06	-3.85E-05
p__Actinobacteria	g__Eggerthella	5.24E-05	4.30E-05	1.46E-05	1.79E-05	0.01709	0.0402	-6.58E-05	-9.20E-06	-3.78E-05
p__Actinobacteria	g__Acidipropionibacterium	5.94E-05	5.10E-05	2.30E-05	1.49E-05	0.007285	0.02069	-7.35E-05	-1.18E-05	-3.65E-05
p__Actinobacteria	g__Schumannella	0.0001607	6.32E-05	0.0001248	5.85E-05	0.03121	0.06485	-8.56E-05	1.75E-05	-3.59E-05
p__Actinobacteria	g__Serinibacter	6.12E-05	3.23E-05	2.85E-05	1.87E-05	0.01133	0.02909	-5.52E-05	-9.05E-06	-3.28E-05
p__Actinobacteria	g__Kineosphaera	7.06E-05	3.21E-05	4.17E-05	1.86E-05	0.04515	0.08665	-5.04E-05	-7.73E-06	-2.89E-05
p__Actinobacteria	g__Devriesea	3.13E-05	2.35E-05	4.70E-06	5.96E-06	0.002811	0.01032	-4.18E-05	-1.24E-05	-2.66E-05
p__Actinobacteria	g__Dermabacter	3.67E-05	2.99E-05	1.05E-05	1.09E-05	0.01722	0.0402	-4.46E-05	-9.19E-06	-2.62E-05
p__Actinobacteria	g__Demetria	5.19E-05	2.18E-05	2.73E-05	2.41E-05	0.02575	0.05574	-4.19E-05	-5.70E-06	-2.46E-05
p__Actinobacteria	g__Flaviflexus	3.60E-05	2.08E-05	1.16E-05	1.11E-05	0.004571	0.01474	-3.99E-05	-1.15E-05	-2.44E-05
p__Actinobacteria	g__Ponticoccus_f__Propionibacteriace	3.18E-05	2.70E-05	7.61E-06	7.16E-06	0.002194	0.008721	-4.15E-05	-8.74E-06	-2.42E-05
p__Actinobacteria	g__Polymorphospora	2.50E-05	2.32E-05	3.35E-06	5.46E-06	0.007922	0.02245	-3.57E-05	-9.03E-06	-2.16E-05
p__Actinobacteria	g__Lysinibacter	2.57E-05	2.34E-05	6.37E-06	1.04E-05	0.04642	0.08904	-3.46E-05	-3.07E-06	-1.93E-05
p__Actinobacteria	g__Gardnerella	3.22E-05	1.42E-05	1.43E-05	1.41E-05	0.01133	0.02909	-2.98E-05	-5.90E-06	-1.80E-05
p__Actinobacteria	g__Renibacterium	2.06E-05	1.57E-05	3.75E-06	5.39E-06	0.004815	0.01547	-2.70E-05	-7.16E-06	-1.68E-05
p__Actinobacteria	g__Adlercreutzia	2.05E-05	1.48E-05	4.92E-06	7.53E-06	0.007334	0.0208	-2.57E-05	-5.94E-06	-1.56E-05
p__Actinobacteria	g__Haematomicrobium	1.48E-05	1.03E-05	4.39E-06	9.79E-06	0.01245	0.03191	-1.84E-05	-2.16E-06	-1.04E-05
p__Actinobacteria	g__Rhodoluna	2.80E-06	4.09E-06	0	0	0.03498	0.07215	-5.36E-06	-4.73E-07	-2.80E-06
p__Actinobacteria	g__Protaetiibacter	8.92E-06	1.16E-05	7.77E-06	2.17E-05	0.04329	0.08576	-1.34E-05	1.59E-05	-1.15E-06
p__Nitrospinae	g__unclassified_p__Nitrospinae	0.009499	0.001142	0.00712	0.001108	0.001315	0.006216	-0.00331	-0.0015	-0.00238
p__Nitrospinae	g__Nitrospina	0.002451	0.0004616	0.001462	0.000141	0.000183	0.002329	-0.00127	-0.00071	-0.00099
p__Nitrospinae	g__unclassified_f__Nitrospinaceae	0.0007067	0.0001814	0.0004043	0.000192	0.002827	0.01032	-0.00045	-0.00014	-0.0003
p__Nitrospirae	g__unclassified_o__Nitrospirales	0.003378	0.0006784	0.002637	0.00047	0.02113	0.04755	-0.00122	-0.00026	-0.00074
p__Firmicutes	g__Propionispora	0.003466	0.00198	0.0002242	0.000138	0.000183	0.002329	-0.00461	-0.00213	-0.00324
p__Firmicutes	g__Desulfotomaculum	0.003762	0.001545	0.001704	0.000677	0.001008	0.00518	-0.00321	-0.00115	-0.00206
p__Firmicutes	g__Kurthia	0.00394	0.001062	0.002317	0.00025	0.000246	0.00248	-0.00232	-0.00098	-0.00162
p__Firmicutes	g__Staphylococcus	0.002906	0.0003949	0.001344	0.000352	0.000183	0.002329	-0.00187	-0.00125	-0.00156
p__Firmicutes	g__Tumebacillus	0.002507	0.0006737	0.001151	0.000282	0.000183	0.002329	-0.00179	-0.00094	-0.00136
p__Firmicutes	g__Oceanobacillus	0.003274	0.0004891	0.002017	0.000637	0.001315	0.006216	-0.00175	-0.00074	-0.00126
p__Firmicutes	g__Domibacillus	0.001928	0.0005166	0.0008516	0.000261	0.00033	0.002769	-0.0014	-0.00076	-0.00108
p__Firmicutes	g__unclassified_f__Ruminococcaceae	0.001206	0.0006388	0.0004109	0.000128	0.00044	0.003169	-0.00118	-0.00044	-0.00079
p__Firmicutes	g__Geobacillus	0.001599	0.0002718	0.0008645	0.000157	0.000183	0.002329	-0.00092	-0.00055	-0.00073
p__Firmicutes	g__Thermoanaerosceptrum	0.001155	0.0004551	0.0004247	9.77E-05	0.000183	0.002329	-0.00102	-0.00046	-0.00073
p__Firmicutes	g__Moorella	0.002816	0.0005122	0.002099	0.000588	0.01726	0.0402	-0.00113	-0.00021	-0.00072
p__Firmicutes	g__Aneurinibacillus	0.002135	0.0005134	0.00145	0.000236	0.001315	0.006216	-0.00102	-0.00036	-0.00069
p__Firmicutes	g__Alicyclobacillus	0.002338	0.0003332	0.001748	0.00026	0.001008	0.00518	-0.00085	-0.00035	-0.00059
p__Firmicutes	g__Streptococcus	0.002318	0.0004056	0.001781	0.000281	0.007285	0.02069	-0.00083	-0.00026	-0.00054
p__Firmicutes	g__unclassified_f__Veillonellaceae	0.0009143	0.0002282	0.0003992	8.09E-05	0.000183	0.002329	-0.00065	-0.00037	-0.00052
p__Firmicutes	g__Heliobacterium	0.001004	0.000138	0.000509	0.000109	0.000183	0.002329	-0.00059	-0.0004	-0.00049
p__Firmicutes	g__Pelotomaculum	0.002729	0.0004956	0.002245	0.000251	0.02113	0.04755	-0.00083	-0.00016	-0.00048
p__Firmicutes	g__Sporosarcina	0.00068	0.0005031	0.0002433	7.34E-05	0.01402	0.03427	-0.00076	-0.00016	-0.00044
p__Firmicutes	g__Desnuesiella	0.0008396	0.0004127	0.0004227	0.000192	0.009108	0.02457	-0.0007	-0.00017	-0.00042
p__Firmicutes	g__unclassified_f__Lachnospiraceae	0.001395	0.000202	0.0009977	0.000154	0.001008	0.00518	-0.00054	-0.00026	-0.0004
p__Firmicutes	g__Megasphaera	0.0004938	0.0001466	0.0001483	8.43E-05	0.00033	0.002769	-0.00045	-0.00024	-0.00035

p__Firmicutes	g__Dethiobacter	0.0005335	0.0001284	0.0002242	7.66E-05	0.00033	0.002769	-0.0004	-0.00022	-0.00031
p__Firmicutes	g__Alteribacillus	0.0007139	0.0001776	0.0004275	0.000153	0.003611	0.01236	-0.00042	-0.00014	-0.00029
p__Firmicutes	g__Enterococcus	0.000586	0.0001922	0.0003303	0.000124	0.003611	0.01236	-0.00039	-0.00011	-0.00026
p__Firmicutes	g__Salicibibacter	0.0002707	0.0001626	2.01E-05	1.09E-05	0.000183	0.002329	-0.00035	-0.00016	-0.00025
p__Firmicutes	g__Sporotomaculum	0.0003219	0.0003395	8.04E-05	6.37E-05	0.02575	0.05574	-0.00046	-5.88E-05	-0.00024
p__Firmicutes	g__Metabacillus	0.0003339	0.0001235	0.000114	5.35E-05	0.000583	0.003709	-0.0003	-0.00014	-0.00022
p__Firmicutes	g__Vallitalea	0.0003794	0.0001807	0.0001705	7.93E-05	0.002202	0.008721	-0.00033	-9.25E-05	-0.00021
p__Firmicutes	g__Symbiobacterium	0.0008617	0.0002147	0.0006803	0.000144	0.04515	0.08665	-0.00033	-2.40E-05	-0.00018
p__Firmicutes	g__Numidum	0.0004907	0.0001404	0.000312	8.64E-05	0.01402	0.03427	-0.00028	-8.82E-05	-0.00018
p__Firmicutes	g__Anoxybacillus	0.0004676	0.0001101	0.0002905	7.70E-05	0.001315	0.006216	-0.00026	-9.71E-05	-0.00018
p__Firmicutes	g__Halothermothrix	0.0002405	0.0001719	6.74E-05	3.57E-05	0.000769	0.004362	-0.0003	-8.30E-05	-0.00017
p__Firmicutes	g__Desulfurispora	0.0003521	0.0002108	0.0001806	0.000111	0.02575	0.05574	-0.00032	-4.67E-05	-0.00017
p__Firmicutes	g__Kyrpidia	0.0008099	0.0001433	0.0006399	0.000172	0.04515	0.08665	-0.00031	-3.82E-05	-0.00017
p__Firmicutes	g__Propionispira	0.0002627	0.0001393	0.0001031	5.96E-05	0.02575	0.05574	-0.00024	-6.85E-05	-0.00016
p__Firmicutes	g__Anaerotruncus	0.0003628	0.000128	0.0002036	3.93E-05	0.001706	0.007373	-0.00024	-7.88E-05	-0.00016
p__Firmicutes	g__Salipaludibacillus	0.0001832	0.0001184	2.83E-05	1.86E-05	0.00033	0.002769	-0.00022	-8.62E-05	-0.00015
p__Firmicutes	g__Mesobacillus	0.00029	9.95E-05	0.000137	3.93E-05	0.00044	0.003169	-0.00022	-9.21E-05	-0.00015
p__Firmicutes	g__Oscillibacter	0.0001701	8.46E-05	3.66E-05	1.36E-05	0.000183	0.002329	-0.00019	-8.47E-05	-0.00013
p__Firmicutes	g__Clostridioides	0.0002994	8.23E-05	0.0001756	5.97E-05	0.002827	0.01032	-0.00018	-6.79E-05	-0.00012
p__Firmicutes	g__Heliophilum	0.0001421	0.0001494	2.22E-05	1.79E-05	0.003611	0.01236	-0.00022	-3.55E-05	-0.00012
p__Firmicutes	g__Thermincola	0.0002482	8.21E-05	0.0001284	3.63E-05	0.001706	0.007373	-0.00017	-6.39E-05	-0.00012
p__Firmicutes	g__Thermovenabulum	0.0001237	0.0001235	1.22E-05	1.13E-05	0.002194	0.008721	-0.00019	-4.32E-05	-0.00011
p__Firmicutes	g__Orenia	0.0001251	0.0001063	1.65E-05	6.73E-06	0.000583	0.003709	-0.00018	-5.28E-05	-0.00011
p__Firmicutes	g__Planifilum	0.0006021	0.000152	0.0004965	0.000107	0.04515	0.08665	-0.00022	6.46E-06	-0.00011
p__Firmicutes	g__Caldanaerobacter	0.0001653	8.32E-05	6.17E-05	2.63E-05	0.001706	0.007373	-0.00015	-5.16E-05	-0.0001
p__Firmicutes	g__Agitococcus	0.0003665	0.0001196	0.0002653	8.08E-05	0.04515	0.08665	-0.0002	-1.83E-05	-0.0001
p__Firmicutes	g__Thermosinus	0.0001295	7.69E-05	3.40E-05	2.52E-05	0.001008	0.00518	-0.00015	-5.06E-05	-9.55E-05
p__Firmicutes	g__unclassified_f__Sporomusaceae	0.0001014	5.56E-05	5.93E-06	6.48E-06	0.000173	0.002329	-0.00013	-6.42E-05	-9.54E-05
p__Firmicutes	g__Psychrobacillus	0.0001171	4.31E-05	2.57E-05	3.20E-05	0.000769	0.004362	-0.00012	-5.74E-05	-9.14E-05
p__Firmicutes	g__unclassified_c__Erysipelotrichia	0.0002107	9.25E-05	0.000121	9.72E-05	0.03764	0.07496	-0.00017	-1.41E-05	-8.97E-05
p__Firmicutes	g__Leuconostoc	8.85E-05	7.83E-05	0	0	6.39E-05	0.002329	-0.00014	-4.74E-05	-8.85E-05
p__Firmicutes	g__Fontibacillus	0.0001695	0.0001015	8.24E-05	4.51E-05	0.03764	0.07496	-0.00016	-2.72E-05	-8.72E-05
p__Firmicutes	g__Acidibacillus	0.0002438	8.31E-05	0.0001615	6.62E-05	0.02575	0.05574	-0.00015	-2.00E-05	-8.23E-05
p__Firmicutes	g__Desulfitibacter	9.74E-05	6.48E-05	1.55E-05	1.26E-05	0.000183	0.002329	-0.00012	-4.61E-05	-8.20E-05
p__Firmicutes	g__Lucifera	0.0001676	4.31E-05	8.85E-05	4.16E-05	0.004586	0.01474	-0.00011	-4.25E-05	-7.91E-05
p__Firmicutes	g__Listeria	0.0004906	0.0001019	0.0004154	4.67E-05	0.04515	0.08665	-0.00014	-1.33E-05	-7.52E-05
p__Firmicutes	g__Brockia	0.0001739	6.74E-05	0.0001078	3.63E-05	0.02575	0.05574	-0.00011	-2.13E-05	-6.61E-05
p__Firmicutes	g__Chengkuizengella	0.000132	7.85E-05	6.63E-05	1.91E-05	0.04515	0.08665	-0.00012	-1.94E-05	-6.57E-05
p__Firmicutes	g__Butyrivibrio	0.0001538	5.23E-05	9.43E-05	2.17E-05	0.01402	0.03427	-9.22E-05	-2.20E-05	-5.95E-05
p__Firmicutes	g__Caldicoprobacter	0.0001476	6.25E-05	8.84E-05	6.61E-05	0.03764	0.07496	-0.00011	-5.94E-06	-5.93E-05
p__Firmicutes	g__Tuberibacillus	6.55E-05	2.05E-05	6.63E-06	1.29E-05	0.000132	0.002329	-7.31E-05	-4.52E-05	-5.89E-05
p__Firmicutes	g__Youngiibacter	0.0001084	3.75E-05	4.96E-05	3.23E-05	0.009108	0.02457	-8.66E-05	-2.64E-05	-5.87E-05
p__Firmicutes	g__Oxobacter	6.99E-05	3.53E-05	1.17E-05	9.14E-06	0.00044	0.003169	-7.86E-05	-3.67E-05	-5.82E-05
p__Firmicutes	g__Oribacterium	0.0001704	4.58E-05	0.0001136	3.75E-05	0.01402	0.03427	-9.38E-05	-2.04E-05	-5.68E-05
p__Firmicutes	g__Veillonella	9.36E-05	3.81E-05	3.70E-05	2.01E-05	0.001315	0.006216	-8.24E-05	-3.13E-05	-5.67E-05
p__Firmicutes	g__Acidaminococcus	7.72E-05	5.06E-05	2.15E-05	1.57E-05	0.000246	0.00248	-9.08E-05	-3.01E-05	-5.58E-05
p__Firmicutes	g__Aquibacillus	9.31E-05	3.61E-05	3.85E-05	1.59E-05	0.000769	0.004362	-7.70E-05	-3.33E-05	-5.47E-05
p__Firmicutes	g__Metasolibacillus	7.89E-05	2.83E-05	2.52E-05	1.56E-05	0.000769	0.004362	-7.19E-05	-3.31E-05	-5.37E-05

p__Firmicutes	g__Megamonas	5.94E-05	5.85E-05	6.66E-06	1.15E-05	0.00176	0.007593	-8.92E-05	-2.34E-05	-5.27E-05
p__Firmicutes	g__Viridibacillus	6.58E-05	4.48E-05	1.66E-05	1.32E-05	0.001706	0.007373	-7.97E-05	-2.25E-05	-4.92E-05
p__Firmicutes	g__Dehalobacterium	0.0001167	7.20E-05	6.77E-05	9.35E-05	0.04515	0.08665	-0.00011	2.23E-05	-4.90E-05
p__Firmicutes	g__Paenisporosarcina	9.67E-05	4.82E-05	5.26E-05	3.46E-05	0.03764	0.07496	-7.77E-05	-1.02E-05	-4.40E-05
p__Firmicutes	g__Phascolarctobacterium	8.02E-05	4.58E-05	3.82E-05	2.15E-05	0.01133	0.02909	-7.41E-05	-1.56E-05	-4.21E-05
p__Firmicutes	g__Thermoclostridium	6.16E-05	2.46E-05	1.97E-05	1.78E-05	0.002194	0.008721	-6.07E-05	-2.25E-05	-4.19E-05
p__Firmicutes	g__Roseburia	6.94E-05	3.08E-05	2.96E-05	2.62E-05	0.004586	0.01474	-6.36E-05	-1.52E-05	-3.98E-05
p__Firmicutes	g__Planomicrobium	6.97E-05	3.70E-05	2.99E-05	2.13E-05	0.01133	0.02909	-6.58E-05	-1.46E-05	-3.97E-05
p__Firmicutes	g__Gelria	7.34E-05	4.41E-05	3.45E-05	3.17E-05	0.02575	0.05574	-7.10E-05	-6.60E-06	-3.89E-05
p__Firmicutes	g__Defluviitalea	3.85E-05	4.69E-05	1.20E-06	3.80E-06	0.006328	0.01919	-6.69E-05	-1.16E-05	-3.73E-05
p__Firmicutes	g__unclassified_f__Tissierellaceae	5.37E-05	3.01E-05	1.68E-05	1.53E-05	0.003611	0.01236	-5.79E-05	-1.82E-05	-3.69E-05
p__Firmicutes	g__Filobacillus	5.61E-05	4.61E-05	2.01E-05	2.54E-05	0.01398	0.03427	-6.94E-05	-6.69E-06	-3.61E-05
p__Firmicutes	g__Lactococcus	4.48E-05	2.29E-05	9.17E-06	7.44E-06	0.001008	0.00518	-5.05E-05	-2.26E-05	-3.56E-05
p__Firmicutes	g__Emergencia	3.48E-05	4.62E-05	0	0	0.000751	0.004362	-6.57E-05	-1.00E-05	-3.48E-05
p__Firmicutes	g__Clostridiisalibacter	5.45E-05	1.45E-05	2.07E-05	1.26E-05	0.000583	0.003709	-4.55E-05	-2.16E-05	-3.38E-05
p__Firmicutes	g__Peptoclostridium	4.60E-05	1.96E-05	1.35E-05	1.19E-05	0.003611	0.01236	-4.54E-05	-1.82E-05	-3.25E-05
p__Firmicutes	g__Butyricicoccus	4.22E-05	1.38E-05	9.95E-06	1.18E-05	0.00058	0.003709	-4.23E-05	-2.12E-05	-3.23E-05
p__Firmicutes	g__Soehngenia	6.43E-05	2.66E-05	3.23E-05	2.12E-05	0.004586	0.01474	-5.22E-05	-1.15E-05	-3.20E-05
p__Firmicutes	g__Hydrogenoanaerobacterium	3.33E-05	3.70E-05	2.65E-06	8.39E-06	0.01048	0.02822	-5.39E-05	-1.05E-05	-3.07E-05
p__Firmicutes	g__Erysipelothrix	7.87E-05	3.94E-05	4.81E-05	6.09E-05	0.03121	0.06485	-7.07E-05	1.58E-05	-3.06E-05
p__Firmicutes	g__unclassified_f__Halanaerobiaceae	6.22E-05	2.06E-05	3.29E-05	2.53E-05	0.01726	0.0402	-4.73E-05	-1.05E-05	-2.93E-05
p__Firmicutes	g__Proteiniborus	4.83E-05	2.60E-05	1.95E-05	1.24E-05	0.004586	0.01474	-4.76E-05	-1.26E-05	-2.88E-05
p__Firmicutes	g__Caproiciproducens	3.13E-05	1.91E-05	3.05E-06	3.62E-06	0.000163	0.002329	-3.97E-05	-1.81E-05	-2.82E-05
p__Firmicutes	g__Massilibacillus	3.47E-05	2.67E-05	6.44E-06	1.18E-05	0.007334	0.0208	-4.49E-05	-1.07E-05	-2.82E-05
p__Firmicutes	g__Gallicola	4.18E-05	2.29E-05	1.57E-05	1.93E-05	0.009004	0.02457	-4.37E-05	-8.29E-06	-2.61E-05
p__Firmicutes	g__Acetoanaerobium	2.65E-05	1.59E-05	1.56E-06	3.78E-06	0.000757	0.004362	-3.44E-05	-1.44E-05	-2.50E-05
p__Firmicutes	g__Absiella	2.46E-05	2.67E-05	0	0	0.002213	0.008721	-4.29E-05	-1.00E-05	-2.46E-05
p__Firmicutes	g__Desulfuribacillus	4.49E-05	2.01E-05	2.11E-05	1.35E-05	0.01133	0.02909	-3.80E-05	-8.89E-06	-2.38E-05
p__Firmicutes	g__Anaeromicrobium	4.55E-05	3.06E-05	2.21E-05	1.42E-05	0.04515	0.08665	-4.40E-05	-4.46E-06	-2.34E-05
p__Firmicutes	g__Indiicoccus	5.35E-05	2.23E-05	3.04E-05	2.65E-05	0.02113	0.04755	-4.20E-05	-1.32E-06	-2.31E-05
p__Firmicutes	g__Facklamia	2.49E-05	1.72E-05	3.02E-06	4.15E-06	0.002811	0.01032	-3.21E-05	-1.18E-05	-2.19E-05
p__Firmicutes	g__Tetragenococcus	2.44E-05	2.07E-05	2.66E-06	5.97E-06	0.002959	0.01079	-3.56E-05	-8.69E-06	-2.17E-05
p__Firmicutes	g__Agathobaculum	2.49E-05	2.20E-05	4.64E-06	7.46E-06	0.0239	0.05364	-3.51E-05	-6.77E-06	-2.03E-05
p__Firmicutes	g__Tenuibacillus	2.03E-05	1.67E-05	7.71E-07	2.44E-06	0.000311	0.002769	-2.99E-05	-9.80E-06	-1.95E-05
p__Firmicutes	g__Longirhabdus	2.17E-05	2.07E-05	3.80E-06	5.55E-06	0.01207	0.03096	-3.06E-05	-6.07E-06	-1.79E-05
p__Firmicutes	g__Melghiribacillus	1.81E-05	1.40E-05	4.40E-07	1.39E-06	0.000121	0.002329	-2.68E-05	-9.52E-06	-1.77E-05
p__Firmicutes	g__Jeotgalicoccus	1.91E-05	2.20E-05	1.60E-06	5.07E-06	0.01335	0.03409	-3.17E-05	-5.40E-06	-1.75E-05
p__Firmicutes	g__Anaerococcus	2.27E-05	1.53E-05	5.47E-06	5.54E-06	0.005613	0.0177	-2.74E-05	-8.02E-06	-1.73E-05
p__Firmicutes	g__Candidatus_Arthromitus	1.65E-05	2.17E-05	4.43E-07	1.40E-06	0.01472	0.03593	-2.96E-05	-4.89E-06	-1.61E-05
p__Firmicutes	g__unclassified_f__Selenomonadacea	2.22E-05	1.32E-05	6.33E-06	6.49E-06	0.002786	0.01032	-2.52E-05	-7.89E-06	-1.58E-05
p__Firmicutes	g__Salisediminibacterium	1.94E-05	1.98E-05	3.68E-06	6.33E-06	0.02603	0.0563	-2.87E-05	-4.09E-06	-1.57E-05
p__Firmicutes	g__Anaerocolumna	3.22E-05	1.48E-05	1.74E-05	1.47E-05	0.03121	0.06485	-2.64E-05	-2.13E-06	-1.48E-05
p__Firmicutes	g__Thermacetogenium	2.12E-05	1.28E-05	6.51E-06	8.63E-06	0.01072	0.02883	-2.39E-05	-5.19E-06	-1.47E-05
p__Firmicutes	g__Christensenella	1.41E-05	8.61E-06	7.71E-07	2.44E-06	0.000167	0.002329	-1.86E-05	-7.94E-06	-1.33E-05
p__Firmicutes	g__Caryophanon	1.54E-05	2.15E-05	2.58E-06	3.03E-06	0.02772	0.05985	-2.85E-05	-2.11E-06	-1.28E-05
p__Firmicutes	g__Acetobacterium	3.23E-05	9.32E-06	1.97E-05	1.66E-05	0.03764	0.07496	-2.36E-05	1.78E-07	-1.26E-05
p__Firmicutes	g__Halonatronum	2.11E-05	1.70E-05	8.59E-06	1.77E-05	0.01331	0.03401	-2.68E-05	2.68E-06	-1.25E-05
p__Firmicutes	g__Scopulibacillus	1.36E-05	2.01E-05	2.57E-06	2.98E-06	0.0227	0.05104	-2.47E-05	-1.51E-06	-1.10E-05

p__Firmicutes	g__Pediococcus	1.20E-05	1.16E-05	1.32E-06	4.16E-06	0.004307	0.0147	-1.77E-05	-3.69E-06	-1.07E-05
p__Firmicutes	g__Colidextribacter	1.20E-05	1.22E-05	2.07E-06	4.44E-06	0.02832	0.06108	-1.78E-05	-2.25E-06	-9.90E-06
p__Firmicutes	g__Colibacter	9.64E-06	7.55E-06	0	0	0.000751	0.004362	-1.41E-05	-5.00E-06	-9.64E-06
p__Firmicutes	g__Dethiosulfatibacter	1.25E-05	6.99E-06	2.96E-06	3.45E-06	0.003363	0.01221	-1.42E-05	-4.82E-06	-9.49E-06
p__Firmicutes	g__Thermosyntropha	9.28E-06	1.31E-05	0	0	0.01493	0.03629	-1.72E-05	-2.17E-06	-9.28E-06
p__Firmicutes	g__Fusibacter	1.00E-05	1.17E-05	8.01E-07	2.53E-06	0.01048	0.02822	-1.66E-05	-2.69E-06	-9.22E-06
p__Firmicutes	g__Anaerostipes	1.43E-05	9.54E-06	5.54E-06	5.63E-06	0.03514	0.07239	-1.53E-05	-2.08E-06	-8.77E-06
p__Firmicutes	g__unclassified_o__Erysipelotrichales	8.60E-06	9.59E-06	0	0	0.002213	0.008721	-1.48E-05	-3.62E-06	-8.60E-06
p__Firmicutes	g__Natribacillus	1.00E-05	1.35E-05	1.76E-06	4.24E-06	0.03486	0.07215	-1.73E-05	-9.78E-07	-8.27E-06
p__Firmicutes	g__Candidatus_Syntrophocurvum	8.12E-06	8.30E-06	0	0	0.005972	0.01818	-1.32E-05	-2.89E-06	-8.12E-06
p__Firmicutes	g__Lawsonibacter	7.99E-06	1.10E-05	0	0	0.01493	0.03629	-1.55E-05	-1.87E-06	-7.99E-06
p__Firmicutes	g__Bittarella	8.00E-06	7.94E-06	3.85E-07	1.22E-06	0.001916	0.008236	-1.27E-05	-3.39E-06	-7.62E-06
p__Firmicutes	g__Herbinix	7.45E-06	7.67E-06	0	0	0.002213	0.008721	-1.24E-05	-3.17E-06	-7.45E-06
p__Firmicutes	g__Oenococcus	1.08E-05	7.35E-06	3.37E-06	4.43E-06	0.01423	0.03476	-1.23E-05	-2.61E-06	-7.43E-06
p__Firmicutes	g__Centipeda_f__Selenomonadaceae	7.73E-06	9.61E-06	4.42E-07	1.40E-06	0.04033	0.08009	-1.30E-05	-2.23E-06	-7.29E-06
p__Firmicutes	g__Eggerthia	7.08E-06	1.35E-05	0	0	0.03498	0.07215	-1.65E-05	-1.15E-06	-7.08E-06
p__Firmicutes	g__Weissella	8.24E-06	9.19E-06	1.28E-06	2.06E-06	0.03573	0.07356	-1.30E-05	-1.76E-06	-6.96E-06
p__Firmicutes	g__Massilimaliae	7.18E-06	8.56E-06	4.39E-07	1.39E-06	0.04033	0.08009	-1.16E-05	-1.68E-06	-6.74E-06
p__Firmicutes	g__Atopococcus	6.73E-06	1.19E-05	0	0	0.01493	0.03629	-1.51E-05	-1.33E-06	-6.73E-06
p__Firmicutes	g__Bavariicoccus	6.33E-06	1.11E-05	0	0	0.03498	0.07215	-1.36E-05	-8.62E-07	-6.33E-06
p__Firmicutes	g__Ileibacterium	6.18E-06	6.22E-06	0	0	0.002213	0.008721	-1.01E-05	-2.87E-06	-6.18E-06
p__Firmicutes	g__Caldisalinibacter	7.50E-06	8.69E-06	1.36E-06	4.29E-06	0.02977	0.06411	-1.18E-05	-6.37E-07	-6.14E-06
p__Firmicutes	g__Pectinatus	6.42E-06	9.66E-06	4.39E-07	1.39E-06	0.04033	0.08009	-1.25E-05	-8.84E-07	-5.98E-06
p__Firmicutes	g__Faecalibacillus	7.26E-06	6.63E-06	1.55E-06	3.73E-06	0.03486	0.07215	-1.01E-05	-1.32E-06	-5.71E-06
p__Firmicutes	g__unclassified_f__Christensenellacea	4.23E-06	6.45E-06	0	0	0.03498	0.07215	-8.46E-06	-8.70E-07	-4.23E-06
p__Firmicutes	g__Faecalibaculum	3.85E-06	6.42E-06	0	0	0.03498	0.07215	-8.32E-06	-4.35E-07	-3.85E-06
p__Firmicutes	g__Kallipyga	2.57E-06	3.89E-06	0	0	0.03498	0.07215	-4.98E-06	-4.73E-07	-2.58E-06
p__Chloroflexi	g__unclassified_p__Chloroflexi	0.7567	0.1362	0.5988	0.09081	0.01402	0.03427	-0.2524	-0.06197	-0.1579
p__Chloroflexi	g__unclassified_f__Anaerolineaceae	0.05314	0.02204	0.02604	0.004814	0.000583	0.003709	-0.04043	-0.01485	-0.0271
p__Chloroflexi	g__unclassified_c__Dehalococcoidia	0.04994	0.007426	0.04047	0.008099	0.01726	0.0402	-0.01597	-0.0025	-0.00947
p__Chloroflexi	g__unclassified_c__Candidatus_Therr	0.01346	0.006662	0.004068	0.000918	0.00044	0.003169	-0.01328	-0.00548	-0.00939
p__Chloroflexi	g__unclassified_f__Chloroflexaceae	0.01471	0.001896	0.01192	0.000837	0.001008	0.00518	-0.00403	-0.00152	-0.00279
p__Chloroflexi	g__Litorilinea	0.008673	0.001651	0.006394	0.001161	0.005795	0.0177	-0.00344	-0.00105	-0.00228
p__Chloroflexi	g__Roseiflexus	0.009792	0.001149	0.007844	0.001046	0.003611	0.01236	-0.00287	-0.00105	-0.00195
p__Chloroflexi	g__unclassified_f__Caldilineaceae	0.007645	0.001352	0.006102	0.001041	0.01726	0.0402	-0.00257	-0.00053	-0.00154
p__Chloroflexi	g__Chloroflexus	0.009254	0.001557	0.007762	0.000818	0.02575	0.05574	-0.00251	-0.00041	-0.00149
p__Chloroflexi	g__unclassified_c__Thermoflexia	0.003182	0.0007804	0.001972	0.000425	0.001706	0.007373	-0.00175	-0.00069	-0.00121
p__Chloroflexi	g__Caldilinea	0.003102	0.001029	0.001903	0.000201	0.000583	0.003709	-0.00181	-0.00062	-0.0012
p__Chloroflexi	g__Candidatus_Promineofilum	0.00274	0.000658	0.001853	0.000506	0.003611	0.01236	-0.00135	-0.00037	-0.00089
p__Chloroflexi	g__Thermanaerothrix	0.0009715	0.0003864	0.0001869	5.24E-05	0.000183	0.002329	-0.00101	-0.00056	-0.00078
p__Chloroflexi	g__Thermoflexus	0.002189	0.0005792	0.001552	0.000232	0.001706	0.007373	-0.00104	-0.00027	-0.00064
p__Chloroflexi	g__unclassified_c__Ardenticatenia	0.001259	0.0004013	0.0008341	0.000105	0.01726	0.0402	-0.00069	-0.00017	-0.00042
p__Chloroflexi	g__Tepidiforma	0.00111	0.0002667	0.0006997	0.000107	0.000583	0.003709	-0.00058	-0.00024	-0.00041
p__Chloroflexi	g__Longilinea	0.0009964	0.0003095	0.0007002	0.000194	0.03121	0.06485	-0.00054	-8.75E-05	-0.0003
p__Chloroflexi	g__Bellilinea	0.0008323	0.0001619	0.0005499	0.000267	0.02113	0.04755	-0.00046	-9.31E-05	-0.00028
p__Chloroflexi	g__Levilinea	0.0005011	0.0001325	0.0002807	0.000109	0.002202	0.008721	-0.00033	-0.00012	-0.00022
p__Chloroflexi	g__Pelolinea	0.0003994	7.71E-05	0.0002414	7.42E-05	0.001008	0.00518	-0.00022	-9.21E-05	-0.00016
p__Chloroflexi	g__Brevefilum	6.12E-05	3.17E-05	1.61E-05	1.16E-05	0.001008	0.00518	-6.48E-05	-2.48E-05	-4.51E-05

p__Verrucomicrobia	g__unclassified_o__Methylacidiphilal	0.003367	0.0003194	0.002425	0.000348	0.000183	0.002329	-0.00122	-0.00069	-0.00094
p__Aquificae	g__Phorcysia	0.001491	0.0009247	0.000109	8.01E-05	0.000183	0.002329	-0.00195	-0.00086	-0.00138
p__Aquificae	g__unclassified_f__Aquificaceae	0.0004164	0.0001434	0.0002191	9.32E-05	0.004586	0.01474	-0.0003	-9.40E-05	-0.0002
p__Aquificae	g__Thermovibrio	0.0002075	0.000188	2.10E-05	3.49E-05	0.000572	0.003709	-0.00031	-8.80E-05	-0.00019
p__Aquificae	g__Thermosulfidibacter	0.0004064	9.40E-05	0.0002832	0.000203	0.009108	0.02457	-0.00024	2.14E-05	-0.00012
p__Aquificae	g__Hydrogenivirga	3.38E-05	3.05E-05	1.08E-05	9.73E-06	0.03096	0.06485	-4.19E-05	-5.65E-06	-2.30E-05
p__Aquificae	g__Hydrogenobaculum	4.52E-06	7.75E-06	0	0	0.03498	0.07215	-9.56E-06	-4.73E-07	-4.52E-06
p__Balneolaeota	g__unclassified_f__Balneolaceae	0.003005	0.0006007	0.001747	0.000275	0.00033	0.002769	-0.00165	-0.00087	-0.00126
p__Balneolaeota	g__Balneola	0.001874	0.0006292	0.001265	0.000537	0.02575	0.05574	-0.0011	-0.00013	-0.00061
p__Balneolaeota	g__Gracilimonas	0.0006863	0.0001344	0.0005116	0.000232	0.02113	0.04755	-0.00032	-1.57E-05	-0.00017
p__Caldiserica	g__unclassified_p__Caldiserica	0.001598	0.0005737	0.0006738	0.000233	0.001008	0.00518	-0.00132	-0.00055	-0.00092
p__Caldiserica	g__Caldisericum	0.0001259	8.15E-05	2.05E-05	1.77E-05	0.000182	0.002329	-0.00016	-6.26E-05	-0.00011
p__Calditrichaeota	g__unclassified_p__Calditrichaeota	0.02085	0.002911	0.01761	0.001293	0.002202	0.008721	-0.00513	-0.00146	-0.00323
p__Calditrichaeota	g__unclassified_c__Calditrichae	0.0006901	0.000276	0.0004178	0.000172	0.02113	0.04755	-0.00046	-9.98E-05	-0.00027
p__candidate_division_	g__unclassified_p__candidate_divisio	5.82E-05	5.15E-05	1.90E-05	1.83E-05	0.01722	0.0402	-7.38E-05	-1.19E-05	-3.93E-05
p__candidate_division_	g__unclassified_p__candidate_divisio	0.0007267	0.0003569	9.38E-05	6.38E-05	0.000183	0.002329	-0.00087	-0.00043	-0.00063
p__candidate_division_	g__unclassified_p__candidate_divisio	0.0002216	9.63E-05	0.0001261	4.31E-05	0.01726	0.0402	-0.00016	-3.35E-05	-9.56E-05
p__candidate_division_	g__unclassified_p__candidate_divisio	0.03095	0.004395	0.02466	0.004436	0.01726	0.0402	-0.01038	-0.0029	-0.00629
p__candidate_division_	g__Candidatus_Methylomirabilis	0.0141	0.001974	0.009034	0.000543	0.000183	0.002329	-0.00621	-0.00393	-0.00507
p__Candidatus_Aeroph	g__unclassified_p__Candidatus_Aero	0.001498	0.0002429	0.001085	0.000222	0.004586	0.01474	-0.00062	-0.00021	-0.00041
p__Candidatus_Atribac	g__unclassified_p__Candidatus_Atrib	0.002437	0.0004918	0.00114	0.000271	0.000183	0.002329	-0.00161	-0.00096	-0.0013
p__Candidatus_Atribac	g__Candidatus_Caldatribacterium	0.0004801	0.0001362	0.000302	9.32E-05	0.002827	0.01032	-0.00027	-7.99E-05	-0.00018
p__Candidatus_Aureab	g__unclassified_p__Candidatus_Aure	0.001403	0.000494	0.0008278	0.000369	0.01726	0.0402	-0.00096	-0.00023	-0.00058
p__Candidatus_Azamba	g__unclassified_p__Candidatus_Azam	0.001718	0.0003795	0.001173	0.000454	0.02113	0.04755	-0.00089	-0.00021	-0.00055
p__Candidatus_Berkelb	g__unclassified_p__Candidatus_Berke	0.0002066	6.16E-05	9.13E-05	2.34E-05	0.000183	0.002329	-0.00015	-7.85E-05	-0.00012
p__Candidatus_Bipolar	g__Candidatus_Bipolaricaulis	0.0006384	0.0002097	0.0004006	0.000224	0.03764	0.07496	-0.00044	-5.58E-05	-0.00024
p__Candidatus_Calesca	g__unclassified_p__Candidatus_Cales	5.93E-05	3.58E-05	3.31E-05	1.62E-05	0.04515	0.08665	-5.28E-05	-7.47E-06	-2.62E-05
p__Candidatus_Chishol	g__unclassified_p__Candidatus_Chish	0.0001412	9.14E-05	5.24E-05	1.76E-05	0.001008	0.00518	-0.00015	-3.90E-05	-8.88E-05
p__Candidatus_Cloacin	g__Candidatus_Cloacimonas	0.001205	0.0004962	0.0006362	0.000273	0.002827	0.01032	-0.00091	-0.00022	-0.00057
p__Candidatus_Coatesb	g__unclassified_p__Candidatus_Coate	0.0002948	7.59E-05	0.0001292	4.71E-05	0.000246	0.00248	-0.00022	-0.00011	-0.00017
p__Candidatus_Collierb	g__unclassified_p__Candidatus_Colli	0.00015	8.46E-05	7.65E-05	4.03E-05	0.03121	0.06485	-0.00013	-2.11E-05	-7.35E-05
p__Candidatus_Colwell	g__unclassified_p__Candidatus_Colw	0.00011	6.55E-05	3.75E-05	3.06E-05	0.007285	0.02069	-0.00011	-3.24E-05	-7.25E-05
p__Candidatus_Curtissb	g__unclassified_p__Candidatus_Curti	0.0006604	0.0002416	0.0002491	0.000103	0.00033	0.002769	-0.00058	-0.00026	-0.00041
p__Candidatus_Dadaba	g__unclassified_p__Candidatus_Dada	1.653	0.7481	0.8803	0.4259	0.01133	0.02909	-1.251	-0.2403	-0.7732
p__Candidatus_Delongb	g__unclassified_p__Candidatus_Delon	8.91E-05	4.33E-05	5.51E-05	2.43E-05	0.03121	0.06485	-6.74E-05	-5.38E-06	-3.41E-05
p__Candidatus_Desanti	g__unclassified_p__Candidatus_Desa	0.0004597	0.0001167	0.0002328	0.000116	0.001315	0.006216	-0.00032	-0.00012	-0.00023
p__Candidatus_Dormib	g__unclassified_p__Candidatus_Dorm	0.004042	0.0006926	0.003131	0.000683	0.01402	0.03427	-0.00149	-0.00033	-0.00091
p__Candidatus_Eisenba	g__unclassified_p__Candidatus_Eisen	0.04244	0.006797	0.03523	0.005377	0.03764	0.07496	-0.01219	-0.00222	-0.00721
p__Candidatus_Eremiol	g__unclassified_p__Candidatus_Erem	0.0008327	0.0001924	0.0005724	0.000141	0.003611	0.01236	-0.00041	-0.00012	-0.00026
p__Candidatus_Falkow	g__unclassified_p__Candidatus_Falko	0.002968	0.00089	0.0007653	0.000138	0.000183	0.002329	-0.00272	-0.00174	-0.0022
p__Candidatus_Fermen	g__Candidatus_Fermentibacter	0.0002356	9.50E-05	0.0001192	4.37E-05	0.007285	0.02069	-0.00018	-5.70E-05	-0.00012
p__Candidatus_Firestor	g__unclassified_p__Candidatus_Firest	0.0002217	5.73E-05	9.28E-05	3.34E-05	0.00044	0.003169	-0.00016	-8.80E-05	-0.00013
p__Candidatus_Fraserb	g__unclassified_p__Candidatus_Frase	0.001756	0.0002665	0.001372	0.000282	0.01133	0.02909	-0.00063	-0.00017	-0.00038
p__Candidatus_Gottesn	g__unclassified_p__Candidatus_Gotte	0.00138	0.0002747	0.001012	0.00027	0.007285	0.02069	-0.00061	-0.00015	-0.00037
p__Candidatus_Hydrog	g__unclassified_p__Candidatus_Hydr	0.02258	0.004481	0.01517	0.001943	0.000246	0.00248	-0.01029	-0.00469	-0.00741
p__Candidatus_Hydrog	g__Candidatus_Hydrogenedens	0.00105	0.0001978	0.0008562	0.000187	0.04515	0.08665	-0.00035	-4.57E-05	-0.00019
p__Candidatus_Jorgens	g__unclassified_p__Candidatus_Jorge	0.001361	0.0005842	0.0008189	0.000457	0.02575	0.05574	-0.00101	-0.00015	-0.00054
p__Candidatus_Kaiserb	g__unclassified_p__Candidatus_Kaise	0.002998	0.0004231	0.002518	0.000475	0.03764	0.07496	-0.00085	-0.00012	-0.00048

p__Candidatus_Komeil	g__unclassified_p__Candidatus_Kom	0.0001389	7.17E-05	4.84E-05	3.35E-05	0.002827	0.01032	-0.00014	-4.61E-05	-9.05E-05
p__Candidatus_Krypton	g__Candidatus_Chrysopegis	0.0003871	0.0002202	3.38E-05	2.86E-05	0.000183	0.002329	-0.00048	-0.00023	-0.00035
p__Candidatus_Krypton	g__unclassified_p__Candidatus_Kryp	7.28E-05	5.15E-05	2.49E-05	1.57E-05	0.01726	0.0402	-7.92E-05	-1.93E-05	-4.79E-05
p__Candidatus_Levyba	g__unclassified_p__Candidatus_Levy	0.0008177	0.0002398	0.0005432	8.32E-05	0.003611	0.01236	-0.00042	-0.00013	-0.00027
p__Candidatus_Lindow	g__unclassified_p__Candidatus_Lind	0.0008066	0.0001653	0.0005846	0.000156	0.009108	0.02457	-0.00036	-9.24E-05	-0.00022
p__Candidatus_Lloydba	g__unclassified_p__Candidatus_Lloy	0.0004016	0.0001473	0.0002139	7.63E-05	0.007285	0.02069	-0.00029	-8.94E-05	-0.00019
p__Candidatus_Magasa	g__unclassified_p__Candidatus_Maga	0.001682	0.0003708	0.001012	0.000258	0.001315	0.006216	-0.00094	-0.00039	-0.00067
p__Candidatus_Marguli	g__unclassified_c__Candidatus_Mari	0.0001311	5.94E-05	4.09E-05	2.82E-05	0.001008	0.00518	-0.00013	-5.12E-05	-9.02E-05
p__Candidatus_Melaina	g__unclassified_p__Candidatus_Mela	0.07775	0.0715	0.01514	0.002543	0.005795	0.0177	-0.1057	-0.02183	-0.0626
p__Candidatus_Melaina	g__unclassified_o__Candidatus_Gastr	0.0002721	0.0001835	1.32E-05	1.03E-05	0.000182	0.002329	-0.00037	-0.00015	-0.00026
p__Candidatus_Melaina	g__unclassified_o__Candidatus_Caen	0.0003354	0.0001572	8.10E-05	3.25E-05	0.000183	0.002329	-0.00036	-0.00017	-0.00025
p__Candidatus_Moranb	g__unclassified_p__Candidatus_Mora	0.001492	0.0004685	0.001132	0.000186	0.03121	0.06485	-0.00067	-0.0001	-0.00036
p__Candidatus_Nealson	g__unclassified_p__Candidatus_Neals	0.03035	0.006436	0.01888	0.002525	0.000583	0.003709	-0.01564	-0.0076	-0.01146
p__Candidatus_Omnitro	g__unclassified_p__Candidatus_Omni	0.02435	0.001801	0.02049	0.001807	0.000583	0.003709	-0.00543	-0.00249	-0.00386
p__Candidatus_Paceba	g__unclassified_p__Candidatus_Pace	0.0005536	0.0009425	0.0001512	6.86E-05	0.04515	0.08665	-0.00102	-4.52E-05	-0.0004
p__Candidatus_Parcuba	g__unclassified_p__Candidatus_Parcu	0.0007692	0.000109	0.0004738	9.91E-05	0.00033	0.002769	-0.00038	-0.0002	-0.0003
p__Candidatus_Ryanba	g__unclassified_p__Candidatus_Ryan	0.0008413	0.0002198	0.0002888	6.10E-05	0.000183	0.002329	-0.00069	-0.00043	-0.00055
p__Candidatus_Schekm	g__unclassified_p__Candidatus_Sche	0.003548	0.0008215	0.002298	0.000451	0.001315	0.006216	-0.00183	-0.00072	-0.00125
p__Candidatus_Shapiro	g__unclassified_p__Candidatus_Shap	0.0002727	0.0002292	5.58E-05	3.32E-05	0.000583	0.003709	-0.00038	-0.00011	-0.00022
p__Candidatus_Sungba	g__unclassified_p__Candidatus_Sung	0.0005181	0.0001153	0.0003321	0.000117	0.002827	0.01032	-0.00028	-8.94E-05	-0.00019
p__Candidatus_Taylorb	g__unclassified_p__Candidatus_Taylo	0.0029	0.001077	0.001666	0.000876	0.01402	0.03427	-0.00208	-0.00041	-0.00123
p__Candidatus_Terryba	g__unclassified_p__Candidatus_Terry	0.0005219	0.0001965	0.0002581	7.93E-05	0.002827	0.01032	-0.00038	-0.00013	-0.00026
p__Candidatus_Uhrbact	g__unclassified_p__Candidatus_Uhrb	0.008004	0.008654	0.001384	0.000224	0.000183	0.002329	-0.01211	-0.00249	-0.00662
p__Candidatus_Veblen	g__unclassified_p__Candidatus_Vebl	1.91E-05	1.20E-05	8.10E-06	6.47E-06	0.03096	0.06485	-1.86E-05	-3.21E-06	-1.10E-05
p__Candidatus_Woeseb	g__unclassified_p__Candidatus_Woes	0.003813	0.001052	0.001793	0.000363	0.000183	0.002329	-0.00266	-0.0014	-0.00202
p__Candidatus_Wolfeb	g__unclassified_p__Candidatus_Wolf	0.0004004	0.0001557	0.0002547	0.000127	0.04515	0.08665	-0.00026	-3.38E-05	-0.00015
p__Candidatus_Yanofs	g__unclassified_p__Candidatus_Yano	0.0007307	0.0002606	0.0002579	6.17E-05	0.000183	0.002329	-0.00063	-0.00031	-0.00047
p__Candidatus_Zambry	g__unclassified_p__Candidatus_Zamb	0.00334	0.001386	0.001595	0.000866	0.005795	0.0177	-0.00278	-0.00081	-0.00175
p__Chlamydiae	g__Parachlamydia	0.02584	0.01419	0.002401	0.000818	0.000183	0.002329	-0.03263	-0.01574	-0.02343
p__Chlamydiae	g__unclassified_p__Chlamydiae	0.01983	0.008572	0.004168	0.000629	0.000183	0.002329	-0.02093	-0.01096	-0.01567
p__Chlamydiae	g__unclassified_o__Chlamydiales	0.01463	0.008068	0.001498	0.000416	0.000183	0.002329	-0.01893	-0.00879	-0.01314
p__Chlamydiae	g__Candidatus_Protochlamydia	0.008437	0.004437	0.001696	0.000336	0.000183	0.002329	-0.00982	-0.00442	-0.00674
p__Chlamydiae	g__Neochlamydia	0.005029	0.002433	0.0008633	0.000237	0.000183	0.002329	-0.00564	-0.00284	-0.00417
p__Chlamydiae	g__Waddlia	0.002408	0.001264	0.0003569	0.000115	0.000183	0.002329	-0.00285	-0.00137	-0.00205
p__Chlamydiae	g__unclassified_f__Parachlamydiacea	0.00201	0.001073	0.0003962	0.000138	0.000183	0.002329	-0.00225	-0.00103	-0.00161
p__Chlamydiae	g__Chlamydia	0.002608	0.0008167	0.001144	0.000177	0.000183	0.002329	-0.00195	-0.00101	-0.00146
p__Chlamydiae	g__Criblamydia	0.001805	0.0008636	0.0003619	0.000103	0.000183	0.002329	-0.00196	-0.00095	-0.00144
p__Chlamydiae	g__unclassified_f__Waddliaceae	0.00143	0.0007381	0.0003037	0.00013	0.000183	0.002329	-0.00163	-0.00073	-0.00113
p__Chlamydiae	g__Candidatus_Rubidus	0.001185	0.0006096	0.0001503	5.95E-05	0.000183	0.002329	-0.00142	-0.00071	-0.00104
p__Chlamydiae	g__Estrella	0.001205	0.0005766	0.0003151	8.39E-05	0.000183	0.002329	-0.00124	-0.00057	-0.00089
p__Chlamydiae	g__Simkania	0.0004245	0.0001305	0.0001106	5.38E-05	0.000183	0.002329	-0.0004	-0.00023	-0.00031
p__Chlamydiae	g__unclassified_o__Anoxychlamydial	0.0003666	0.0001481	0.0001577	5.28E-05	0.000583	0.003709	-0.00031	-0.00012	-0.00021
p__Chlamydiae	g__Candidatus_Rhabdochlamydia	0.000263	8.78E-05	0.0001644	5.16E-05	0.03764	0.07496	-0.00016	-3.56E-05	-9.87E-05
p__Chlamydiae	g__unclassified_c__Chlamydiia	0.0001258	5.23E-05	3.92E-05	2.14E-05	0.000769	0.004362	-0.00012	-5.16E-05	-8.66E-05
p__Chlorobi	g__Pelodictyon	0.002227	0.0005619	0.001092	0.000477	0.000769	0.004362	-0.00156	-0.00066	-0.00114
p__Chlorobi	g__unclassified_f__Chlorobiaceae	0.0003331	0.0002474	7.86E-05	3.09E-05	0.000183	0.002329	-0.00042	-0.00014	-0.00025
p__Chrysiogenetes_d__	g__unclassified_o__Chrysiogenales	0.0005154	0.0002929	0.0001671	6.77E-05	0.001706	0.007373	-0.00054	-0.00018	-0.00035
p__Deferribacteres	g__Denitrovibrio	0.0003114	0.0001606	7.70E-05	4.55E-05	0.00044	0.003169	-0.00034	-0.00014	-0.00023

p__Deferribacteres	g__Mucispirillum	0.0001062	7.38E-05	3.07E-05	1.99E-05	0.001706	0.007373	-0.00012	-3.51E-05	-7.54E-05
p__Deferribacteres	g__Calditerrivibrio	0.0001847	4.15E-05	0.0001407	4.73E-05	0.03764	0.07496	-7.91E-05	-6.70E-06	-4.40E-05
p__Deferribacteres	g__Flexistipes	2.81E-05	2.18E-05	7.04E-06	1.70E-05	0.006253	0.01898	-3.84E-05	-4.48E-06	-2.11E-05
p__Deinococcus-Therm	g__Deinococcus	0.02191	0.003154	0.01628	0.001458	0.000246	0.00248	-0.00779	-0.00364	-0.00563
p__Deinococcus-Therm	g__Marinithermus	0.002068	0.000922	0.0005538	7.45E-05	0.000183	0.002329	-0.00209	-0.00101	-0.00151
p__Deinococcus-Therm	g__Thermus	0.003557	0.0003869	0.002859	0.00037	0.001315	0.006216	-0.001	-0.00037	-0.0007
p__Deinococcus-Therm	g__Oceanithermus	0.001544	0.0002708	0.001224	0.000212	0.009108	0.02457	-0.00052	-0.00012	-0.00032
p__Elusimicrobia	g__unclassified_p__Elusimicrobia	0.02073	0.002525	0.0143	0.001307	0.000183	0.002329	-0.00825	-0.00485	-0.00642
p__Elusimicrobia	g__Endomicrobium	3.23E-05	3.13E-05	7.21E-06	1.19E-05	0.01072	0.02883	-4.58E-05	-5.55E-06	-2.51E-05
p__Fibrobacteres	g__Chitinispirillum	0.0002052	7.28E-05	8.75E-05	4.94E-05	0.002827	0.01032	-0.00017	-6.57E-05	-0.00012
p__Fusobacteria	g__Fusobacterium	0.000236	8.18E-05	0.0001596	4.80E-05	0.02575	0.05574	-0.00013	-1.74E-05	-7.64E-05
p__Fusobacteria	g__Psychrilyobacter	3.49E-05	3.83E-05	2.23E-06	4.84E-06	0.000903	0.005107	-5.80E-05	-1.24E-05	-3.26E-05
p__Gemmatimonadetes	g__Gemmatimonas	0.03754	0.01049	0.02429	0.003645	0.001706	0.007373	-0.02012	-0.00655	-0.01325
p__Gemmatimonadetes	g__Longimicrobium	0.009552	0.002202	0.006362	0.000947	0.002202	0.008721	-0.00453	-0.00177	-0.00319
p__Gemmatimonadetes	g__unclassified_f__Gemmatimonadac	0.003127	0.0009305	0.001925	0.000386	0.01133	0.02909	-0.0018	-0.00062	-0.0012
p__Ignavibacteriae	g__Melioribacter	0.0004697	0.0002175	0.0002534	9.63E-05	0.007285	0.02069	-0.00038	-7.16E-05	-0.00022
p__Ignavibacteriae	g__unclassified_f__Melioribacteracea	0.0003063	0.0001624	0.0001532	7.07E-05	0.03764	0.07496	-0.00027	-5.16E-05	-0.00015
p__Kiritimatiellaeota	g__unclassified_f__Kiritimatiellaceae	0.0003765	9.32E-05	0.000272	7.57E-05	0.02113	0.04755	-0.00017	-3.59E-05	-0.0001
p__Lentisphaerae	g__Victivallis	0.0006498	0.0004635	0.0002253	6.92E-05	0.003611	0.01236	-0.00073	-0.00018	-0.00042
p__Lentisphaerae	g__unclassified_o__Lentisphaerales	0.0009843	0.0002037	0.0005782	0.00014	0.00044	0.003169	-0.00056	-0.00026	-0.00041
p__Lentisphaerae	g__Lentisphaera	0.0004639	0.0002087	0.0002903	0.000213	0.04515	0.08665	-0.00033	7.19E-06	-0.00017
p__Rhodothermaeota	g__Rubrivirga	0.003192	0.0009827	0.001829	0.000198	0.000246	0.00248	-0.00194	-0.00081	-0.00136
p__Rhodothermaeota	g__unclassified_o__Rhodothermales	0.002727	0.000742	0.002003	0.000305	0.03121	0.06485	-0.0012	-0.00021	-0.00072
p__Rhodothermaeota	g__Rubricoccus	0.001169	0.0002854	0.0008576	0.000321	0.04515	0.08665	-0.00055	-5.50E-05	-0.00031
p__Spirochaetes	g__unclassified_p__Spirochaetes	0.0148	0.002235	0.008446	0.000979	0.000183	0.002329	-0.00775	-0.00491	-0.00636
p__Spirochaetes	g__unclassified_f__Leptospiraceae	0.00152	0.001035	0.0003884	0.000222	0.000583	0.003709	-0.00178	-0.00058	-0.00113
p__Spirochaetes	g__Treponema	0.0026	0.0003798	0.001538	0.000315	0.000246	0.00248	-0.00133	-0.00077	-0.00106
p__Spirochaetes	g__Leptonema_f__Leptospiraceae	0.001458	0.0006491	0.0007931	0.000212	0.002827	0.01032	-0.00109	-0.00027	-0.00067
p__Spirochaetes	g__Turneriella	0.0007777	0.0003463	0.000204	7.67E-05	0.000183	0.002329	-0.00082	-0.00038	-0.00057
p__Spirochaetes	g__Spirochaeta	0.0008039	0.000244	0.0004325	0.000102	0.000583	0.003709	-0.00052	-0.00022	-0.00037
p__Spirochaetes	g__Sphaerochaeta	0.0001523	0.0001069	6.91E-05	4.02E-05	0.02113	0.04755	-0.00016	-1.98E-05	-8.32E-05
p__Spirochaetes	g__Exilispira	7.69E-05	3.80E-05	1.21E-05	1.32E-05	0.000163	0.002329	-9.02E-05	-4.15E-05	-6.48E-05
p__Spirochaetes	g__Rectinema	7.06E-05	5.65E-05	8.64E-06	7.57E-06	0.000572	0.003709	-9.83E-05	-3.02E-05	-6.19E-05
p__Spirochaetes	g__unclassified_o__Brevinematales	6.66E-05	4.30E-05	2.11E-05	1.41E-05	0.007263	0.02069	-7.47E-05	-2.15E-05	-4.55E-05
p__Spirochaetes	g__Borreliella	4.29E-05	4.03E-05	1.32E-06	2.98E-06	0.003198	0.01162	-6.82E-05	-2.06E-05	-4.16E-05
p__Synergistetes	g__unclassified_p__Synergistetes	0.001635	0.001404	0.000305	0.000141	0.000183	0.002329	-0.00227	-0.00079	-0.00133
p__Synergistetes	g__unclassified_o__Synergistales	0.0002496	8.64E-05	8.50E-05	2.31E-05	0.000183	0.002329	-0.00022	-0.00012	-0.00016
p__Synergistetes	g__Acetomicrobium	0.0001274	7.95E-05	6.39E-05	3.06E-05	0.04515	0.08665	-0.00012	-1.49E-05	-6.35E-05
p__Synergistetes	g__Dethiosulfovibrio	8.32E-05	3.63E-05	3.10E-05	1.06E-05	0.000246	0.00248	-7.77E-05	-3.11E-05	-5.23E-05
p__Synergistetes	g__Fretibacterium	7.58E-05	5.15E-05	3.06E-05	1.84E-05	0.03121	0.06485	-7.79E-05	-1.52E-05	-4.52E-05
p__Synergistetes	g__Aminomonas	4.93E-05	1.79E-05	1.52E-05	1.18E-05	0.001309	0.006216	-4.56E-05	-2.11E-05	-3.40E-05
p__Synergistetes	g__Cloacibacillus	4.53E-05	3.63E-05	1.14E-05	1.31E-05	0.02094	0.04755	-5.66E-05	-1.34E-05	-3.39E-05
p__Synergistetes	g__Jonquetella	2.99E-05	2.84E-05	3.94E-06	4.88E-06	0.0423	0.0839	-4.38E-05	-9.66E-06	-2.59E-05
p__Thermodesulfobacte	g__unclassified_o__Thermodesulfoba	0.1225	0.05433	0.06802	0.03179	0.01133	0.02909	-0.08911	-0.01784	-0.05447
p__Thermodesulfobacte	g__unclassified_p__Thermodesulfoba	0.0006953	0.0001625	0.0004883	0.000128	0.007285	0.02069	-0.00033	-7.78E-05	-0.00021
p__Thermotogae	g__Petrotoga	0.001599	0.0005786	0.0009889	0.000477	0.02113	0.04755	-0.00105	-0.00017	-0.00061
p__Thermotogae	g__Thermotoga	0.0006351	0.0002475	0.0004087	8.90E-05	0.02575	0.05574	-0.00037	-6.70E-05	-0.00023
p__Thermotogae	g__Fervidobacterium	0.000262	0.0002321	6.37E-05	2.75E-05	0.01726	0.0402	-0.00034	-8.10E-05	-0.0002

p__Thermotogae	g__Thermosipho_f__Fervidobacteriac	0.0001571	8.01E-05	3.28E-05	2.46E-05	0.00044	0.003169	-0.00017	-7.65E-05	-0.00012
p__Thermotogae	g__unclassified_o__Thermotogales	3.47E-05	2.50E-05	1.05E-05	7.96E-06	0.001706	0.007373	-4.22E-05	-1.04E-05	-2.42E-05
p__unclassified_d__Bac	g__Candidatus_Stahlbacteria	0.0007647	0.0001683	0.0005093	0.000156	0.009108	0.02457	-0.00039	-0.00012	-0.00026
p__unclassified_d__Bac	g__Candidatus_Aegiribacteria	0.0002233	0.0001456	0.0001076	7.56E-05	0.03764	0.07496	-0.00021	-2.19E-05	-0.00012
p__unclassified_d__Bac	g__Candidatus_Chazhemtobacterium	7.60E-05	9.28E-05	4.21E-06	7.12E-06	0.000441	0.003172	-0.00013	-2.49E-05	-7.18E-05

Taxa significantlly decreased under blue light when compared to white light	Proportions of the reduced functional genes related to biodeterioration (that is, carbon fixation, denitrification, and dissimilatory sulfate reduction) contributed by the significantly decreased taxa after blue light illuminating										
	Sample_1	Sample_2	Sample_3	Sample_4	Sample_5	Sample_6	Sample_7	Sample_8	Sample_9	Sample_10	AVE
**Cyanobacteria**	0.0%	0.0%	0.0%	0.0%	0.0%	0.0%	0.0%	0.0%	0.0%	0.0%	**0.0%**
**Acidobacteria**	0.0%	0.0%	0.0%	0.0%	0.0%	0.0%	0.0%	0.0%	0.0%	0.0%	**0.0%**
**Actinobacteria**	0.1%	0.2%	0.1%	0.2%	0.1%	0.1%	0.2%	0.2%	0.1%	0.1%	**0.1%**
**Bacteroidetes**	0.0%	0.0%	0.0%	0.0%	0.0%	0.0%	0.0%	0.0%	0.0%	0.0%	**0.0%**
**Chloroflexi**	0.0%	0.0%	0.0%	0.0%	0.0%	0.0%	0.0%	0.0%	0.0%	0.0%	**0.0%**
**Firmicutes**	0.0%	0.0%	0.0%	0.0%	0.0%	0.0%	0.0%	0.0%	0.0%	0.0%	**0.0%**
**Nitrospinae**	0.0%	0.0%	0.0%	0.0%	0.0%	0.0%	0.0%	0.0%	0.0%	0.0%	**0.0%**
**Planctomycetes**	0.0%	0.0%	0.0%	0.0%	0.0%	0.0%	0.0%	0.0%	0.0%	0.0%	**0.0%**
**Proteobacteria**	1.4%	1.3%	1.1%	1.1%	1.4%	1.3%	1.2%	1.1%	1.6%	1.1%	**1.3%**
**Verrucomicrobia**	0.0%	0.0%	0.0%	0.0%	0.0%	0.0%	0.0%	0.0%	0.0%	0.0%	**0.0%**
**Others**	0.0%	0.0%	0.0%	0.0%	0.0%	0.0%	0.0%	0.0%	0.0%	0.0%	**0.0%**

Taxa significantlly decreased under green light when compared to white light	Proportions of the reduced functional genes related to biodeterioration (that is, carbon fixation, denitrification, and dissimilatory sulfate reduction) contributed by the significantly decreased taxa after green light illuminating										
	Sample_1	Sample_2	Sample_3	Sample_4	Sample_5	Sample_6	Sample_7	Sample_8	Sample_9	Sample_10	AVE
**Cyanobacteria**	0.8%	0.7%	1.8%	1.0%	0.6%	1.3%	1.2%	0.9%	0.9%	1.5%	**1.1%**
**Acidobacteria**	0.0%	0.0%	0.0%	0.0%	0.0%	0.0%	0.0%	0.0%	0.0%	0.0%	**0.0%**
**Actinobacteria**	0.0%	0.0%	0.0%	0.0%	0.0%	0.0%	0.0%	0.0%	0.0%	0.0%	**0.0%**
**Bacteroidetes**	0.0%	0.0%	0.0%	0.0%	0.0%	0.0%	0.0%	0.0%	0.0%	0.0%	**0.0%**
**Chloroflexi**	0.0%	0.0%	0.0%	0.0%	0.0%	0.0%	0.0%	0.0%	0.0%	0.0%	**0.0%**
**Firmicutes**	0.0%	0.0%	0.0%	0.0%	0.0%	0.0%	0.0%	0.0%	0.0%	0.0%	**0.0%**
**Nitrospinae**	0.0%	0.0%	0.0%	0.0%	0.0%	0.0%	0.0%	0.0%	0.0%	0.0%	**0.0%**
**Planctomycetes**	0.0%	0.0%	0.0%	0.0%	0.0%	0.0%	0.0%	0.0%	0.0%	0.0%	**0.0%**
**Proteobacteria**	4.9%	4.2%	4.2%	4.0%	5.1%	4.5%	4.4%	3.8%	5.2%	4.4%	**4.5%**
**Verrucomicrobia**	0.0%	0.0%	0.0%	0.0%	0.0%	0.0%	0.0%	0.0%	0.0%	0.0%	**0.0%**
**Others**	0.0%	0.0%	0.0%	0.0%	0.0%	0.0%	0.0%	0.0%	0.0%	0.0%	**0.0%**

Taxa significantlly decreased under red light when compared to white light	Proportions of the reduced functional genes related to biodeterioration (that is, carbon fixation, denitrification, and dissimilatory sulfate reduction) contributed by the significantly decreased taxa after red light illuminating										
	Sample_1	Sample_2	Sample_3	Sample_4	Sample_5	Sample_6	Sample_7	Sample_8	Sample_9	Sample_10	AVE
**Cyanobacteria**	0.0%	0.0%	0.0%	0.0%	0.0%	0.0%	0.0%	0.0%	0.0%	0.0%	**0.0%**
**Acidobacteria**	0.0%	0.0%	0.0%	0.0%	0.0%	0.0%	0.0%	0.0%	0.0%	0.0%	**0.0%**
**Actinobacteria**	0.0%	0.0%	0.0%	0.0%	0.0%	0.0%	0.0%	0.0%	0.0%	0.0%	**<0.1%**
**Bacteroidetes**	0.0%	0.0%	0.0%	0.0%	0.0%	0.0%	0.0%	0.0%	0.0%	0.0%	**0.0%**
**Chloroflexi**	0.0%	0.0%	0.0%	0.0%	0.0%	0.0%	0.0%	0.0%	0.0%	0.0%	**0.0%**
**Firmicutes**	0.0%	0.0%	0.0%	0.0%	0.0%	0.0%	0.0%	0.0%	0.0%	0.0%	**0.0%**
**Nitrospinae**	0.0%	0.0%	0.0%	0.0%	0.0%	0.0%	0.0%	0.0%	0.0%	0.0%	**0.0%**
**Planctomycetes**	0.0%	0.0%	0.0%	0.0%	0.0%	0.0%	0.0%	0.0%	0.0%	0.0%	**0.0%**
**Proteobacteria**	3.9%	3.7%	3.6%	3.4%	3.8%	3.8%	3.7%	3.6%	4.1%	3.7%	**3.7%**
**Verrucomicrobia**	0.0%	0.0%	0.0%	0.0%	0.0%	0.0%	0.0%	0.0%	0.0%	0.0%	**0.0%**
**Others**	0.0%	0.0%	0.0%	0.0%	0.0%	0.0%	0.0%	0.0%	0.0%	0.0%	**0.0%**

Supplementary Table 6. The design of buffer and substrate for each enzyme measurement (n = 16).

Enzymes	Buffer	Substrate
β-glucosidase	2-[N-Morpholino]ethanesulfonic acid (MES, 0.1M, pH=6.1)	4-Methylumbelliferyl *β*-D-glucopyranoside
Cellobiohydrolase	2-[N-Morpholino]ethanesulfonic acid (MES, 0.1M, pH=6.1)	4-Methylumbelliferyl *β*-D-cellobioside
Xylanase	2-[N-Morpholino]ethanesulfonic acid (MES, 0.1M, pH=6.1)	4-Methylumbelliferyl-*β*-D-xylopyranoside
β-galactosidase	2-[N-Morpholino]ethanesulfonic acid (MES, 0.1M, pH=6.1)	4-Methylumbelliferyl *β*-D-galactopyranoside
Leucine amino peptidase	2-[N-Morpholino]ethanesulfonic acid (MES, 0.1M, pH=6.1)	L-Leucine-7-amido-4-methylcoumarin hydrochloride
β-N-acetylglucosaminidase	2-[N-Morpholino]ethanesulfonic acid (MES, 0.1M, pH=6.1)	4-Methylumbelliferyl N-acetyl-*β*-D-glucosaminide
N-acetyl-*β*-galactosaminidase	Trizma (Tris, 0.05M, pH=7.8)	4-Methylumbelliferyl N-acetyl-*β*-D-galactosaminide
Alkaline phosphatase	2-[N-Morpholino]ethanesulfonic acid (MES, 0.1M, pH=6.1)	4-Methylumbelliferyl phosphate
