# Supplementary material for: Innovative strategy for the conservation of a millennial mausoleum from biodeterioration through artificial light management
Source: NPJ Biofilms Microbiomes. 2023 Sep 23;9:69. doi: 10.1038/s41522-023-00438-9 (PMC10516906; doi:10.1038/s41522-023-00438-9)
Supplement: Supplementary file 2 — Reporting Summary [file 41522_2023_438_MOESM2_ESM.pdf]

nature portfolio

Corresponding author(s): Youzhi Feng and Yonghui Li

Last updated by author(s): Jul 26, 2023

Reporting Summary

Nature Portfolio wishes to improve the reproducibility of the work that we publish. This form provides structure for consistency and transparency in reporting. For further information on Nature Portfolio policies, see our Editorial Policies and the Editorial Policy Checklist.

Statistics

For all statistical analyses, confirm that the following items are present in the figure legend, table legend, main text, or Methods section.

n/a	Confirmed	
☐	☒	The exact sample size (n) for each experimental group/condition, given as a discrete number and unit of measurement
☐	☒	A statement on whether measurements were taken from distinct samples or whether the same sample was measured repeatedly
☐	☒	The statistical test(s) used AND whether they are one- or two-sided<br>*Only common tests should be described solely by name; describe more complex techniques in the Methods section.*
☐	☒	A description of all covariates tested
☐	☒	A description of any assumptions or corrections, such as tests of normality and adjustment for multiple comparisons
☐	☒	A full description of the statistical parameters including central tendency (e.g. means) or other basic estimates (e.g. regression coefficient) AND variation (e.g. standard deviation) or associated estimates of uncertainty (e.g. confidence intervals)
☐	☒	For null hypothesis testing, the test statistic (e.g. F, t, r) with confidence intervals, effect sizes, degrees of freedom and P value noted<br>*Give P values as exact values whenever suitable.*
☒	☐	For Bayesian analysis, information on the choice of priors and Markov chain Monte Carlo settings
☒	☐	For hierarchical and complex designs, identification of the appropriate level for tests and full reporting of outcomes
☒	☐	Estimates of effect sizes (e.g. Cohen's d, Pearson's r), indicating how they were calculated

Our web collection on statistics for biologists contains articles on many of the points above.

Software and code

Policy information about availability of computer code

Data collection	Samples with 2 years of exposure to different wavelengths of artificial lights were collected to reveal the related phototrophic bacteriome of this study.
Data analysis	For bioinformatic analysis, QIIME was used. The statistical analyses were performed using R 4.0.0 and the IBM Statistical Product and Service Solutions (SPSS) Statistics for Windows (Version 13).

For manuscripts utilizing custom algorithms or software that are central to the research but not yet described in published literature, software must be made available to editors and reviewers. We strongly encourage code deposition in a community repository (e.g. GitHub). See the Nature Portfolio guidelines for submitting code & software for further information.

Data

Policy information about availability of data

All manuscripts must include a data availability statement. This statement should provide the following information, where applicable:

- Accession codes, unique identifiers, or web links for publicly available datasets
- A description of any restrictions on data availability
- For clinical datasets or third party data, please ensure that the statement adheres to our policy

The amplicon sequences (accession number: DRA014568) and the shotgun metagenomics (DRA014569) were deposited in the DNA Data Bank of Japan (DDBJ)

database (https://www.ddbj.nig.ac.jp/index-e.html), respectively. All other data generated or analyzed during this study are included in this published article or are available from the corresponding author upon reasonable request.

Research involving human participants, their data, or biological material

Policy information about studies with human participants or human data. See also policy information about sex, gender (identity/presentation), and sexual orientation and race, ethnicity and racism.

Reporting on sex and gender	n/a
Reporting on race, ethnicity, or other socially relevant groupings	n/a
Population characteristics	n/a
Recruitment	n/a
Ethics oversight	n/a

Note that full information on the approval of the study protocol must also be provided in the manuscript.

Field-specific reporting

Please select the one below that is the best fit for your research. If you are not sure, read the appropriate sections before making your selection.

☐ Life sciences ☐ Behavioural & social sciences ☒ Ecological, evolutionary & environmental sciences

For a reference copy of the document with all sections, see nature.com/documents/nr-reporting-summary-flat.pdf

Ecological, evolutionary & environmental sciences study design

All studies must disclose on these points even when the disclosure is negative.

Study description	Four ear chambers in the back part of the tomb were selected for the artificial light irradiation experiment. Before this study, these ear chambers were illuminated with white LED light containing full wavelengths (400~700 nm). Three of these four ear chambers were randomly selected, and each was replaced by different wavelengths. They were blue LEDs peaking at ~455 nm, green at ~530 nm, and red at ~640 nm.
Research sample	Samples with 2 years of exposure to different wavelengths of artificial lights were collected on August 20, 2021 (10 for each, and a total of 40 samples were collected).
Sampling strategy	Samples were randomly scrape the colonies to avoid damage to the cultural relic.
Data collection	A sterile scalpel was carefully used to randomly scrape the colonies to avoid damage to the cultural relic. After collection, all the samples were immediately delivered to the laboratory on ice and frozen at -80 °C until further use.
Timing and spatial scale	August 20, 2021.
Data exclusions	No data were excluded.
Reproducibility	Information about the sample locations and methods used in this paper are in our material and methods.
Randomization	The Shunling imperial mausoleum is located in Nanjing city, Jiangsu Province, China (31°89′N, 118°74′E).
Blinding	n/a

Did the study involve field work? ☒ Yes ☐ No

Field work, collection and transport

Field conditions	The Shunling imperial mausoleum is located in Nanjing city, Jiangsu Province, China, which has a mean annual average temperature of 15.4°C and a mean annual precipitation of ?1106.5 mm. The tomb was built in 961 A.D. with a multichamber structure composed of brick. It is approximately 9 m below ground level, 21.90 m in length, 10.12 m in width, and 5.42 m in height, and is composed of one corridor with four ear chambers along each side. It was excavated by the Nanjing Museum in 1950, with more than 600 precious cultural relics unearthed, and opened to public visits in 1985.

Location	The Shunling imperial mausoleum is located in Nanjing city, Jiangsu Province, China (31°89′N, 118°74′E).
Access & import/export	Samples were collected by the authors in the locations with permit.
Disturbance	This study did not cause any disturbance.

Reporting for specific materials, systems and methods

We require information from authors about some types of materials, experimental systems and methods used in many studies. Here, indicate whether each material, system or method listed is relevant to your study. If you are not sure if a list item applies to your research, read the appropriate section before selecting a response.

Materials & experimental systems

n/a	Involved in the study
☒	☐ Antibodies
☒	☐ Eukaryotic cell lines
☒	☐ Palaeontology and archaeology
☒	☐ Animals and other organisms
☒	☐ Clinical data
☒	☐ Dual use research of concern
☒	☐ Plants

Methods

n/a	Involved in the study
☒	☐ ChIP-seq
☒	☐ Flow cytometry
☒	☐ MRI-based neuroimaging
